# Simple ruthenium-catalyzed reductive amination enables the synthesis of a broad range of primary amines

**DOI:** 10.1038/s41467-018-06416-6

**Published:** 2018-10-08

**Authors:** Thirusangumurugan Senthamarai, Kathiravan Murugesan, Jacob Schneidewind, Narayana V. Kalevaru, Wolfgang Baumann, Helfried Neumann, Paul C. J. Kamer, Matthias Beller, Rajenahally V. Jagadeesh

**Affiliations:** Leibniz-Institut für Katalyse e. V. an der Universität Rostock, Albert-Einstein-Str. 29a, 18059 Rostock, Germany

## Abstract

The production of primary benzylic and aliphatic amines, which represent essential feedstocks and key intermediates for valuable chemicals, life science molecules and materials, is of central importance. Here, we report the synthesis of this class of amines starting from carbonyl compounds and ammonia by Ru-catalyzed reductive amination using H_2_. Key to success for this synthesis is the use of a simple RuCl_2_(PPh_3_)_3_ catalyst that empowers the synthesis of >90 various linear and branched benzylic, heterocyclic, and aliphatic amines under industrially viable and scalable conditions. Applying this catalyst, −NH_2_ moiety has been introduced in functionalized and structurally diverse compounds, steroid derivatives and pharmaceuticals. Noteworthy, the synthetic utility of this Ru-catalyzed amination protocol has been demonstrated by upscaling the reactions up to 10 gram-scale syntheses. Furthermore, in situ NMR studies were performed for the identification of active catalytic species. Based on these studies a mechanism for Ru-catalyzed reductive amination is proposed.

## Abstract (web summary)

Synthesis of primary amines via operationally simple, inexpensive and environmentally friendly methodologies has high impact in industrial settings. Here, the authors show a reductive amination process involving a ruthenium catalyst, aldehydes/ketones, ammonia, and hydrogen that displays a remarkable scope of primary amine products.

## Introduction

The development of efficient catalytic reactions for the selective and sustainable synthesis of amines from readily available and inexpensive starting materials by utilizing abundant and green reagents continues to be an important goal of chemical research^1–6^. In particular, the development of simple and easily accessible catalysts for reductive aminations is highly important because these reactions allow for the cost-efficient production of different kinds of amines^7–30^. Among reductive aminations, the reaction of carbonyl compounds with ammonia in presence of molecular hydrogen to produce primary amines is of central importance and continues to be a major challenge^17–30^. In general, amines are essential chemicals used widely in many research areas and industrial productions related to chemistry, medicine, biology, and material science^1–6^. The majority of existing pharmaceuticals, agrochemicals, biomolecules, and natural products contain amine functionalities, which constitute key structural motifs and play vital roles in their functions^1–6^. Among different kinds of amines, primary benzylic and aliphatic amines constitute valuable fine and bulk chemicals, that serve as versatile feedstocks and key intermediates for advanced chemicals, life science molecules and polymers^1–34^. Regarding their synthesis, catalytic reductive amination of carbonyl compounds (aldehydes and ketones) with ammonia in presence of molecular hydrogen represents a waste-free process to access various linear and branched benzylic and aliphatic amines^17–27^. In addition, catalytic amination of alcohols with ammonia also constitutes a sustainable methodology to produce primary amines^35–38^. Apart from transition metal-catalyzed aminations, the Leuckart-Wallach reaction^39–41^ and reduction of oxime ethers with borane^42–44^ have also been applied. Noteworthy, selective introduction of primary amine moieties in functionalized compounds by utilizing ammonia constitutes a benign and economic methodology^17–27^. Ammonia, which is produced in >175 million tons per year scale, is considered to be an abundant and green chemical used enormously for the large scale production of urea and other fertilizers as well as various basic chemicals^45–50^. Although ammonia is used extensively for the production of simple molecules, its reactions still encounter common problems such as the requirement of high temperatures or pressures and low selectivity towards the formation of a single desired product^45–50^. Hence, the development of more active and selective catalysts for an effective utilization of ammonia, especially for its insertion in advanced and complex molecules, is highly demanded and challenging.

Reductive amination for the preparation of primary amines, especially in industry, is mainly carried out using heterogeneous catalysts^17–23^. Compared to heterogeneous catalysts, homogeneous catalysis for amination of structurally diverse molecules is less studied and remains challenging^24–27^. Transition metal-catalyzed reactions involving ammonia are often difficult to perform or do not even occur. This problem is mainly due to the deactivation of homogeneous catalysts by the formation of stable Werner-type ammine complexes as well as due to the harsh conditions required for the activation of ammonia. In addition, common problems in reductive aminations, such as over alkylation and reduction to the corresponding alcohols, also affect catalyst viability. In order to utilize ammonia successfully and to overcome these problems, there is a need to develop highly efficient homogeneous catalysts, which is the prime task of this investigation. To date, a few catalysts based on Rh-^24,25^, Ir-^25^ and Ru-^26,27^ complexes were reported for the preparation of primary amines from carbonyl compounds and ammonia using hydrogen. Initially, Beller and co-workers^24^ have reported a [Rh(COD)Cl]_2_-TPPTS catalyst system for the synthesis of simple primary amines from aldehydes and aqueous ammonia using NH_4_OAc as additive. Following this work, Rh[(dppb)(COD)]BF_4_ and [Rh(COD)Cl]_2_-BINAS catalysts were also applied^25^. Next, [Ir(COD)Cl]_2_-BINAS was found to be able to catalyze the amination of a few simple ketones with ammonia^25^. Regarding Ru-catalysts, RuHCl(CO)(PPh_3_)_3_-xantphos/-dppe in presence of Al(OTf)_3_ is known to catalyze the preparation of simple primary amines from ketones^26^. Recently, RuHCl(CO)(PPh_3_)_3_-(S,S)-f-binaphane^27^ in presence of NaPF_6_ or NH_4_I using NH_3_, as well as Ru(OAc)_2_-C_3_-TunePhos^30^ using NH_4_OAc have been used for enantioselective reductive amination of ketones to obtain chiral primary amines. These homogeneous catalysts, however, have only been applied in (enantioselective) reductive aminations of simple substrates and have not been used for the preparation of functionalized amines. Despite these advances, the design of simpler yet efficient homogeneous catalysts for the preparation of a broad range of structurally diverse primary amines is highly desired and continues to be an important task from both a research and an industry perspective.

In a lot of cases, homogeneous catalysts applied for challenging reactions and advanced organic synthesis operations are based on sophisticated or synthetically demanding metal complexes and ligands. However, a fundamental and economically important principle is that to achieve a convenient and practical chemical synthesis, the catalyst must be simple, effective and commercially available and/or easily accessible. In this regard, triphenylphosphine (PPh_3_)-based metal complexes are found to be expedient and advantageous for catalysis applications, since PPh_3_ is a stable and comparatively cheap ligand^51–55^. Among PPh_3_-based Ru-complexes, RuCl_2_(PPh_3_)_3_ is considered to be the simplest and least expensive one and is also commercially available. Interestingly, RuCl_2_(PPh_3_)_3_ is known to catalyze a number of organic reactions^56–62^. Herein we demonstrate that RuCl_2_(PPh_3_)_3_ is an efficient and highly selective homogeneous precatalyst for reductive amination, allowing the preparation of a variety of primary amines of industrial importance. By applying this Ru-precatalyst and starting from inexpensive and readily available carbonyl compounds (aldehydes, ketones), ammonia and molecular hydrogen, we undertook the synthesis of functionalized and structurally diverse linear and branched benzylic, heterocyclic, and aliphatic amines including drugs and steroid derivatives. Another objective is to demonstrate up-scaling of the homogeneous amination protocol to gram-scale syntheses. Furthermore, efforts were also made to identify catalytically active species and reaction intermediates by performing kinetic and in situ NMR investigations. Based on these studies, a plausible reaction mechanism is proposed.

## Results

### Selection of catalyst and reaction conditions

Reductive amination of benzaldehyde (1) to benzylamine (2) with ammonia using molecular hydrogen was chosen as a benchmark reaction. At first, in presence of PPh_3_ different metal precursors were tested. As shown in Table 1, the in situ generated Fe-, Co-, Mn-, Ni- and Cu-PPh_3_ complexes were not active for the formation of benzylamine (Table 1 entries 1–5). However, in situ generated Ru(II)-PPh_3_ complexes showed some activity and produced benzylamine in up to 40% yield (Table 1, entries 6 and 7).

**Table 1:** Reductive amination of benzaldehyde: activity of different catalysts

Reaction conditions: 0.5 mmol benzaldehyde, 2 mol% metal precursor, 6 mol% PPh_3_, 5–7 bar NH_3_, 40 bar H_2_ 1.5 mL *t*-amyl alcohol, 130 ^o^C, 24 h, GC yields using n-hexadecane as standard

*L* Ligand, *nd* not detected

After observing this reactivity, we next tested in situ generated Ru-complexes with differently substituted PPh_3_-type ligands as well as simple nitrogen ligands (L1–L10). Among these, Ru-catalysts containing either PPh_3_ or derivatives with electron donating groups in *para* position showed the highest activity (Table 2; entries 1,4,5). However, none of the tested nitrogen ligands (L7–L10) produced the desired product (Table 2, entries 7–10). Unfortunately, using in situ generated Ru-complexes the yield of benzylamine did not improve beyond 53% (Table 2).

**Table 2:** Reductive amination of benzaldehyde using ruthenium catalysts

Next we turned our interest to molecularly defined Ru-complexes. To our delight, the commercially available complexes RuCl_2_(PPh_3_)_3_ and RuCl_2_(PPh_3_)_4_ showed excellent activity and selectivity for the formation of benzylamine in 92–95% yields (Table 2, entries 11–12). Further, Ru(tris(4-methoxyphenyl)phosphine)_3_Cl_2_ and Ru(tris(4-chlorophenyl)phosphine)_3_Cl_2_ were also prepared and tested for their reactivity (Table 2; entries 13 and 14). The former displays similar activity compared to RuCl_2_(PPh_3_)_3_ (Table 2, entry 13), while the latter was less active (Table 2, entry 14), reflecting the same ligand trend observed in case of in situ generated complexes. In presence of highly active catalysts, we observed 4–7 % of benzyl alcohol (**3**) as the side-product (Table 2, entries 11–13). In case of less-active and or non-active catalysts, undesired side products such as N-benzylidenebenzylamine (**4**) and 2,4,5-triphenyl-2-imidazoline (**5**) were formed (Table 2, entries 1–10). Dibenzylamine (**6**) was not observed under any of these conditions.

### Kinetic investigations

**Fig. 1:**
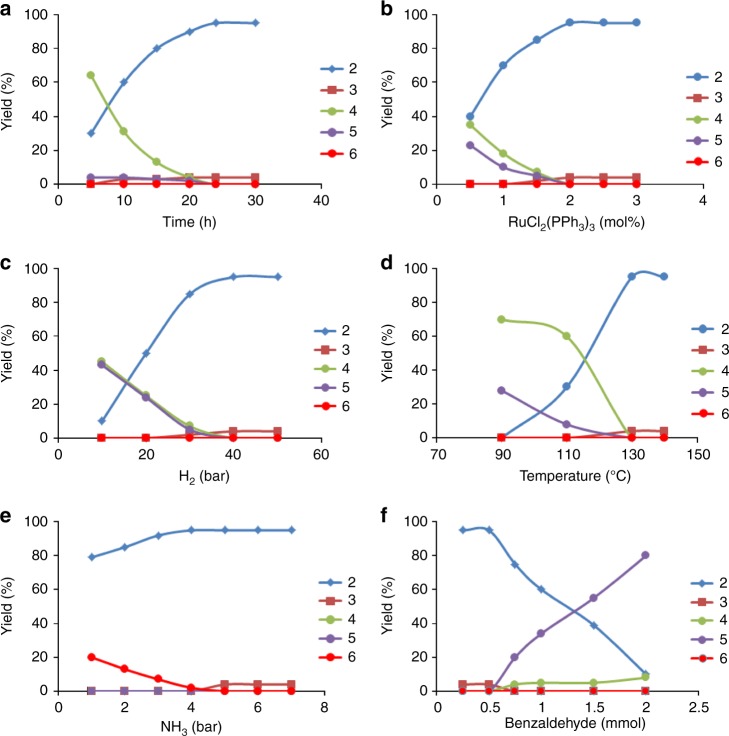
Kinetic investigations on the Ru-catalyzed reductive amination of benzaldehyde. **a** Yield vs reaction time, **b** yield vs concentration of RuCl_2_(PPh_3_)_3_, **c** yield vs pressure of H_2_, **d** yield vs temperature, **e** yield vs pressure of NH_3_, **f** yield vs concentration of benzaldehyde. 2 = Yield of benzylamine; 3 = yield of benzyl alcohol; 4 = yield of *N*-benzylidenebenzylamine; 5 = yield of 2,4,5-triphenyl-4,5-dihydro-1H-imidazole, 6 = yield of dibenzylamine. Reaction conditions: For Fig. 1a: 0.5 mmol benzaldehyde, 2 mol% RuCl_2_(PPh_3_)_3_, 5–7 bar NH_3_, 40 bar H_2_, 1.5 mL t-amyl alcohol, 130 ^o^C, 5–30 h; for Fig. 1b: 0.5 mmol benzaldehyde, 0.5–3 mol% RuCl_2_(PPh_3_)_3_, 5–7 bar NH_3_, 40 bar H_2_, 1.5 mL t-amyl alcohol, 130 ^o^C, 24 h; for Fig. 1c: 0.5 mmol benzaldehyde, 2 mol% RuCl_2_(PPh_3_)_3_, 5–7 bar NH_3_, 10–50 bar H_2_, 1.5 mL t-amyl alcohol, 130 ^o^C, 24 h; for Fig. 1d: 0.5 mmol benzaldehyde, 2 mol% RuCl_2_(PPh_3_)_3_, 5–7 bar NH_3_, 40 bar H_2_, 1.5 mL t-amyl alcohol, 90–140 ^o^C, 24 h. for Fig. 1e: 0.5 mmol benzaldehyde, 2 mol% RuCl_2_(PPh_3_)_3_, 1–7 bar NH_3_, 40 bar H_2_, 1.5 mL t-amyl alcohol, 130 ^o^C, 24 h; for Fig. 1f: 0.25–2 mmol benzaldehyde, 2 mol% RuCl_2_(PPh_3_)_3_, 5–7 bar NH_3_, 40 bar H_2_, 1.5 mL t-amyl alcohol, 130 ^o^C, 24 h. Yields were determined by GC using n-hexadecane as standard

After having identified RuCl_2_(PPh_3_)_3_ as one of the most active precatalysts, we performed kinetic investigations on this system and examined the effect of (a) reaction time, (b) catalyst concentration, (c) hydrogen pressure, (d) reaction temperature, (e) ammonia pressure, and (f) substrate (benzaldehyde) concentration on activity and product distribution (Fig. 1). For reaction time, in Fig. 1a it can be seen that after 5 h, secondary imine **4** is predominantly present (ca. 60%), with only 30% of target product **2**. Over the course of the reaction, **4**, which appears to be an intermediate, is consumed to yield up to 95% **2** after 24 h (for the mechanism of this transformation vide infra). During the reaction, an increasing amount (up to 4%) of benzyl alcohol is also formed. The cyclic side product **5** can be observed at various reaction times and its amount appears to decrease. This trend, however, is presumably an artifact of the kinetic measurements (see SI). From Fig. 1a it can be concluded that 24 h is an ideal reaction time to obtain maximum yield of **2**. Fig. 1b shows how catalyst loading affects the product distribution. At lower (<2 mol%) loadings, increased amounts of intermediate **4** and side product **5** are obtained, while beyond 2 mol% almost no **4** or **5** along with maximum yield of **2** and some benzyl alcohol **3** were observed. A catalyst loading of 2 mol% is therefore necessary to achieve excellent yield of benzylamine. Similar trends in the product distribution are observed for varied H_2_ pressure (Fig. 1c) and reaction temperature (Fig. 1d). Thus 40 bar H_2_ pressure and 130 °C reaction temperature are found to be optimum to suppress the formation of intermediates/side products (**4/5**) and to yield maximum amounts of the target product benzylamine. When investigating the effect of ammonia pressure, we found that at less than 5 bar side product **6** (dibenzylamine) is formed in up to 20% (Fig. 1e) yield with a concomitant decrease in the yield of **2**. Hence, a minimum NH_3_ pressure of 5 bar is required to selectively form the desired product **2** (for mechanistic details vide infra). Further, on increasing the concentration of benzaldehyde (>0.5 mmol) the amount of **5** gradually increases, leading to formation of **5** in up to 80% yield (for 2 mmol benzaldehyde) (Fig. 1e).

### Synthesis of linear primary amines from aldehydes

**Fig. 2:**
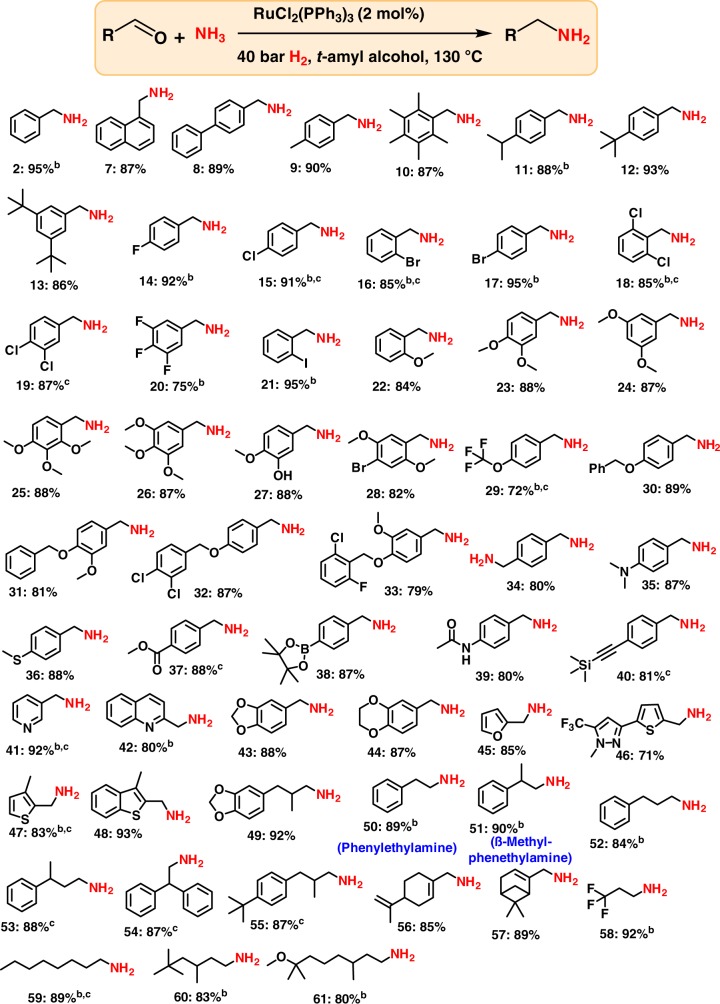
Ru-catalyzed synthesis of linear primary benzylic, heterocyclic, and aliphatic amines. ^a^Reaction conditions: ^a^0.5 mmol aldehyde, 2 mol% RuCl_2_(PPh_3_)_3_, 5–7 bar NH_3_, 40 bar H_2_ 1.5 mL *t*-amyl alcohol, 130 ^o^C, 24 h, isolated yields. ^b^GC yields using n-hexadecane as standard. ^c^same as ‘a’ for 30 h. Isolated as free amines and converted to hydrochloride salts. Corresponding hydrochloride salts were subjected to NMR analysis

Under optimized reaction conditions, we explored the scope of RuCl_2_(PPh_3_)_3_-catalyzed reductive amination for the synthesis of various primary amines. As shown in Fig. 2, industrially relevant and structurally diverse benzylic, heterocyclic, and aliphatic aldehydes underwent reductive amination and offered linear primary amines in good to excellent yields.

Simple as well as sterically hindered benzaldehydes were selectively converted to their corresponding benzyl amines in up to 95% yield (Fig. 2; products **2** and **7–13**). In order to apply this amination methodology for organic synthesis and drug discovery, achieving a high degree of chemoselectivity is important. In this regard, we conducted the reaction of sensitive halogenated and functionalized benzaldehydes. Delightfully, halogen-substituted benzaldehydes, including more sensitive iodo-substituted compounds, selectively underwent reductive amination without any significant dehalogenation (Fig. 2; products **14–21**). Gratifyingly, various functional groups such as ethers, thio-ethers, carboxylic acid-esters and boronic acid-esters, amides and challenging C–C triple bonds were all well-tolerated without being reduced (Fig. 2; products **22–40**). In all these cases, the aldehyde group was selectively aminated to produce functionalized amines in up to 88% yield.

Heterocycles are regarded as highly valuable compounds and these motifs serve as integral parts of a large number of life science molecules and natural products. Thus, the preparation of heterocyclic primary amines is routinely needed en route to the production of pharmaceutically and agriculturally valuable products. Consequently, a series of different heterocyclic amines were synthesized (Fig. 2; products **41–49**). The primary amines of pyridine, methylenedioxybenzene and benzodioxane, furan and thiophene were obtained in 87–92% yields.

Success in the amination of aromatic and heterocyclic aldehydes prompted us to validate this catalyst also for aliphatic substrates. Commonly, amination of aliphatic aldehydes is more challenging and most reported catalysts exhibit lower reactivity towards these substrates. In addition, the reaction of aliphatic aldehydes is often troubled by the formation of unwanted aldol reaction products. In spite of these problems, the RuCl_2_(PPh_3_)_3_ precatalyst is found to be highly active and selective for the preparation of aliphatic primary linear amines too (Fig. 2). Accordingly, various primary araliphatic and aliphatic linear amines including allylic ones (products **56** and **57**) were obtained in up to 92% yield. Importantly, phenylethylamines (products **50** and **51**), which function as monoaminergic neuromodulators and neurotransmitters in the human CNS, have been prepared in up to 90% yield.

### Synthesis of branched primary amines from ketones

**Fig. 3:**
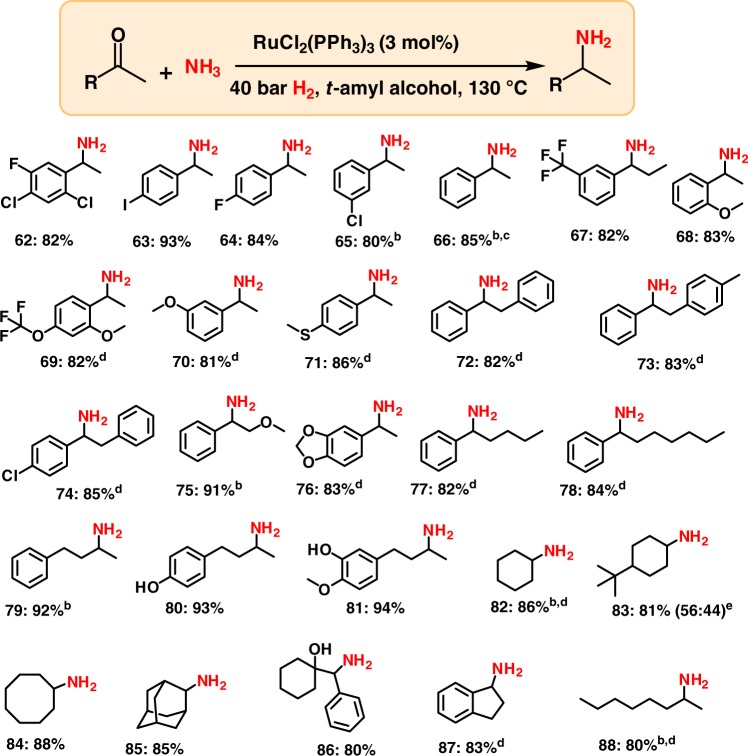
RuCl_2_(PPh_3_)_3_-catalyzed synthesis of branched primary amines from ketones. ^a^Reaction conditions: 0.5 mmol ketone, 3 mol% RuCl_2_(PPh_3_)_3_, 5–7 bar NH_3_, 40 bar H_2_ 1.5 mL *t*-amyl alcohol, 130 ^o^C, 24 h, isolated yields. ^b^GC yields using n-hexadecane as standard. ^c^Same as ‘a’ with 1 mol% catalyst. ^d^Same as ‘a’ for 30 h. ^e^Diastereomeric ratio. Isolated as free amines and converted to hydrochloride salts. Corresponding hydrochloride salts were subjected to NMR analysis

After having successfully performed the reductive amination of aldehydes, we were interested in the general applicability of this ruthenium precatalyst for the synthesis of branched primary amines starting from different ketones (Fig. 3). Compared to aldehydes, the reaction of ketones with ammonia to form primary amines is more difficult. Remarkably, the RuCl_2_(PPh_3_)_3_ precatalyst is also active towards aromatic ketones (Fig. 3). Further, the applicability of this catalyst system was also explored for aliphatic ketones. Here, the aliphatic branched primary amines were obtained in up to 94% yield (Fig. 3).

### Applications to life science molecules

**Fig. 4:**
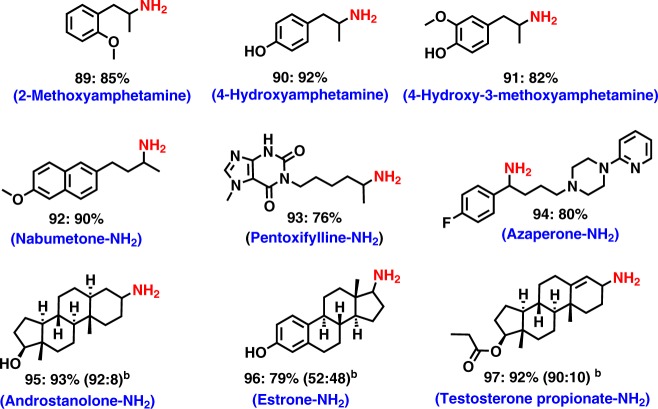
Synthesis of drugs and amination of complex molecules. ^a^Reaction conditions: 0.5 mmol ketone, 3 mol% RuCl_2_(PPh_3_)_3_, 5–7 bar NH_3_, 40 bar H_2_ 1.5 mL t-amyl alcohol, 130 ^o^C, 24 h, isolated yields. Isolated as free amines and converted to hydrochloride salts. Corresponding hydrochloride salts were subjected to NMR analysis. ^b^Diastereomeric ratio

To showcase the valuable applications of this amination protocol, we carried out the preparation of existing drugs as well as the introduction of –NH_2_ moieties into drugs and complex molecules. The amination of important drugs such as Nabumetone, Pentoxifylline, and Azaperone (Fig. 4; products **92–94**) as well as steroid derivatives has been demonstrated (Fig. 4; products **95–97**). Such an insertion of amino groups into life science molecules represents a resourceful technique for further functionalization and modulation of their activities, which is highly useful in drug discovery.

### Upscaling for the preparation of amines on gram-scale

In order to show practical utility and to demonstrate potential for implementation in industrial production, the upscaling of synthetic methodologies is very important. Especially in homogeneous catalysis upscaling is a challenging task. Therefore, to demonstrate the applicability of this homogeneous catalytic amination protocol, we performed gram-scale synthesis of six selected amines. As shown in (see Supplementary Figure 1), 2–10 g of four aldehydes and two ketones were successfully aminated to yield their corresponding primary amines in more or less similar yields to those of 50–100 mg scale reactions.

We were interested to compare our methodology to an established amination protocol. The Leuckart-Wallach reaction is a prime example, finding application also on an industrial scale^39–41^. We therefore subjected 15 aldehydes and ketones, which have been studied in this work, to Leuckart-Wallach reaction conditions^39–41^ to prepare the corresponding primary amines. As shown in Supplementary Table 1, the reaction worked well for simple aldehydes/ketones and gave 53–75% of corresponding primary amines (Supplementary Table 1; entries 1–3). For a majority of substituted and structurally diverse as well as heterocyclic aldehydes and ketones it gave poor yields (5–15%) (Supplementary Table 1; entries 4–9). The Leuckart-Wallach reaction failed to yield the desired primary amine for sensitive substrates (e.g., TMS or halogen containing) as well as some (hetero)cyclic and steroid derivatives (Supplementary Table 1; entries 10–15). A majority of the sensitive functional groups were not tolerated. Gratifyingly, for all these substrates, the RuCl_2_(PPh_3_)_3_ precatalyst using ammonia and hydrogen worked well and produced the corresponding primary amines in 72–93% yields. These results clearly reveal that catalytic reductive amination using RuCl_2_(PPh_3_)_3_ is more generally applicable for the preparation of primary amines compared to the traditional Leuckart-Wallach reaction.

## Discussion

**Fig. 5:**
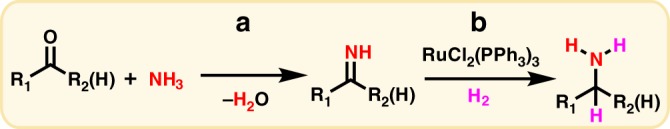
Ru-catalyzed reductive amination of carbonyl compounds with NH_3_ using H_2_. **a** Noncatalytic condensation reaction; **b** catalytic hydrogenation reaction

A general reaction pathway for the catalytic reductive amination of carbonyl compounds is shown in Fig. 5. Initially, the carbonyl compound undergoes condensation with ammonia to form the corresponding primary imine. Subsequently, the intermediate imine is hydrogenated to give the primary amine. The hydrogenation step is catalyzed by a catalytic species derived from the precatalyst, RuCl_2_(PPh_3_)_3_.

**Fig. 6:**
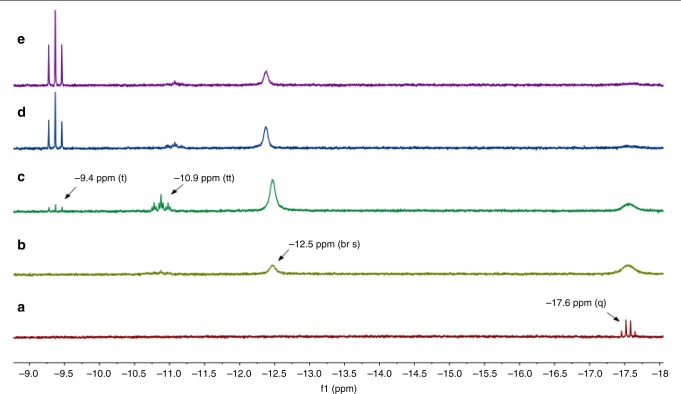
Hydride region of ^1^H NMR spectra of RuCl_2_(PPh_3_)_3_ in C_6_D_6_/methanol. **a** RT, argon atmosphere; **b** RT, H_2_ atmosphere (1.5 bar), 10 min; **c** RT, H_2_ atmosphere (1.5 bar), 2.5 h; **d** 60 °C, H_2_ atmosphere (1.5 bar), 30 min; **e** 60 °C, H_2_ atmosphere (1.5 bar), 1.5 h

We were interested to gain mechanistic insight into the hydrogenation step and to determine the nature of the active catalyst species. For this purpose, we studied the interaction of RuCl_2_(PPh_3_)_3_ with hydrogen using in situ NMR in a model system consisting solely of the ruthenium precatalyst, methanol and C_6_D_6_. Figure 6 depicts the hydride region of the obtained ^1^H NMR spectra. Initially, even in the absence of H_2_, a quartet at δ_H_ = −17.6 ppm is observed along with a broad singlet at δ_P_ = 55 ppm in the ^31^P{^1^H} NMR spectrum (see SI). We assign these signals to [RuHCl(PPh_3_)_3_]^63^, which is likely formed in small amounts via methanol oxidation. In presence of H_2_ (1.5 bar) at room temperature, the quartet corresponding to [RuHCl(PPh_3_)_3_] broadens^63^ and two new hydride signals appear: a broad singlet at δ_H_ = −12.5 ppm and a triplet of triplets at δ_H_ = −10.9 ppm. Using ^1^H-^31^P HMBC NMR (see Supplementary Figures 6–13) we were able to assign the hydride triplet of triplets to two multiplets in the ^31^P{^1^H} NMR spectrum (at δ_P_ = 34.8 ppm and δ_P_ = 58.4 ppm; see SI), which is consistent with the structure of [Ru(H)_2_(PPh_3_)_4_]^64^. We tentatively assign the broad singlet at δ_H_ = −12.5 ppm to [Ru(H)_2_(PPh_3_)_3_], which is corroborated by the appearance of a broad signal at δ_P_ = 58 ppm in the ^31^P{^1^H} NMR spectrum^65^. [Ru(H)_2_(PPh_3_)_3_] would be in equilibrium with [Ru(H)_2_(PPh_3_)_4_] via association/dissociation of a PPh_3_ ligand. After 2.5 h at room temperature a new triplet at δ_H_ = −9.4 ppm appears in the hydride region, which further increases in intensity upon heating to 60 °C. Using ^1^H-^31^P HMBC NMR we could assign this hydride signal to a singlet in the ^31^P{^1^H} NMR spectrum at δ_P_ = 50.4 ppm (see Supplementary Figures 6–13). After 1.5 h at 60 °C, it is the dominant species in the hydride region and in the ^31^P{^1^H} NMR spectrum. The triplet hydride splitting (37 Hz), which collapses to a singlet in the ^1^H{^31^P} NMR spectrum (see Supplementary Figures 6–13), indicates the presence of just two equivalent PPh_3_ ligands. When the ^31^P{^1^H} NMR experiment is decoupled with reduced power (only aromatic protons are decoupled) the singlet at δ_P_ = 50.4 ppm splits into a doublet (see Supplementary Figures 6–13)), indicating a monohydride structure. Although this species appears to have only two PPh_3_ and one hydride ligand, its accumulation indicates high stability under experimental conditions, suggesting the presence of other stabilizing ligands (such as CO). Since [Ru(H)_2_(PPh_3_)_3_] is known to decarbonylate methanol^66^ and due to similar spectral characteristics compared to [RuHCl(CO)(PPh_3_)_2_(pyrazine)]^67^ we tentatively assign this species to the carbonyl-containing complex [RuHCl(CO)(PPh_3_)_2_(Y)] (with Y possibly being a solvent molecule) formed via methanol decarbonylation.

**Fig. 7:**
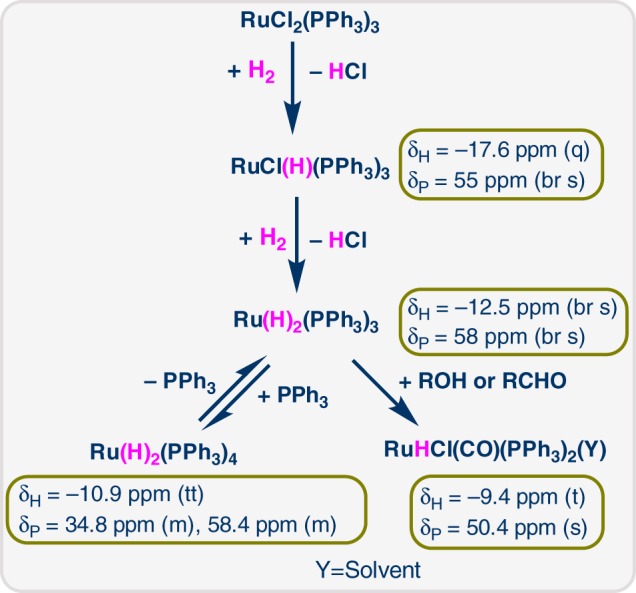
Generation of different species from RuCl_2_(PPh_3_)_3_ in presence of hydrogen. RuCl_2_(PPh_3_)_2_ = precatalyst; Ru(H)Cl(PPh_3_)_3_ and Ru(H)_2_(PPh_3_)_3_ = active catalytic species

An overview of the proposed transformation of RuCl_2_(PPh_3_)_3_ in our model system is provided in Fig. 7: RuCl_2_(PPh_3_)_3_ undergoes a stepwise reaction with H_2_ to form [RuHCl(PPh_3_)_3_] (which is also generated by the reaction with methanol) and Ru(H)_2_(PPh_3_)_3_, which is in equilibrium with Ru(H)_2_(PPh_3_)_4_. Ru(H)_2_(PPh_3_)_3_ can further react via alcohol decarbonylation to form the carbonyl-containing complex [RuHCl(CO)(PPh_3_)_2_(Y)]. While methanol is not present under our reaction conditions for reductive amination, it is known that RuCl_2_(PPh_3_)_3_ can also enable the decarbonylation of benzyl alcohols and aldehydes^65^, which constitute a majority of our substrates.

Also, a number of previously reported ruthenium systems for reductive amination as well as alcohol amination are based on carbonyl-containing (pre-)catalysts^26,27,35–37^. Therefore, the question arises whether our active catalyst contains a carbonyl ligand or if it conforms to a previously proposed [RuHX(PPh_3_)_3_] structure (X = H^−^ or Cl^−^)^68,69^. To answer this question, we have compared the catalytic performance of [RuHCl(PPh_3_)_3_] and [RuHCl(CO)(PPh_3_)_3_] for benzaldehyde amination under standard reaction conditions. Interestingly, [RuHCl(PPh_3_)_3_] performs similarly to RuCl_2_(PPh_3_)_3_ (88% benzylamine, 4% benzyl alcohol, 7% dibenzylamine; Supplementary Figure 2). This confirms that [RuHCl(PPh_3_)_3_] is part of the transformation cascade (as observed in our model system) which also includes the active catalyst. However, [RuHCl(CO)(PPh_3_)_3_] showed poor selectivity under our reaction conditions (3% benzylamine, 5% benzyl alcohol, 90% N-benzylidenebenzylamine; Supplementary Figure 3). In addition, a reported Ru_3_(CO)_12_/CataCxiumPCy catalytic system, which was used in the amination of alcohols with ammonia^36^, was also tested for the reductive amination of cyclohexanone (Supplementary Figure 3). Similarly, this catalyst also showed poor selectivity, yielding only 10% of cyclohexylamine. Therefore, carbonyl-containing complexes are likely not the active species under our reaction conditions. Rather, due to their decreased selectivity, they constitute a possible deactivation pathway for RuCl_2_(PPh_3_)_3_ catalyzed reductive amination. This difference between our observations and previously reported carbonyl-containing ruthenium amination catalysts is attributed to the ligand: carbonyl-containing Ru(II) catalysts typically require bidentate^26^ or tridentate^35^ ligands. Control experiments have shown significantly decreased yield in the absence of those additional ligands^26^. In contrast, the carbonyl-free catalyst type [RuHX(PPh_3_)_3_] appears to be sufficiently active and selective with only PPh_3_-derived ligands.

**Fig. 8:**
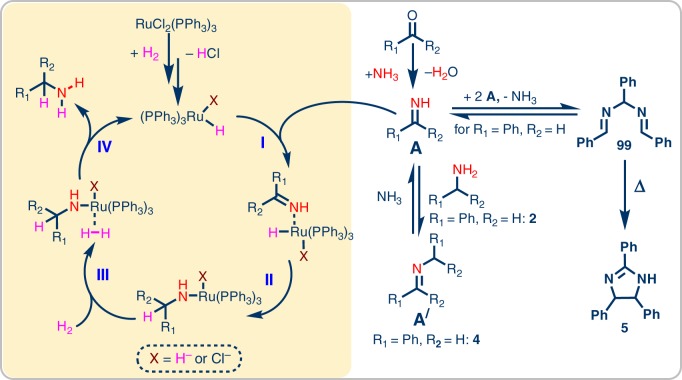
Proposed reaction mechanism for the RuCl_2_(PPh_3_)_3_-catalyzed reductive amination. **A** Unstable primary imine; **A**^/^ stable secondary imine. **5** **=** 2,4,5-triphenyl-2-imidazoline

After clarifying the pathway for catalyst activation we were interested to investigate the reaction cascade starting from the aldehyde/ketone and ammonia using the benzaldehyde benchmark system. The starting materials can undergo a condensation to form primary imine **A**. Intermediate **A**, however, was never detected in the reaction mixture, presumably due to its high reactivity. Instead (as can be seen in Fig. 1a), secondary imine **4** was determined to be the major intermediate. **4** is formed via condensation of the product **2** with either the starting aldehyde/ketone (releasing water) or via condensation of **2** and **A** (releasing NH_3_). We next reacted isolated **4** under our standard reaction conditions (40 bar H_2_, 5–7 bar NH_3_, 24 h, RuCl_2_(PPh_3_)_3_) and found almost quantitative conversion to **2** (Supplementary Figure 4). In contrast, when **4** was reacted in the absence of ammonia, 98% dibenzylamine was obtained after 24 h (Supplementary Figure 4). These results show that **4**, when exposed to NH_3_, is in an equilibrium with **A** **+** **2**^70^. While the catalyst is able to hydrogenate both **A** and **4**, **A** is hydrogenated preferentially and is replenished by the equilibrium with **4**. If ammonia is absent or only present in low concentrations (see Fig. 1e), the formation of **A** + **2** from **4** is suppressed, leading to hydrogenation of **4** (yielding dibenzlamine). Furthermore, due to rapid hydrogenation under optimized conditions, the stationary concentration of **A** is low, precluding side reactions of this reactive intermediate. When the hydrogenation does not proceed quickly, however, accumulation and side reactions involving **A** can likely occur. Correspondingly, when a mixture of **4** and benzaldehyde was reacted under standard conditions but without H_2_, 20% of the cyclic side-product **5** was obtained (the rest being unreacted starting material, see Supplementary Figure 5). Williams et al.^71^ and Corey et al.^72^ have reported that **A** can trimerize to form **99**, which can subsequently undergo thermal cyclization to form **5** (Fig. 8). This reaction of accumulated **A** likely explains the formation of large amounts of **5** in less active catalyst systems (see Table 1, entries 1–10; Fig. 1).

Based on these observations we propose the following mechanism (Fig. 8): Reaction of a carbonyl compound with NH_3_ yields primary imine **A**, which can be in an equilibrium with secondary imine **A**^**/**^ via condensation with the product amine. The precatalyst RuCl_2_(PPh_3_)_3_ is activated by H_2_ to form the the active catalyst species [RuHX(PPh_3_)_3_] (X being either H^−^ or Cl^−^). This active catalytic species selectively reacts with the primary imine to initially form a substrate complex (**I**). Substrate coordination is followed by hydride insertion (**II**), generating a Ru-amide complex. Coordination of H_2_ (**III**) followed by hydrogenolysis releases the primary amine as the final product with regeneration of the catalytic species (**IV**).

In conclusion, we demonstrated that using a simple RuCl_2_(PPh_3_)_3_ catalyst, the challenging reductive amination of carbonyl compounds using ammonia and molecular hydrogen for the selective synthesis of a variety of primary amines is possible. Applying this Ru-based reductive amination, starting from inexpensive aldehydes and ketones, functionalized and structurally diverse linear and branched primary amines have been synthesized under industrially viable and scalable conditions. In general, achieving a high degree of chemoselectivity in amination/hydrogenation reactions is a challenging task. In this regard our simple Ru-based methodology represents a unique example in homogeneous catalysis for the reductive amination of functionalized and challenging molecules. We have also shown the possibility of scaling this amination protocol up to 10 g without any loss in either activity or selectivity. The application of this approach is also extended to the synthesis and amination of various drug molecules and steroid derivatives. In situ NMR investigations provided clear hints on the formation of Ru-hydride species, which have been elucidated to be the active catalytic species in this RuCl_2_(PPh_3_)_3_-catalyzed reductive amination. With the help of these investigations, an appropriate reaction mechanism has been proposed.

## Methods

### General considerations

All carbonyl compounds (aldehydes and ketones), Ru-precursors and complexes and ligands, were obtained commercially. All catalytic experiments were carried out in 300, 100, and 500 mL autoclaves (PARR Instrument Company). In order to avoid unspecific reactions, all catalytic reactions were carried out either in glass vials, which were placed inside the autoclave, or glass/Teflon vessel fitted autoclaves. GC and GC-MS were recorded on a Agilent 6890N instrument. GC conversion and yields were determined by GC-FID, HP6890 with FID detector, column HP530 m × 250 mm × 0.25 μm. ^1^H, ^13^C NMR data were recorded on a Bruker AV 300 and Bruker AV 400 spectrometers using DMSO-d_6_, CD_3_OD or C_6_D_6_ as solvents. Ru(tris(4-methoxyphenyl)phosphine)_3_Cl_2_ and Ru(tris(4-chlorophenyl)phosphine)_3_Cl_2_ were prepared according to the reported procedure^73^.

### Reductive amination of carbonyl compounds with ammonia

The 8 mL dried glass vial was charged with a magnetic stirring bar and 0.5 mmol of corresponding carbonyl compound (aldehyde or ketone). Then 1.5 mL t-amyl alcohol as solvent and 2–3 mol% RuCl_2_(PPh_3_)_3_ catalysts (2 mol% in case of aldehydes and 3 mol% in case of ketones) were added. The glass vial was fitted with a septum, cap and needle, and placed into a 300 mL autoclave (eight vials with different substrates at a time). The autoclave was flushed with hydrogen twice at 40 bar pressure and then it was pressurized with 5–7 bar ammonia gas and 40 bar hydrogen. The autoclave was placed into an aluminum block preheated at 140 °C (placed inside 30 min before counting the reaction time in order to attain the reaction temperature) and the reactions were stirred for the required time. During the reaction the inside temperature of the autoclave was measured to be 130 °C and this temperature was used as the reaction temperature. After completion of the reactions, the autoclave was cooled to room temperature. The remaining ammonia and hydrogen were discharged and the vials containing reaction products were removed from the autoclave. The reaction products were analyzed by GC-MS and the corresponding primary amines were purified by column chromatography (silica; n-hexane-ethyl acetate mixture). The resulting amines were converted to their respective hydrochloride salt and characterized by NMR. For conversion into the hydrochloride salt, 1–2 mL methanolic HCl or dioxane HCl (1.5 M HCl in methanol or 4 N HCl in dioxane) was added to the ether solution of the respective amine and stirred at room temperature for 4–5 h. Then, the solvent was removed and the resulting hydrochloride salt of the amine was dried under high vacuum. For determining the yields by GC for selected amines, after completion of the reaction n-hexadecane (100 µL) as standard was added to the reaction vials and the reaction products were diluted with ethyl acetate followed by filtration using a plug of silica and then analyzed by GC.

### General procedure for the gram scale reactions

The Teflon or glass fitted 300 (5–10 g) or 500 mL (20 g) (in case 5–20 g) or 100 mL (in case of 2–2.5 g) autoclave was charged with a magnetic stirring bar and the corresponding carbonyl compound (2–20 g). Then 25–150 mL t-amyl alcohol was added. Subsequently, RuCl_2_(PPh_3_)_3_ (amount of catalysts equivalent to 2–3 mol%) was added. The autoclave was flushed with hydrogen twice at 40 bar pressure and then it was pressurized with 5–7 bar ammonia gas and 40 bar hydrogen. The autoclave was placed into an aluminum block preheated to 140 °C (placed 30 min before counting the reaction time in order to attain reaction temperature) and the reaction was stirred for the required time. During the reaction the inside temperature of the autoclave was measured to be 130 °C and this temperature was used as the reaction temperature. After completion of the reaction, the autoclave was cooled to room temperature. The remaining ammonia and hydrogen were discharged and the reaction products were removed from the autoclave. The reaction products were analyzed by GC-MS and the corresponding primary amines were purified by column chromatography (silica; n-hexane-ethyl acetate mixture). The resulting amines were converted to their respective hydrochloride salt and characterized by NMR.

### Procedure for the in situ NMR studies

The in situ observation of the Ru-hydrides was performed under hydrogen saturation conditions in a 5 mm glass NMR tube, equipped with a PTFE gas inlet hose and a circulation unit which produces a continuous gas flow through the solution^74,75^. The brown solution of the precursor complex RuCl_2_(PPh_3_)_3_ (50 mg) in 0.5 mL methanol/0.5 mL benzene-d6 was transferred to the NMR tube under Ar. After assembling the device under inert gas and characterizing the solution by its ^1^H and ^31^P NMR spectra, the system was filled with neat hydrogen (absolute pressure 1.5 bar). A gas flow of 1 mL min^–1^ was used to saturate the solution. ^1^H and ^31^P NMR spectra were taken at regular intervals to monitor the reaction progress. Changes were immediately observable as shown in Fig. 2. After three hours, the temperature was raised and kept at about 60 °C for another three hours to complete the reaction. No further changes were detected thereafter. The color of the solution was changed to brick-red at the end of the experiment. Note that maintaining a continuous gas flow till the very end was not possible, because black particles of precipitating metallic Ru were clogging the tubing.

## Electronic supplementary material

Supplementary Information

Peer Review File

## Data Availability

All data are available from the authors upon reasonable request.

## References

[1] Lawrence, S. A. *Amines: Synthesis, Properties and Applications*. (Cambridge University Press, Cambridge, UK, 2004).

[2] Ricci, A. *Amino Group Chemistry: From Synthesis to the Life Sciences*. (Wiley-VCH, Weinheim, 2008).

[3] Smith, D. T., Delost, M. D., Qureshi, H. & Njarðarson, J. T. Top 200 Pharmaceutical Products by Retail Sales in 2016. https://njardarson.lab.arizona.edu/sites/njardarson.lab.arizona.edu/files/2016Top200PharmaceuticalRetailSalesPosterLowResV3_0.pdf (2017).

[4] Roughley SD, Jordan AM (2011). The medicinal chemist’s toolbox: an analysis of reactions used in the pursuit of drug candidates. J. Med. Chem..

[5] Froidevaux V, Negrell C, Caillol S, Pascault JP, Boutevin B (2016). Biobased amines: from synthesis to polymers; present and future. Chem. Rev..

[6] Mao R, Frey A, Balon J, Hu X, Decarboxylative C (2018). (sp^3^)–N cross-coupling via synergetic photoredox and copper catalysis. Nat. Catal..

[7] Gomez SA, Peters JA, Maschmeyer T (2002). The reductive amination of aldehydes and ketones and the hydrogenation of nitriles: mechanistic aspects and selectivity control. Adv. Synth. Catal..

[8] Alinezhad H, Yavari H, Salehian F (2015). Recent advances in reductive amination catalysis and its applications. Curr. Org. Chem..

[9] Nugenta TC, El-Shazlya M (2010). Chiral amine synthesis-recent developments and trends for enamide reduction, reductive amination, and imine reduction. Adv. Synth. Catal..

[10] Wakchaure VN, Zhou J, Hoffmann S, List B (2010). Catalytic asymmetric reductive amination of α‐branched ketones. Angew. Chem. Int. Ed..

[11] Chusov D, B. List B (2014). Reductive amination without an external hydrogen source. Angew. Chem. Int. Ed..

[12] Natte K, H. Neumann H, Jagadeesh RV, Beller M (2017). Convenient iron-catalyzed reductive aminations without hydrogen for selective synthesis of N-methylamines. Nat. Commun..

[13] Jagadeesh RV (2015). Hydrogenation using iron oxide–based nanocatalysts for the synthesis of amines. Nat. Protoc..

[14] Reductive amination. https://www.reagentguides.com/reagent-guides/reductive-amination (2015).

[15] Gusak KN, Ignatovich ZV, Koroleva EV (2015). New potential of the reductive alkylation of amines. Russ. Chem. Rev..

[16] Senthamarai T (2018). Expedient synthesis of N‐methyl‐ and N‐alkylamines by reductive amination using reusable cobalt oxide nanoparticles. ChemCatChem.

[17] Jagadeesh RV (2017). MOF-derived cobalt nanoparticles catalyze a general synthesis of amines. Science.

[18] Komanoya T, Kinemura T, Kita Y, Kamata YK, Hara M (2017). Electronic effect of ruthenium nanoparticles on efficient reductive amination of carbonyl compounds. J. Am. Chem. Soc..

[19] Nakamura Y, Kon K, Touchy AS, Shimizu Ki, Ueda W (2015). Selective synthesis of primary amines by reductive amination of ketones with ammonia over supported Pt catalysts. ChemCatChem.

[20] Liang G (2017). Production of primary amines by reductive amination of biomass-derived aldehydes/ketones. Angew. Chem. Int. Ed..

[21] Wang, Z. Mignonac reaction. In *Comprehensive organic name reactions and reagents*. (John Wiley & Sons, New Jersey, 2010).

[22] Chatterjee M, Takayuki Ishizakaa T, Kawanami H (2016). Reductive amination of furfural to furfurylamine using aqueous ammonia solution and molecular hydrogen: an environmentally friendly approach. Green. Chem..

[23] Reductive amination review. https://erowid.org/archive/rhodium/chemistry/reductive.amination.html (2004).

[24] Gross T, Seayad AM, Ahmad M, Beller M (2002). Synthesis of primary amines: first homogeneously catalyzed reductive amination with ammonia. Org. Lett..

[25] Riermeier T (2005). Method for producing amines by homogeneously catalyzed reductive amination of carbonyl compounds. US.

[26] Gallardo-Donaire J, Ernst M, Trapp O, Schaub T (2016). Direct synthesis of primary amines via ruthenium‐catalysed amination of ketones with ammonia and hydrogen. Adv. Synth. Catal..

[27] Gallardo-Donaire J (2018). Direct asymmetric ruthenium-catalyzed reductive amination of alkyl-aryl ketones with ammonia and hydrogen. J. Am. Chm. Soc..

[28] Ogo S, Uehara K, Abura T, Fukuzumi S (2004). pH-Dependent chemoselective synthesis of α-amino acids. Reductive amination of α-keto acids with ammonia catalyzed by acid-stable iridium hydride complexes in water. J. Am. Chm. Soc..

[29] Kadyrov R, Riermeier TH (2003). Highly enantioselective hydrogen-transfer reductive amination: catalytic asymmetric synthesis of primary amines. Angew. Chem. Int. Ed..

[30] Tan X (2018). Asymmetric synthesis of chiral primary amines by ruthenium-catalyzed direct reductive amination of alkyl aryl ketones with ammonium salts and molecular H_2_. J. Am. Chem. Soc..

[31] Yan T, Feringa BL, Barta K (2014). Iron catalysed direct alkylation of amines with alcohols. Nat. Commun..

[32] Meindl WR, Angerer EV, Schoenenberger H, Ruckdeschel G (1984). Benzylamines: synthesis and evaluation of antimycobacterial properties. J. Med. Chem..

[33] Yan T, Feringa BL, Barta K (2017). Direct N-alkylation of unprotected amino acids with alcohols. Sci. Adv..

[34] Goldacre RJ (1944). Mode of action of benzylamine sulphonamide (‘Marfanil’). Nature.

[35] Gunanathan C, Milstein D (2008). Selective synthesis of primary amines directly from alcohols and ammonia. Angew. Chem. Int. Ed..

[36] Imm S, Bähn S, Neubert L, Neumann H, Beller M (2010). An efficient and general synthesis of primary amines by ruthenium-catalyzed amination of secondary alcohols with ammonia. Angew. Chem Int. Ed..

[37] Pingen D, Müller C, Vogt D (2010). Direct amination of secondary alcohols using ammonia. Angew. Chem. Int. Ed..

[38] Bähn S (2011). M. The catalytic amination of alcohols. ChemCatChem.

[39] Leuckart R (1885). Ueber eine neue bildungsweise von tribenzylamin. Ber.

[40] Moore, M. L. *Organic Reactions* (Wiley, Hoboken, NJ, 2011).

[41] Crossley FS, Moore ML (1944). Studies on Leuckart reaction. J. Org. Chem..

[42] Feuer H, Braunstein DM (1969). Reduction of oximes, oxime ethers, and oxime esters with diborane. Nov. Synth. Amines J. Org. Chem..

[43] Huang X (2007). Asymmetric synthesis of primary amines via the spiroborate-catalyzed borane reduction of oxime ethers. Org. Lett..

[44] Mirjafary Z, Abdoli M, Saeidian H, Boroon S, Kakanejadifard A (2015). Oxime ethers as versatile precursors in organic synthesis: a review. RSC Adv..

[45] Ammonia. https://pubchem.ncbi.nlm.nih.gov/compound/ammonia (2016).

[46] Appl, M. in *Ullmann’s Encyclopedia of Industrial Chemistry*, 7th edn. (ed. Wiley-VCH) (Wiley, New York, 2011).

[47] van Gysel, A. B. & Musin, W. in *Ullmann’s Encyclopedia of Industrial Chemistr*y, 7th edn. (ed. Wiley-VCH) (Wiley, New York, 2011).

[48] Schirmann, P. & Bourdauducq, J.-P. in *Ullmann’s Encyclopedia of Industrial Chemistry*, 7th edn. (ed. Wiley-VCH) (Wiley, New York, 2011).

[49] Klinkenberg JL, Hartwig JF (2011). Catalytic organometallic reactions of ammonia. Angew. Chem. Int. Ed..

[50] Schranck J, Tlili A (2018). Transition-metal-catalyzed monoarylation of ammonia. ACS Catal..

[51] Cobb, J. E. et al. In *Encyclopedia of Reagents for Organic Synthesis* (eds. Paquette, L. A. et al.) (John Wiley & Sons, New York, 2004).

[52] Burke, S. D. & Danheiser, R. L. *Triphenylphosphine, Handbook of Reagents for Organic synthesis, Oxidizing and Reducing Agents* (Wiley, Hoboken, NJ, 1999).

[53] Pignolet, L. M. *Homogeneous Catalysis with Metal Phosphine Complexes* (Springer US, 2013).

[54] Wilkinson’s catalyst, *Comprehensive Organic Name Reactions and Reagents* (2010).

[55] Müller, C. & Vogt, D. Phosphinines as ligands in homogeneous catalysis: Recent developments, concepts and perspectives. *Dalton. Trans*. 5505–5523 (2007). 10.1039/b712456m 18043811

[56] Plummer, J. S., Shun-Ichi, M. & Changjia, Z. Dichlorotris(triphenylphosphine)ruthenium(II), *e-EROS Encyclopedia of Reagents for Organic Synthesis* (John Wiley, 2010).

[57] Crabtree RH (2017). Homogeneous transition metal catalysis of acceptorless dehydrogenative alcohol oxidation: applications in hydrogen storage and to heterocycle synthesis. Chem. Rev..

[58] Guillena G, Ramon DJ, Yus M (2010). Hydrogen autotransfer in the N-alkylation of amines and related compounds using alcohols and amines as electrophiles. Chem. Rev..

[59] Sameca JSM, Bäckvall JE, Andersson PG, Brandt P (2006). Mechanistic aspects of transition metal-catalyzed hydrogen transfer reactions. Chem. Soc. Rev..

[60] Pingen D, Lutz M, Vogt D (2014). Mechanistic study on the ruthenium-catalyzed direct amination of alcohols. Organometallics.

[61] Evans D, Osborn JA, Jardine FH, Wilkinson G (1965). Homogeneous hydrogenation and hydroformylation using ruthenium complexes. Nature.

[62] Wang, G.-Z. & Bäckvall, J. E. Ruthenium-catalysed transfer hydrogenation of imines by propan-2-ol. *Chem. Commun*. 980–982 (1992). 10.1002/1521-3765(20020703)8:13<2955::AID-CHEM2955>3.0.CO;2-Q 12489225

[63] Kirss RU, Eisenschmid TC, Eisenberg R (1988). Para hydrogen induced polarization in hydrogenation reactions catalyzed by ruthenium phosphine complexes. J. Am. Chem. Soc..

[64] Samouei H, Miloserdov FM, Escudero-Adán EC, Grushin VV (2014). Solid-state structure and solution reactivity of [(Ph3P)4Ru(H)2] and related Ru(II) complexes used in catalysis: a reinvestigation. Organometallics.

[65] Mazziotta A, Madsen R (2017). Ruthenium‐catalyzed dehydrogenative decarbonylation of primary alcohols. Eur. J. Org. Chem..

[66] Van der Sluys LS, Kubas GJ, Caulton KG (1991). Reactivity of (dihydrogen)dihydridotris(triphenylphosphine)ruthenium. Dimerization to form (PPh_3_)_2_(H)Ru(.mu.-H)_3_Ru(PPh_3_)_3_ and decarbonylation of ethanol under mild conditions. Organometallics.

[67] MaŁecki JG, Kruszynski R (2007). Synthesis, crystal and spectroscopic characterization of [RuHCl(CO)(PPh3)2(pyrazine)]. J. Coord. Chem..

[68] Aranyos, A., Csjernyik, G., Szabo, K. S. & Bäckvall, J. E. Evidence for a ruthenium dihydride species as the active catalyst in the RuCl_2_(PPh_3_)-catalyzed hydrogen transfer reaction in the presence of base. *Chem. Commun*. 351–352 (1999).

[69] Masters, C. Homogeneous Transition-Metal Catalysis: A Gentle Art. 51–55 (Chapman & Hall, 1981).

[70] Samouei H, Vladimir V, Grushin VV (2013). New, highly efficient, simple, safe, and scalable synthesis of [(Ph_3_P)_3_Ru(CO)(H)_2_]. Organometallics.

[71] Williams OF, Bailar JC (1959). The stereochemistry of complex inorganic compounds. XXIV. Cobalt stibenediamine complexes. J. Am. Chem. Soc..

[72] Corey EJ, Kühnle FNM (1997). A simplified synthesis of (±)−1,2-diphenyl-1,2-diaminoethane (1) from benzaldehyde and ammonia. Revision of the structures of the long-known intermediates “hydrobenzamide” and “amarine. Tetrahedron Lett..

[73] Samouei, H., Vladimir, V. & Grushin, V. V. New, highly efficient, simple, safe, and scalable synthesis of [(Ph_3_P)_3_Ru(CO)(H)_2_]. *Organometallics* **32**, 4440–4443 (2013).

[74] Baumann W, Mansel S, Heller D, Borns S (1997). Gas bubbles in the NMR tube: an easy way to investigate reactions with gases in the liquid phase. Magn. Reson. Chem..

[75] Baumann, W., Börner, A., Selent, D. Gas injection and circulation device for tracking of reactions in the liquid phase involving gaseous reactants under normal and high pressure by means of nuclear magnetic resonance spectroscopy (NMR spectroscopy pressure) under steady state conditions. *DE10333143B4*, 2008.

