# Supplementary material for: Simple ruthenium-catalyzed reductive amination enables the synthesis of a broad range of primary amines
Source: Nat Commun. 2018 Oct 8;9:4123. doi: 10.1038/s41467-018-06416-6 (PMC6175844; doi:10.1038/s41467-018-06416-6)
Supplement: Supplementary file 1 — Supplementary Information [file 41467_2018_6416_MOESM1_ESM.pdf]

Supporting Information

Simple ruthenium-catalyzed reductive amination enables the synthesis of a broad range of primary amines

Senthamarai, et al

Supplementary methods

Detailed procedure the preparation of primary amines by Leuckart-Wallach reaction

To a 250ml four-necked flask, equipped with a dropping-funnel, thermometer, water segregator and down-directed condenser, 5 equivalents of ammonium formate were added and stirred at 120°C to melt the solid ammonium formate. After complete melting of ammonium formate, 1-5 mmol of substrate (carbonyl compound) was added and allowed to react for 24 h at 140 °C. Then, the flask was cooled down to the room temperature and the obtained formamide of the corresponding amine was hydrolyzed with 5 equivalents of concentrated aqueous hydrochloric acid (36-38%) by refluxing at 110 °C for 9 h. After cooling to the room temperature, the reaction mixture was diluted with 15 ml of water and filtered. Then the filtrate was extracted with 30ml ether to remove water-insoluble material. The filtrate containing the corresponding amine in HCl salt form was neutralized with aqueous NaOH (15%) and extracted thoroughly with ether (5 x 50 mL). The ether factions were combined and were evaporated to obtain the corresponding primary amine.

Supplementary note

Concentration of 5 in Fig. 1a (manuscript)

In the concentration-time graph (Fig. 1a), **5** appears to be formed at the beginning of the reaction and then seems to be consumed during further reaction progress. **5**, however, cannot be converted to any other product under our reaction conditions (*vide supra*). We therefore sought for an explanation of the apparent formation and decrease of **5**. As can be seen in Fig. 1d, the hydrogenation activity of the catalyst system drops rapidly for temperatures > 130 °C. Therefore, we expect that while the reaction is cooled down, the hydrogenation reactions quickly ceases, leading to a higher concentration of primary imine. While the thermal energy is still sufficient, the primary imine can then trimerize (which proceeds even at room temperature, see Ref. 78 manuscript) and cyclize (which requires elevated temperature) to form **5**. While this side reaction during cool down would not affect the optimized reaction (since there is no primary imine left after complete hydrogenation), it could occur for incomplete reactions (e.g. when the reaction is stopped prematurely). In line with this reasoning, we found that when the optimized benzaldehyde amination is stopped after just 30 min, 30% yield for **5** is obtained (see Fig. S4). Therefore, we propose that formation and apparent decrease of **5** in the concentration-time profile is an artifact resulting from prematurely quenching the reaction.

Supplementary Tables

Supplementary Table 1. Preparation of primary amines from aldehydes and ketones by Leuckart-Wallach reaction.

Entry	Aldehyde /Ketone	Conv.	Yield of formamide (GC/GCMS yield)	Yield of primary amine (isolated yield)
1	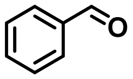	>99	85%<br>(10% diamino benzyl formamide)<br>(3% tri benzylamine)	75%
2	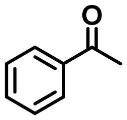	75%	60% formamide<br>14% diamine	53%<br>(14% diamine)
3	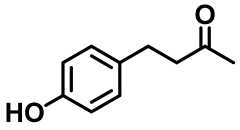	80%	75%	70%
4	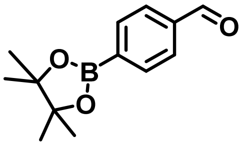	>99%	90%<br>(5-8% of corresponding acid)	5%<br>(84% of benzyl amine. Boronic acid ester group was cleaved)
5	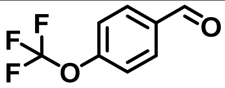	>99%	20%<br>(78% of other products were observed)	15%
6	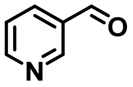	>99%	25%<br>(20% pyridyl carboxylic acid. 53% other products)	15%
7	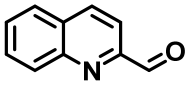	>99%	10%<br>(88% quinoline carboxylic acid	5%
8	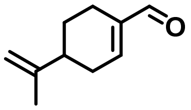	>99%	30%<br>(20% one C-C double bond cleaved formamide product. 48% of other products)	10%

9	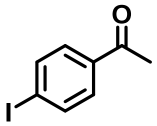	30%	27%	5%<br>(20% of de-iodo product)
10	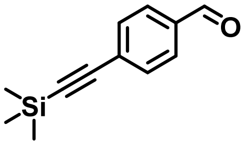	>99%	0%<br>(90% N-Benzyl-formamide.<br>9% benzoic acid. trimethylsilyl group was completely cleaved)	0%<br>(88% benzylamine)
11	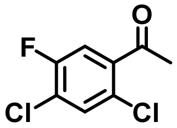	<2%	<1%	<1%
12	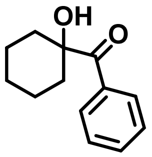	>99	<2%<br>(95% N-Benzyl-formamide. A cleaved product)	<1%<br>(90% benzylamine)
13	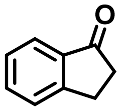	<2%	>1%	-
14	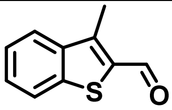	>99%	0%<br>(formamide was not observed. Other products were observed)	0%
15	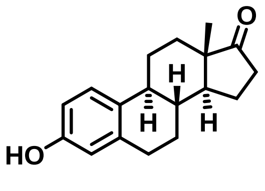	<1%	<1%	<1%

Reaction conditions: 1-5 mmol of substrate, 5 equivalents of ammonium formate, 140 ^{o}C, 24 h. After the formation of corresponding formamide, 5 equivalents of aq. HCl (concentrated 37%) was added and refluxed at 110 ^{o}C for 9h and filtered, washed with diethyl ether and then the filtrate was neutralized with NaOH. Finally, the product was extracted thoroughly with ethyl acetate to obtain the corresponding free primary amine.

Supplementary Table 2. Reductive amination of selected aldehydes and ketones using different $RuCl_2(PPh_3)_3$ loading

Entry	Aldehyde/ketone	$RuCl_2(PPh_3)_3$ loading (mol%)	Conversion	Product (s) (GC yield(s))
1	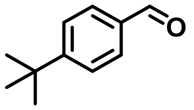	0.1	80%	0% Primary amine<br>77% Secondary imine
2	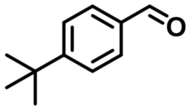	0.5	80%	19% Primary amine<br>60% Secondary imine
3	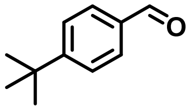	1	99%	70% Primary amine<br>25% Secondary imine<br>4% Side product **5**
4	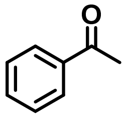	0.1	10%	9% secondary imine
5	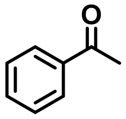	0.5	80%	60% Primary amine<br>15% Alcohol<br>20% Secondary imine
6	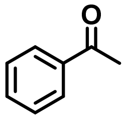	1	99%	95% Primary amine<br>5% Alcohol
7	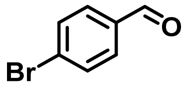	1	99%	99% Secondary imine
8	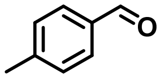	1	99%	99% Secondary imine
9	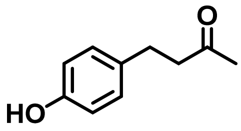	1	30%	30% Imine

10	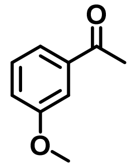	1	30%	25% Primary amine
11	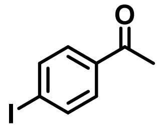	1	30%	20% Primary amine<br>5% Alcohol
12	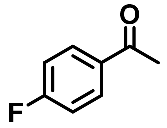	1	80%	50% Primary amine<br>20% Alcohol<br>10% Secondary imine
13	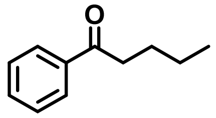	1	75%	25% Primary amine<br>50% Alcohol
14	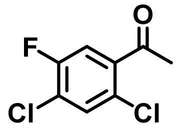	1	40%	20% Primary amine<br>20% Alcohol
15	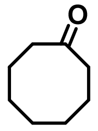	1	70	65% Secondary imine
16	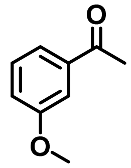	2	90%	25% Primary amine<br>65% Alcohol
17	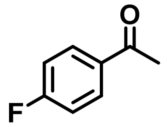	2	88%	40% Primary amine<br>48% Alcohol
18	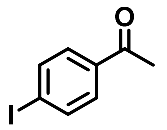	2	85%	70% Primary amine<br>15% Alcohol

Reaction conditions: 0.5 mmol benzaldehyde/ketone, 0.1-2 mol% $RuCl_2(PPh_3)_3$, 5-7 bar NH_3, 40 bar H_2 1.5 mL t-amyl alcohol, 130 ^oC, 24 h

NMR data of amines

TM5-252

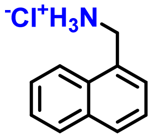

^{1}H NMR (300 MHz, DMSO-d_6): δ 8.64 (br s, 3H), 8.20 – 8.12 (m, 1H), 8.04 – 7.95 (m, 2H), 7.71 – 7.53 (m, 4H), 4.52 (s, 2H).
^{13}C NMR (75 MHz, DMSO): δ 133.67, 131.12, 130.45, 129.48, 129.10, 127.74, 127.21, 126.69, 125.82, 123.93, 40.81. Brown solid.

TM5-325

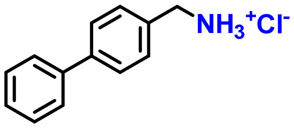

^{1}H NMR (400 MHz, DMSO-d_6): δ 8.70 (br s, 3H), 7.74 – 7.59 (m, 6H), 7.52 – 7.32 (m, 3H), 4.05 (s, 2H).
^{13}C NMR (101 MHz, DMSO): δ 140.61, 139.99, 133.76, 130.08, 129.45, 128.13, 127.20, 127.15, 42.24. White solid.

TM5-210

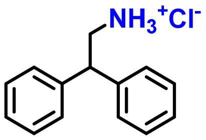

^{1}H NMR (300 MHz, DMSO-d_6): δ 8.24 (br s, 3H), 7.42 – 7.19 (m, 10H), 4.44 (t, J = 7.8 Hz, 1H), 3.57 (d, J = 6.3 Hz, 2H).
^{13}C NMR (75 MHz, DMSO): δ 141.53, 129.24, 128.29, 127.47, 48.90, 42.90. Off-White solid.

TM5-244

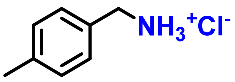

^{1}H NMR (300 MHz, DMSO-d_6): δ 8.62 (br s, 3H), 7.42 (d, J = 8.0 Hz, 2H), 7.18 (d, J = 7.8 Hz, 2H)), 3.94 (s, 2H), 2.29 (s, 3H).
^{13}C NMR (75 MHz, DMSO): δ 138.08, 131.51, 129.46, 129.46, 42.34, 21.24. White solid.

TM5-200

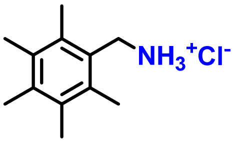

^{1}H NMR (400 MHz, DMSO-d_6): δ 8.31 (br s, 3H), 4.05 (s, 2H), 2.23 (s, 15H).
^{13}C NMR (101 MHz, DMSO): δ 135.75, 133.51, 132.70, 128.46, 37.73, 16.97. White solid.

TM5-331

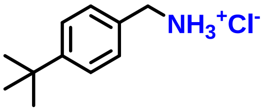

^{1}H NMR (400 MHz, DMSO-d_6): δ 8.68 (br s, 3H), 7.28 (d, J = 7.8 Hz, 2H), 7.11 (d, J = 7.8 Hz, 2H),3.94 (s, 2H), 1.25 (s, 9H).
^{13}C NMR (101 MHz, DMSO): δ 151.24, 131.58, 129.29, 125.67, 42.21, 34.76, 31.53. pale Brown solid.

TM5-220

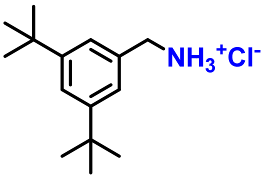

^{1}H NMR (300 MHz, DMSO-d_6): δ 8.61 (br s, 3H), 7.38 (s, 2H), 7.31 (s,1H), 3.95 (s, 2H), 1.24 (s, 18H).
^{13}C NMR (75 MHz, DMSO): δ 150.96, 133.50, 123.79, 122.08, 43.16, 35.01, 31.67. White solid.

TM5-226

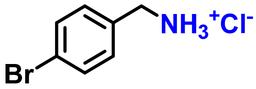

^{1}H NMR (400 MHz, DMSO-d_6): δ 8.61 (br s, 3H), 7.63 (d, J = 8.3 Hz, 2H), 7.51 (d, J = 8.5 Hz, 2H), 4.12 (s, 2H).
^{13}C NMR (101 MHz, DMSO): δ 133.94, 131.85, 131.77, 122.15, 41.90. Brown Solid.

TM5-328

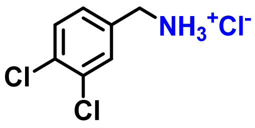

^{1}H NMR (300 MHz, DMSO-d_6): δ 8.72 (br s, 3H), 7.87 (d, J = 2.0 Hz, 1H), 7.67 (d, J = 8.3 Hz, 1H), 7.54 (dd, J = 8.3, 2.1 Hz, 1H), 4.04 (s, 2H).
13C NMR (75 MHz, DMSO): δ 135.62, 131.70, 131.50, 131.42, 131.07, 130.01, 41.36. White solid.

TM5-330

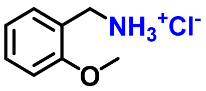

^{1}H NMR (300 MHz, DMSO-d_6): δ 8.52 (br s, 3H), 7.51 – 7.29 (m, 2H), 7.13 – 6.59 (m, 2H), 3.94 (s, 2H), 3.82 (s, 3H).
^{13}C NMR (75 MHz, DMSO): δ 157.58, 130.71, 130.56, 122.20, 120.69, 111.34, 56.04, 37.83. Off-White solid.

TM5-245

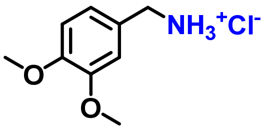

^{1}H NMR (300 MHz, DMSO-d_6): δ 8.63 (br s, 3H), 7.30 (s, 1H), 7.13 (d, J = 8.3 Hz, 2H), 3.92 (s, 2H), 3.76 (s, 6H).
^{13}C NMR (75 MHz, DMSO): δ 149.26, 149.04, 126.79, 121.93, 113.52, 112.00, 56.09, 56.06, 42.50. White solid.

TM5-375

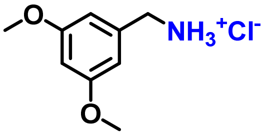

^{1}H NMR (300 MHz, DMSO-d_6): δ 8.83 (br s, 3H), 6.97 (s, 3H), 3.96 (s, 2H), 3.83 (s, 6H).
^{13}C NMR (75 MHz, DMSO): δ 160.91, 136.64, 107.36, 100.40, 56.07, 55.80, 42.67. Off-White solid.

TM5-231

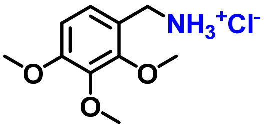

^{1}H NMR (300 MHz, DMSO-d_6): δ 8.56 (br s, 3H), 7.24 (d, J = 8.5 Hz, 1H), 6.83 (d, J = 8.5 Hz, 1H), 3.89 (s, 2H), 3.86 (s, 3H), 3.74 (s, 6H).
^{13}C NMR (75 MHz, DMSO): δ 154.32, 151.78, 141.77, 125.21, 119.87, 108.14, 61.51, 60.86, 56.46, 37.13. Off-White solid.

TM5-243

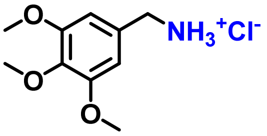

^{1}H NMR (300 MHz, DMSO-d_6): δ 8.71 (br s, 3H), 6.98 (s, 2H), 4.02 (s, 2H), 3.78 (s, 6H), 3.64 (s, 3H).
^{13}C NMR (75 MHz, DMSO): δ 153.22, 137.73, 130.06, 107.10, 60.48, 56.52, 42.86. Off-White solid.

TM5-222

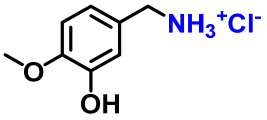

1H NMR (300 MHz, DMSO-d6): δ 10.12 (br s, 1H), 8.32 (br s, 3H), 7.25 – 6.73 (m, 3H), 3.84 (s, 2H), 3.76 (s, 3H).
13C NMR (75 MHz, DMSO): δ 148.32, 146.87, 126.75, 120.46, 116.78, 112.53, 56.16, 42.40. White solid.

TM5-246

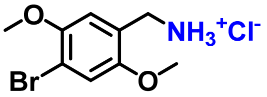

^{1}H NMR (400 MHz, DMSO-d_6): δ 8.63 (br s, 3H), 7.54 (s, 2H), 3.95 (s, 2H), 3.68 (s, 6H).
^{13}C NMR (101 MHz, DMSO): δ 151.71, 149.67, 122.51, 116.31, 115.58, 111.18, 57.29, 56.87, 37.33. Off-White solid.

TM5-324

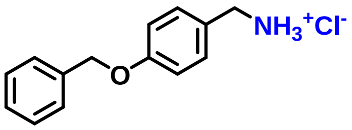

^{1}H NMR (400 MHz, DMSO-d_6): δ 8.53 (br s, 3H), 7.65 – 7.18 (m, 7H), 7.10 – 6.87 (m, 2H), 5.13 (s, 2H), 4.11 (s, 2H).
^{13}C NMR (101 MHz, DMSO): δ 158.76, 137.41, 131.04, 128.90, 128.29, 128.08, 126.68, 115.25, 69.61, 42.06. Off-White solid.

TM5-190

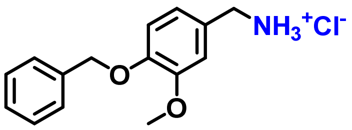

^{1}H NMR (300 MHz, DMSO-d_6): δ 8.50 (br s, 3H), 7.49 – 7.17 (m, 6H), 7.10 – 6.77 (d, J = 8.5 Hz, 2H), 5.10 (s, 2H), 3.99 (s, 2H), 3.80 (s, 3H).
^{13}C NMR (75 MHz, DMSO): δ 149.48, 148.19, 137.48, 128.88, 128.31, 128.15, 127.12, 121.85, 113.87, 113.73, 70.31, 56.17, 42.56. Off-White solid.

TM5-335

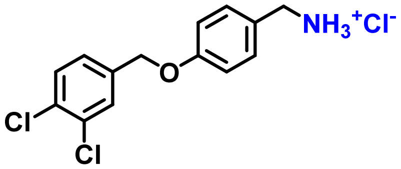

^{1}H NMR (400 MHz, DMSO-d_6): δ 8.55 (br s, 3H), 7.71 (d, J = 1.9 Hz, 1H), 7.67 – 7.62 (m, 1H), 7.49 – 7.41 (m, 3H), 7.06 – 7.01 (m, 2H), 5.15 (s, 2H), 3.92 (s, 2H).
13C NMR (101 MHz, DMSO): δ 158.34, 138.70, 131.55, 131.14, 131.10, 130.79, 129.83, 128.20, 127.02, 115.26, 68.03, 40.37. Off-white solid.

TM5-357

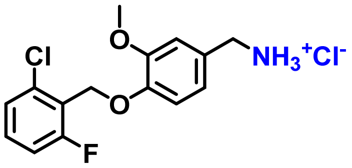

^{1}H NMR (300 MHz, DMSO-d_6): δ 8.43 (br s, 3H), 7.51 (td, J = 8.2, 6.1 Hz, 1H), 7.41 (dt, J = 8.1, 1.0 Hz, 1H), 7.36 – 7.21 (m, 2H), 7.14 (d, J = 8.2 Hz, 1H), 7.02 (dd, J = 8.2, 2.0 Hz, 1H), 5.12 (s, 2H), 3.95 (s, 2H), 3.74 (s, 3H).
13C NMR (75 MHz, DMSO-d6): δ 161.93 (d, J = 249.9 Hz), 149.51 , 148.11 , 135.99 (d, J = 5.2 Hz), 132.31 (d, J = 10.0 Hz), 127.80 , 126.18 (d, J = 3.2 Hz), 122.46 (d, J = 17.9 Hz), 121.86 , 115.22 (d, J = 22.4 Hz), 114.12 , 113.69 , 62.11 (d, J = 3.8 Hz), 56.05 , 42.52 . Off-White solid.

TM5-329

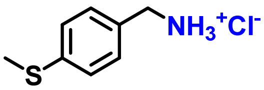

^{1}H NMR (400 MHz, DMSO-d_6): δ 8.68 (br s, 3H), 7.50 (d, J=7.9 Hz, 2H), 7.31 (d, J=8.3 Hz, 2H), 3.94 (s, 2H), 2.46 (s, 3H).
^{13}C NMR (101 MHz, DMSO): δ 138.95, 130.94, 130.18, 126.19, 42.08, 15.11. Brown solid.

TM5-227

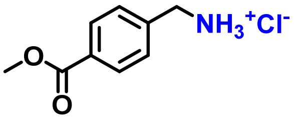

^{1}H NMR (300 MHz, DMSO-d_6): δ 8.75 (br s, 3H), 7.99 (d, J=8.7 Hz, 2H), 7.51 (d, J=8.8 Hz, 2H), 4.10 (s, 2H), 3.86 (s, 3H).
^{13}C NMR (75 MHz, DMSO): δ 166.37, 139.91, 129.91, 129.74, 129.69, 52.71, 42.16. Off-White solid.

TM5-194

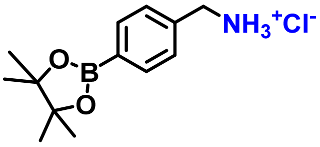

^{1}H NMR (300 MHz, DMSO-d_6): δ 8.61 (br s, 3H), 7.48 (d, J = 8.4 Hz, 2H), 7.32 (d, J = 9.8 Hz, 2H).4.02 (s, 2H), 1.29 (s, 12H).
^{13}C NMR (75 MHz, DMSO): δ 137.82, 134.95,134.73, 128.79, 84.21, 42.52, 25.13. Off-White solid.

TM5-230

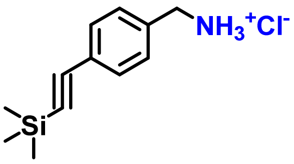

^{1}H NMR (300 MHz, DMSO-d_6): δ 8.23 (br s, 3H), 7.26-7.16 (m, 4H), 3.80 (s, 2H), 0.00 (s, 9H).
^{13}C NMR (75 MHz, DMSO): δ 134.44, 131.35, 128.83, 121.81, 104.28, 65.98, 41.47, -0.50. Off-White solid.

TM5-336

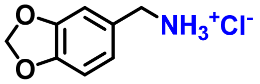

^{1}H NMR (300 MHz, DMSO-*d*$_6$): δ 8.56 (br s, 3H), 7.26 – 6.70 (m, 3H), 6.01 (s, 2H), 3.91 (s,2H).
^{13}C NMR (75 MHz, DMSO): δ 147.70, 147.66, 128.00, 123.41, 109.99, 108.65, 101.65, 42.46.
Off-White solid.

M5-183

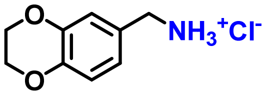

1H NMR (300 MHz, DMSO-d6): δ 8.36 (br s, 3H), 7.03 (d, J = 2.0 Hz, 1H), 6.93 (dd, *J* = 8.3, 2.1 Hz, 1H), 6.83 (d, *J* = 8.2 Hz, 1H), 4.23 (s, 2H), 3.56 (s, 4H).
13C NMR (75 MHz, DMSO): δ 143.98, 143.62, 127.30, 122.52, 118.34, 117.53, 64.53, 42.15.
White solid.

TM5-353

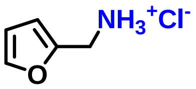

^{1}H NMR (300 MHz, DMSO-*d*$_6$): δ 8.74 (br s, 3H), 7.69 (d, *J* = 1.9 Hz, 1H), 6.70 (d, J = 5.6 Hz, 2H), 4.02 (s, 2H).
^{13}C NMR (75 MHz, DMSO): δ 147.88, 143.96, 111.38, 110.86, 35.41. Off-White solid.

M5-196

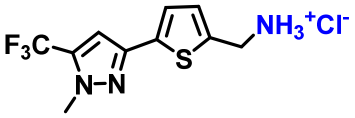

^{1}H NMR (300 MHz, DMSO-*d*$_6$): δ 8.80 (br s, 3H), 7.45 (d, *J* = 3.6 Hz, 1H), 7.33 – 7.27 (m, 2H), 4.11 (s, 2H), 3.95 (s, 3H).
^{13}C NMR (101 MHz, DMSO-d6): δ145.34 , 135.75 , 135.55 , 132.40 (q, *J* = 38.8 Hz), 130.27 , 125.43 , 120.15 (q, *J* = 268.8Hz), 105.26 , 38.51 , 37.25 . Pale brown solid.

TM5-191

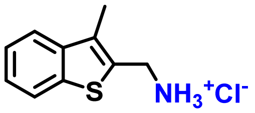

^{1}H NMR (300 MHz, DMSO-*d*$_6$): δ 8.73 (br s, 3H), 8.00 – 7.94 (m, 1H), 7.83 – 7.77 (m, 1H), 7.47 – 7.39 (m, 2H), 4.30 (s, 2H), 2.43 (s, 3H).

^{13}C NMR (75 MHz, DMSO): δ 139.97, 139.09, 132.82, 130.16, 125.67, 124.87, 122.99, 122.94, 35.65, 12.10. Pale yellow solid.

TM5-195

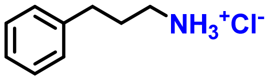

^{1}H NMR (300 MHz, DMSO-d_6): δ 8.38 (br s, 3H), 7.45 – 6.99 (m, 5H), 2.89 – 2.56 (m, 4H), 1.99 – 1.87 (m, 2H).
^{13}C NMR (75 MHz, DMSO): δ 141.35, 128.82, 128.71, 126.41, 38.79, 32.38, 29.10. Off-White solid.

TM5-211

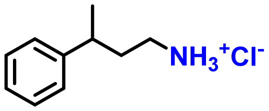

^{1}H NMR (300 MHz, DMSO-d_6): δ 8.19 (br s, 3H), 7.39 – 7.09 (m, 5H), 2.94-2.78 (m, J = 7.1 Hz, 1H), 2.74 – 2.59 (m, 1H), 2.56 – 2.46 (m, 1H), 2.00 – 1.79 (m, 2H), 1.19 (d, J = 6.9 Hz, 3H).
^{13}C NMR (75 MHz, DMSO): δ 146.30, 128.94, 127.25, 126.69, 37.79, 36.85, 35.45, 22.49. Off-White solid.

TM5-188

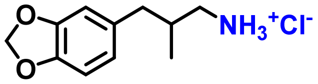

^{1}H NMR (300 MHz, DMSO-d_6): δ 7.99 (br s, 3H), 6.81- 6.76 (m, 2H), 6.67 (dd, J = 8.0, 1.6 Hz, 1H), 5.93 (s, 2H), 3.14 – 1.78 (m, 5H), 0.81 (d, J = 7.3 Hz, 3H).
^{13}C NMR (75 MHz, DMSO): δ 147.52, 145.80, 133.84, 122.40, 109.74, 108.44, 101.13, 44.30, 33.64, 17.28. Off-White solid.

TM5-247

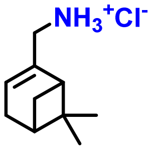

^{1}H NMR (400 MHz, DMSO-d_6): δ 8.31 (br s, 3H), 5.59 (d, J = 3.1 Hz, 1H), 3.34 (s, 2H), 2.56 – 1.91 (m, 6H), 1.25 (s, 3H), 0.79 (s, 3H).
^{13}C NMR (101 MHz, DMSO): δ 141.41, 121.52, 43.66, 43.14, 38.10, 31.41, 31.23, 26.26, 21.30. Brown gum.

TM5-327

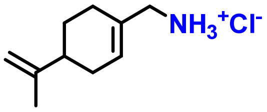

^{1}H NMR (400 MHz, DMSO-d_6): δ 8.39 (br s, 3H), 5.94 – 5.60 (m, 1H), 4.71 (s, 2H), 3.29 (s, 2H), 2.21 – 2.01 (m, 4H), 1.71 (s, 3H), 1.56 – 1.51 (m, 2H).
^{13}C NMR (101 MHz, DMSO): δ 149.31, 131.23, 125.94, 109.47, 48.98, 44.07, 30.29, 27.22, 27.01, 21.02. Colorless gum.

TM5-283

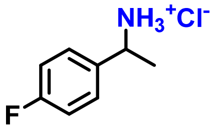

^{1}H NMR (300 MHz, DMSO-d_6): δ 8.66 (br s, 3H), 7.74 – 7.55 (m, 2H), 7.38 – 6.97 (m, 2H), 4.40 (q, J = 6.7 Hz, 1H), 1.53 (d, J = 6.7 Hz, 3H).
13C NMR (75 MHz, DMSO-d6): δ 162.34 (d, J = 244.3 Hz), 136.20 (d, J = 3.1 Hz), 129.72 (d, J = 8.3 Hz), 115.82 (d, J = 21.4 Hz), 49.83 , 21.24. Pale Brown solid.

TM5-338

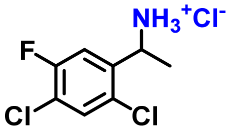

^{1}H NMR (300 MHz, DMSO-d_6): δ 8.88 (br s, 3H), 8.16 (s, 2H), 4.65 (q, J = 6.7 Hz, 1H), 1.51 (d, J = 6.7 Hz, 3H).
^{13}C NMR (75 MHz, DMSO): δ 156.81(d, J=243.2 Hz), 138.49(d, J =6.3 Hz), 131.71(d, J =19.4 Hz), 128.00(d, J =3.1Hz), 120.91(d, J =18.6 Hz), 116.45(d, J =39.4 Hz), 47.29, 20.03. Brown solid.

TM5-337

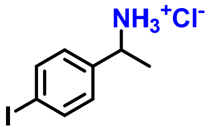

^{1}H NMR (300 MHz, DMSO-d_6): δ 8.84 – 8.57 (br s, 3H), 7.75 (d, J = 7.9 Hz, 2H), 7.36 (d, J = 7.8 Hz, 2H), 4.34 (q, J = 5.9 Hz, 1H), 1.50 (d, J = 6.7 Hz, 3H).
^{13}C NMR (75 MHz, DMSO): δ 139.47, 137.80, 129.78, 95.24, 50.09, 21.03. Brown solid.

TM5-356

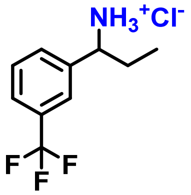

^{1}H NMR (300 MHz, DMSO-d_6): δ 8.83 (br s, 3H), 7.95 (d, J = 1.7 Hz, 1H), 7.87 (d, J = 7.5, Hz, 1H), 7.76 – 7.62 (m, 2H), 4.28 (dd, J = 9.2, 5.5 Hz, 1H), 2.30 – 1.61 (m, 2H), 0.73 (d, J = 7.4 Hz, 3H).
13C NMR (75 MHz, DMSO-d6): δ 139.52 , 132.30 , 130.19 , 129.78 (q, J = 31.8 Hz), 125.50 (q, J = 2.1 Hz), 124.97 (q, J = 3.4 Hz), 124.50 (q, J = 272.2 Hz), 55.75 , 27.76 , 10.29. Brown solid.

TM5-362

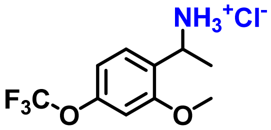

^{1}H NMR (300 MHz, DMSO-d_6): δ 8.74 (br s, 3H), δ 7.81 (d, J = 8.4 Hz, 1H), 7.19 – 6.86 (m, 2H), 4.61 – 4.53 (m,1H). 3.87 (s, 3H), 1.50 (d, J = 6.7 Hz, 3H).
^{13}C NMR (75 MHz, DMSO): δ 157.73 , 149.44 , 128.89 , 126.94 , 122.8 (q, J =255.75 Hz), 112.81 , 105.36 , 56.75 , 44.52 , 19.63. Off-White solid.

TM5-265

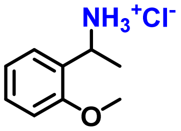

^{1}H NMR (300 MHz, DMSO-d_6): δ 7.82 (br s, 3H), 7.50 (dd, J = 7.6, 1.6 Hz, 1H), 7.35 – 7.25 (m, 1H), 7.04 – 6.91 (m, 2H), 4.53 (q, J = 6.7 Hz, 1H), 3.78 (s, 3H), 1.45 (d, J = 6.7 Hz, 3H).
^{13}C NMR (75 MHz, DMSO): δ 156.39, 130.08, 127.29, 127.21, 120.99, 111.66, 56.15, 45.04, 19.87. White solid.

TM5-202

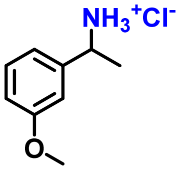

^{1}H NMR (400 MHz, DMSO-d_6): δ 8.77 (br s, 3H), 7.31 (s, 1H), 7.31– 7.20 (d, J = 2.0 Hz, 1H), 7.14 – 7.04 (m, 1H), 6.91 (dd, J = 8.2, 2.4 Hz, 1H), 4.33 (q, J = 6.7 Hz, 1H), 1.52 (d, J = 6.6 Hz, 3H).
^{13}C NMR (101 MHz, DMSO): δ 159.85, 141.54, 130.18, 119.36, 114.20, 113.05, 55.72, 50.50, 21.38. White solid.

TM5-289

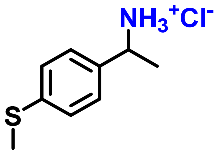

^{1}H NMR (300 MHz, DMSO-d_6): δ 8.61 (br s, 3H), 7.57 (d, J = 7.1 Hz, 2H), 7.36 ((d, J = 7.9 Hz, 2H),), 4.61 – 4.16 (m, 1H), 2.47 (s, 3H), 1.51 (d, J = 6.8 Hz, 3H).
^{13}C NMR (75 MHz, DMSO): δ 138.95, 136.23, 127.97, 126.38, 49.01, 21.02, 15.06. Pale brown solid.

TM5-207

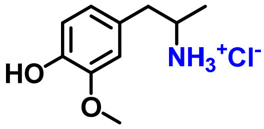

^{1}H NMR (300 MHz, DMSO-d_6): δ 10.05 (br s, 1H), 8.44 (br s, 3H), 6.88 – 6.80 (m, 2H), 6.66 (dd, J = 8.1, 1.9 Hz, 1H), 4.03-3.96 (m, 1H), 3.79 (s, 3H), 3.01 (dd, J = 13.4, 5.1 Hz, 1H), 2.64 (dd, J = 13.3, 8.9 Hz, 1H), 1.18 (d, J = 6.5 Hz, 3H).
13C NMR (75 MHz, DMSO): δ 147.98, 145.75, 127.90, 121.93, 115.95, 113.75, 56.03, 48.80, 40.63,17.97. White solid.

TM5-301

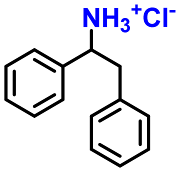

^{1}H NMR (400 MHz, DMSO-d_6): δ 8.11 (br s, 3H), 7.47-7.40 (m, 2H), 7.33 – 7.24 (m, 3H), 7.19 – 6.95 (m, 5H), 4.46 (t, J = 7.7 Hz, 1H), 3.57 – 3.41 (m, 1H), 3.13-3.03 (m, 1H).
^{13}C NMR (101 MHz, DMSO): δ 137.45, 136.73, 129.67, 128.87, 128.83, 128.69, 128.42, 127.01, 56.44, 42.57. Pale Yellow solid.
TM5-346

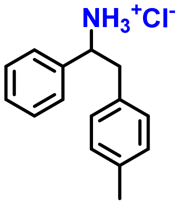

^{1}H NMR (300 MHz, DMSO-d_6): δ 8.10 (br s, 3H), 7.35-7.29 (m, 2H), 7.24-7.19 (m, 2H), 6.97 – 6.78 (m, 5H), 4.36 (dd, J = 10.5, 4.7 Hz, 1H), 3.34 (dd, J = 13.3, 4.8 Hz, 1H), 3.09 – 2.88 (m, 1H). 2.11 (s, 3H).
^{13}C NMR (75 MHz, DMSO): δ 137.21, 135.96, 133.44, 129.47, 129.26, 128.92, 128.85, 128.36, 56.39, 40.46, 21.05. Off-White solid.

TM5-300

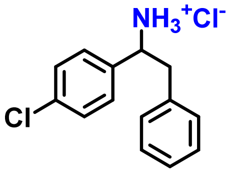

^{1}H NMR (300 MHz, DMSO-d_6): δ 9.02 (br s, 3H), 7.51 (d, J = 8.1 Hz, 2H), 7.38 (d, J = 8.1 Hz, 2H), 7.20 – 7.13 (m, 3H), 7.10 – 6.98 (m, 2H), 4.52 (t, J = 7.3 Hz, 1H), 3.64 – 3.31 (m, 2H).
^{13}C NMR (75 MHz, DMSO): δ 136.54, 136.52, 133.52, 130.44, 129.70, 128.82, 128.77, 127.11, 55.63, 40.50. Pale yellow solid.

TM5-294

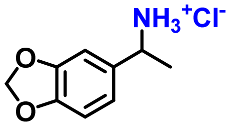

^{1}H NMR (400 MHz, DMSO-d_6): δ 8.56 (br s, 3H), 7.46 (d, J = 1.7 Hz, 1H),7.1 (dd, J = 8.1, 1.7 Hz, 1H), 6.95 (d, J = 8.0 Hz, 1H), 6.21 (s, 2H), 4.29 (q, J = 6.1 Hz, 1H), 1.48 (d, J = 6.8 Hz, 3H).
^{13}C NMR (101 MHz, DMSO): δ 147.82, 147.55, 133.49, 121.12, 108.68, 107.76, 101.65, 50.40, 21.19. Off-White solid.

TM5-279

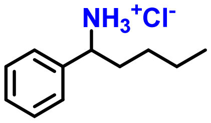

^{1}H NMR (300 MHz, DMSO-d_6): δ 8.77 (br s, 3H), 7.64 – 7.50 (m, 2H), 7.39 – 7.29 (m, 3H).4.35 – 3.93 (m, 1H), 2.24 – 1.63 (m, 2H), 1.40 – 0.89 (m, 4H), 0.78 (t, J = 7.0 Hz, 3H).
^{13}C NMR (75 MHz, DMSO): δ 138.53, 129.07, 128.83, 128.03, 55.00, 34.38, 27.67, 22.12, 14.19. Off-White solid.

TM5-413

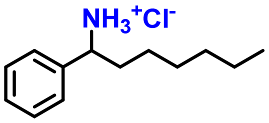

^{1}H NMR (400 MHz, DMSO-d_6): δ 8.41 (br s, 3H), 7.53 – 7.32 (m, 5H), 4.20–4.14 (t, 1H), 2.01–2.65 (m, 2H), 1.33 – 0.94 (m, 8H), 0.90 – 0.62 (m, 3H).
13C NMR (101 MHz, DMSO): δ 138.32, 129.22, 129.04, 127.82, 54.91, 34.65, 31.41, 28.54, 25.41, 22.36, 14.34. Off-white solid.

TM5-298

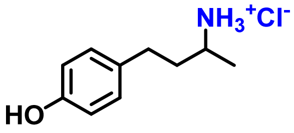

^{1}H NMR (400 MHz, DMSO-d_6): δ 9.30 (br s, 1H), 8.22 (br s, 3H), 7.01(d, J = 8.4 Hz, 2H),6.75 (d, J = 8.4 Hz, 2H),3.08 (dt, J = 12.2, 6.1 Hz, 1H), 2.59 – 2.46 (m, 2H), 1.96 – 1.83 (m, 1H), 1.71–1.64 (m, 1H),1.22 (d, J = 6.5 Hz, 3H).
^{13}C NMR (101 MHz, DMSO): δ 156.03, 131.30, 129.46, 115.65, 46.86, 36.62, 30.47, 18.46. Off-White solid.

TM5-342

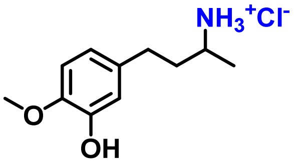

^{1}H NMR (300 MHz, DMSO-d_6): δ 8.82 (br s, 1H), 8.26 (br s, 3H), 7.01 – 6.35 (m, 3H), 3.74 (s, 3H), 3.08 – 2.99 (m, 1H), 2.62 – 2.42 (m, 2H), 2.29 – 1.56 (m, 2H), 1.23 (d, J = 6.3 Hz, 3H). **13C NMR (75 MHz, DMSO):** δ 147.92, 145.15, 132.12, 120.73, 115.86, 112.99, 56.09, 46.89, 36.49, 30.87, 18.48. Off-White solid.

TM5-274 (Diastereomeric mixture)

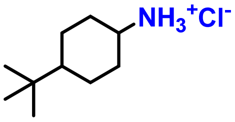

^{1}H NMR (300 MHz, DMSO-d_6): δ 8.18 (br s, 3H), 3.21 – 2.76 (m, 1H), 2.08 – 1.82 (m, 2H), 1.74 – 1.65 (m, 1H), 1.56 – 1.23 (m, 4H), 1.03 – 0.83 (m, 2H), 0.78 (s, 9H).
^{13}C NMR (75 MHz, DMSO): δ 50.05, 47.47, 46.68, 46.15, 32.71, 32.42, 30.81, 28.86, 27.91, 27.79, 25.38, 20.71. White solid.

TM5-206

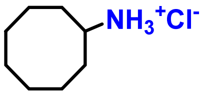

^{1}H NMR (400 MHz, DMSO-d_6): δ 8.21 (br s, 3H), 3.23 – 2.97 (m, 1H), 1.91- 1.84 (m, 2H), 1.63-1.58 (m, 4H), 1.49-1.36 (m, 8H).
^{13}C NMR (101 MHz, DMSO): δ 51.36, 30.30, 26.72, 25.30, 23.30. Brown solid.

TM5-306

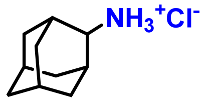

^{1}H NMR (300 MHz, DMSO-d_6): δ 8.40 (br s, 3H), 3.64 – 3.48 (m, 1H), 2.24 – 1.95 (m, 4H), 1.90 – 1.61 (m, 8H), 1.51 (d, *J* = 13.3 Hz, 2H).
^{13}C NMR (75 MHz, DMSO): δ 55.18, 37.34, 36.64, 30.38, 29.97, 26.90, 26.82. White solid.

TM5-282

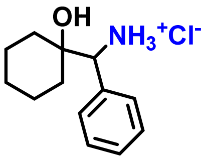

^{1}H NMR (300 MHz, DMSO-d_6): δ 8.35 (br s, 3H), 7.41–7.33 (m, *J* = 7.2, 2.6 Hz, 2H), 7.40 – 7.32 (m, 3H), 5.03 (s, 1H), 4.11 (s, 1H), 1.85 – 1.65 (m, 1H), 1.64 – 1.40 (m, 4H), 1.38 – 0.97 (m, 5H).
^{13}C NMR (75 MHz, DMSO): δ 135.84, 129.42, 128.58, 128.33, 71.25, 63.31, 34.85, 32.98, 25.47, 21.47, 21.12. Brown solid.

TM5-287

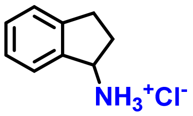

^{1}H NMR (300 MHz, DMSO-d_6): δ 8.50 (br s, 3H), 7.61 (d, *J* = 7.1 Hz, 1H), 7.31 – 7.29 (m, 3H), 4.70 (dd, *J* = 8.5, 4.3 Hz, 1H), 3.07 – 2.99 (m, 1H), 2.87 – 2.79 (m, 1H), 2.44 – 2.31 (m, 1H), 2.06 – 1.93 (m, 1H).
^{13}C NMR (75 MHz, DMSO): δ 144.39, 139.71, 129.44, 127.11,125.38, 125.37, 55.12, 30.73, 30.27. Off-White solid.

TM5-415

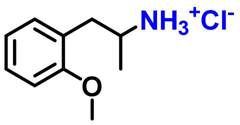

^{1}H NMR (400 MHz, DMSO-d_6): δ 8.38 – 8.08 (br s, 3H), 7.38 – 7.07 (m, 2H), 7.05 – 6.74 (m, 2H), 3.78 (s, 3H), 3.51 – 3.24 (m, 1H), 3.01 (dd, *J* = 13.1, 5.1 Hz, 1H), 2.70 (dd, *J* = 13.1, 9.2 Hz, 1H), 1.09 (d, *J* = 6.5 Hz, 3H).
^{13}C NMR (101 MHz, DMSO): δ 157.72, 131.36, 128.82, 125.04, 120.84, 111.36, 55.79, 47.15, 35.32, 18.10. White solid.

TM-416

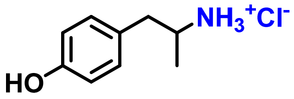

^{1}H NMR (400 MHz, DMSO-*d*$_6$): δ 8.27 (br s,3H), 7.18 (d, *J* = 8.5 Hz, 2H), 6.88 (d, *J* = 8.6 Hz, 2H), 3.40 – 3.17 (m, 1H), 3.01 (dd, *J* = 13.4, 4.9 Hz, 1H), 2.75 – 2.59 (m, 1H), 1.16 (d, *J* = 6.4 Hz, 3H).
^{13}C NMR (101 MHz, DMSO): δ 156.65, 130.57, 127.18, 115.83, 66.80, 48.79, 17.83. Off-white solid.

TM5-207

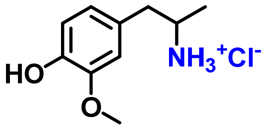

^{1}H NMR (300 MHz, DMSO-*d*$_6$): δ 10.05 (br s, 1H), 8.44 (br s, 3H), 6.88 (d, *J* = 8.0 Hz, 2H),6.66 (dd, *J* = 8.1, 1.9 Hz, 1H), 4.03-3.96 (m, 1H), 3.79 (s, 3H), 3.01 (dd, *J* = 13.4, 5.1 Hz, 1H), 2.64 (dd, *J* = 13.3, 8.9 Hz, 1H), 1.18 (d, *J* = 6.5 Hz, 3H).
13C NMR (75 MHz, DMSO): δ 147.98, 145.75, 127.90, 121.93, 115.95, 113.75, 56.03, 48.80, 40.63,17.97. White solid.

TM-414

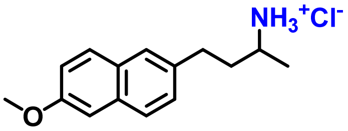

1H NMR (400 MHz, DMSO-d6): δ 8.31 (br s, 3H), 7.81(m, 3H), 7.45 – 6.96 (m, 3H), 3.82 (s, 3H), 3.30 – 3.00 (m, 1H), 2.93 – 2.63 (m, 2H), 2.18 – 1.63 (m, 2H), 1.28 (d, J = 6.5 Hz, 3H). **13C NMR (101 MHz, DMSO):** δ 157.25, 136.54, 133.26, 129.26, 129.01, 128.02, 127.32, 126.40, 118.99, 106.22, 55.59, 47.03, 36.20, 31.28, 18.49. White solid.

TM5-427

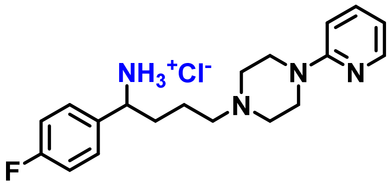

^{1}H NMR (400 MHz, DMSO-*d*$_6$): δ 8.80 (br s, 3H), 8.14 (d, *J* = 6.6 Hz, 2H), 7.76 (d, *J* = 5.3 Hz, 2H), 7.56 (d, *J* = 9.2 Hz, 1H), 7.32 (t, *J* = 8.7 Hz, 2H), 7.14 (d, *J* = 6.6 Hz, 1H), 4.74 – 4.57 (m, 2H), 4.46 – 4.33 (m, 1H), 3.90 – 3.52 (m, 4H), 3.39 – 3.18 (m, 4H), 2.16-2.05 (m, 1H), 1.99-1.87 (m,1H), 1.82-1.73 (m,1H), 1.67-1.55 (m,1H).

^{13}C NMR (101 MHz, DMSO): 162.45 (d, J = 246.7 Hz), 152.41 , 144.56 , 138.10 , 134.51 (d, J = 2.9 Hz),130.44(d, J =8.2 Hz), 115.91 (d, J = 21.2 Hz), 114.38, 113.24, 54.96, 53.51, 50.12, 43.79, 31.73, 19.90. Pale-yellow solid.

TM5-428

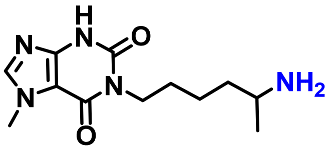

^{1}H NMR (300 MHz, Methanol-d_4): δ 8.07 (s, 1H), 3.92 (s, 3H), 3.91 – 3.85 (m, 2H), 3.22 (d, *J* = 13.4 Hz, 1H), 1.69 – 1.51 (m, 4H), 1.36 (p, *J* = 7.9 Hz, 2H), 1.23 (d, *J* = 6.5 Hz, 3H).
^{13}C NMR (75 MHz, MeOD): δ 154.84, 151.28, 146.82, 141.62, 107.36, 40.57, 33.92, 33.22, 29.19, 27.14, 22.31, 17.34. Off-white solid.

TM5-307 (diastereomeric mixture)

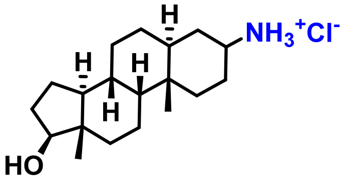

1H NMR (300 MHz, DMSO-d6) δ 8.13 (br s, 3H), 3.48 – 3.15 (m, 2H), 2.09 – 0.99 (m, 22H), 0.89 – 0.66 (m, 3H), 0.67 – 0.35 (m, 3H). **13C NMR (75 MHz, DMSO-d6)** δ 80.48 , 53.63 , 51.17 , 46.69 , 43.01 , 38.40 , 37.10 , 35.98 , 35.49 , 31.59 , 31.26 , 30.97 , 30.29 , 28.22 , 24.28 , 23.50 , 20.40 , 11.79 , 11.55 . White solid.

TM5-363(diastereomeric mixture)

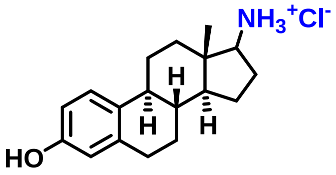

^{1}H NMR (300 MHz, DMSO-d_6): δ 8.14 (br s, 3H), 7.05 (d, *J* = 8.0 Hz, 1H), 6.65 – 6.25 (m, 2H), 3.22-2.71 (m, 3H), 2.45-2.05 (m, 3H), 1.87 – 1.12 (m, 10H), 0.76 (d, *J* = 4.4 Hz, 3H).
13C NMR (101 MHz, DMSO): δ 155.58 , 155.56 , 137.46 , 137.44 , 130.41 , 130.29 , 126.57,126.48 , 115.47 , 115.46 , 113.32 , 113.29 , 59.98 , 59.26 , 51.30 , 47.95 , 43.90 , 43.59,43.29 , 42.52 , 38.75 , 38.70 , 36.26 , 32.41 , 29.67 , 29.56 , 28.40 , 28.28 , 27.53 , 26.73,26.21 , 26.13 , 24.69 , 23.57 , 18.36 , 12.18. White solid.

TM5-311 (diastereomeric mixture)

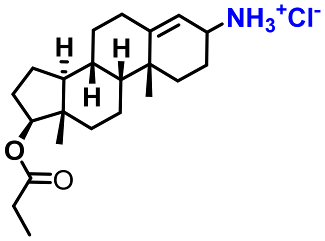

^{1}H NMR (300 MHz, DMSO-d_6): δ 7.52 (br s, 3H), 5.26–5.19 (m, 1H), 4.48 (dd, J = 9.1, 7.6 Hz, 1H), 3.62–3.53 (m, 2H), 2.36 – 1.18 (m, 17H), 1.09 – 0.89 (m, 8H), 0.67 (t, J = 34.4 Hz, 3H), 0.63-0.59 (m, 1H).
^{13}C NMR (75 MHz, DMSO): δ 174.07, 149.61, 117.36, 82.11, 53.97, 50.13, 47.73, 42.59, 37.14, 35.46, 32.37, 31.99, 29.09, 27.50, 24.21, 23.49, 20.58, 18.93, 12.31, 9.61. **HRMS (ESI):** Calcd for C22H35O2N2 [M]+ 345.26601; found 345.26632. Off-white solid.

TM5-493

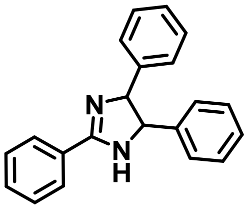

^{1}H NMR (300 MHz, DMSO-d_6): δ 8.13-8.01 (m, 2H), 7.60 – 7.42 (m, 4H), 7.09 – 6.92 (m, 10H), 5.45 (s, 2H).
^{13}C NMR (75 MHz, DMSO): δ 164.25, 140.51, 131.08, 130.79, 128.84, 128.02, 127.91, 127.71, 126.69, 70.03. . **HRMS (ESI):** Calcd for C21H18N2 [M]+ 298.14657; found 298.14645. Pale yellow gum.

<u>**AAS analysis of purified benzylamine to determine Ru content**</u>

Dateiname 18062102

Instrument: contrAA 800D # Tech: Flamme
SW-Version: ASpect CS 2.2.1.0 Created: 21.06.2018 10:32

Datum	Zeit	Name(2)	Linie	Konz.2	Einheit	Ext.
21.06.2018	10:18	Thiru TM 5	Ru349	Messwert 0.000	M-%	0.000
21.06.2018	10:19	Thiru TM 5	Ru349	Ru n.n.	M-%	0.000

AAS measurement for the detection of metal content in the isolated and purified amine product.

Supplementary Figures

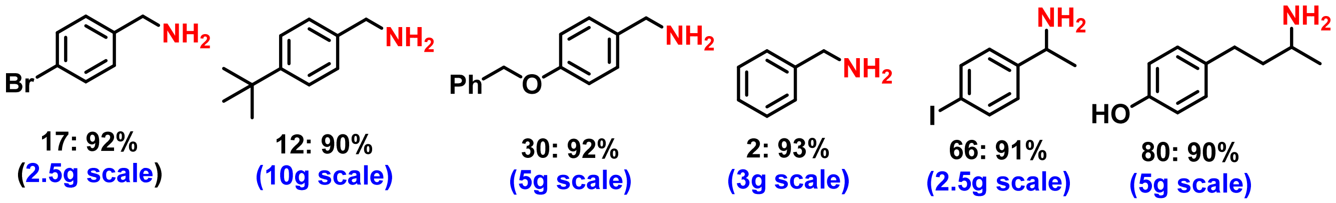

Supplementary Figure 1. Demonstrating the synthetic utility for gram-scale reactions.

Reaction conditions: 2-20 g carbonyl compound, 2-3 mol% of $RuCl_2(PPh_3)_3$ (2 mol% in case of aldehyde, 3 mol% in case of ketone), 5-7 bar NH_3, 40 bar H_2, 25-150 mL t-amyl alcohol, 130 °C, 24-30 h. Isolated as free amines and converted to hydrochloride salts. Corresponding hydrochloride salts were subjected to NMR analysis.

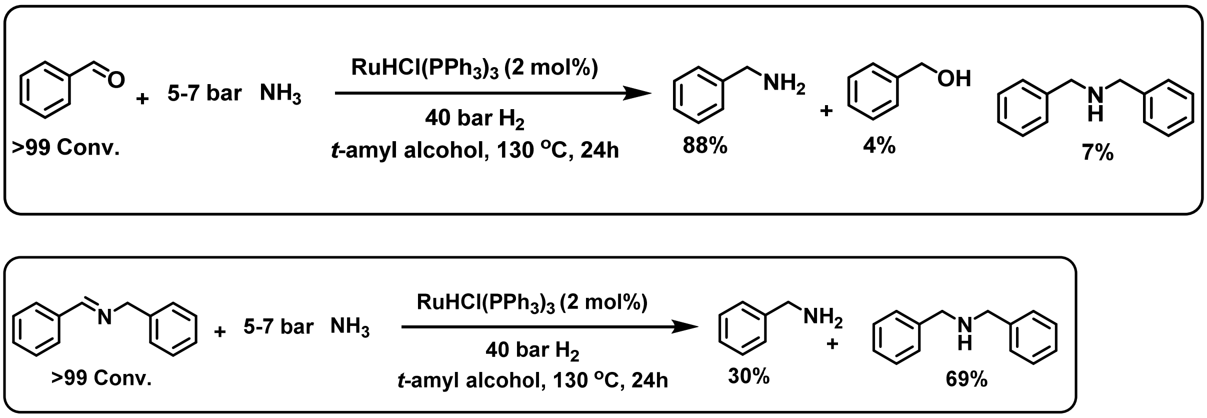

Supplementary Figure 2. Reductive amination of benzaldehyde and N-benzylidenebenzylamine using $RuHCl(PPh_3)_3$.

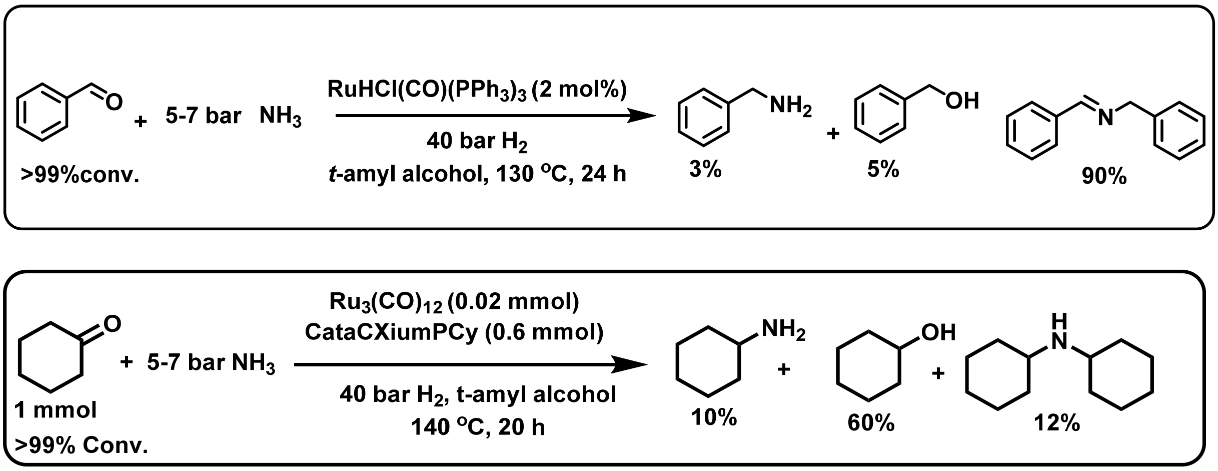

Supplementary Figure 3. Reductive amination of benzaldehyde using $RuHCl(CO)(PPh_3)_3$ and cyclohexanone using $Ru_3(CO)_{12}$/CataCxiumPCy.

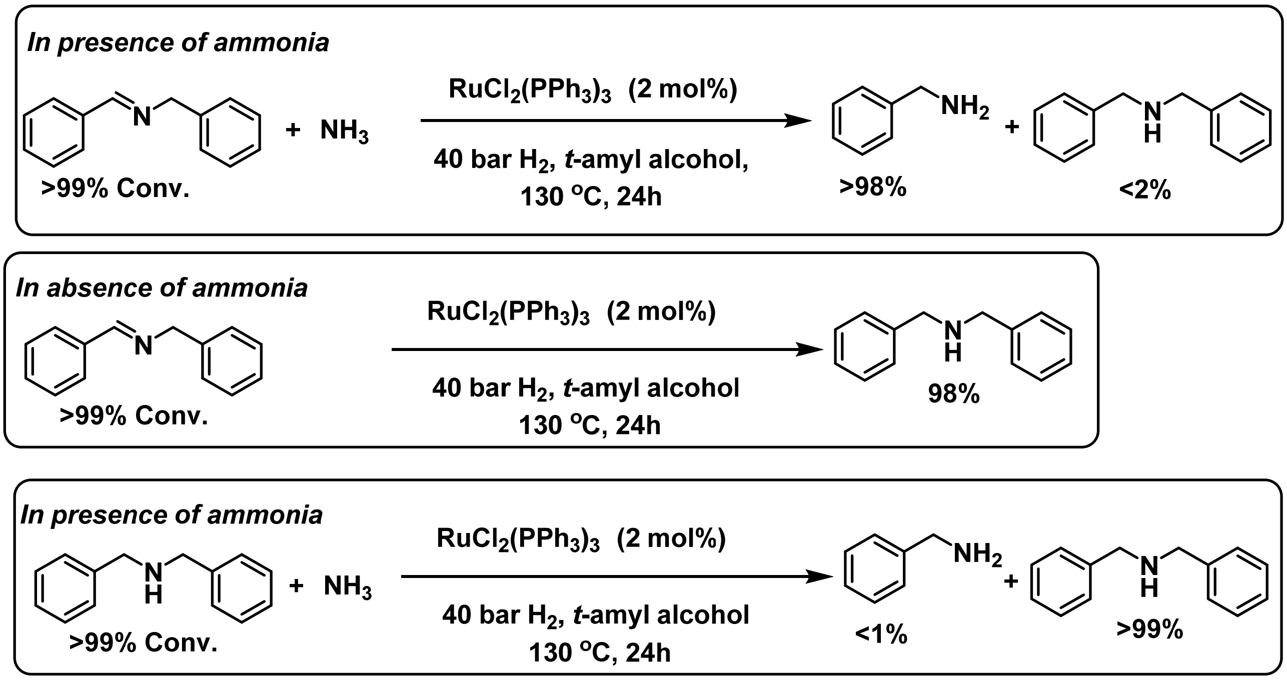

Supplementary Figure 4. Reaction of N-benzylidenebenzylamine and dibenzylamine in presence and absence of ammonia with $RuCl_2(PPh_3)_3$ and hydrogen.

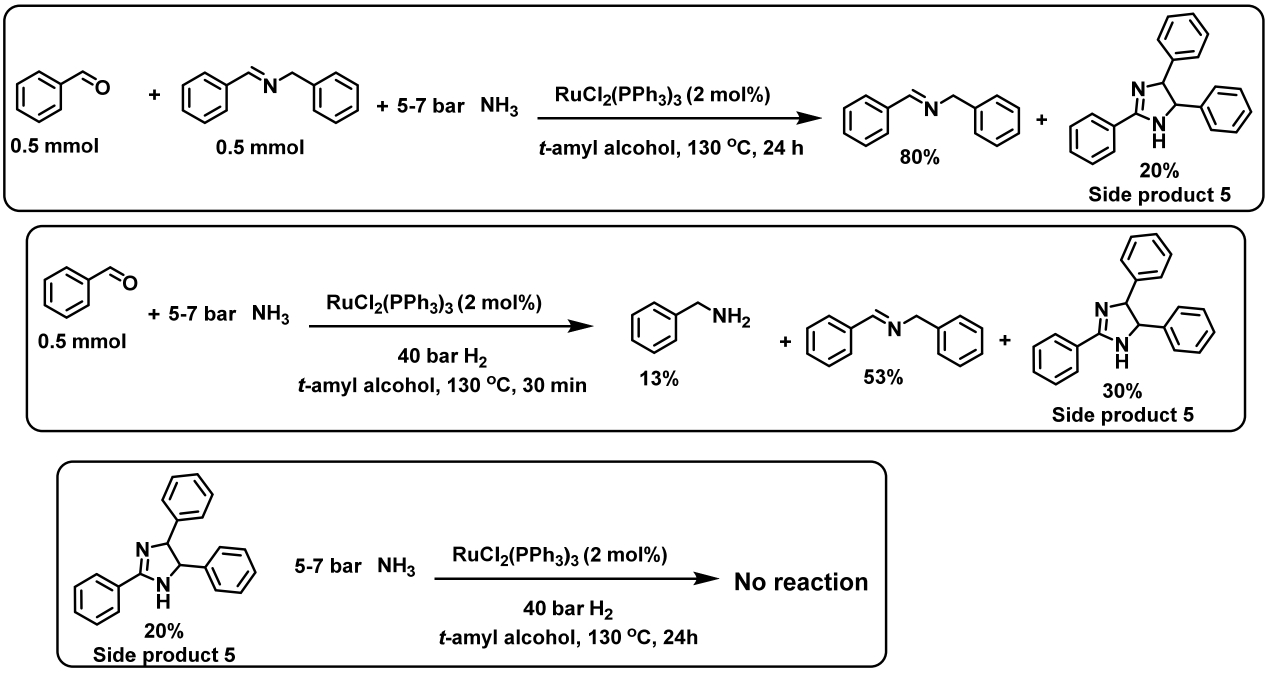

Supplementary Figure 5. Controll reactions to confirm the formation of side product 5.

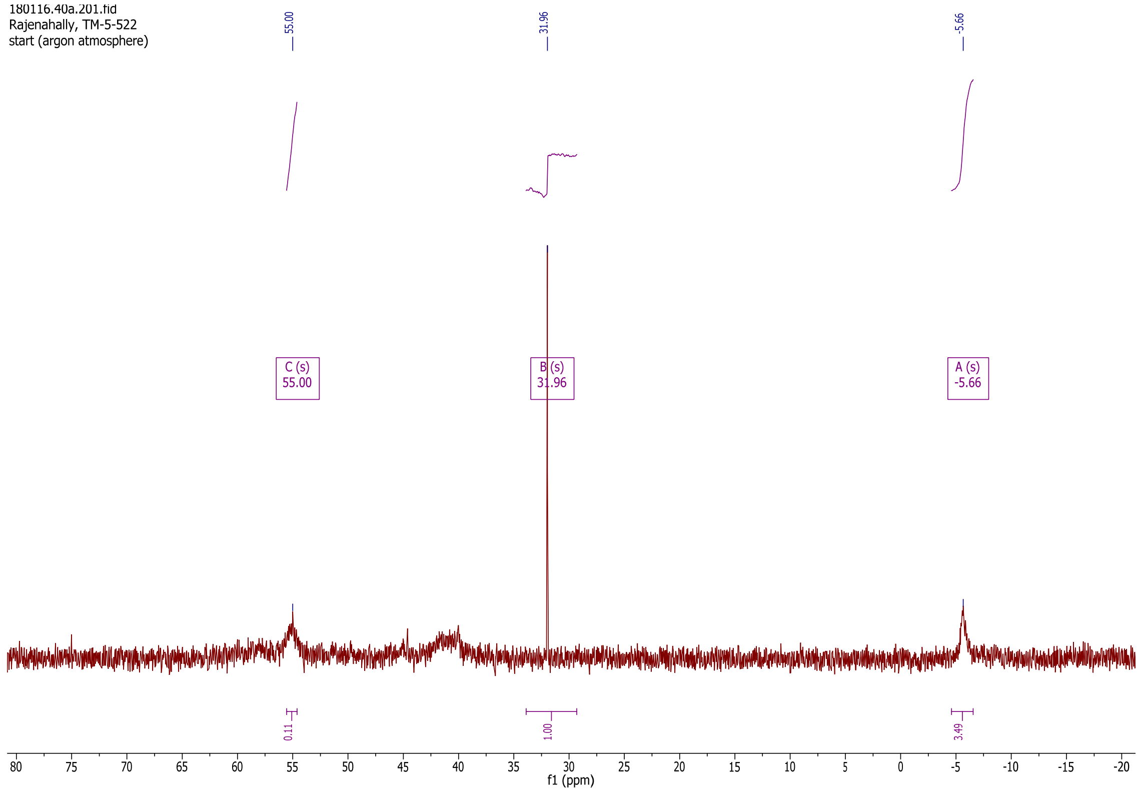

Supplementary Figure 6. $^{31}P\{^{1}H\}$ NMR (162 MHz, -20 to 80 ppm) of $RuCl_2(PPh_3)_3$ in methanol, C_6D_6 at RT, argon atmosphere.

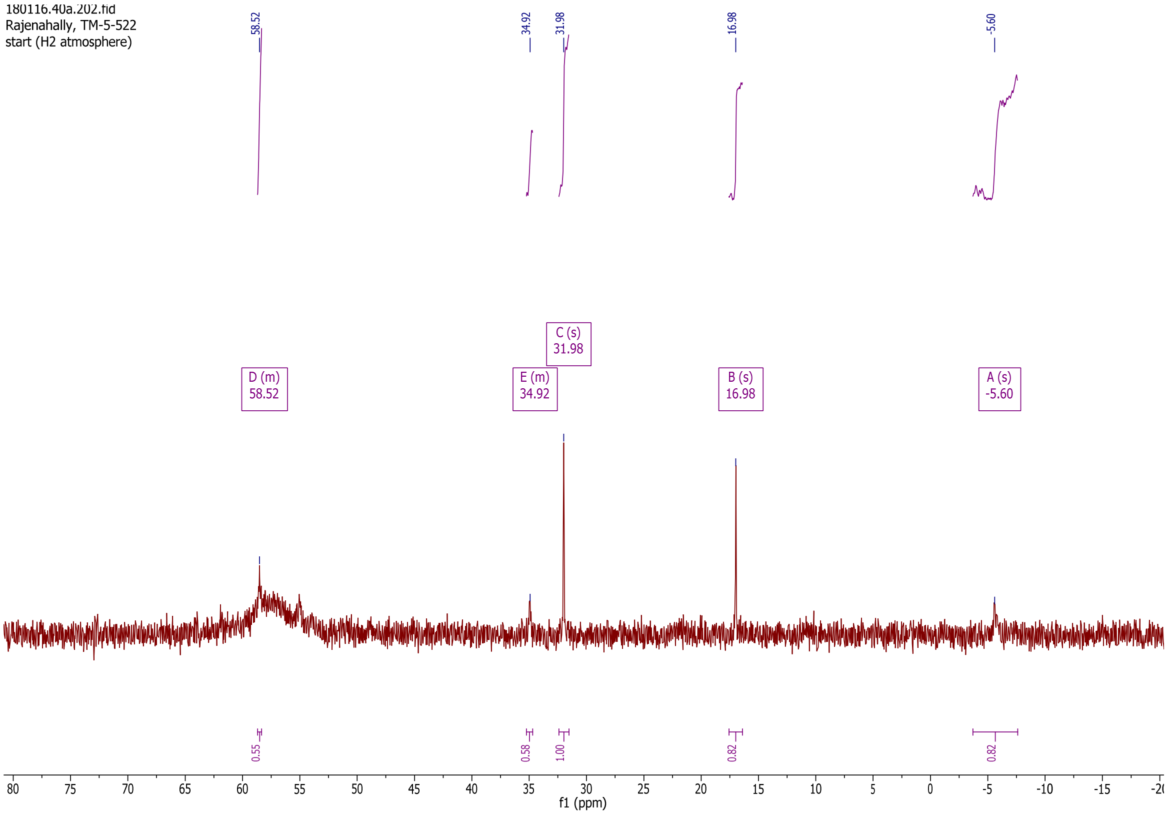

Supplementary Figure 7. $^{31}P\{^{1}H\}$ NMR (162 MHz, -20 to 80 ppm) of $RuCl_2(PPh_3)_3$ in methanol, C_6D_6 at RT, H_2 atmosphere (1.5 bar), 10 min.

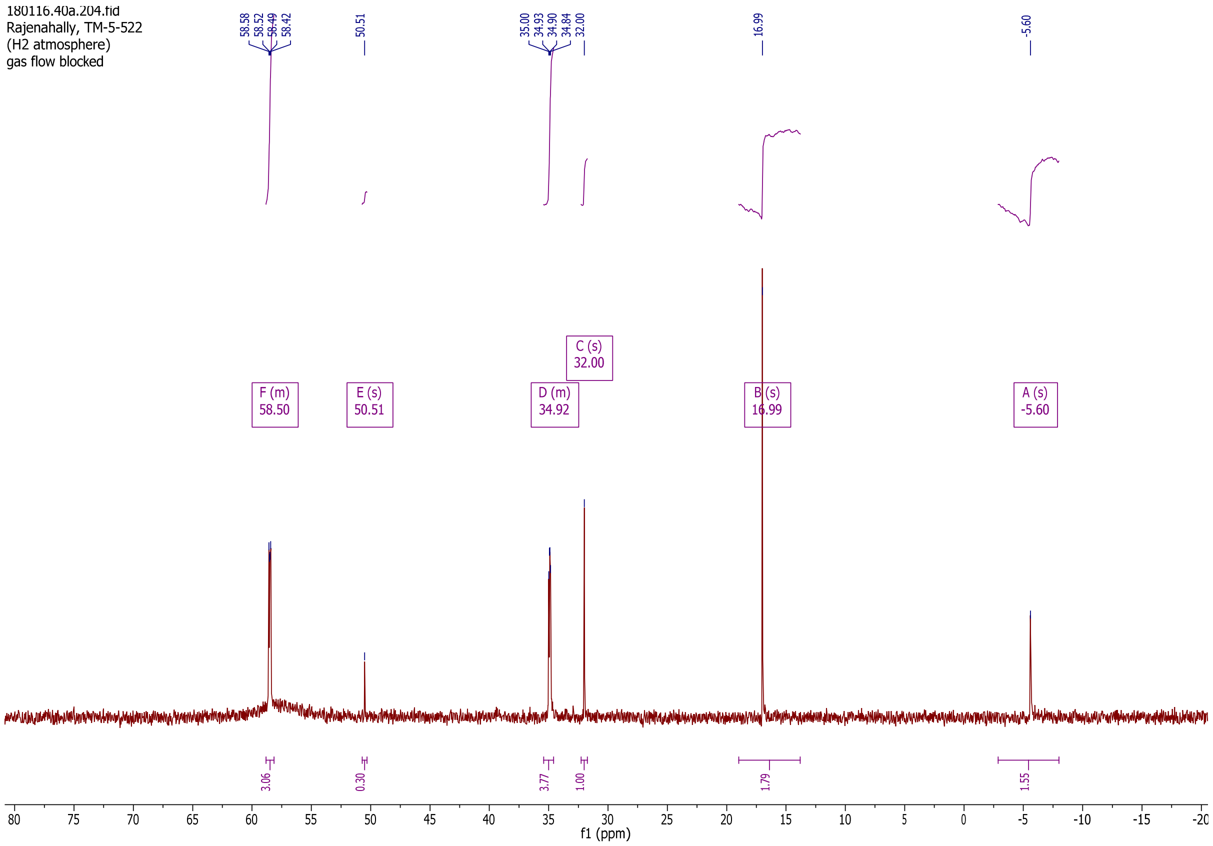

Supplementary Figure 8. $^{31}P\{^{1}H\}$ NMR (162 MHz, -20 to 80 ppm) of $RuCl_2(PPh_3)_3$ in methanol, C_6D_6 at RT, H_2 atmosphere (1.5 bar), 2.5h.

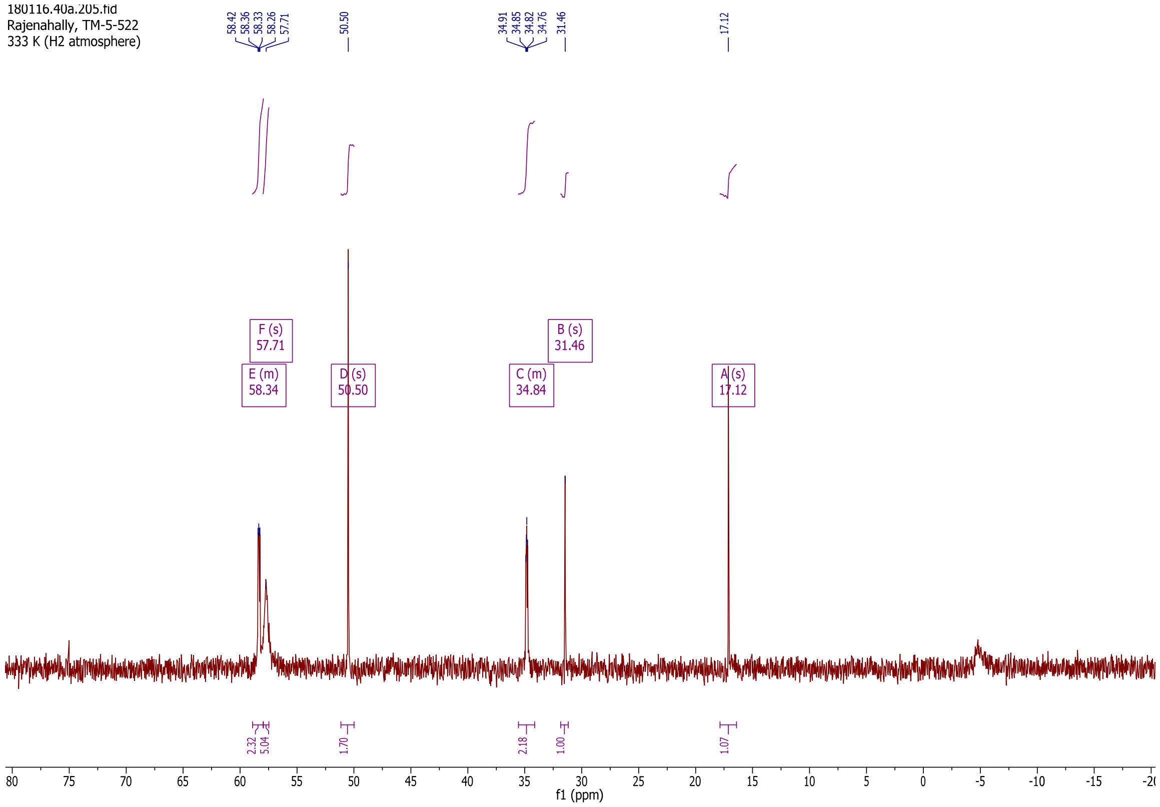

Supplementary Figure 9. $^{31}P\{^{1}H\}$ NMR (162 MHz, -20 to 80 ppm) of $RuCl_2(PPh_3)_3$ in methanol, C_6D_6 at 60°C, H_2 atmosphere (1.5 bar), 30 min.

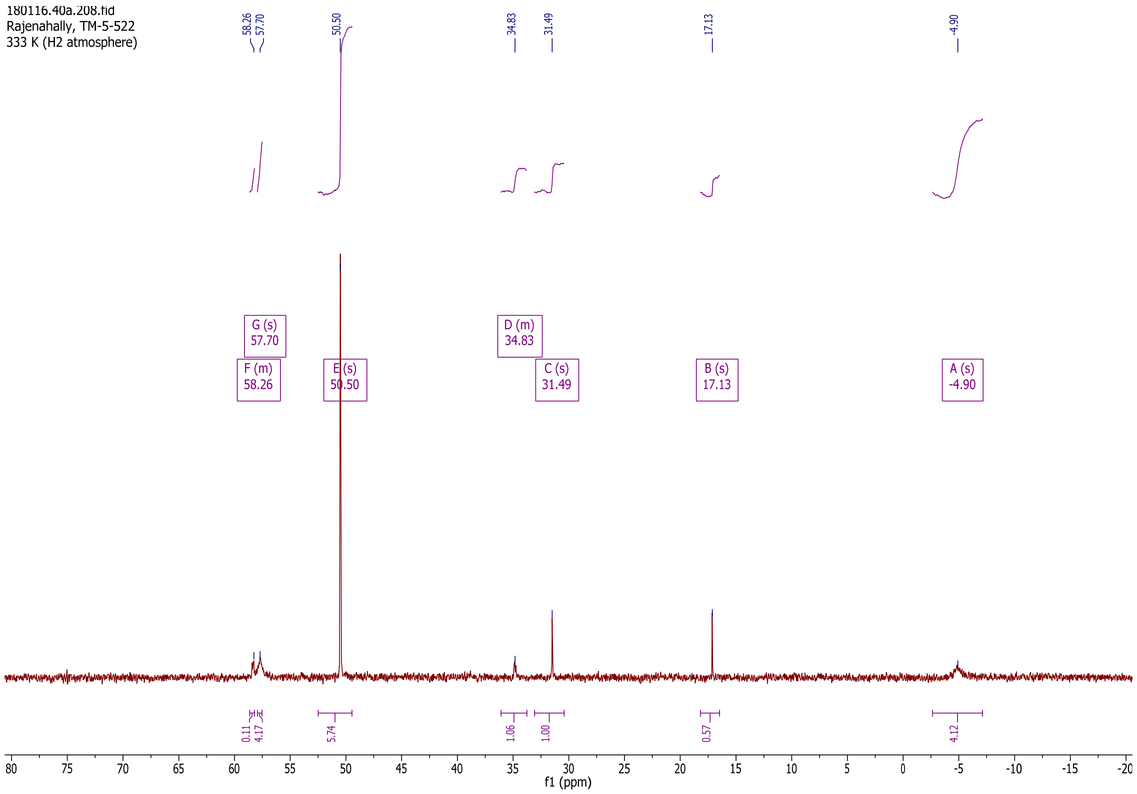

Supplementary Figure 10. $^{31}P\{^{1}H\}$ NMR (162 MHz, -20 to 80 ppm) of $RuCl_2(PPh_3)_3$ in methanol, C_6D_6 at 60°C, H_2 atmosphere (1.5 bar), 2.5h.

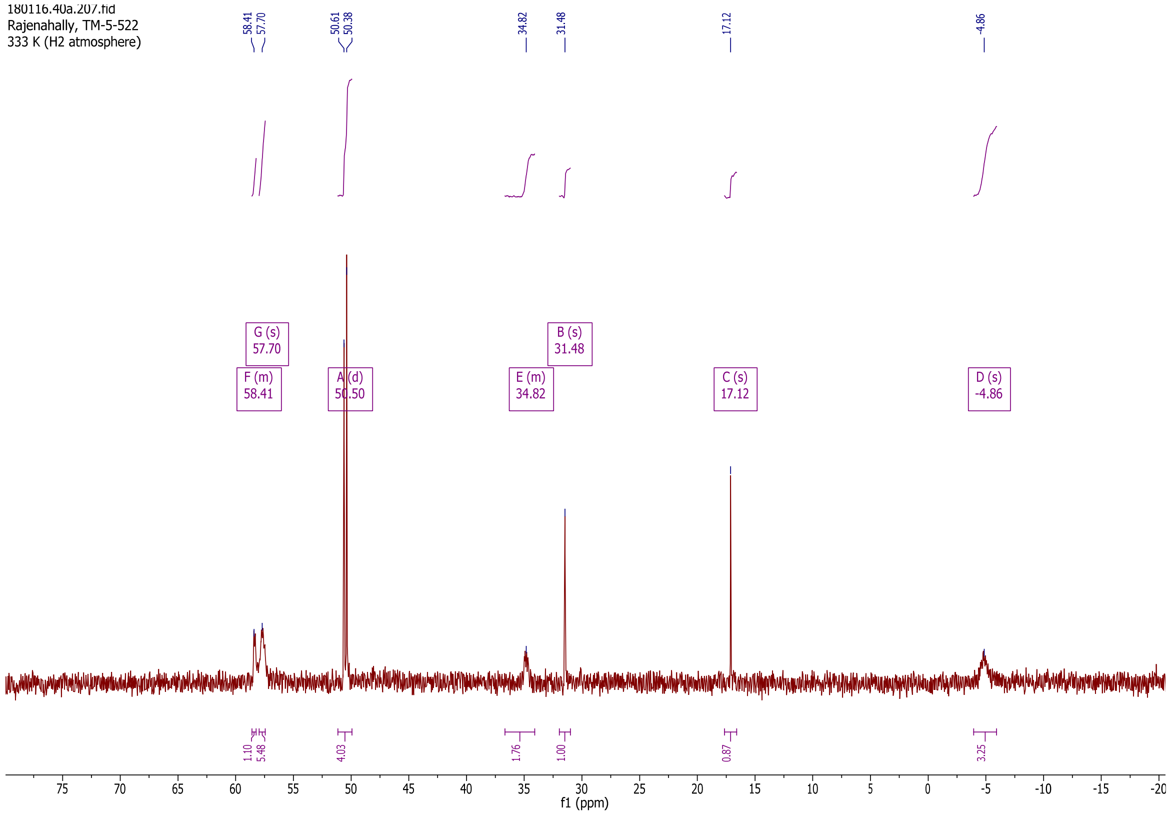

Supplementary Figure 11. $^{31}P\{^{1}H\}$ NMR (162 MHz, -20 to 80 ppm) of $RuCl_2(PPh_3)_3$ in methanol, C_6D_6 at 60°C, H_2 atmosphere (1.5 bar), 1.5h, decoupled with reduced power (only aromatic protons are decoupled).

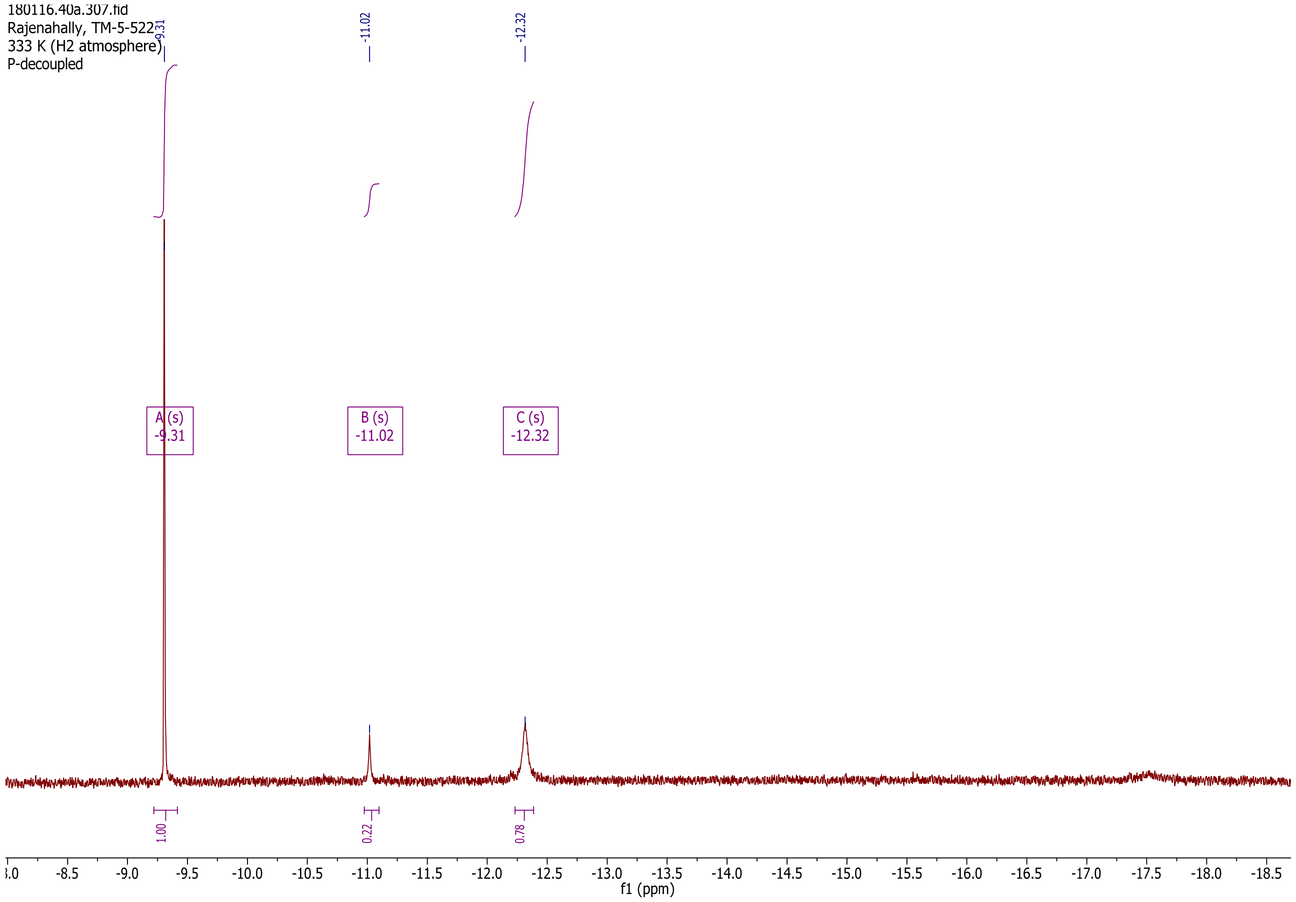

Supplementary Figure 12. $^{1}H\{^{31}P\}$ NMR (400 MHz, -18 to -8 ppm) of $RuCl_2(PPh_3)_3$ in methanol, C_6D_6 at 60°C, H_2 atmosphere (1.5 bar), 1.5h.

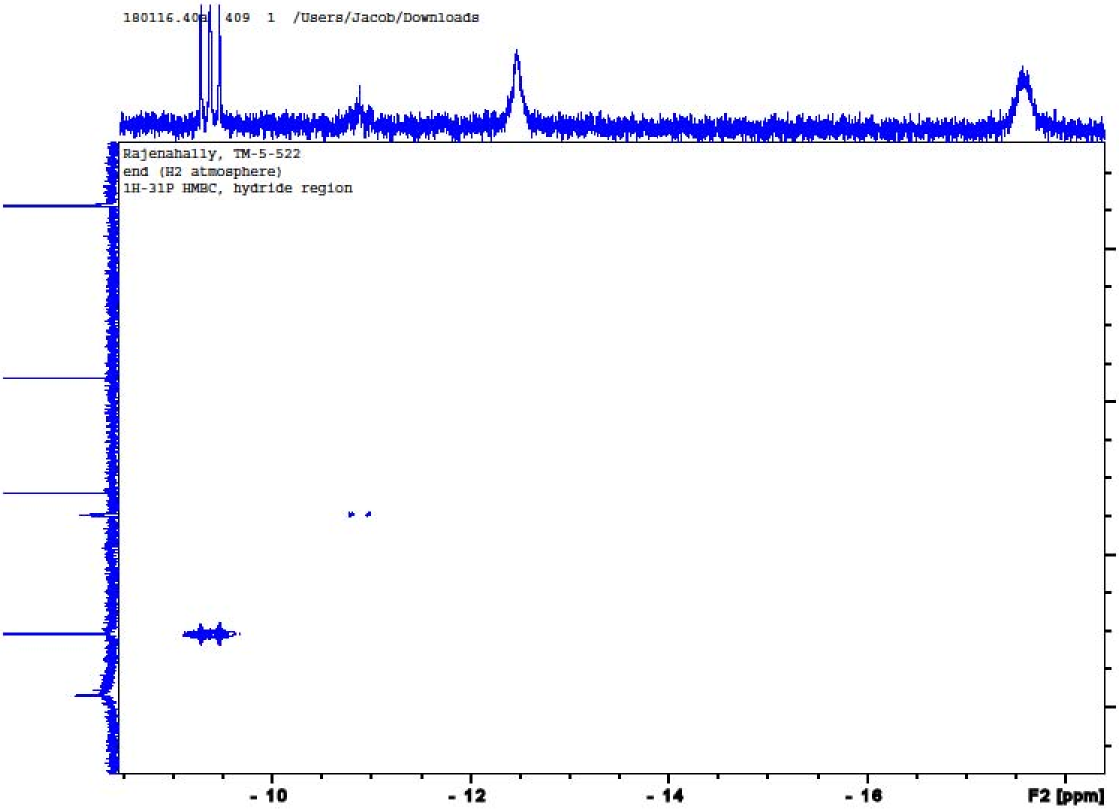

Supplementary Figure 13. ^{1}H-^{31}P HMBC NMR (400 MHz for ^{1}H) of $RuCl_2(PPh_3)_3$ in methanol, C_6D_6 after 2.5h at 60°C under H_2 atmosphere (1.5 bar), measured at RT under H_2 atmosphere.

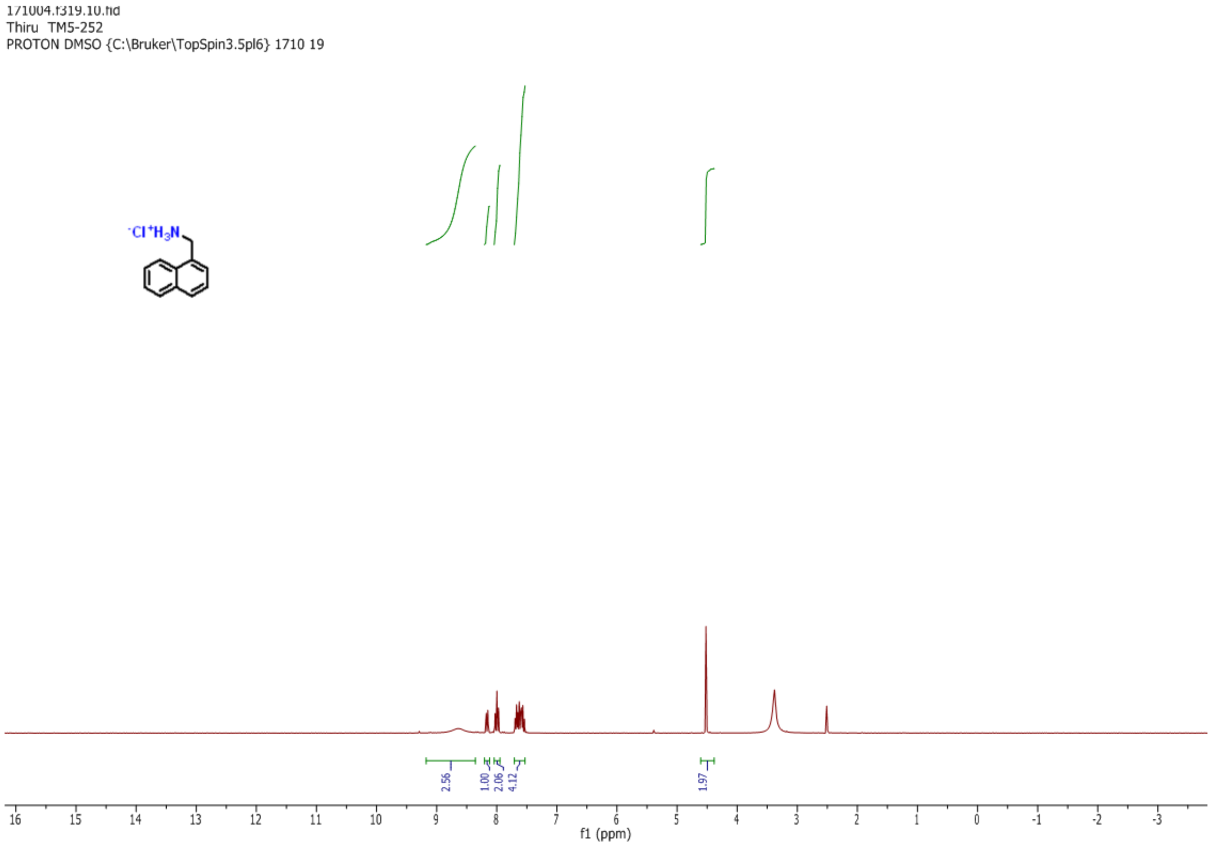

Supplementary Figure 14. ^{1}H NMR spectrum

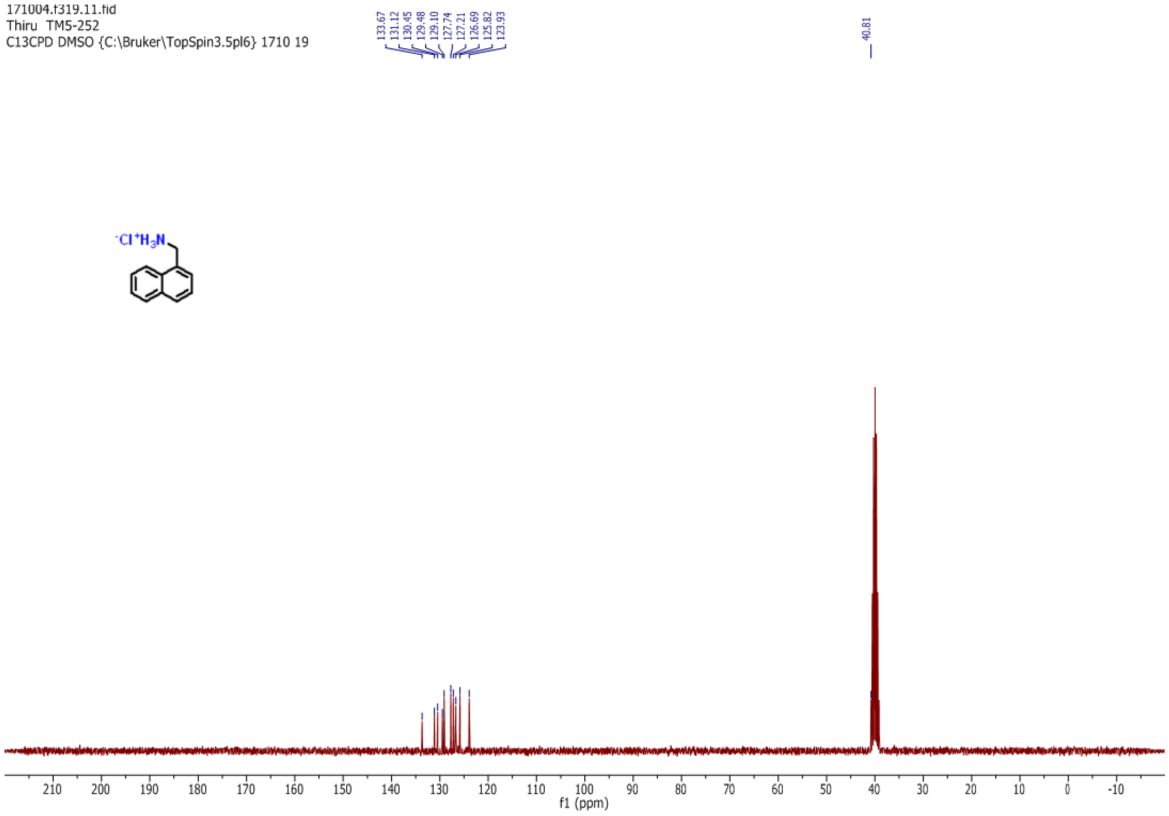

Supplementary Figure 15. ^{13}C NMR spectrum

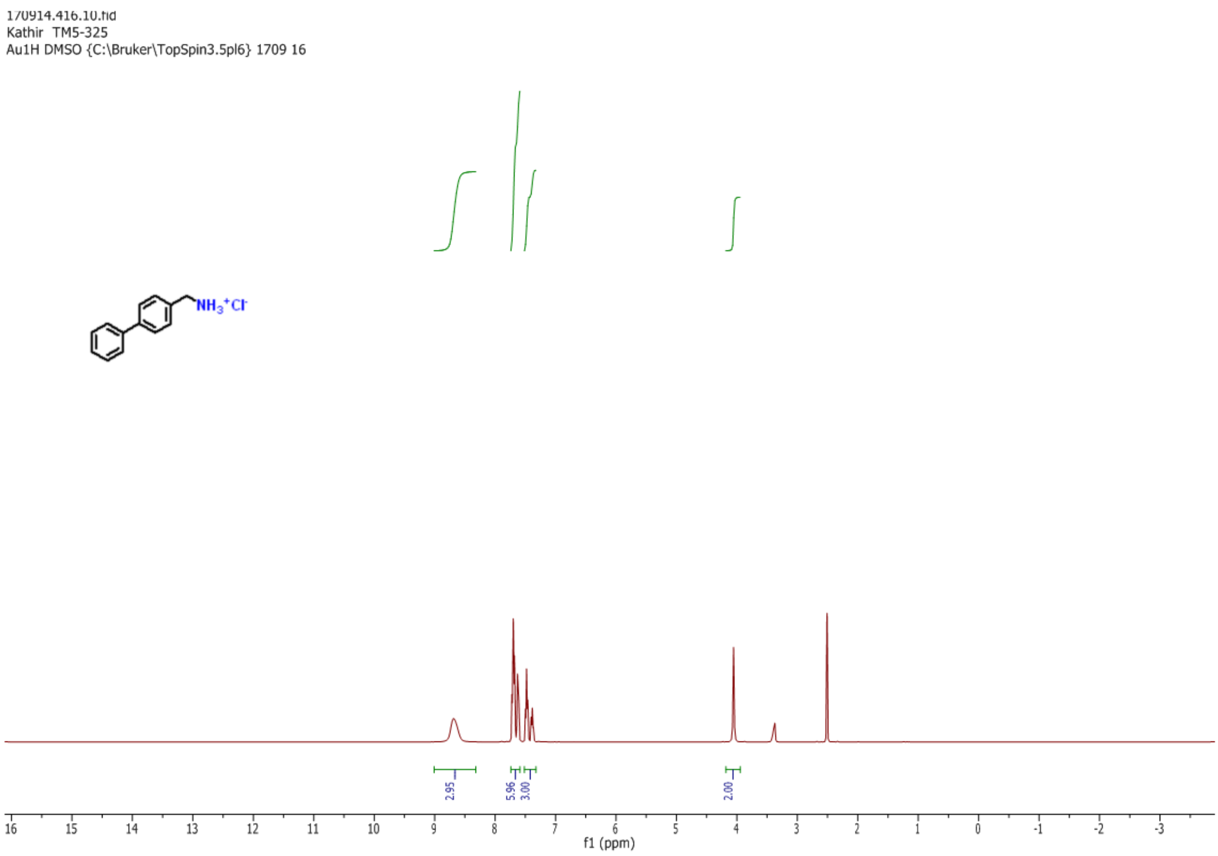

Supplementary Figure 16. ^{1}H NMR spectrum

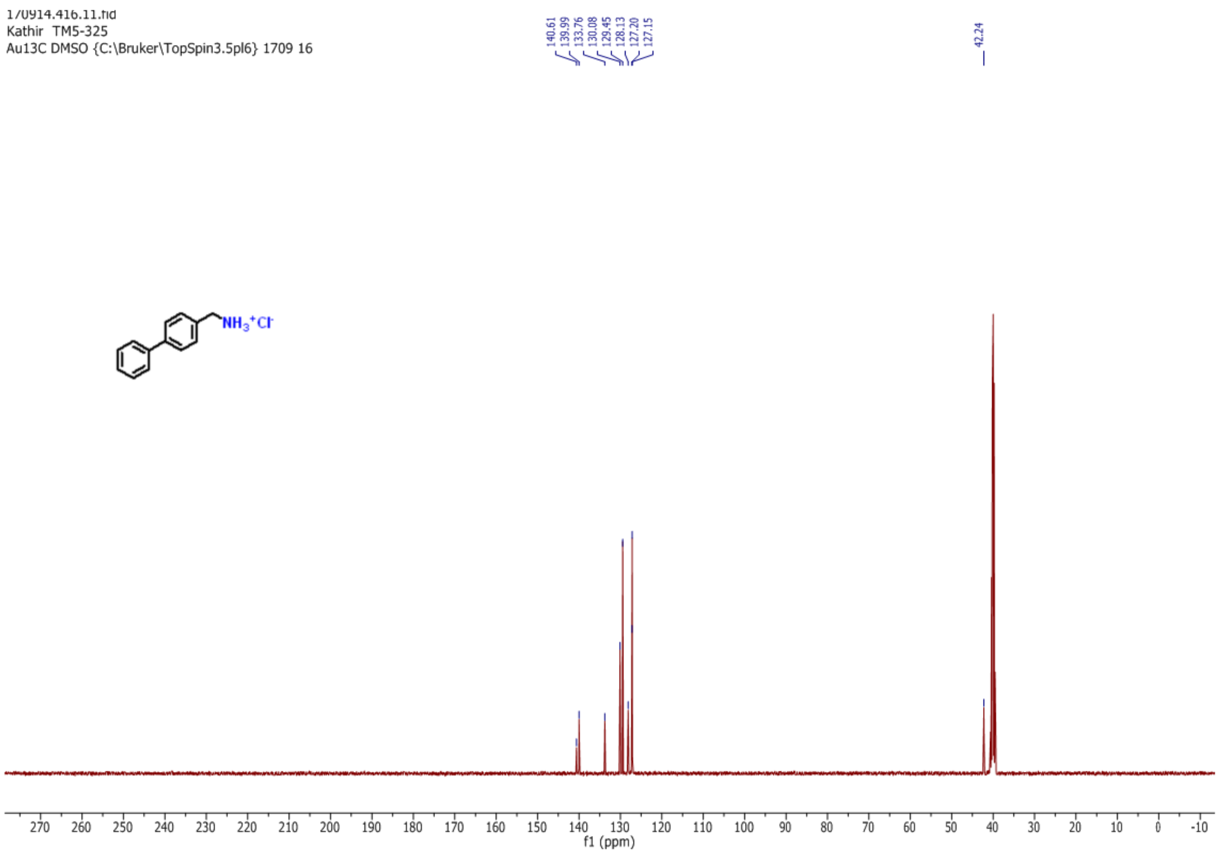

Supplementary Figure 17. ^{13}C NMR spectrum

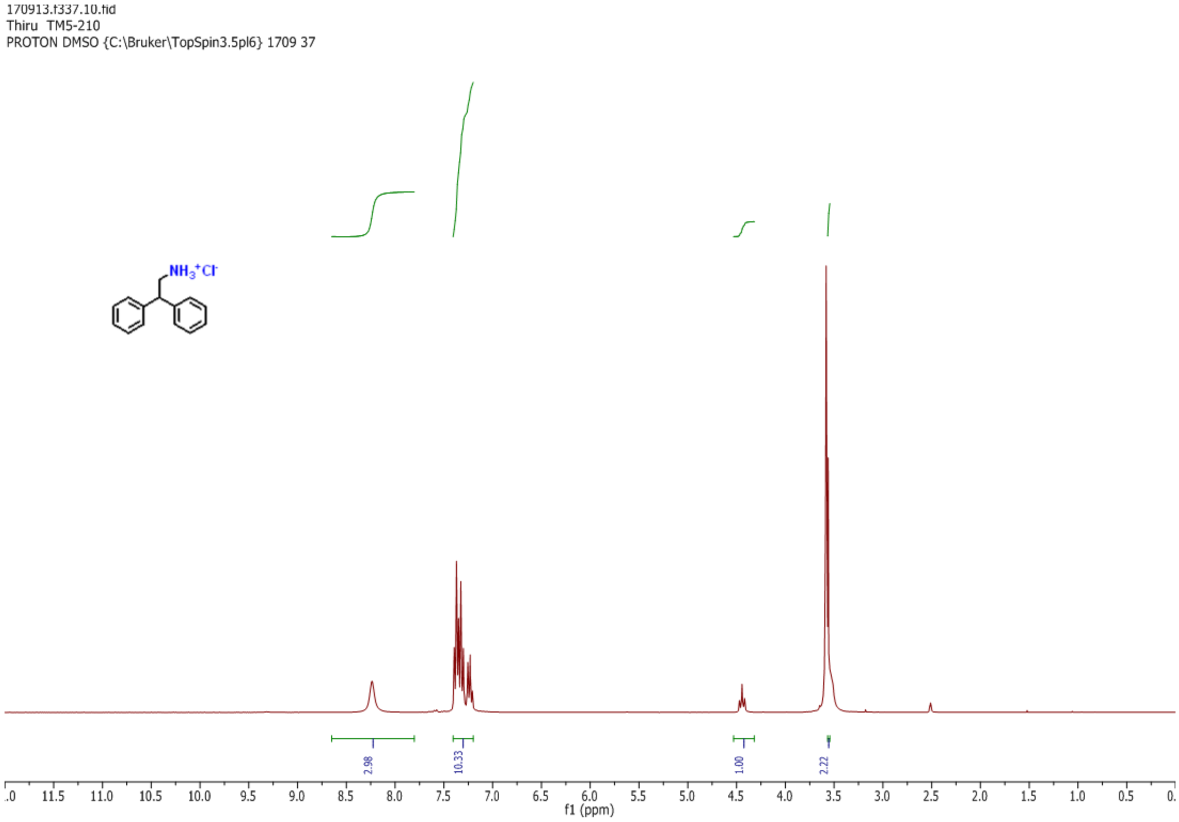

Supplementary Figure 18. ^{1}H NMR spectrum

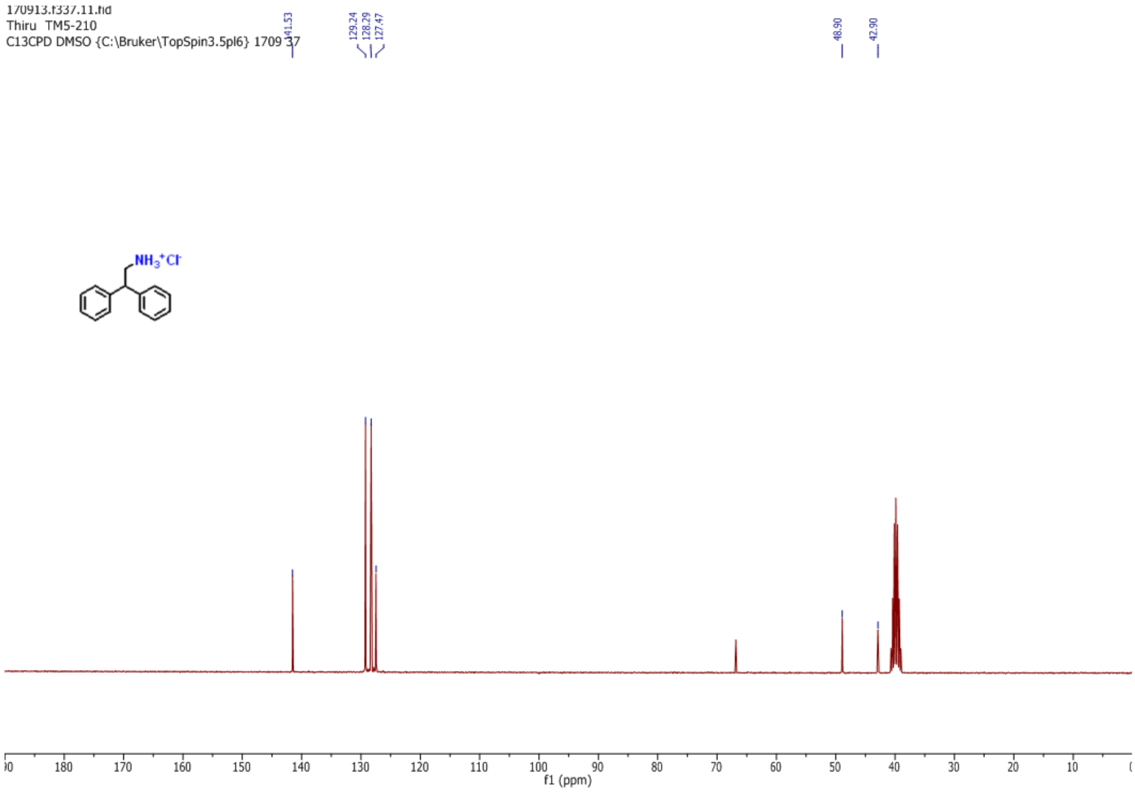

Supplementary Figure 19. ^{13}C NMR spectrum

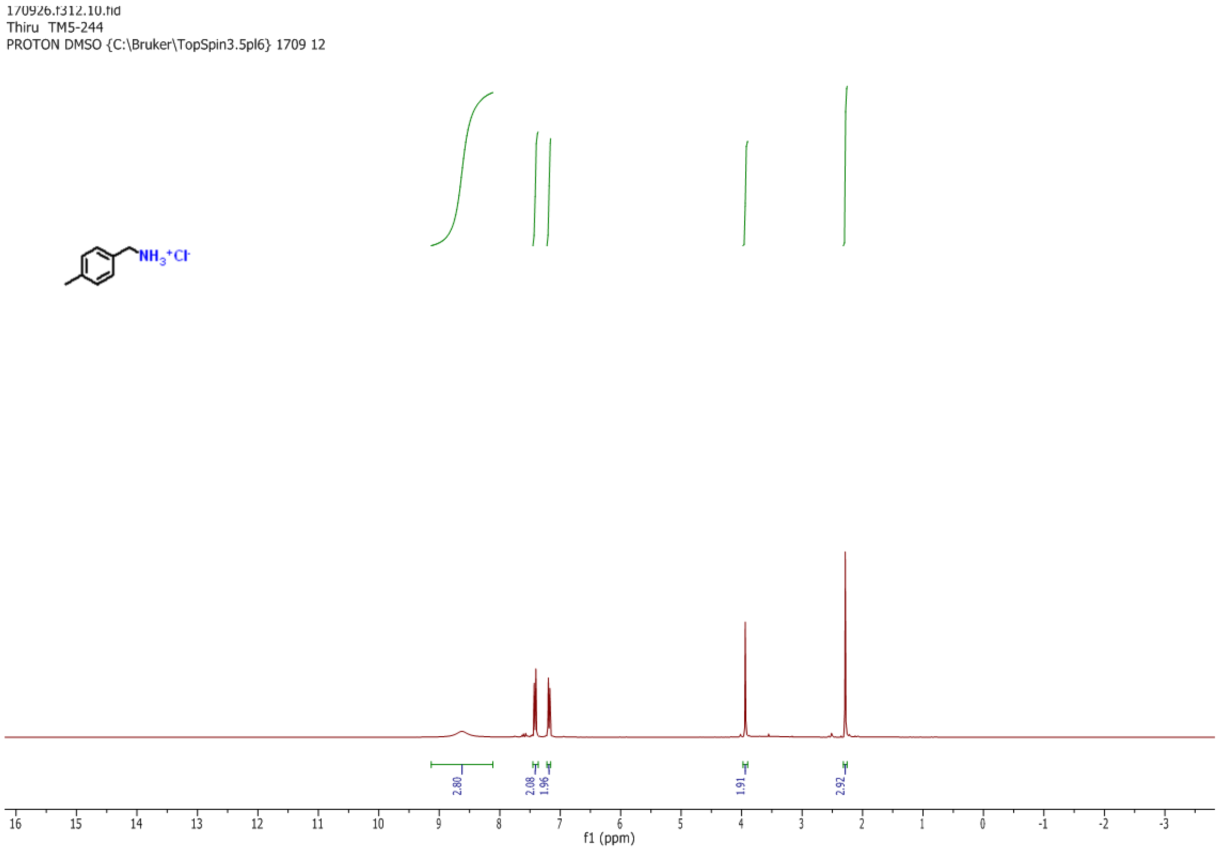

Supplementary Figure 20. ^{1}H NMR spectrum

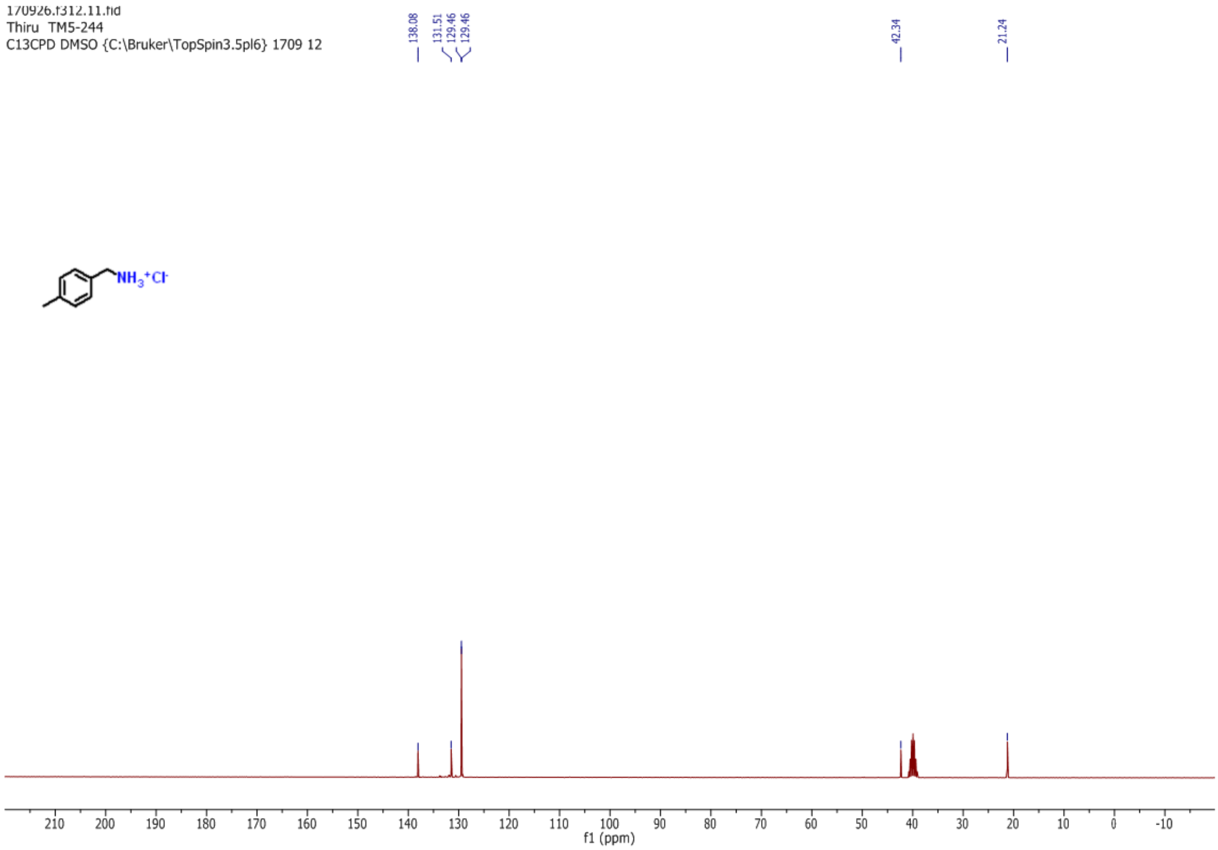

Supplementary Figure 21. ^{13}C NMR spectrum

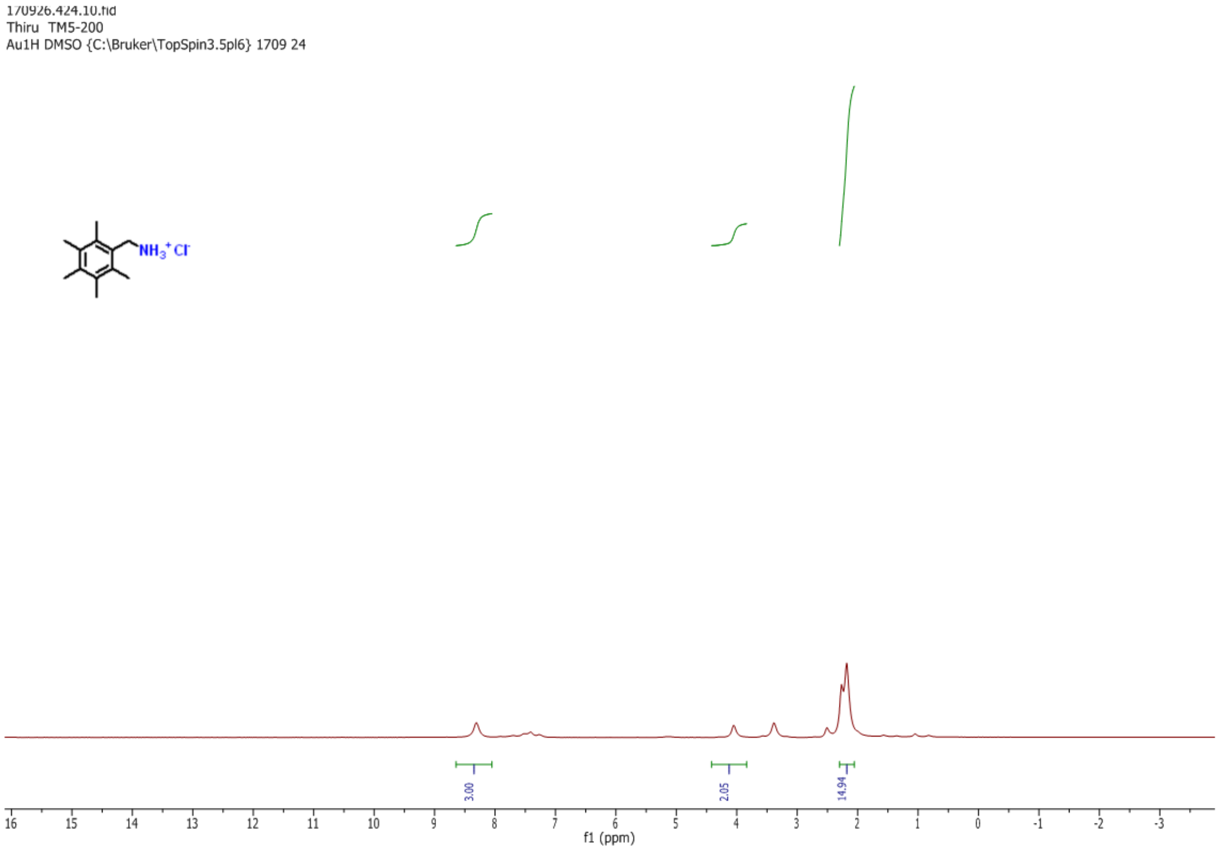

Supplementary Figure 22. ^{1}H NMR spectrum

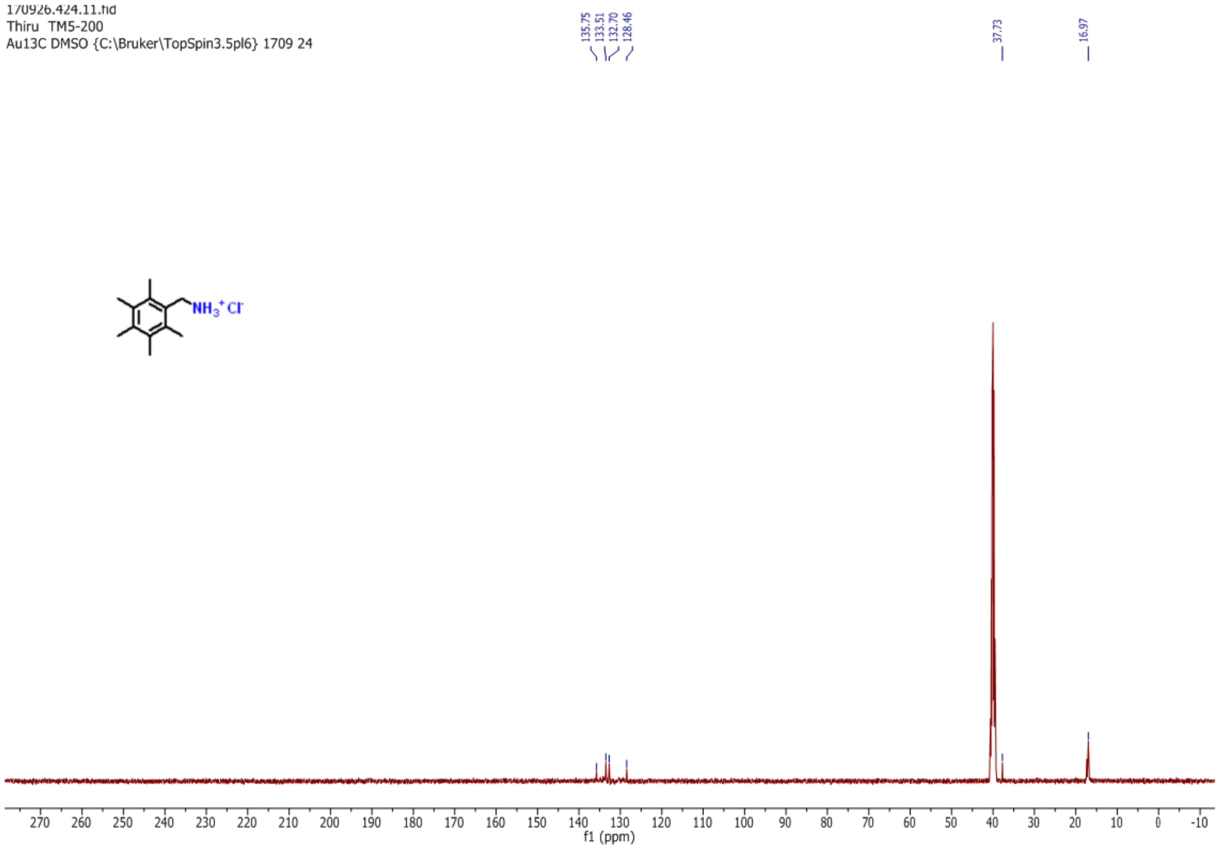

Supplementary Figure 23. ^{13}C NMR spectrum

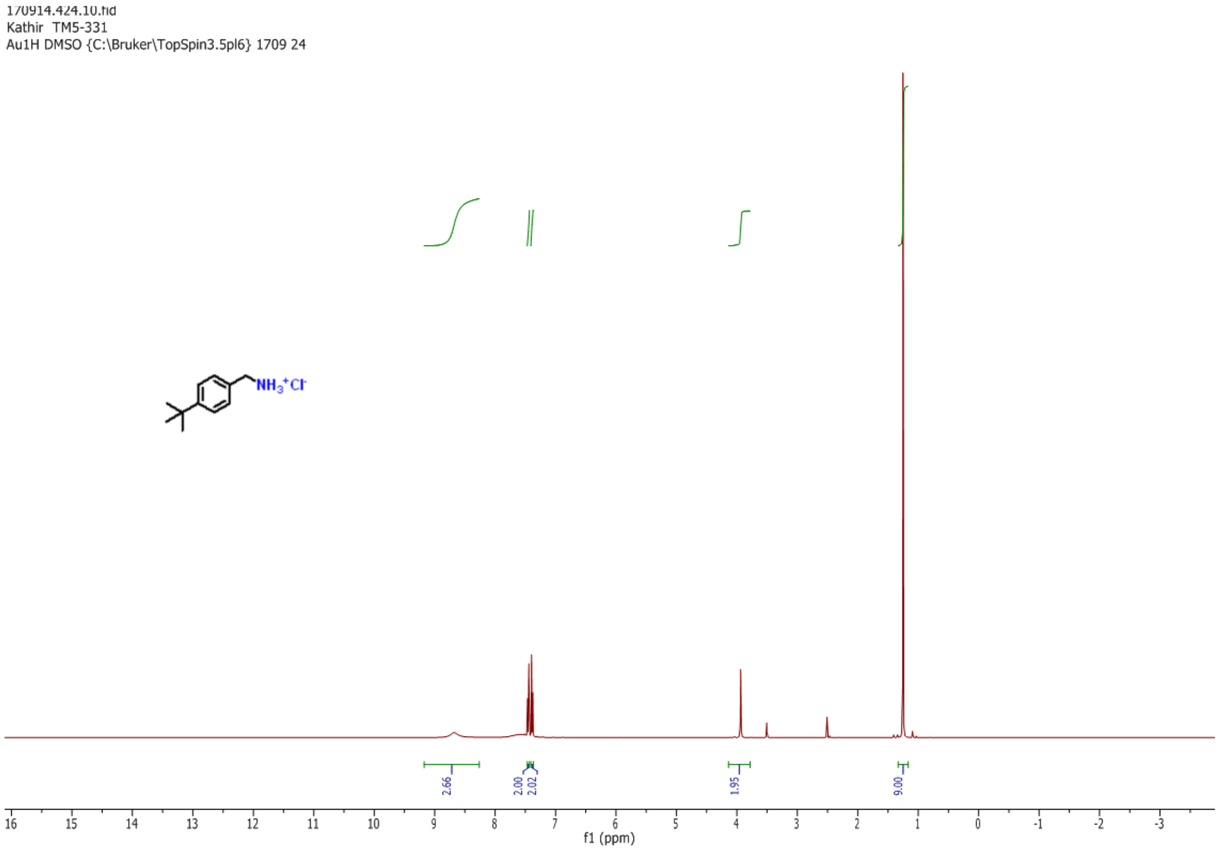

Supplementary Figure 24. ^{1}H NMR spectrum

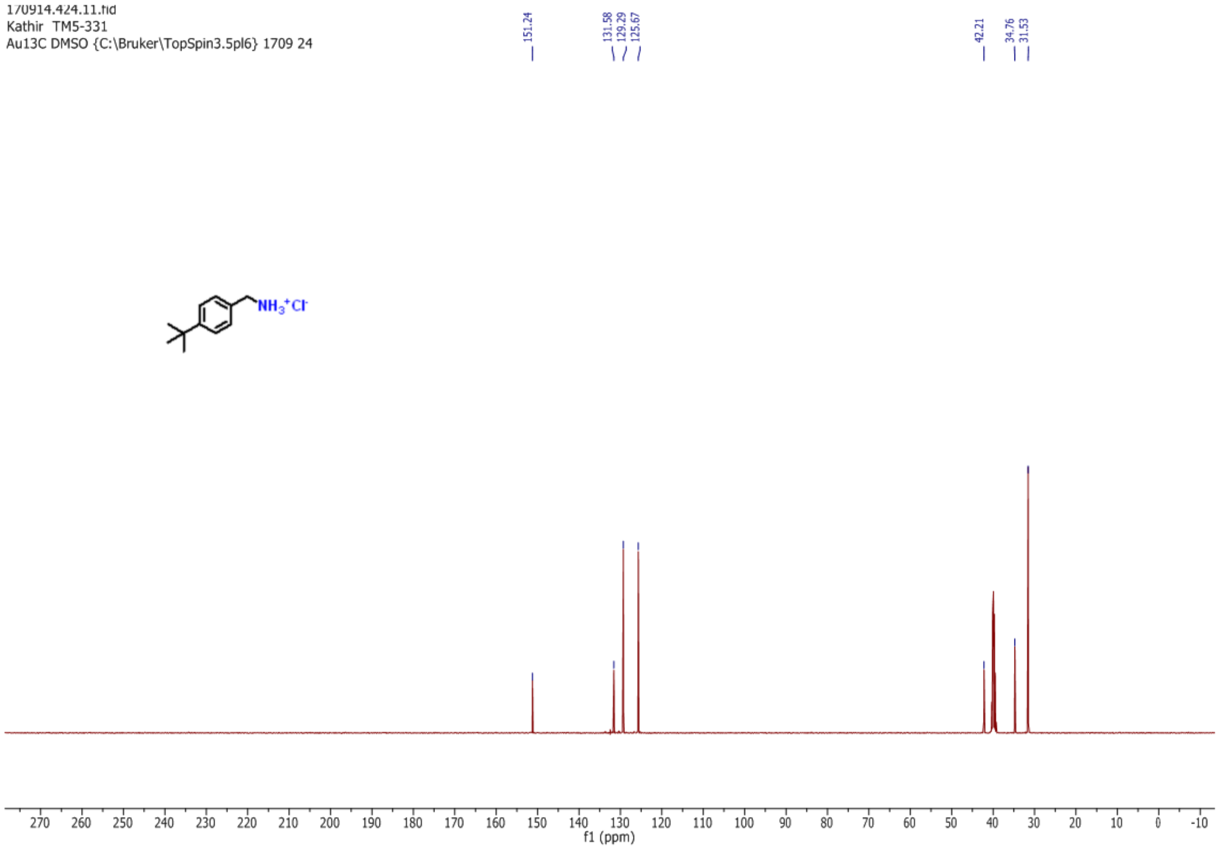

Supplementary Figure 25. ^{13}C NMR spectrum

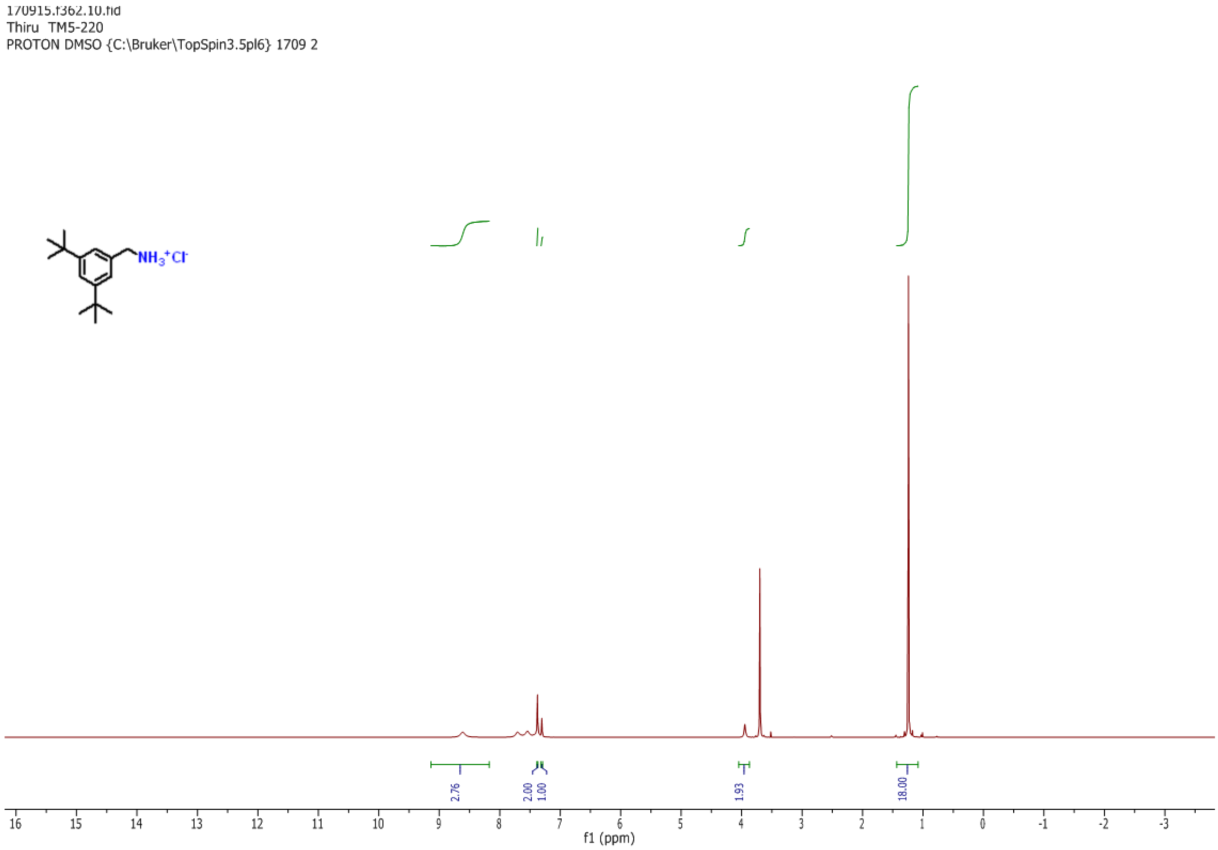

Supplementary Figure 26. ^{1}H NMR spectrum

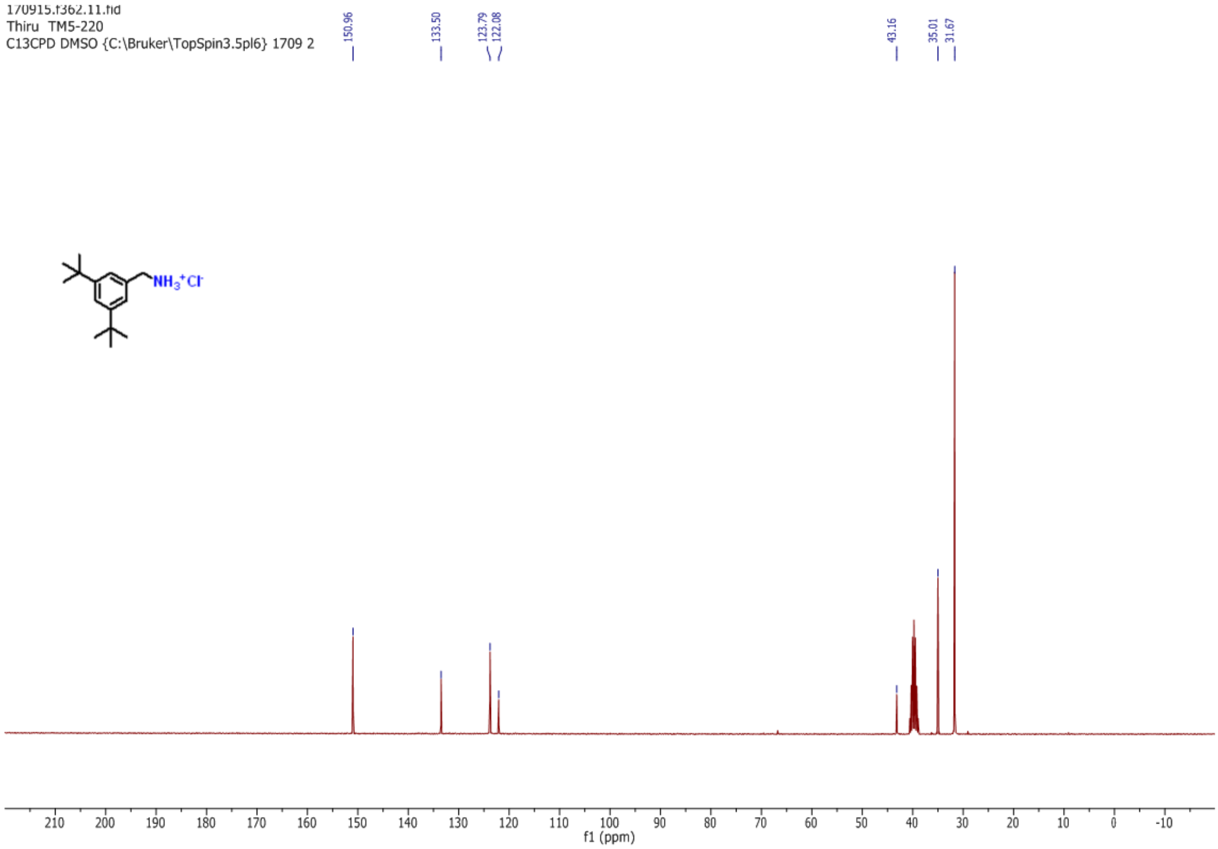

Supplementary Figure 27. ^{13}C NMR spectrum

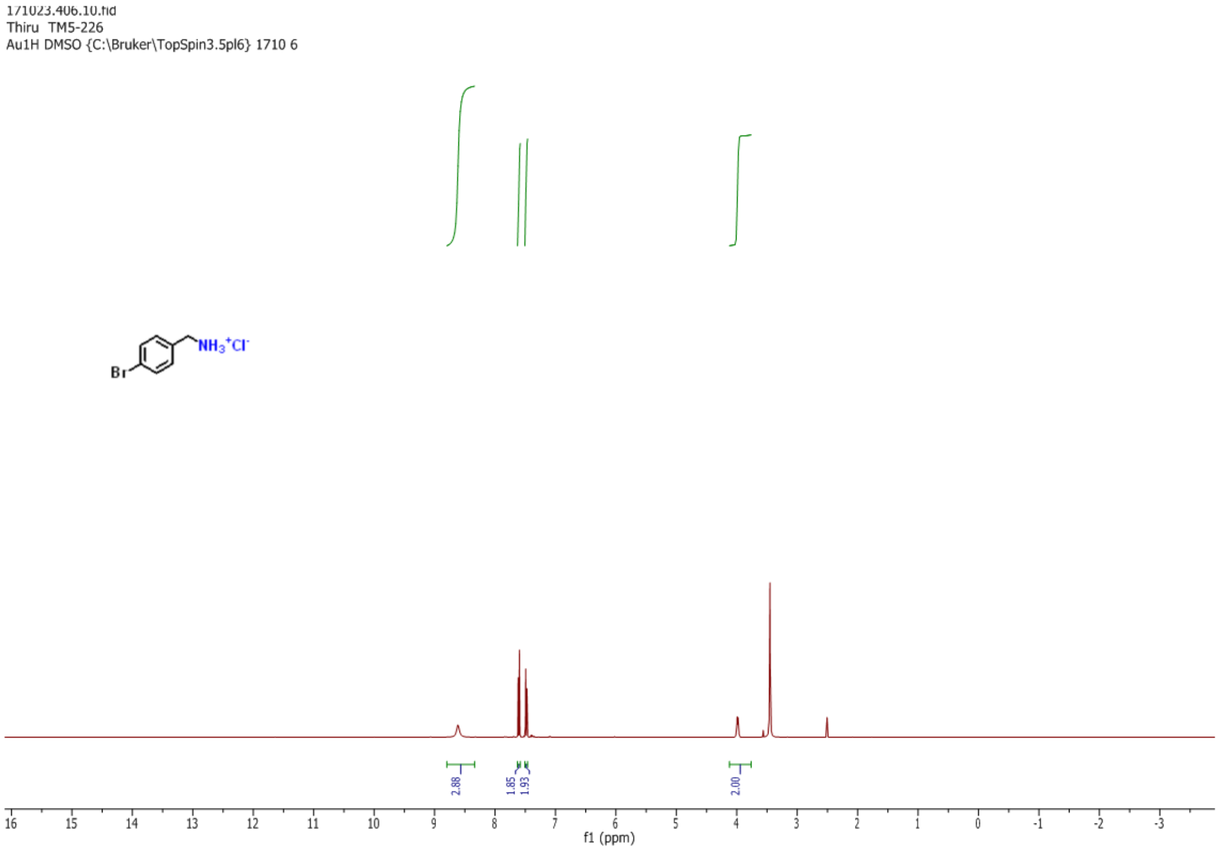

Supplementary Figure 28. ^{1}H NMR spectrum

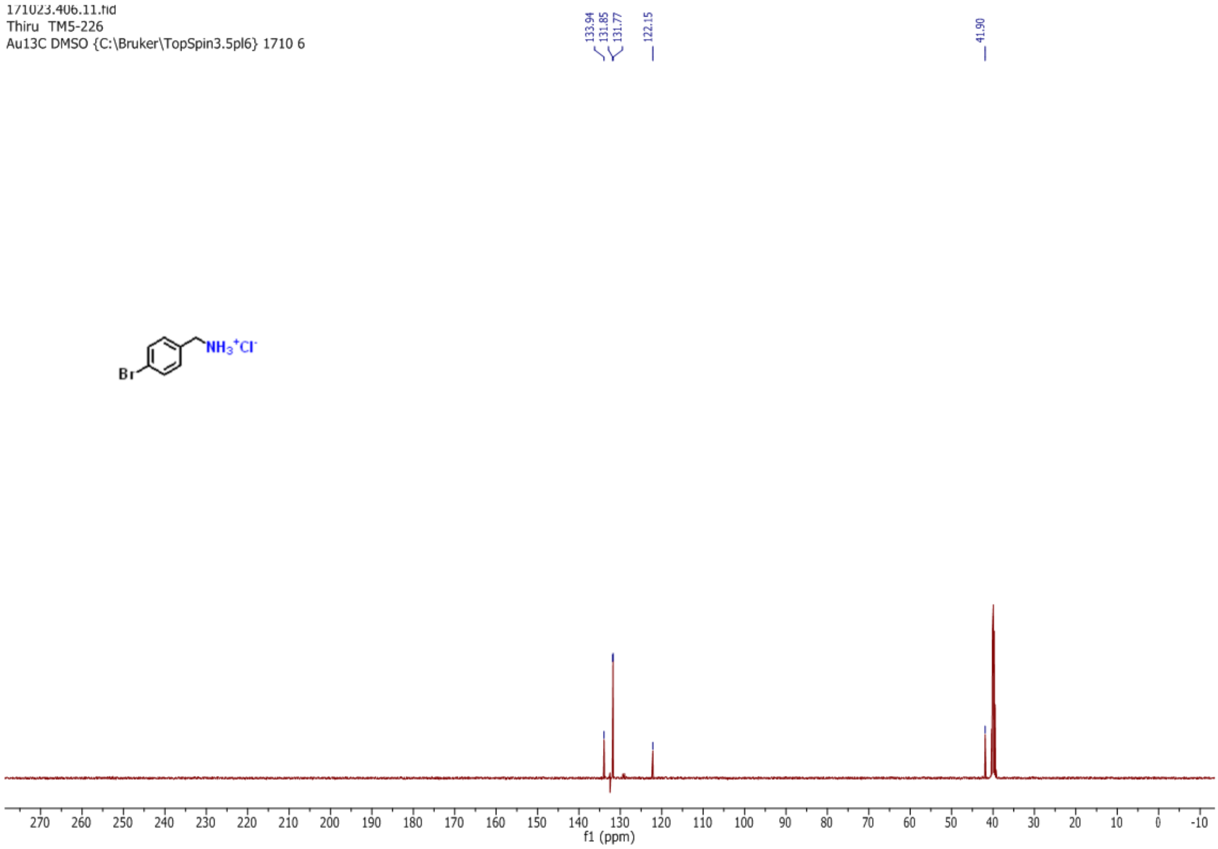

Supplementary Figure 29. ^{13}C NMR spectrum

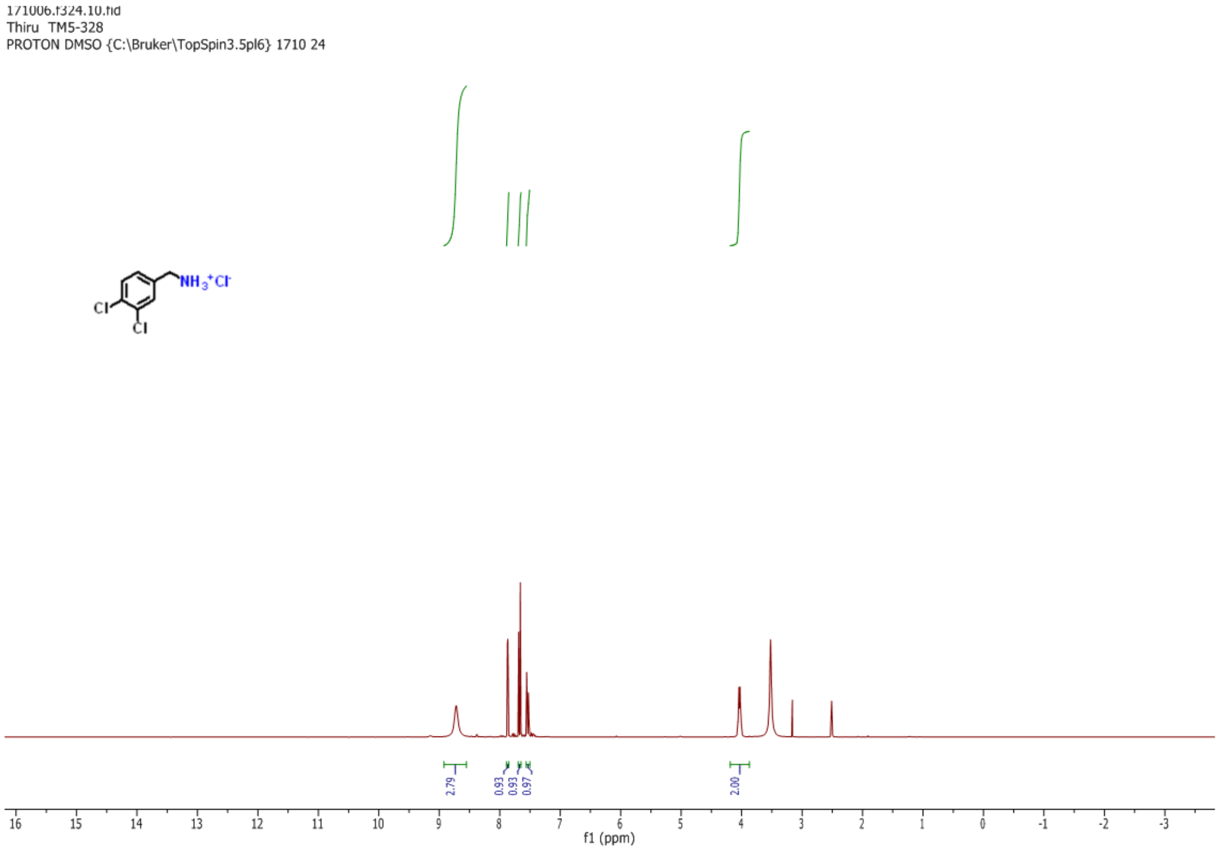

Supplementary Figure 30. ^{1}H NMR spectrum

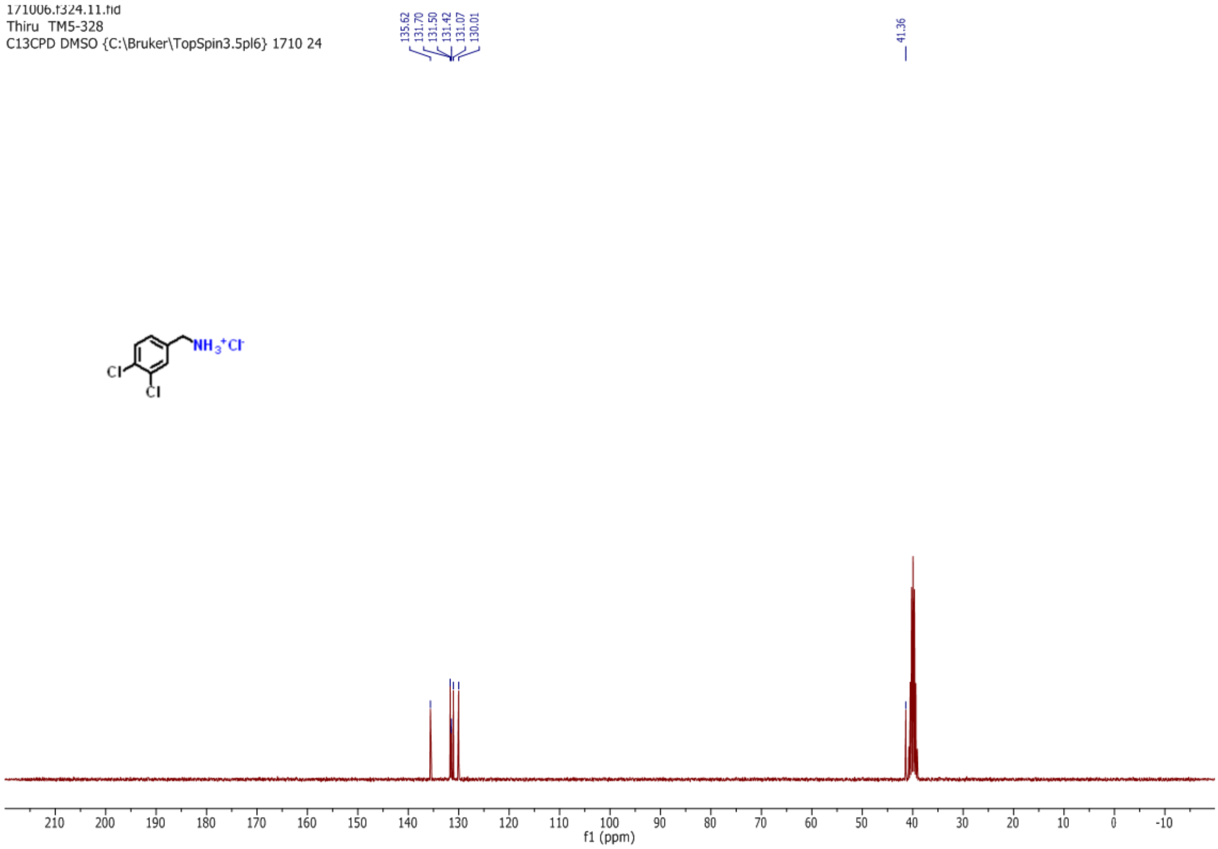

Supplementary Figure 31. ^{13}C NMR spectrum

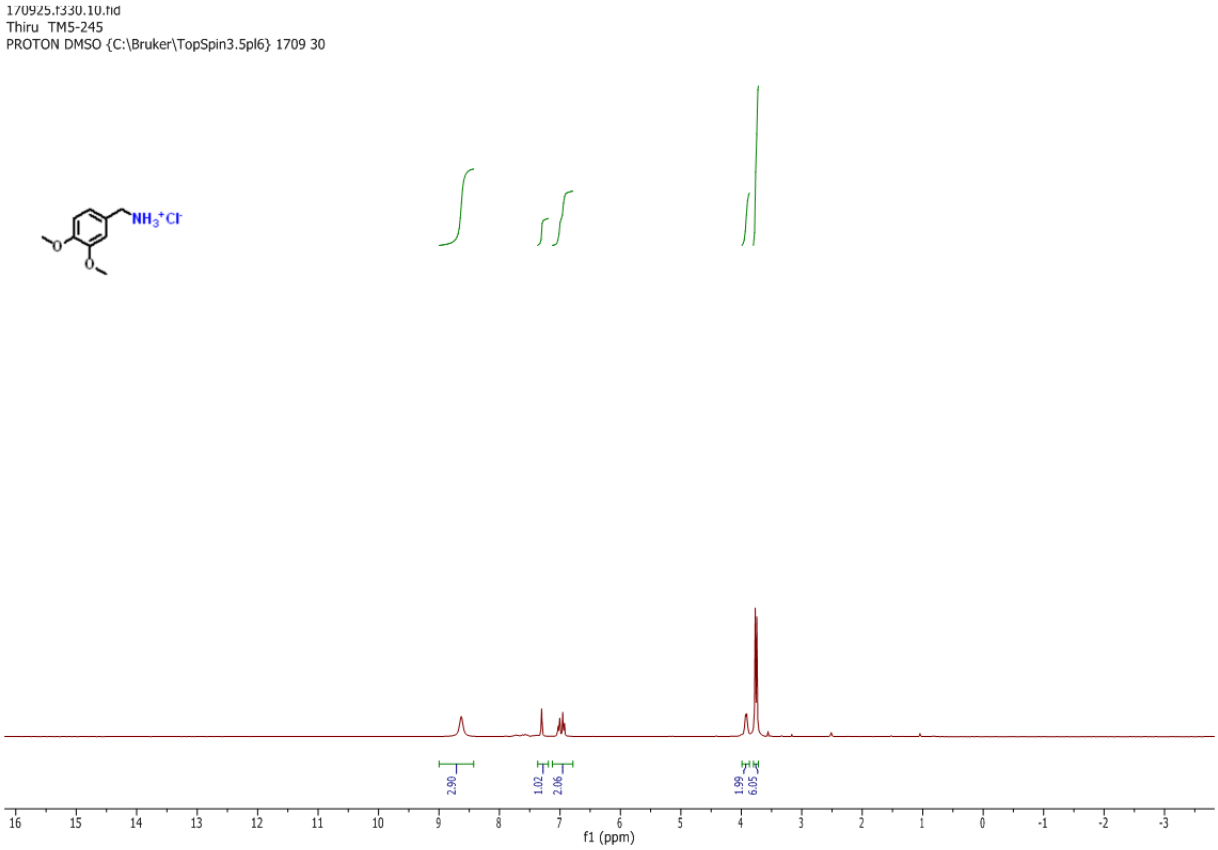

Supplementary Figure 32. ^{1}H NMR spectrum

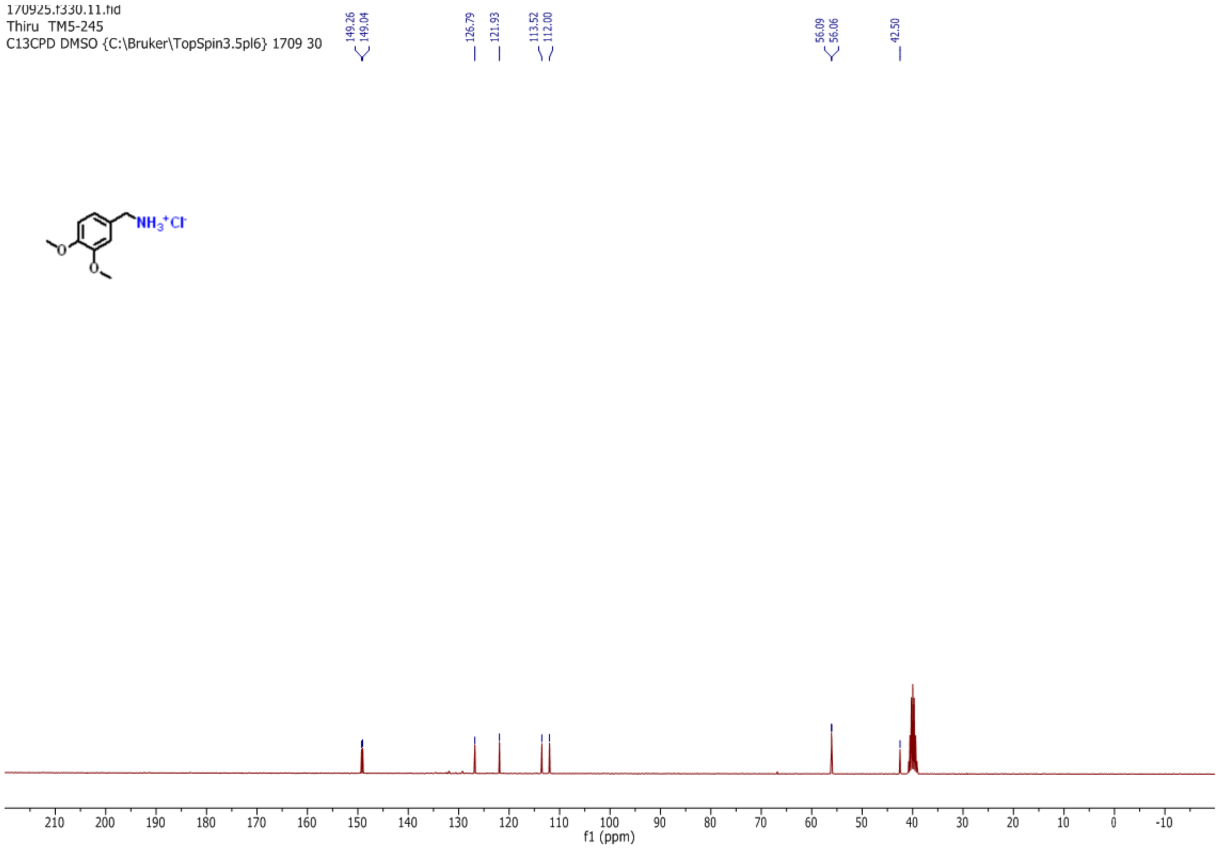

Supplementary Figure 33. ^{13}C NMR spectrum

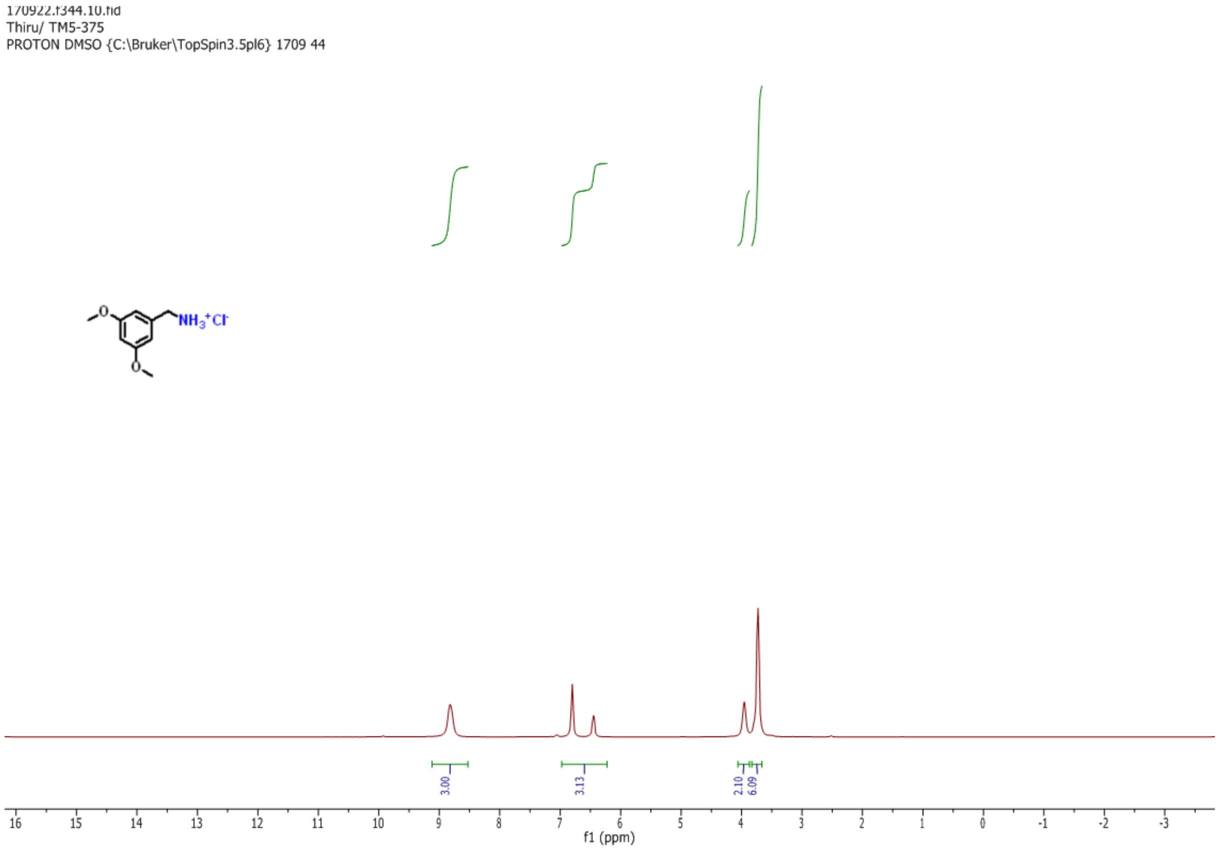

Supplementary Figure 34. ^{1}H NMR spectrum

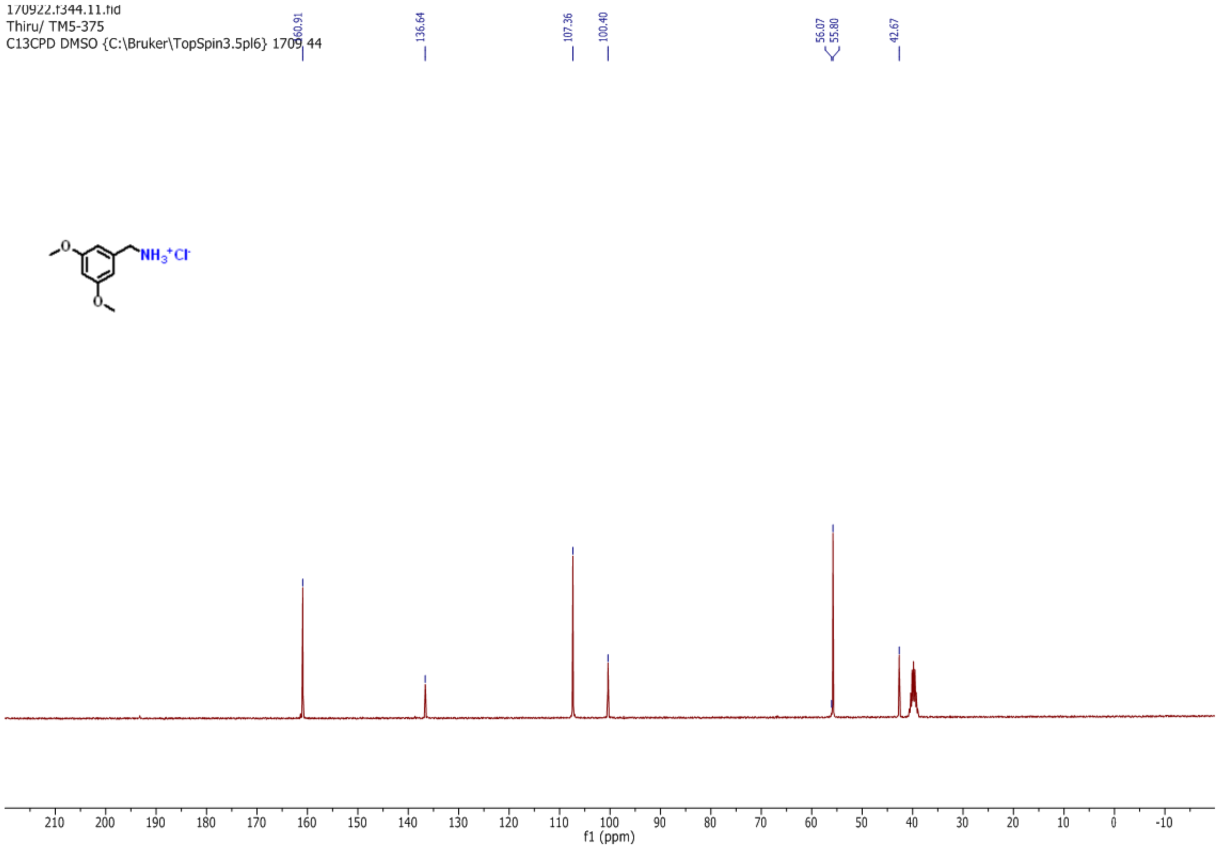

Supplementary Figure 35. ^{13}C NMR spectrum

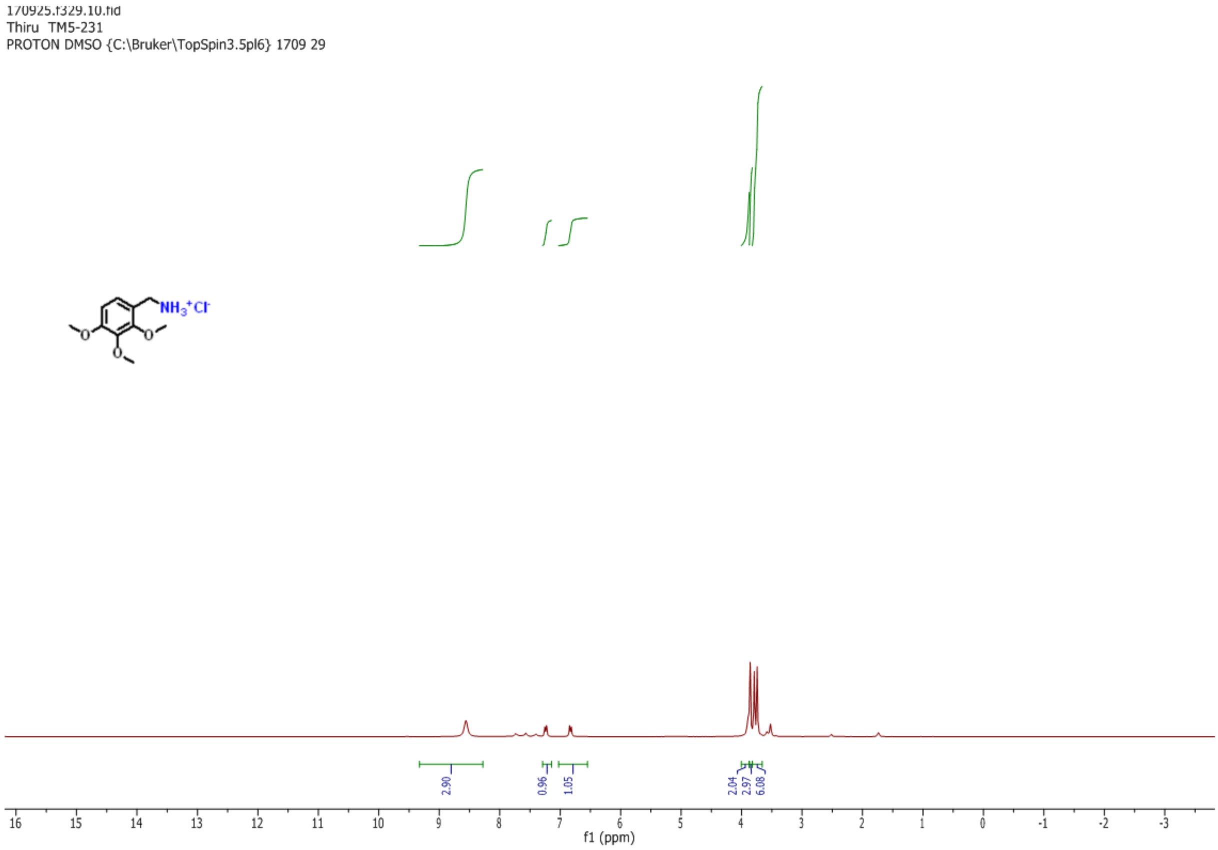

Supplementary Figure 36. ^{1}H NMR spectrum

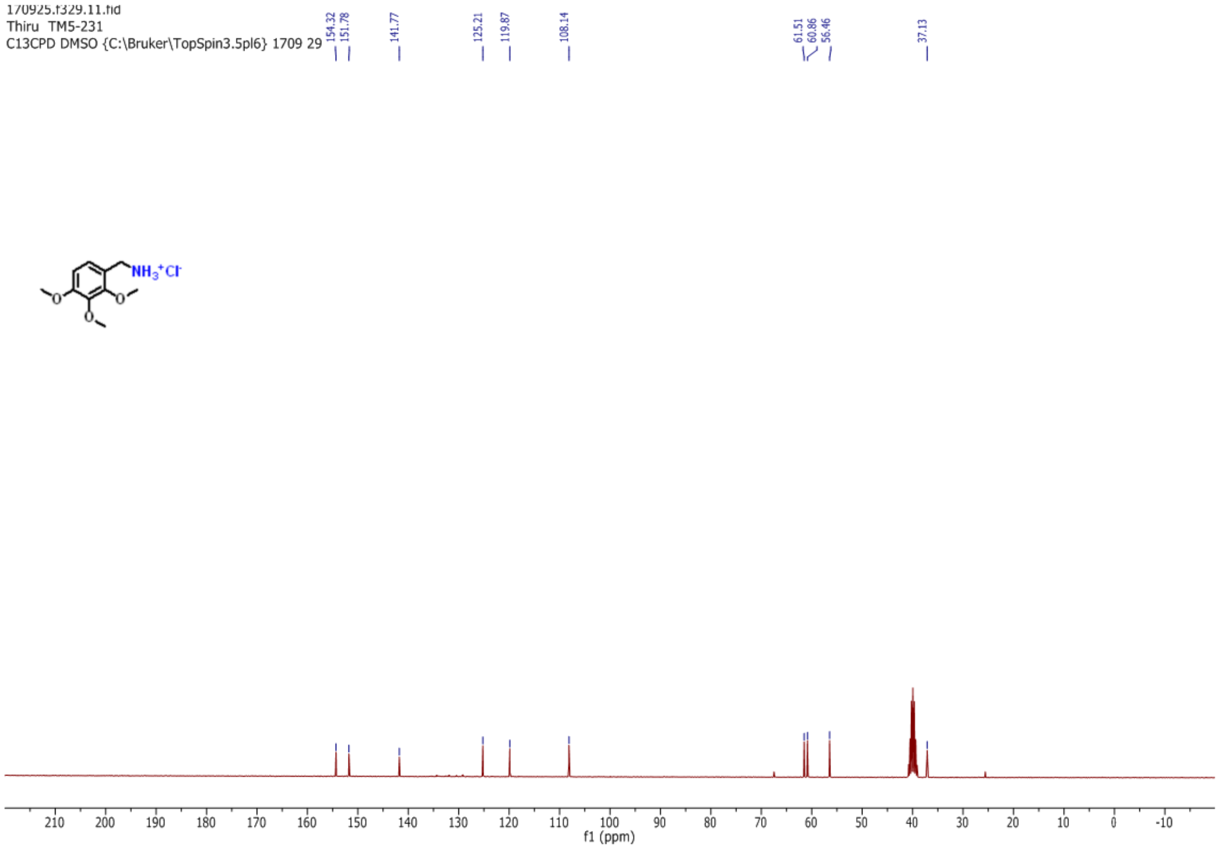

Supplementary Figure 37. ^{13}C NMR spectrum

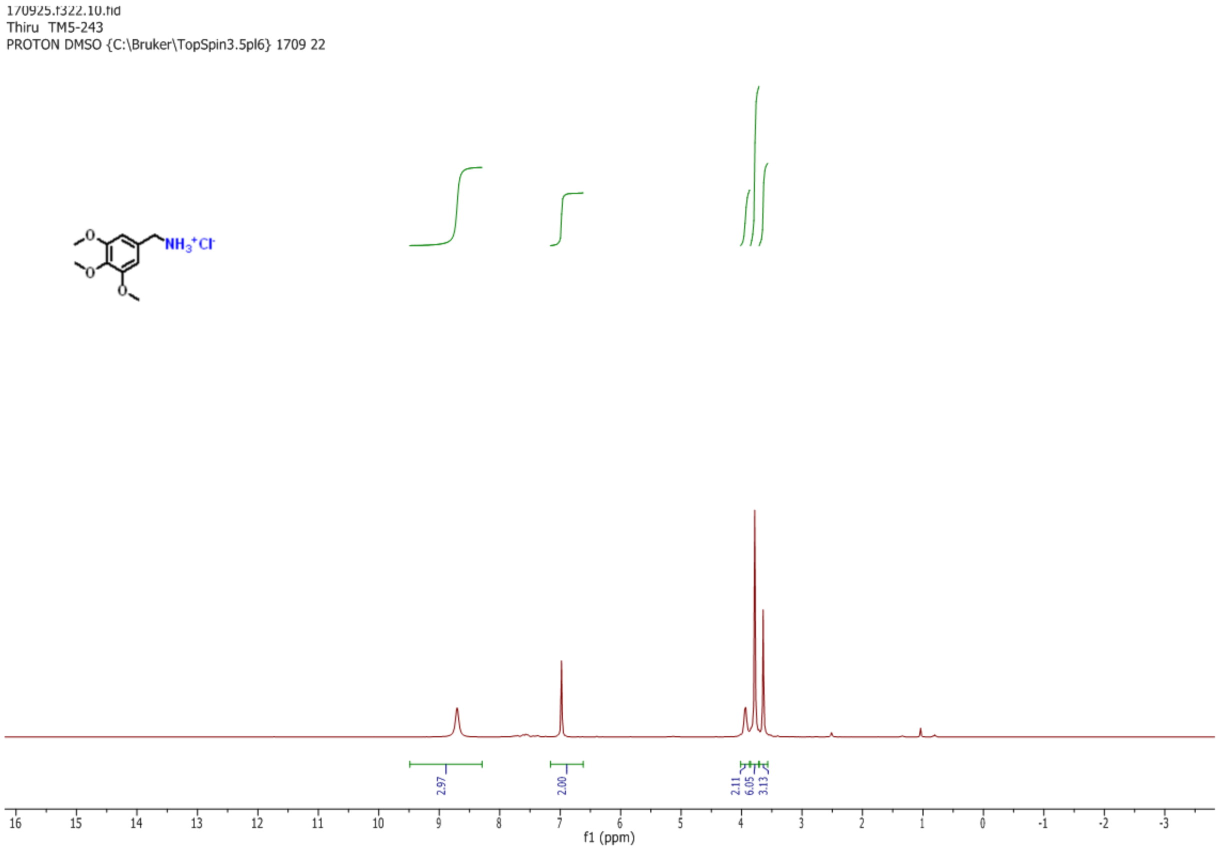

Supplementary Figure 38. ^{1}H NMR spectrum

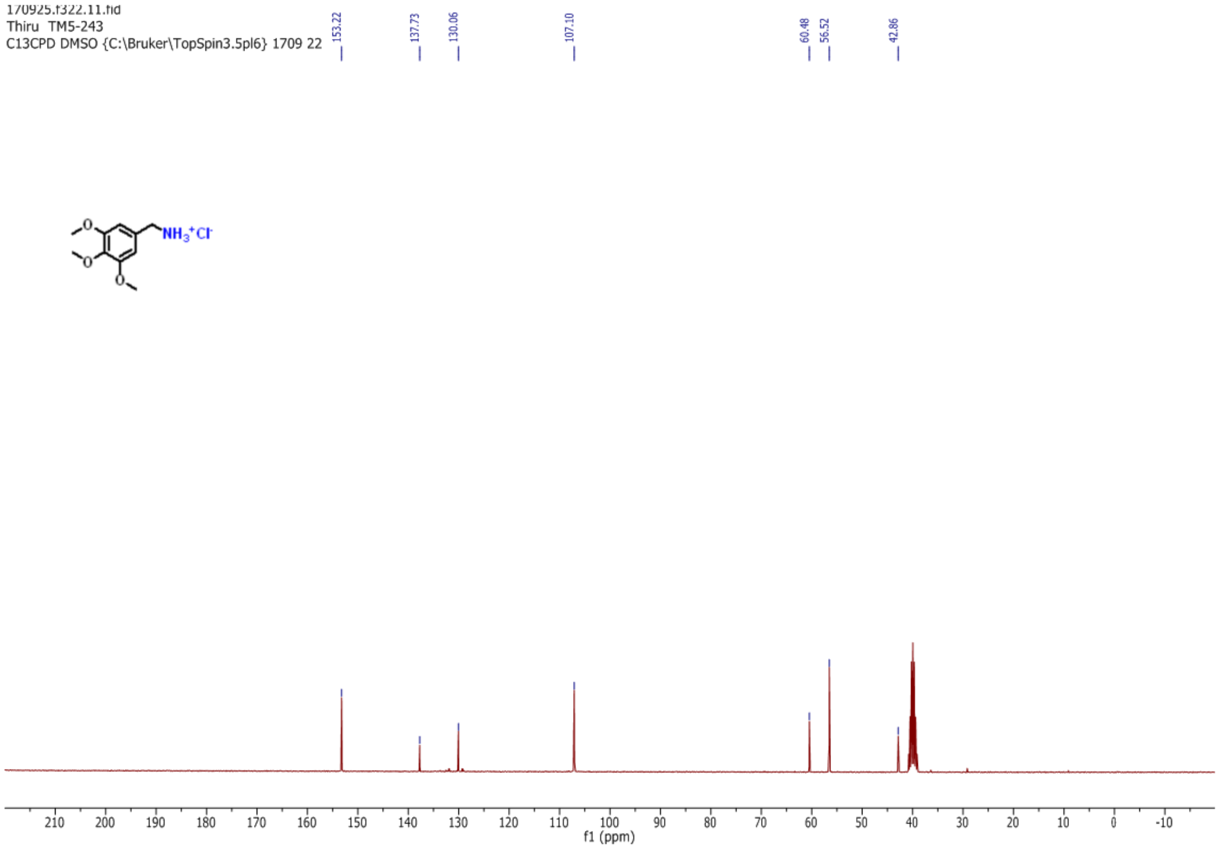

Supplementary Figure 39. ^{13}C NMR spectrum

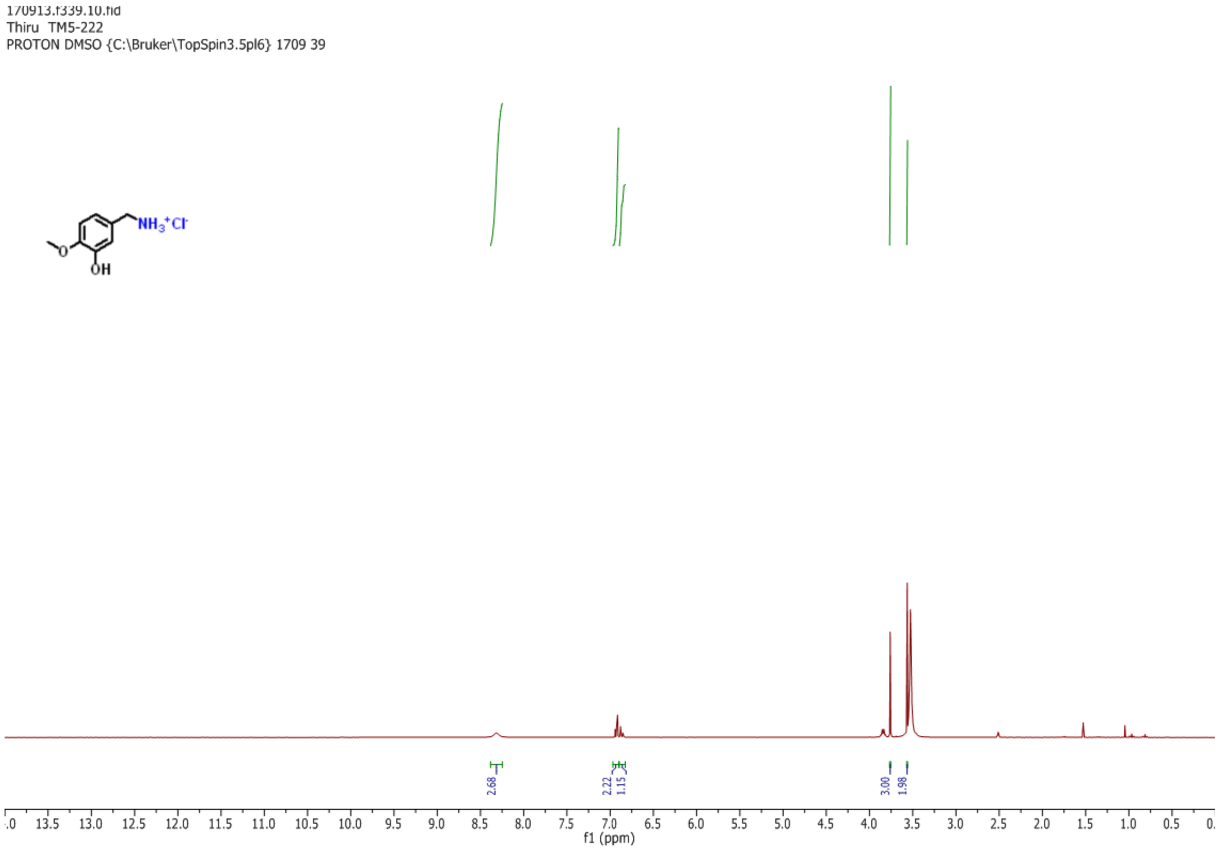

Supplementary Figure 40. ^{1}H NMR spectrum

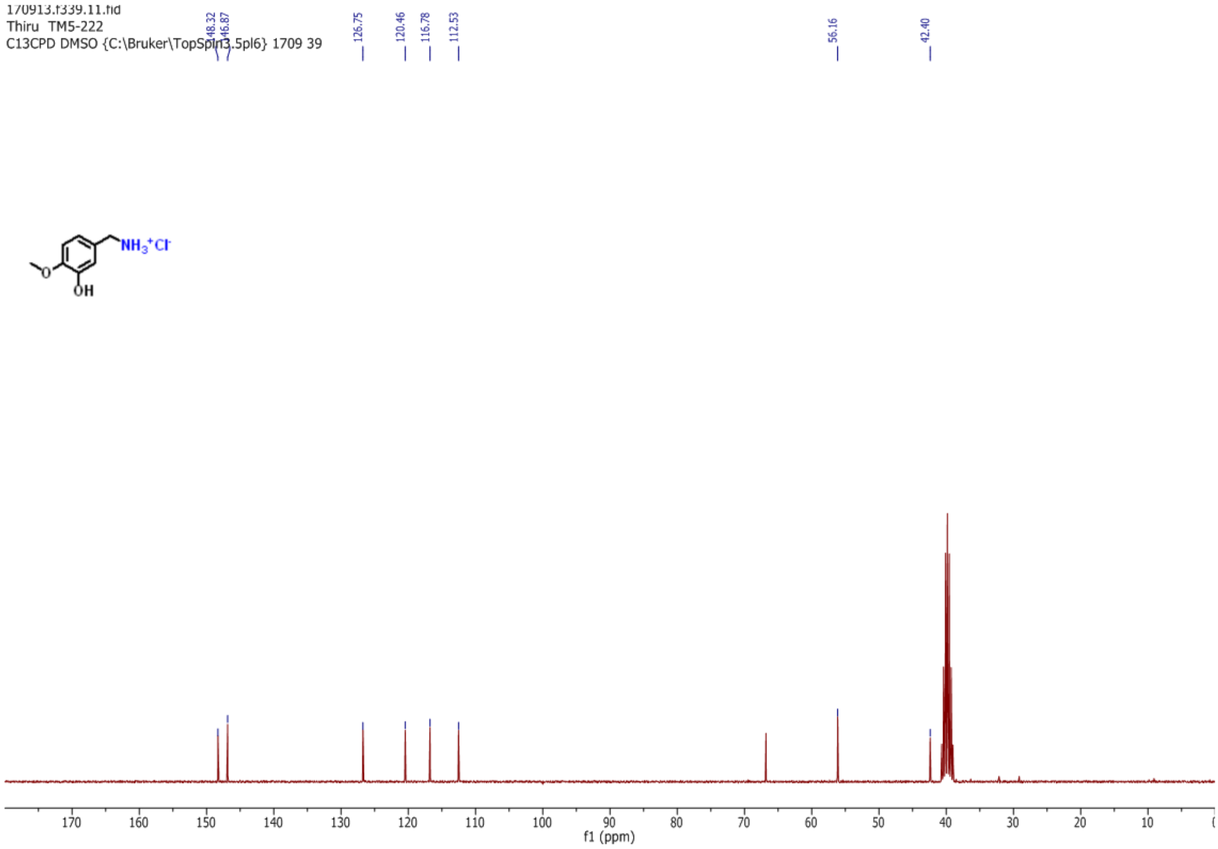

Supplementary Figure 41. ^{13}C NMR spectrum

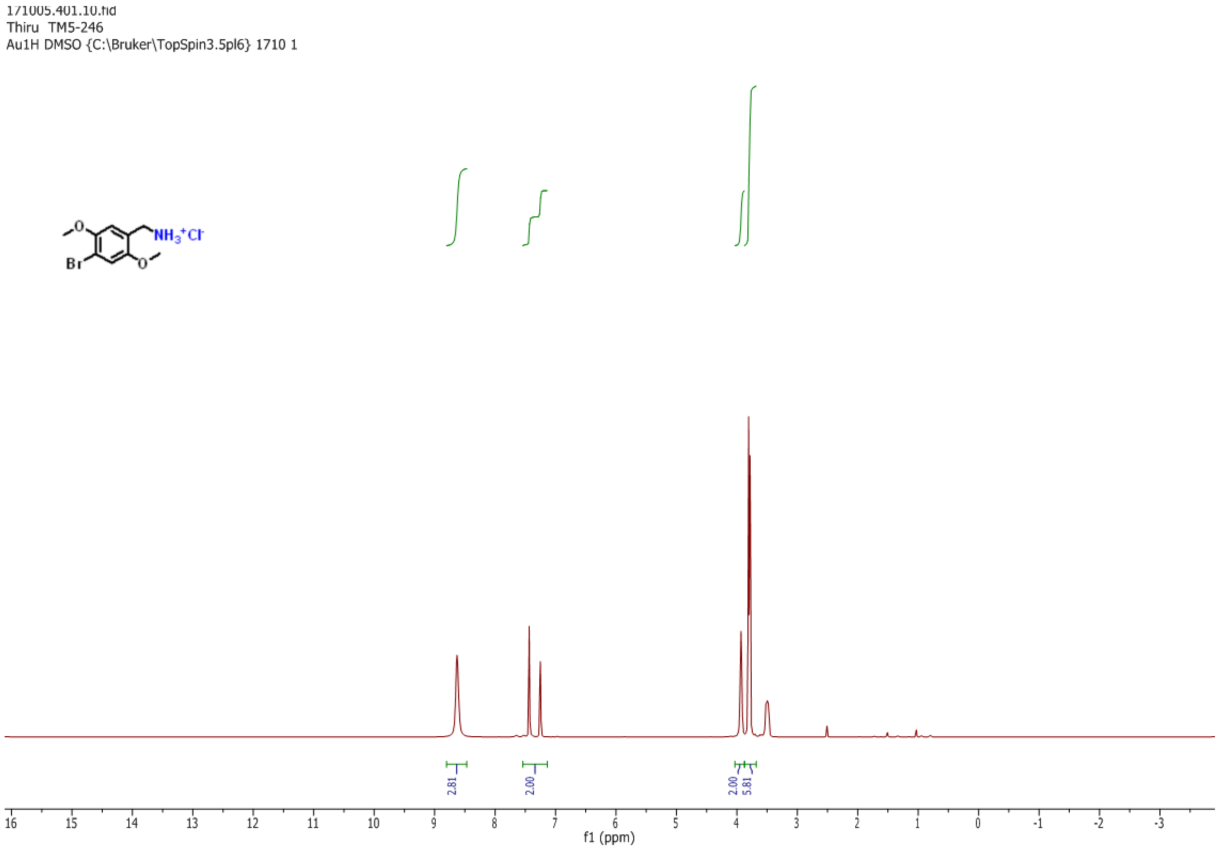

Supplementary Figure 42. ^{1}H NMR spectrum

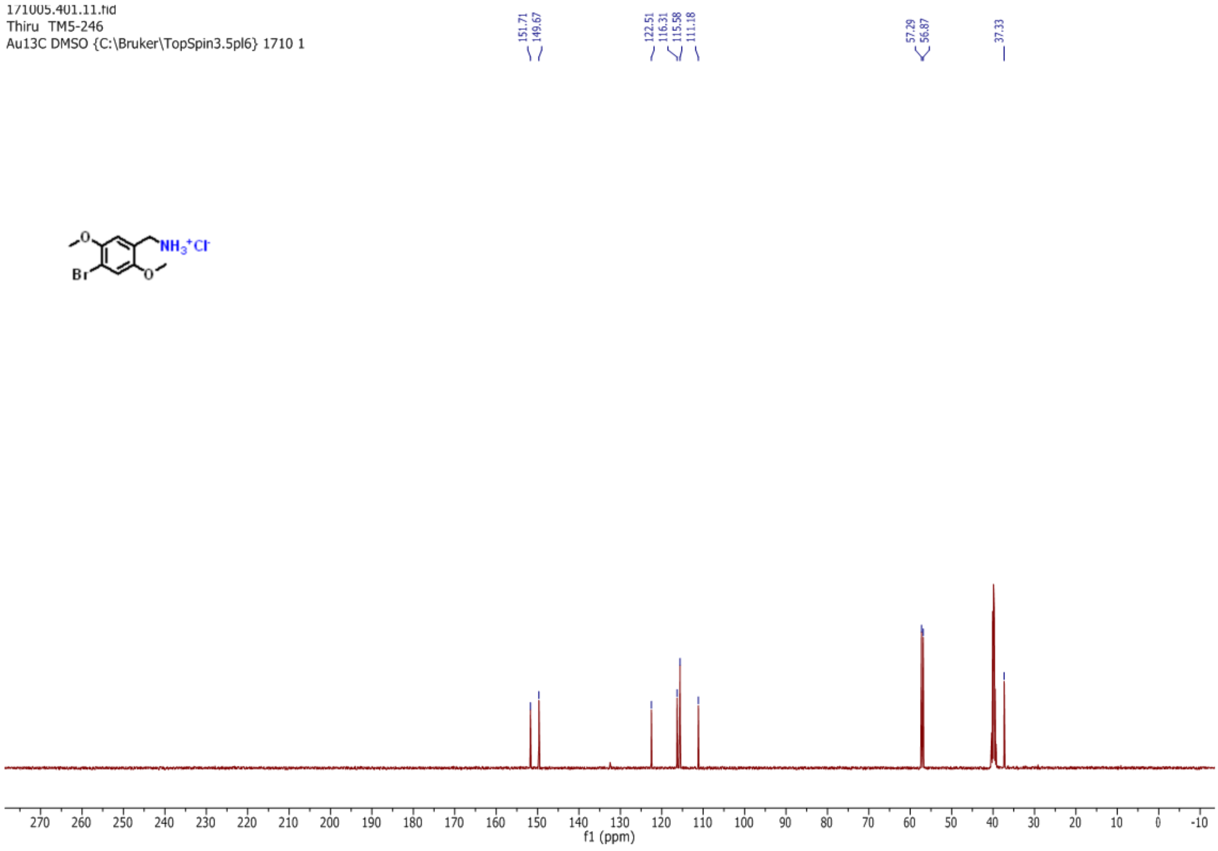

Supplementary Figure 43. ^{13}C NMR spectrum

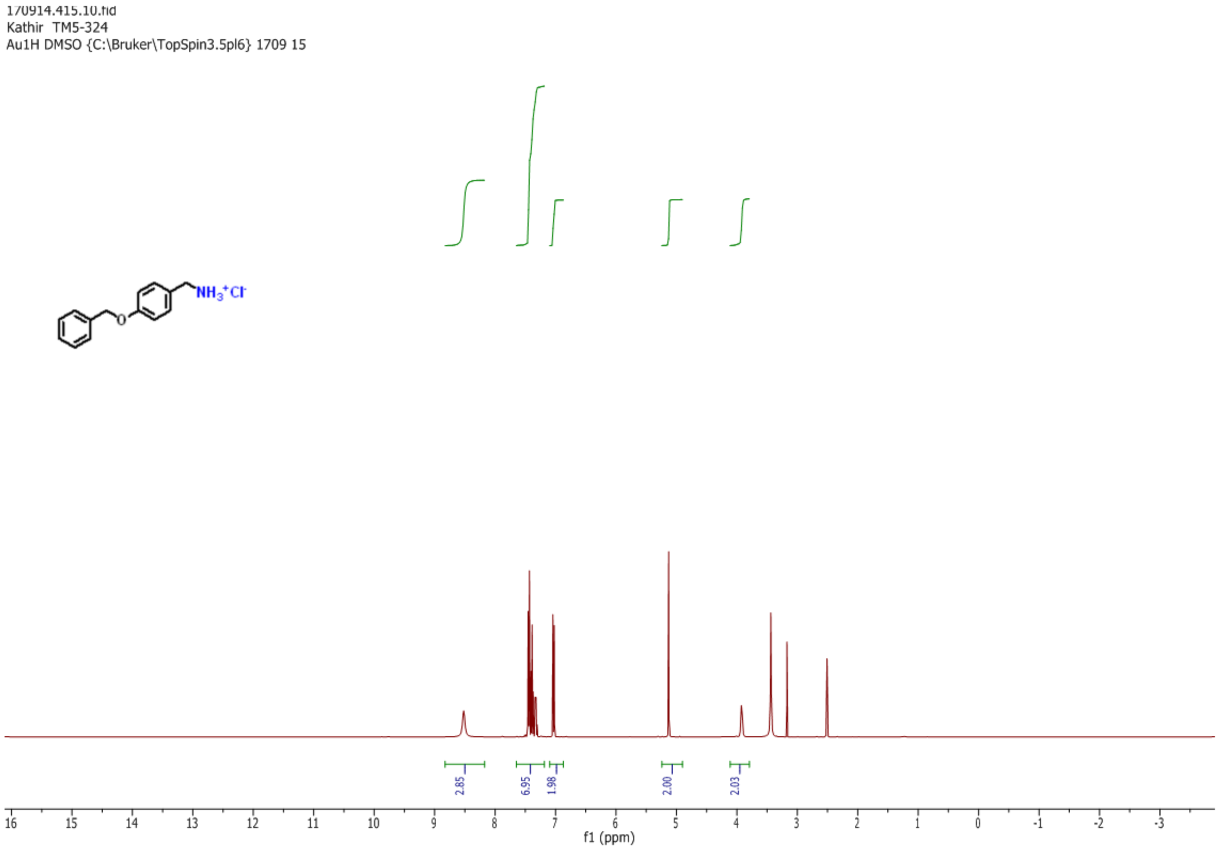

Supplementary Figure 44. ^{1}H NMR spectrum

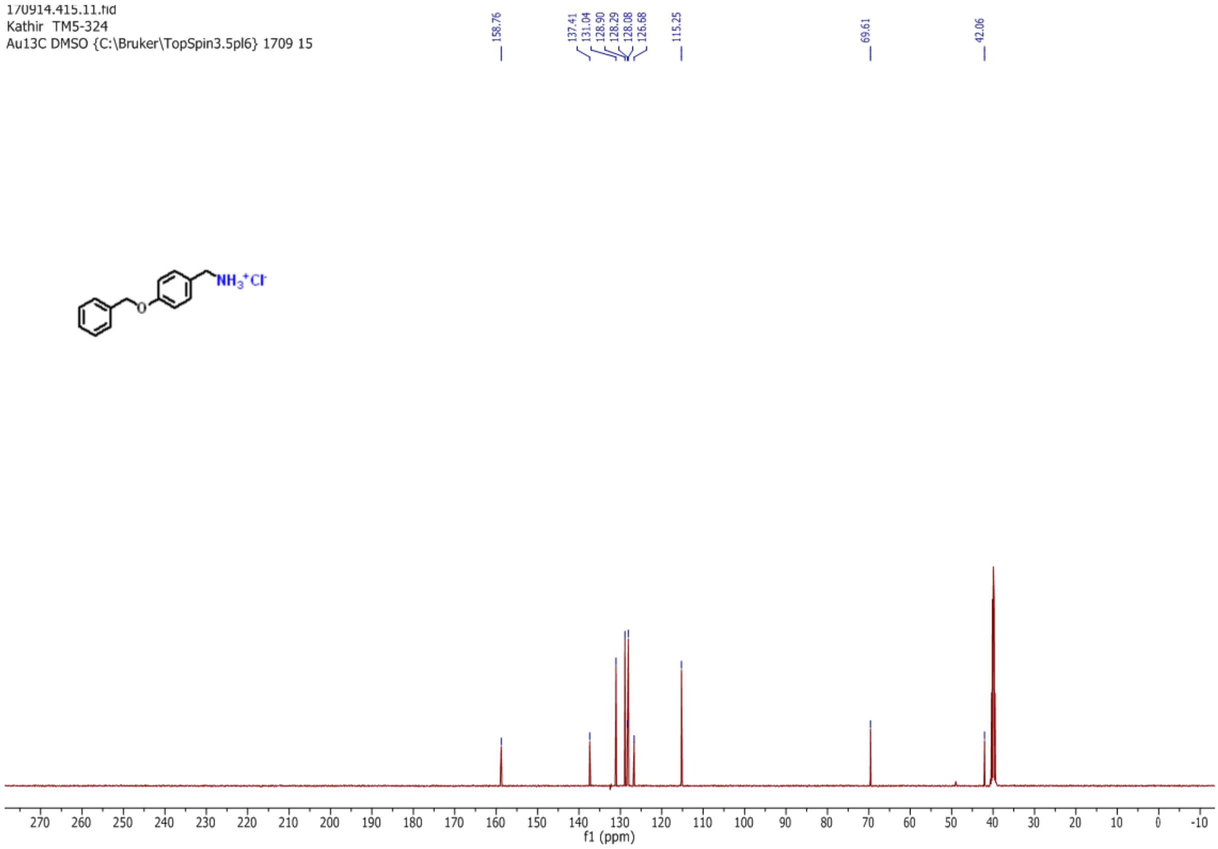

Supplementary Figure 45. ^{13}C NMR spectrum

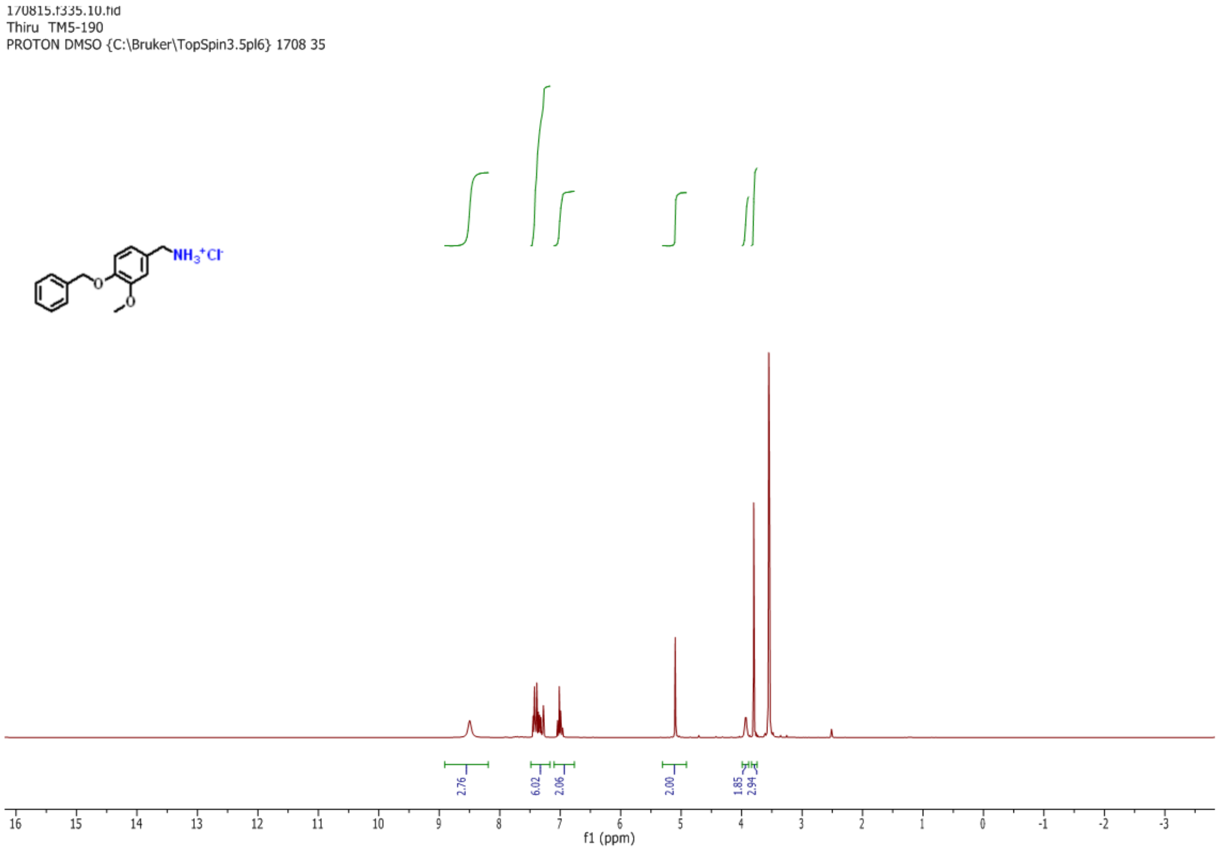

Supplementary Figure 46. ^{1}H NMR spectrum

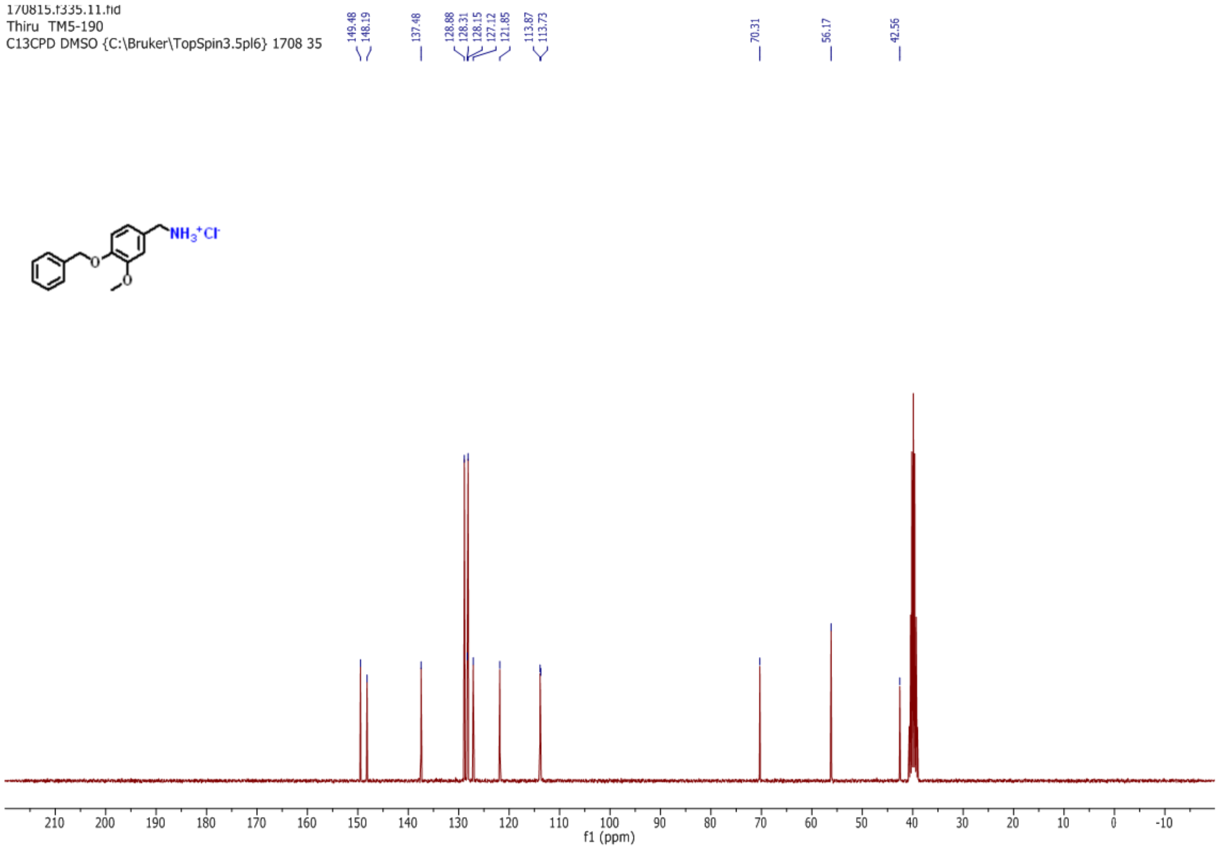

Supplementary Figure 47. ^{13}C NMR spectrum

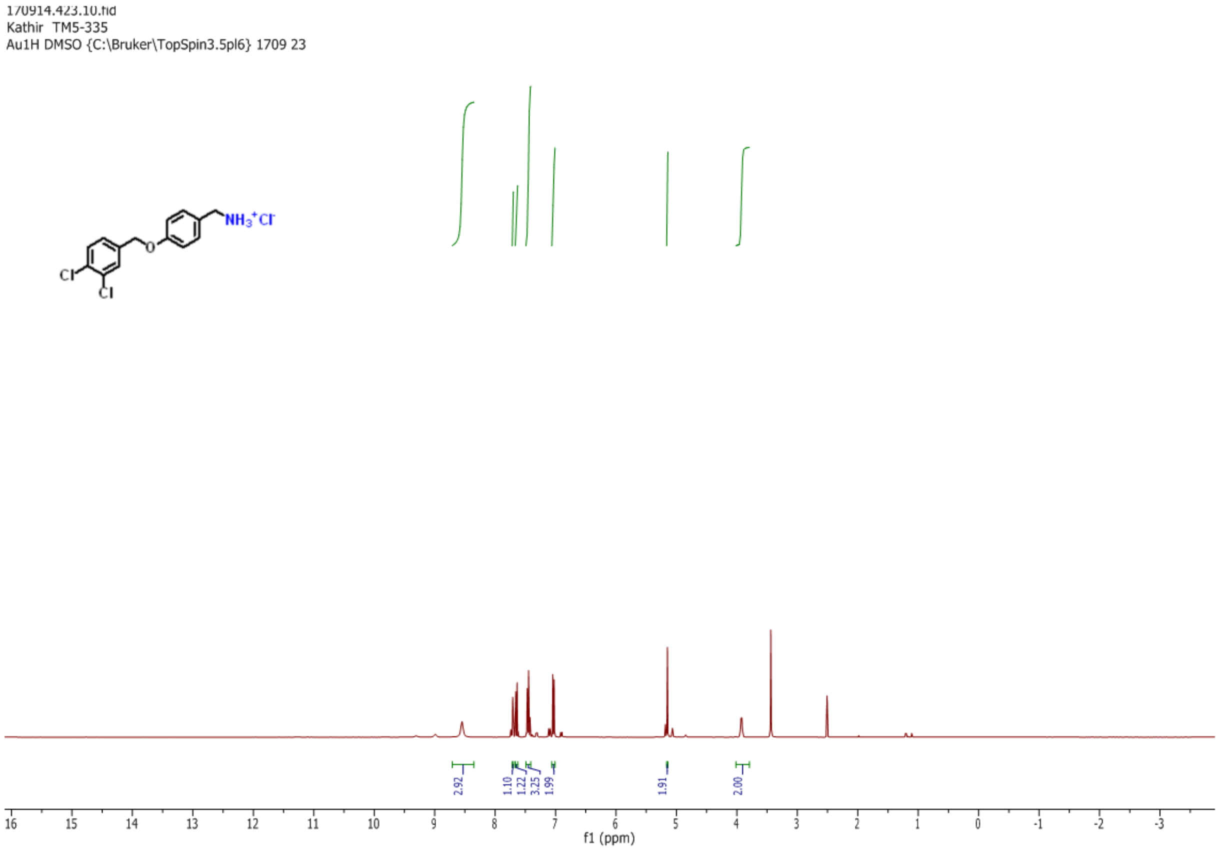

Supplementary Figure 48. ^{1}H NMR spectrum

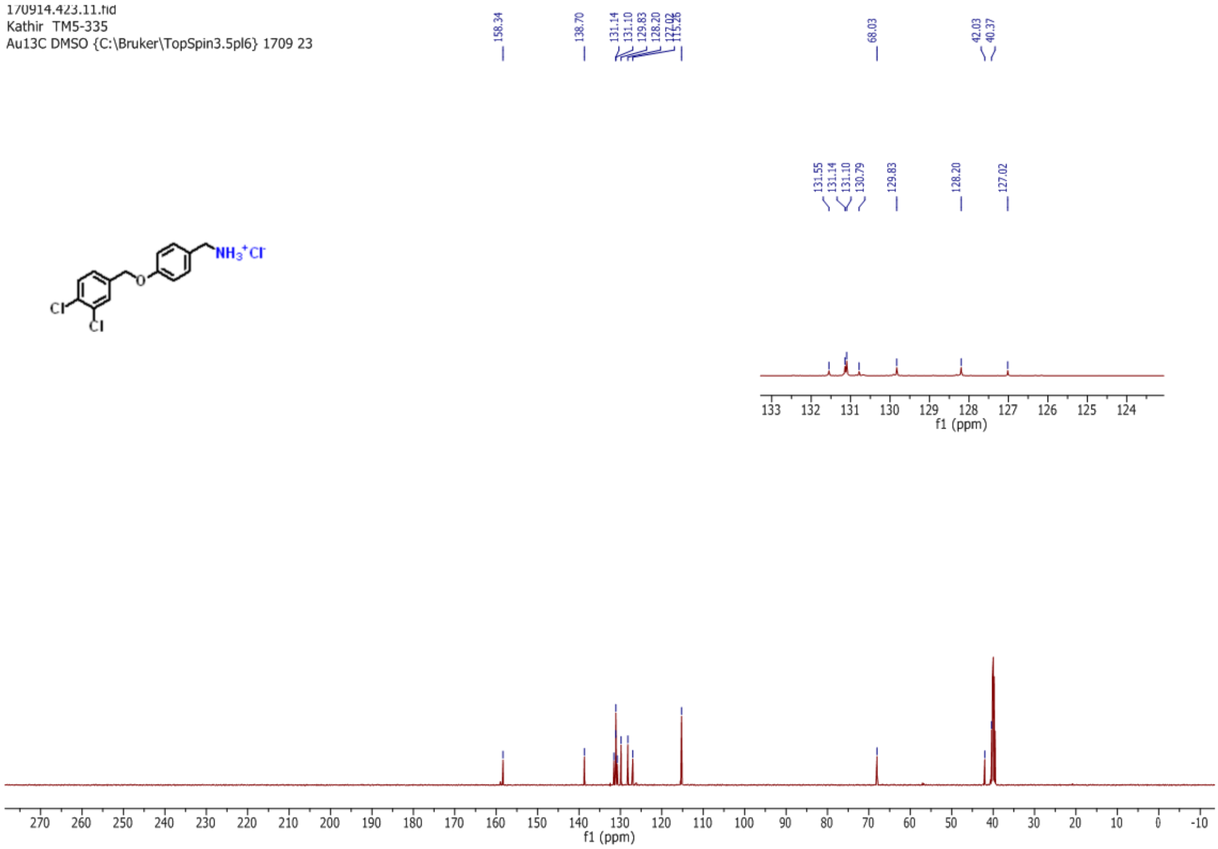

Supplementary Figure 49. ^{13}C NMR spectrum

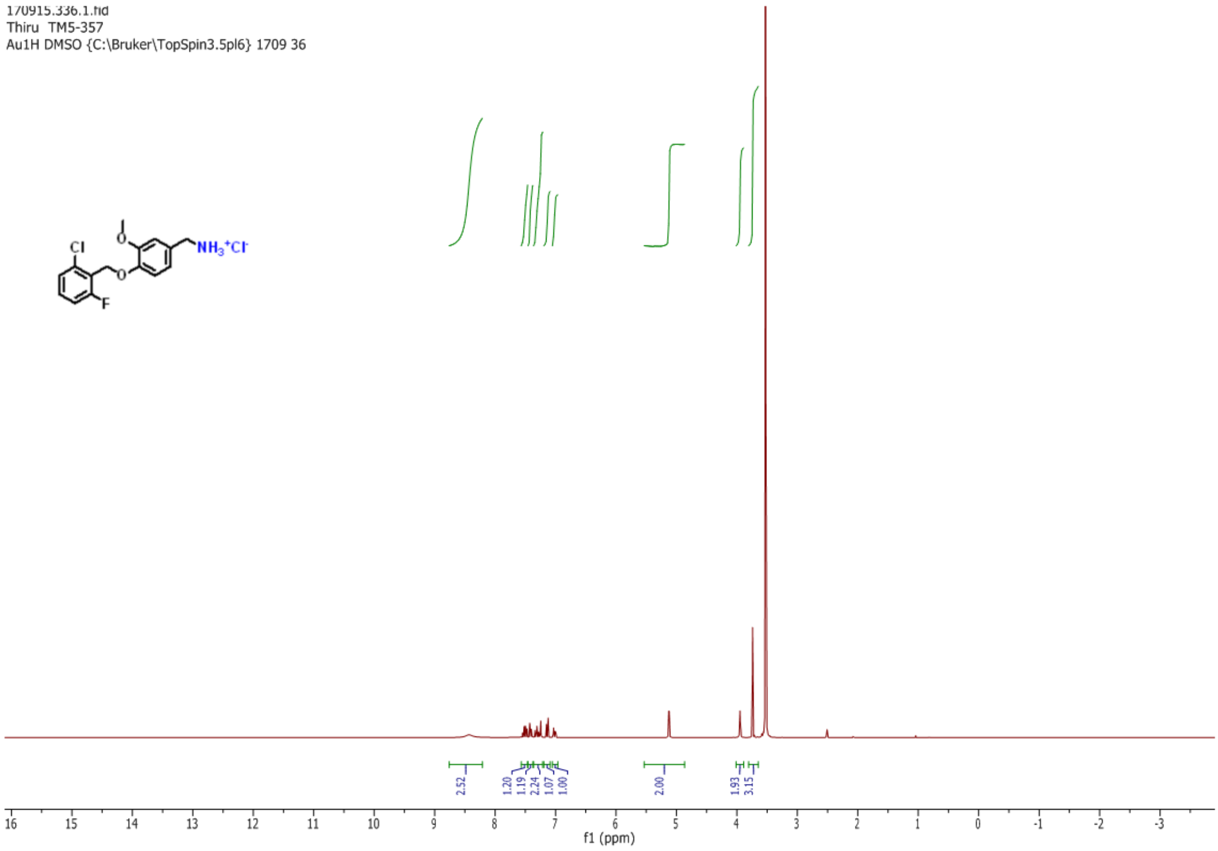

Supplementary Figure 50. ^{1}H NMR spectrum

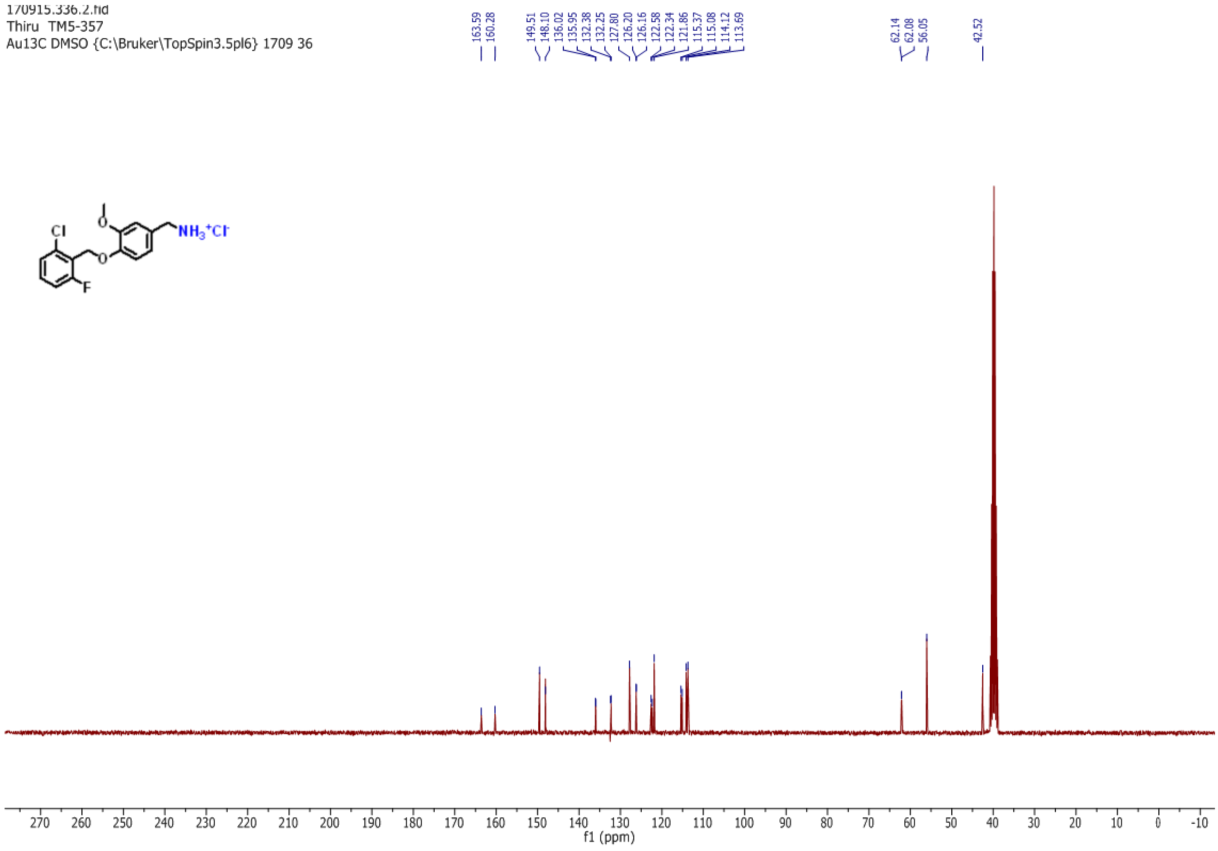

Supplementary Figure 51. ^{13}C NMR spectrum

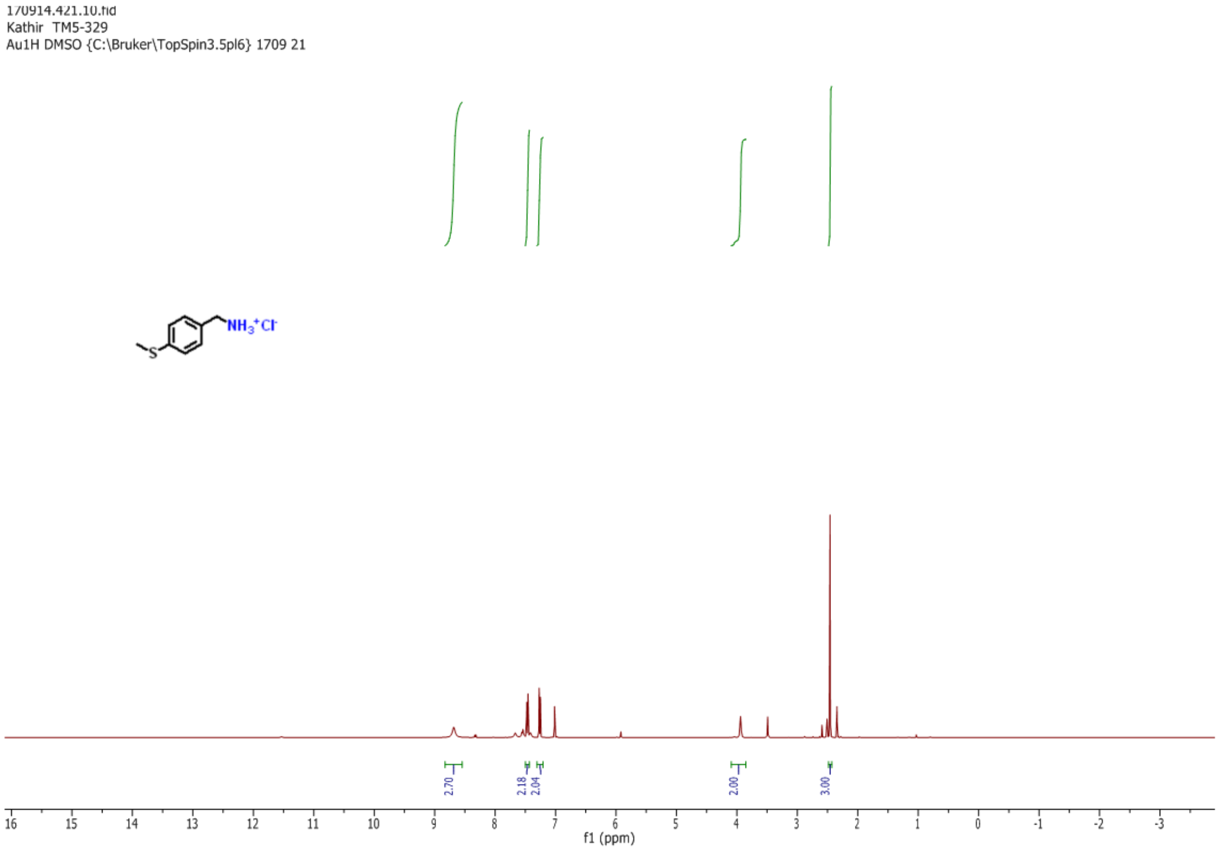

Supplementary Figure 52. ^{1}H NMR spectrum

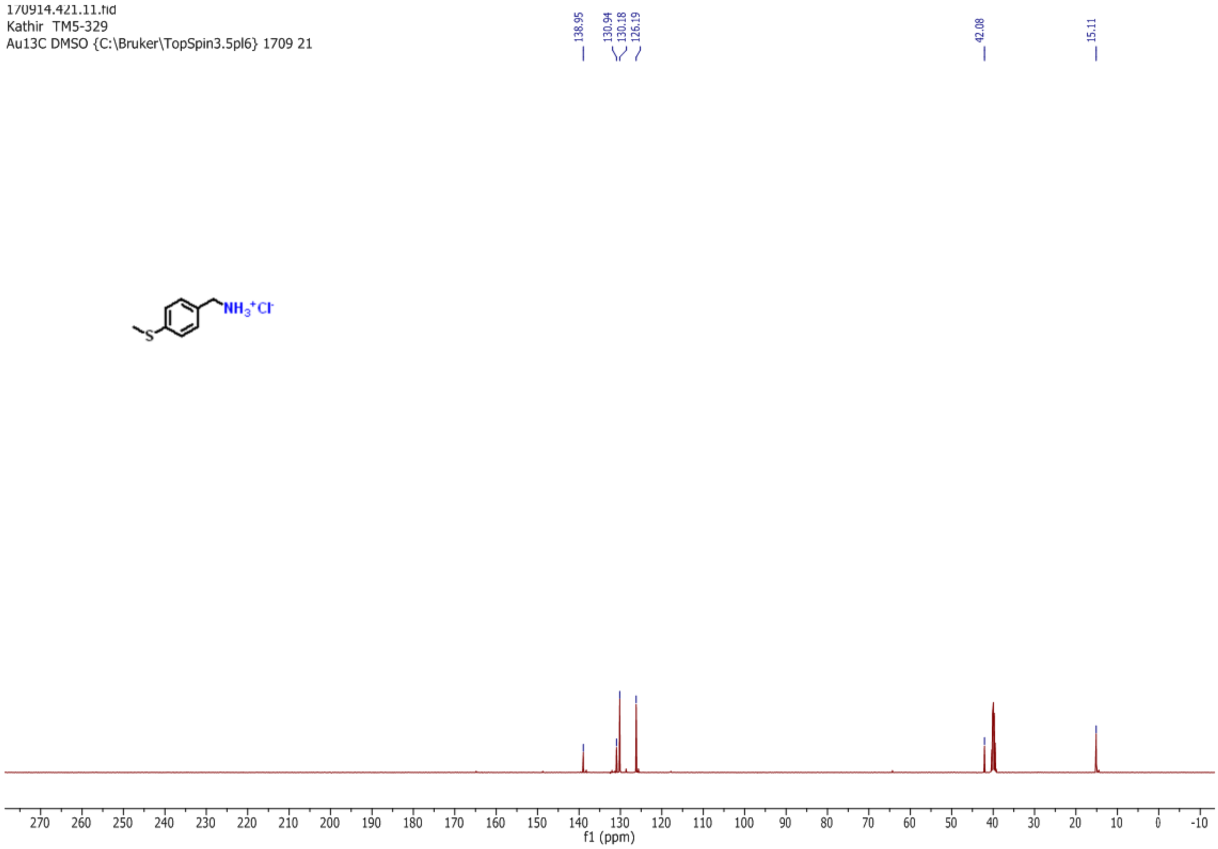

Supplementary Figure 53. ^{13}C NMR spectrum

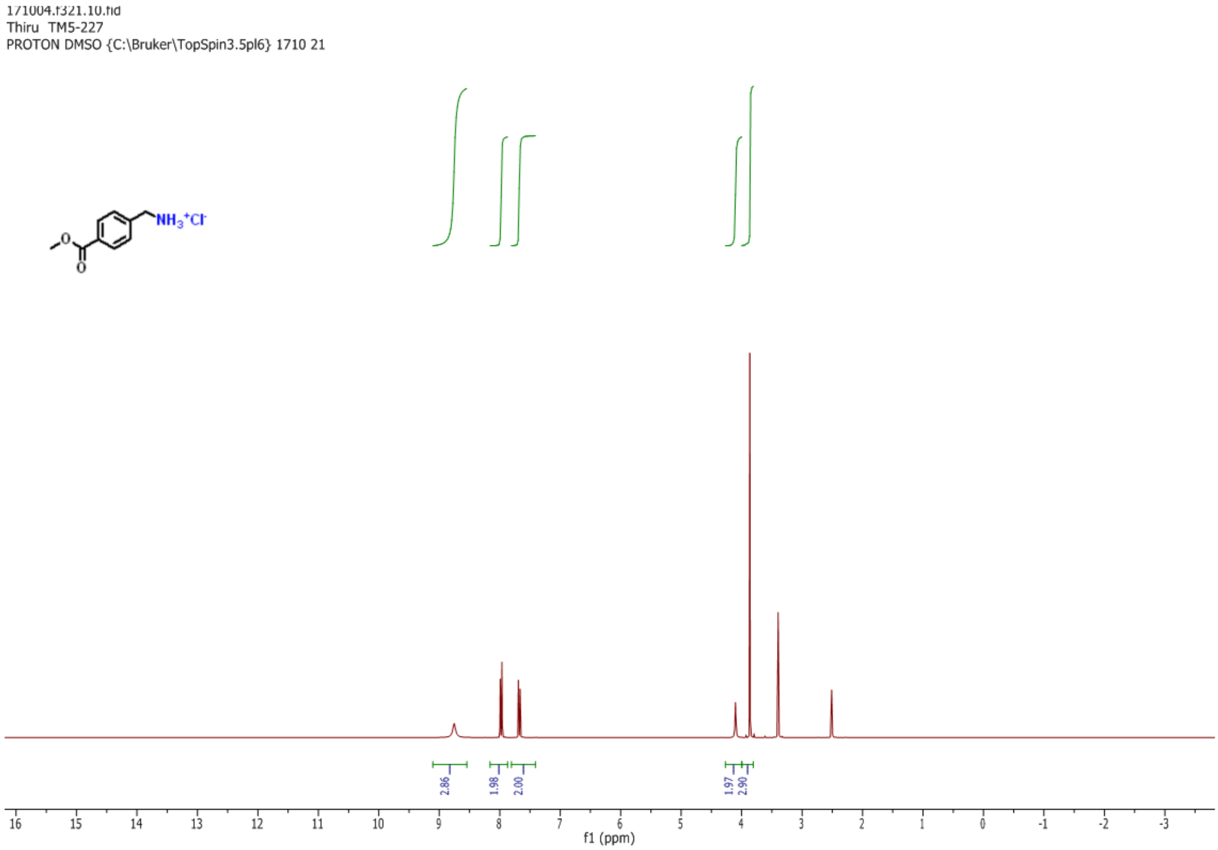

Supplementary Figure 54. ^{1}H NMR spectrum

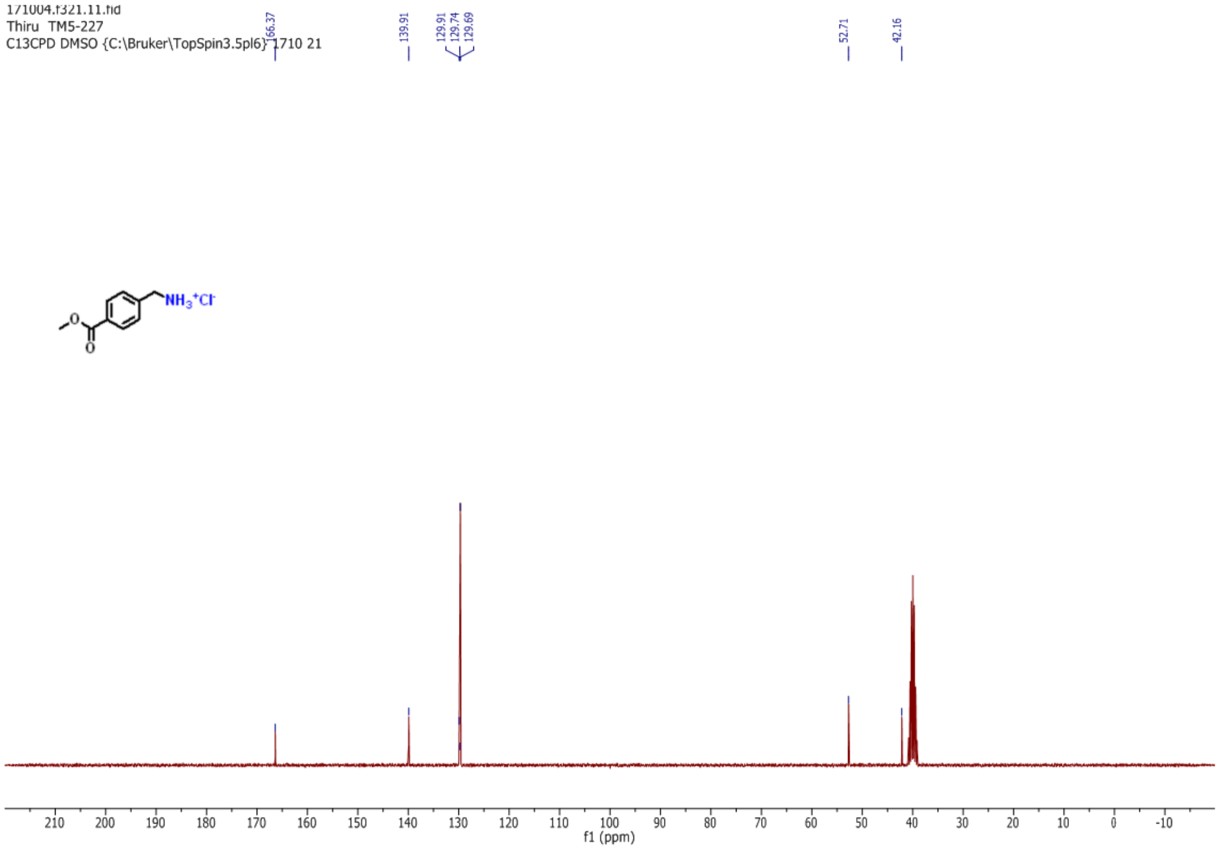

Supplementary Figure 55. ^{13}C NMR spectrum

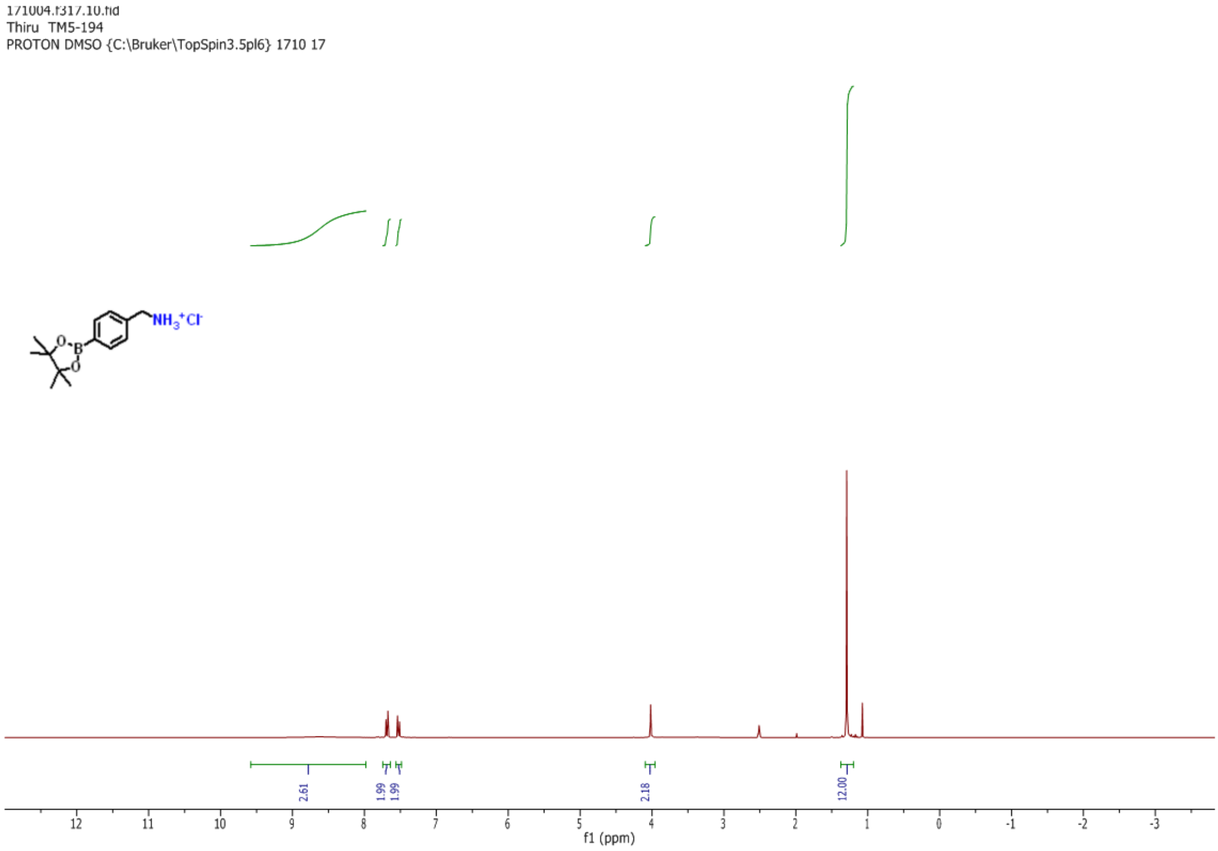

Supplementary Figure 56. ^{1}H NMR spectrum

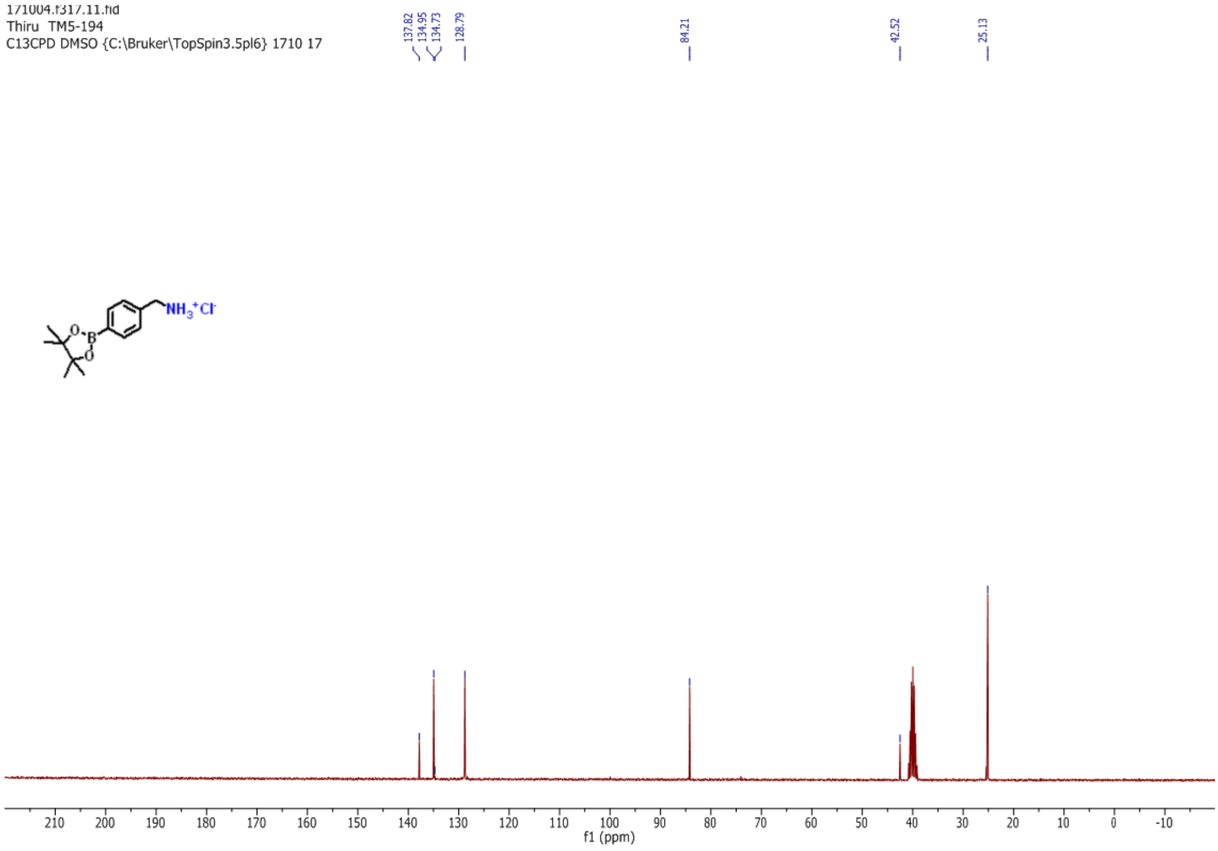

Supplementary Figure 57. ^{13}C NMR spectrum

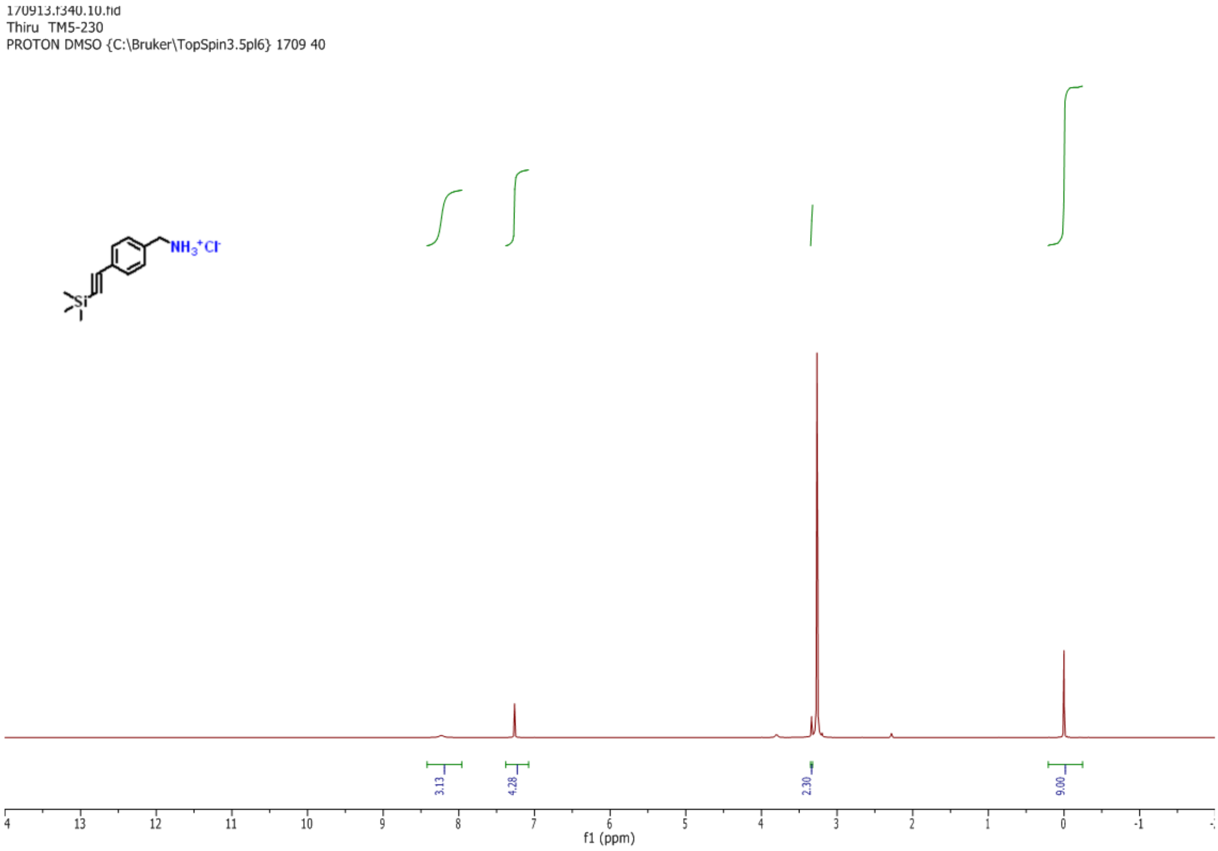

Supplementary Figure 58. ^{1}H NMR spectrum

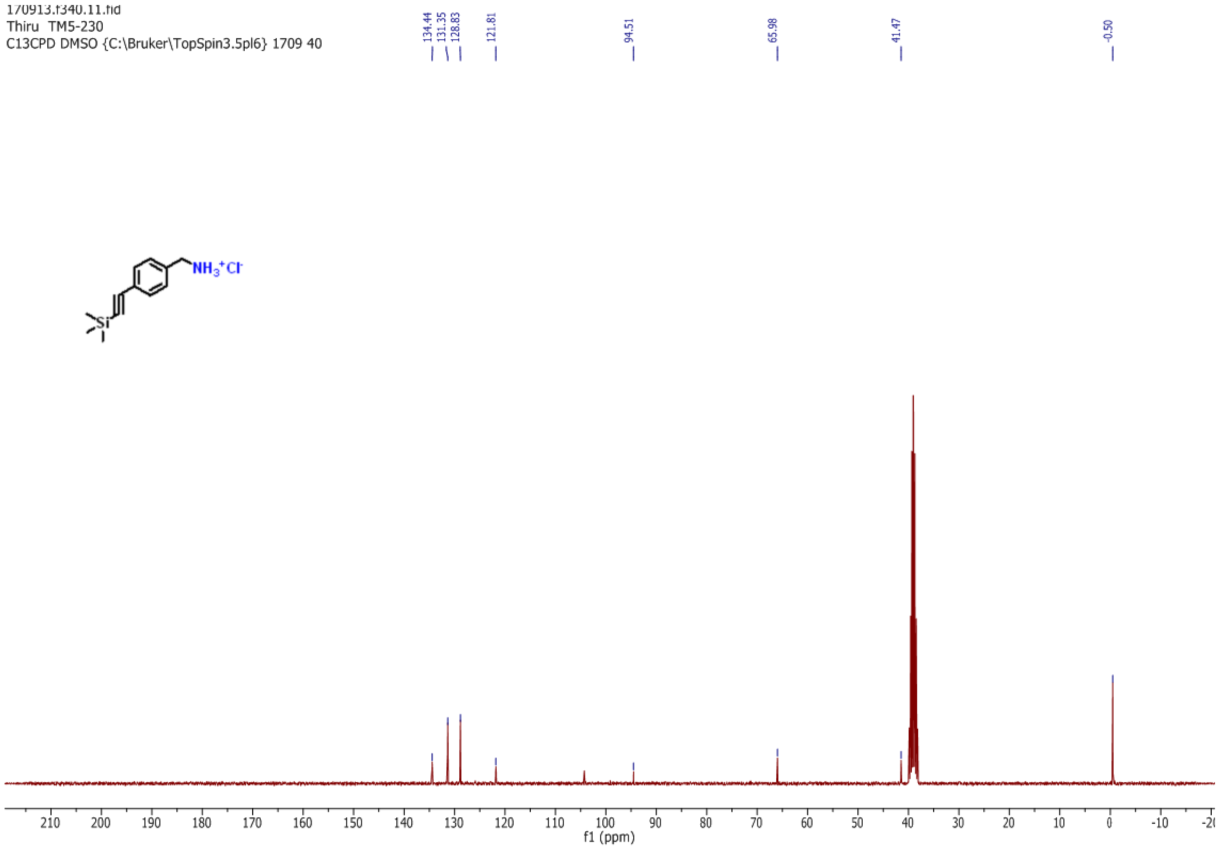

Supplementary Figure 59. ^{13}C NMR spectrum

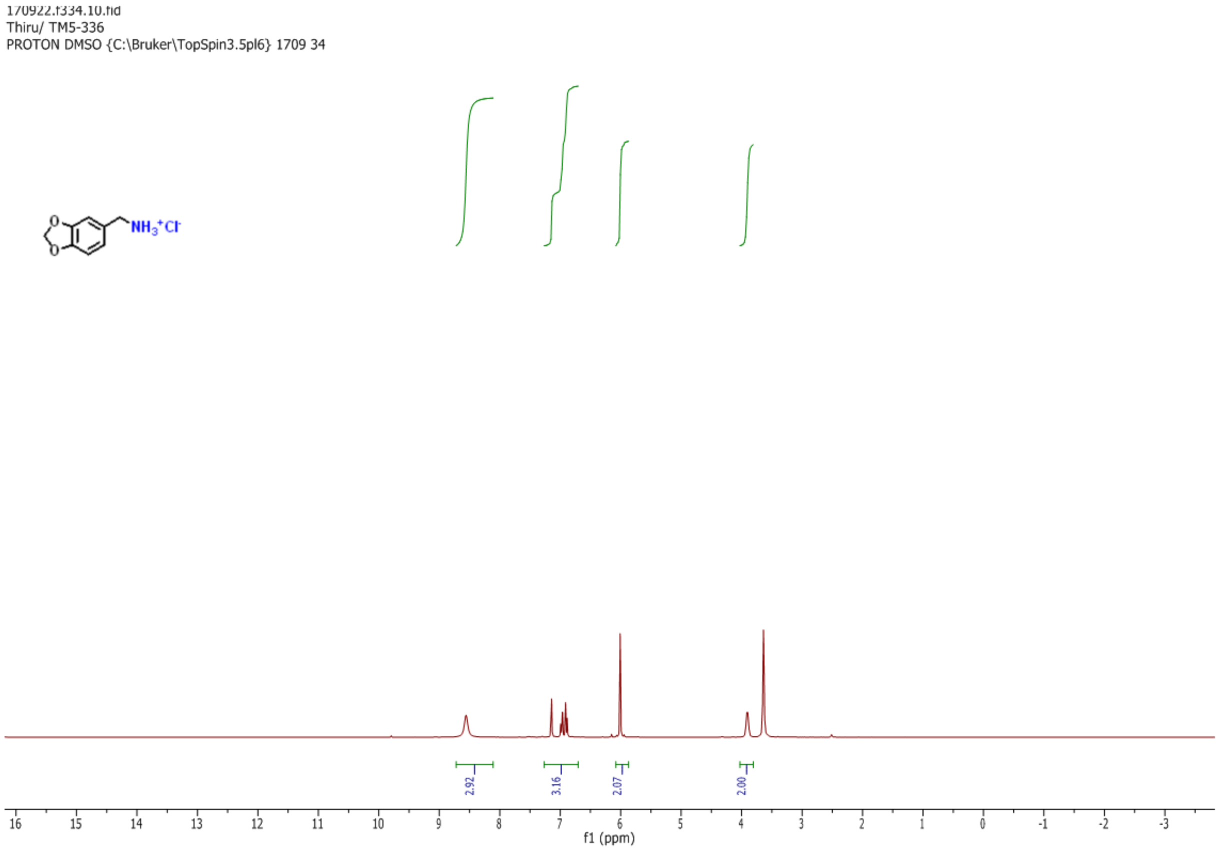

Supplementary Figure 60. ^{1}H NMR spectrum

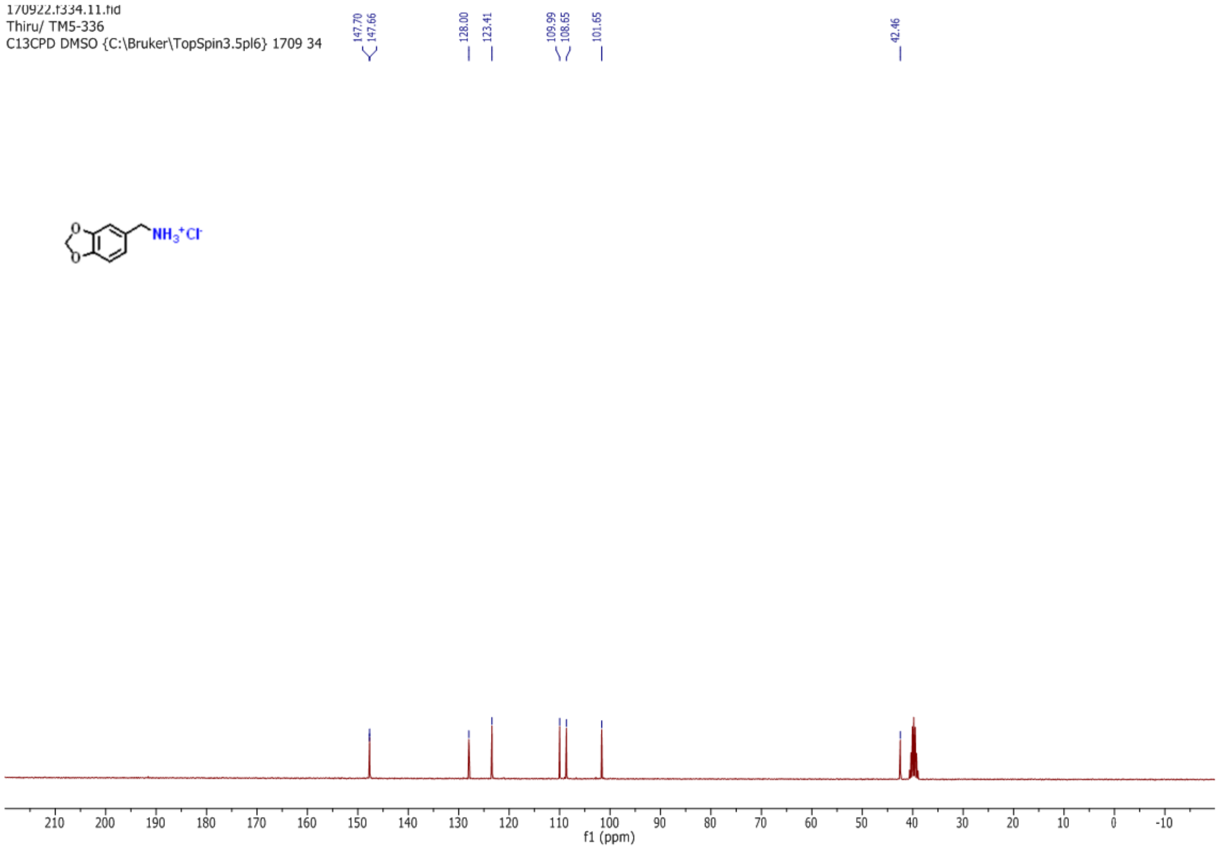

Supplementary Figure 61. ^{13}C NMR spectrum

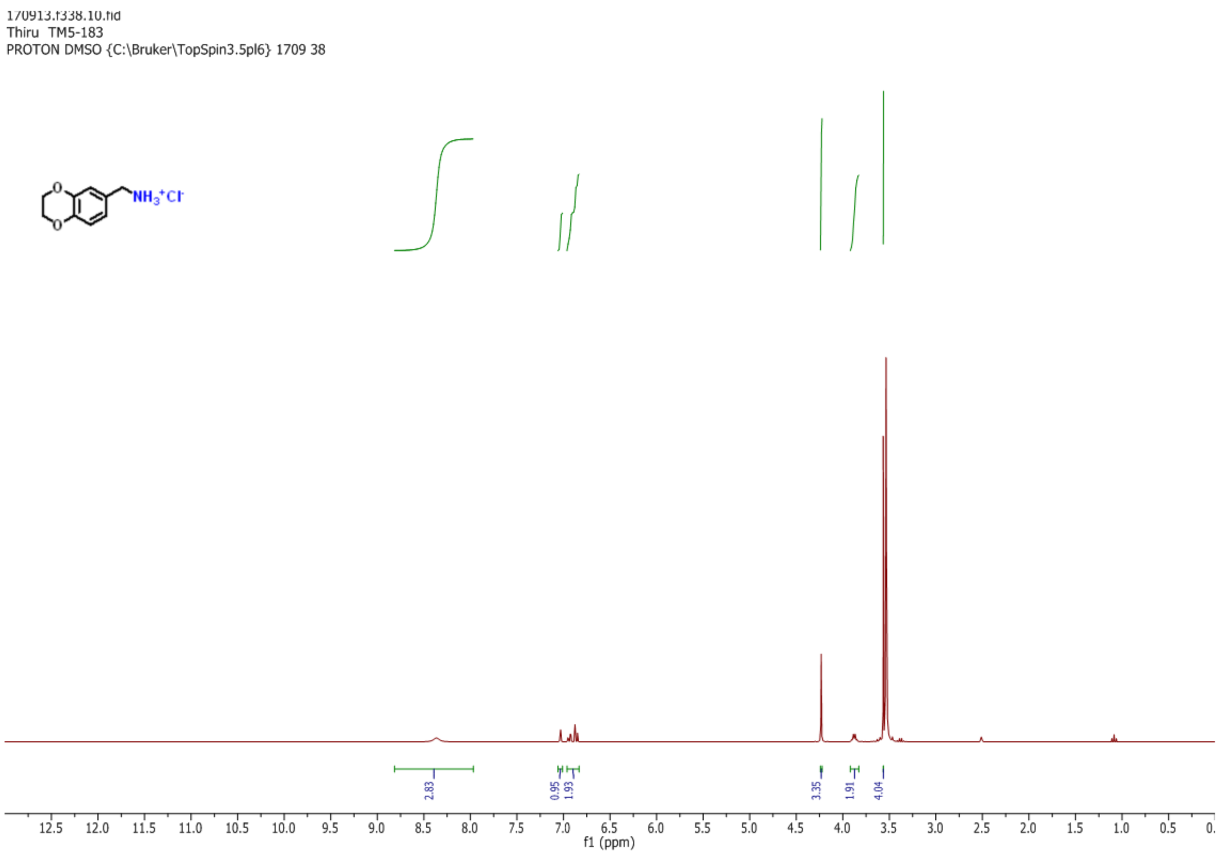

Supplementary Figure 62. ^{1}H NMR spectrum

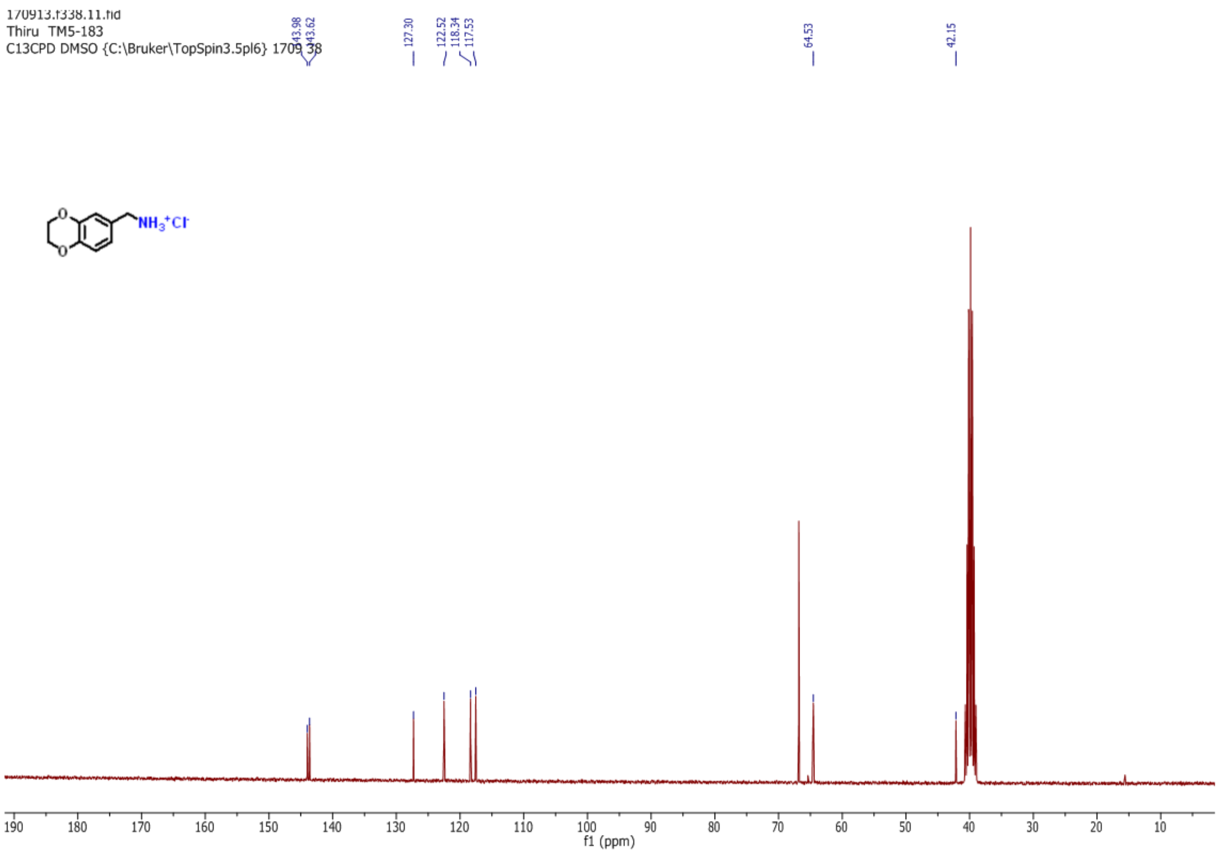

Supplementary Figure 63. ^{13}C NMR spectrum

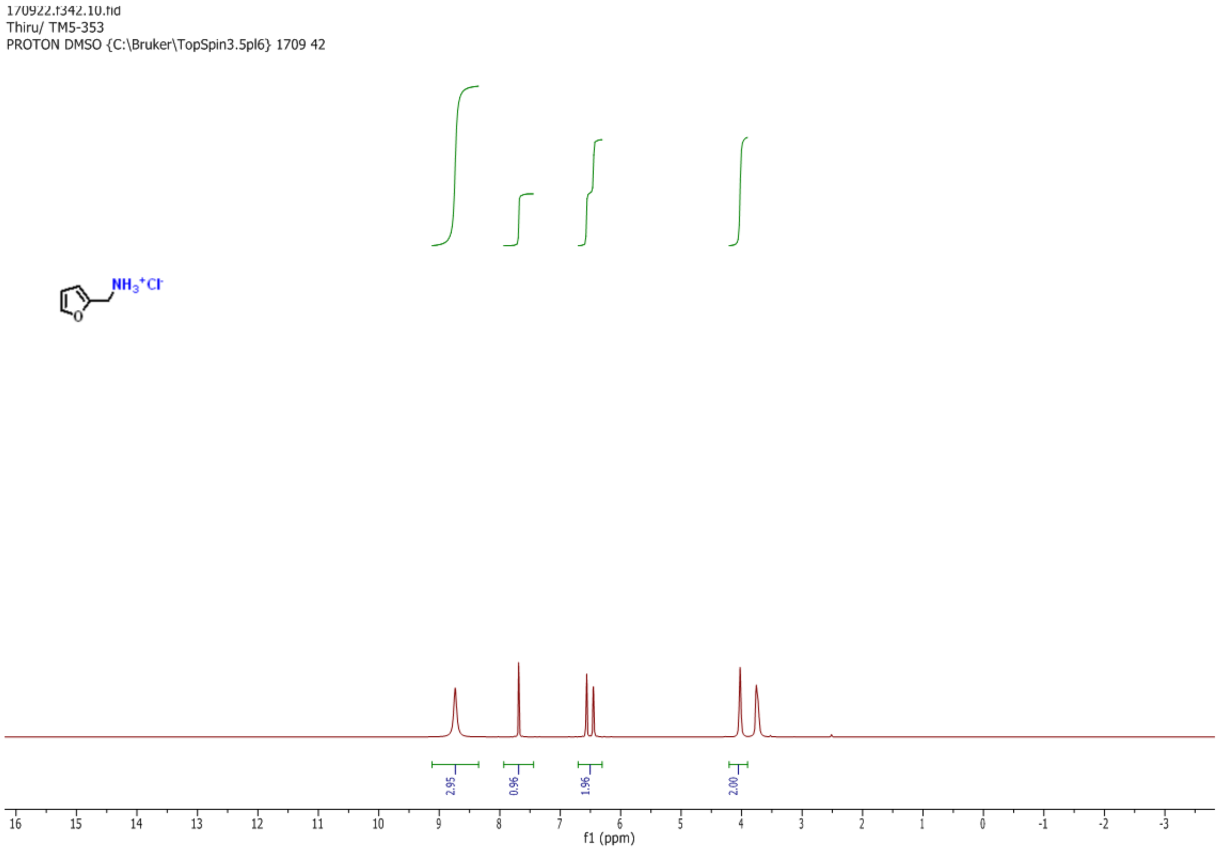

Supplementary Figure 64. ^{1}H NMR spectrum

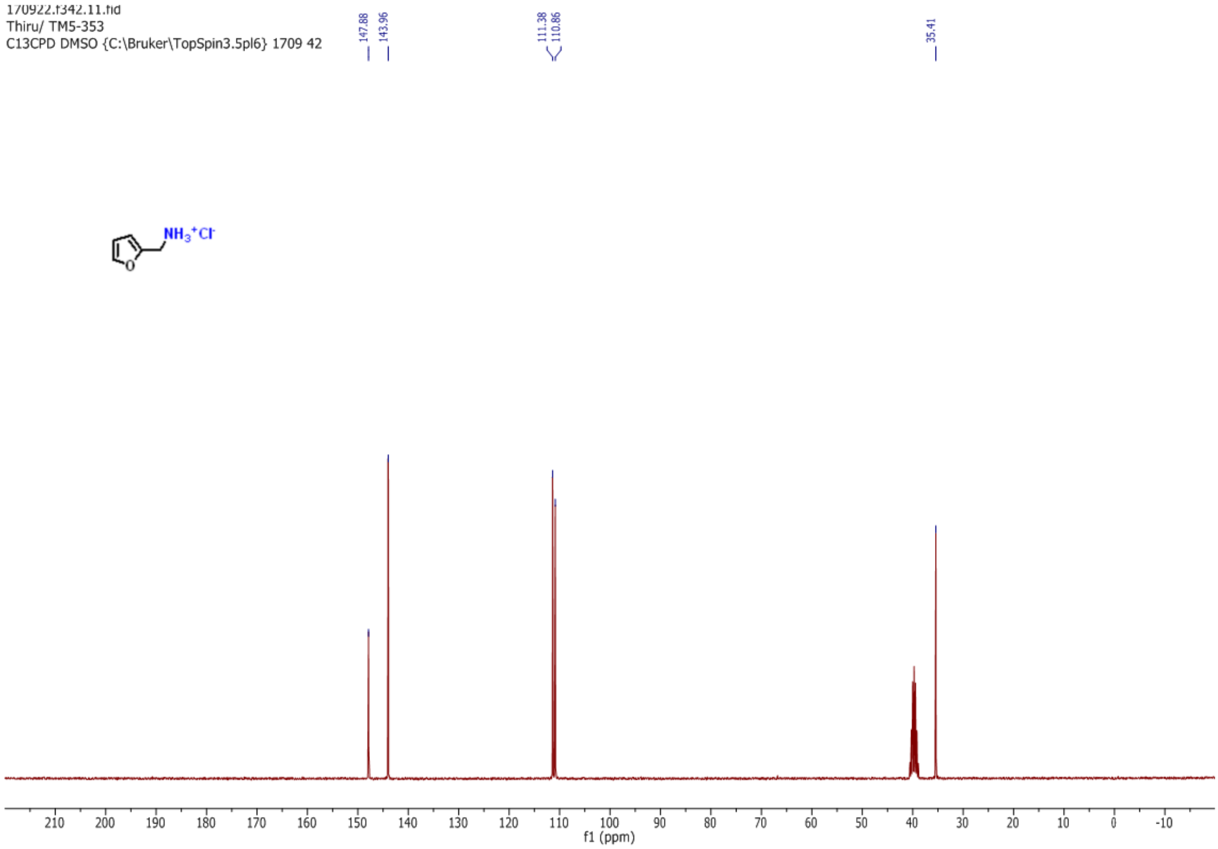

Supplementary Figure 65. ^{13}C NMR spectrum

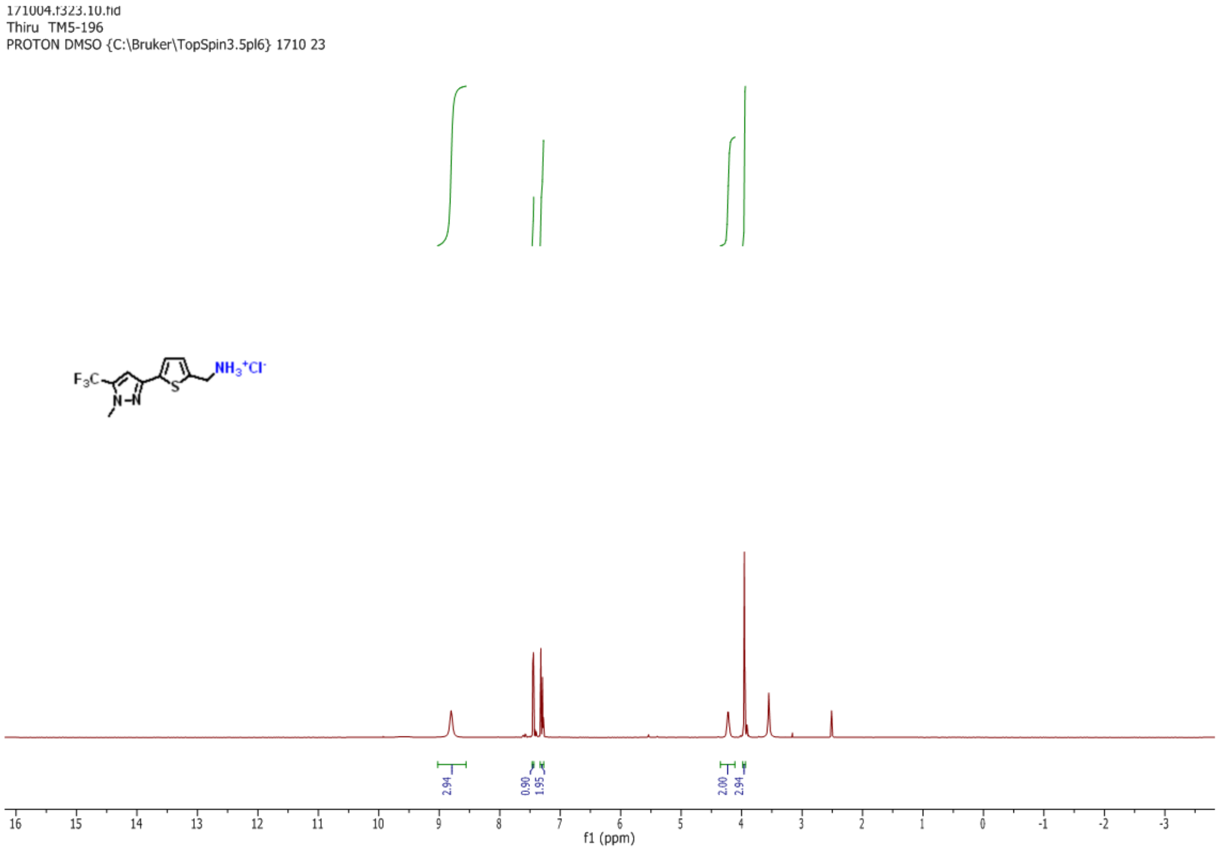

Supplementary Figure 66. ^{1}H NMR spectrum

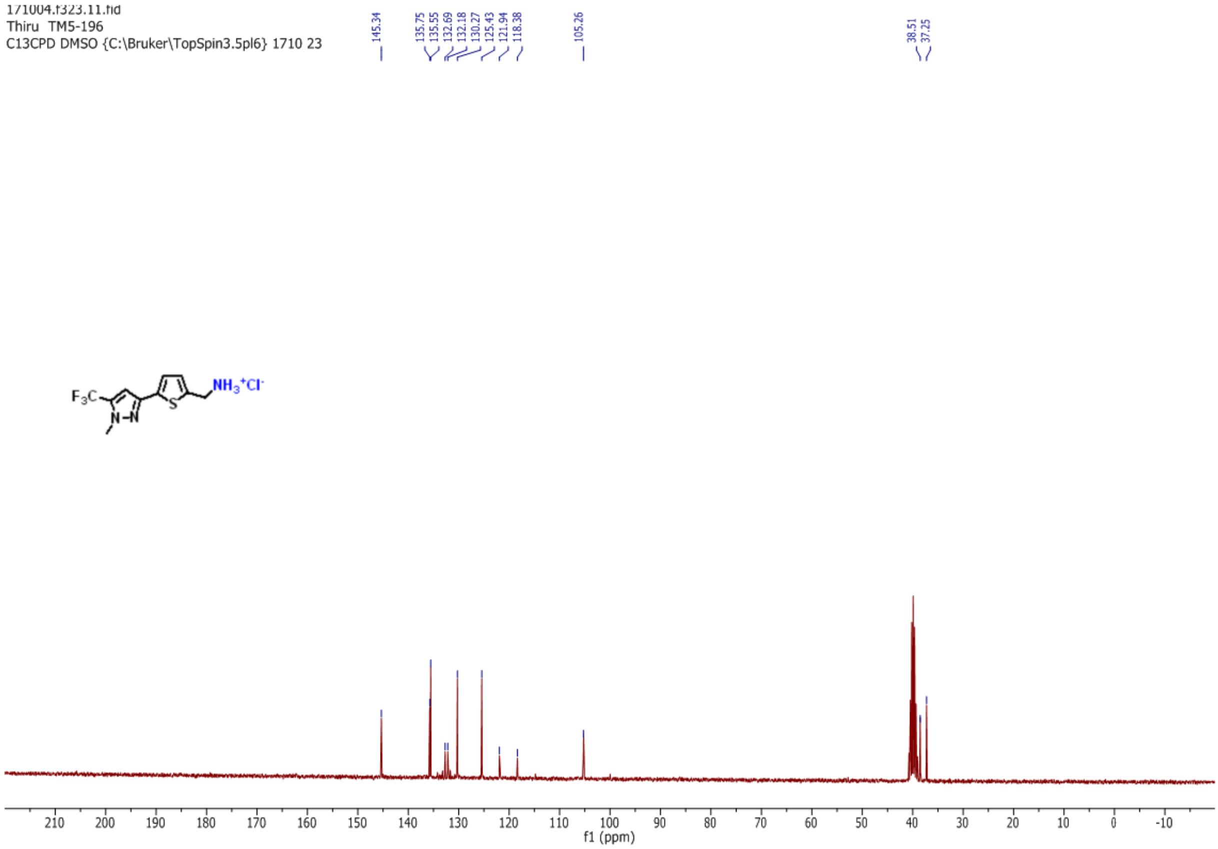

Supplementary Figure 67. ^{13}C NMR spectrum

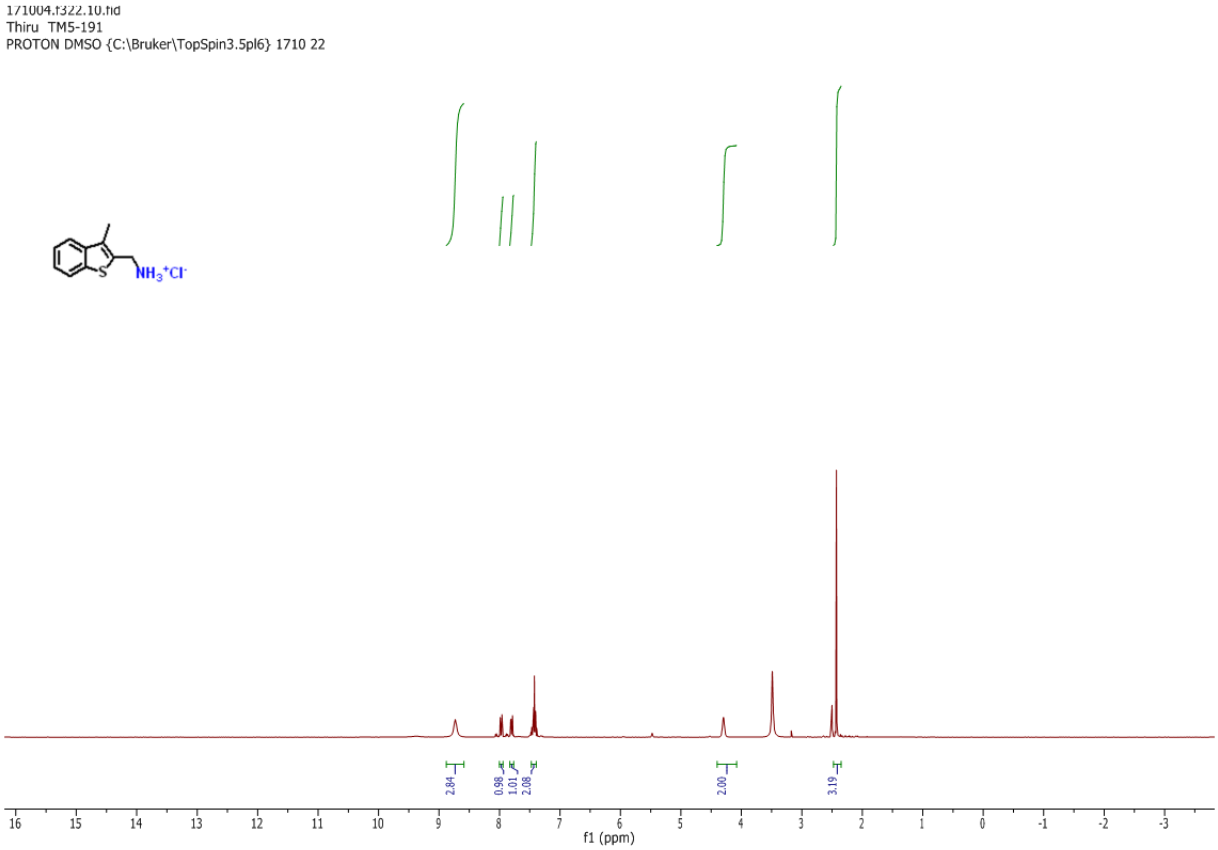

Supplementary Figure 68. ^{1}H NMR spectrum

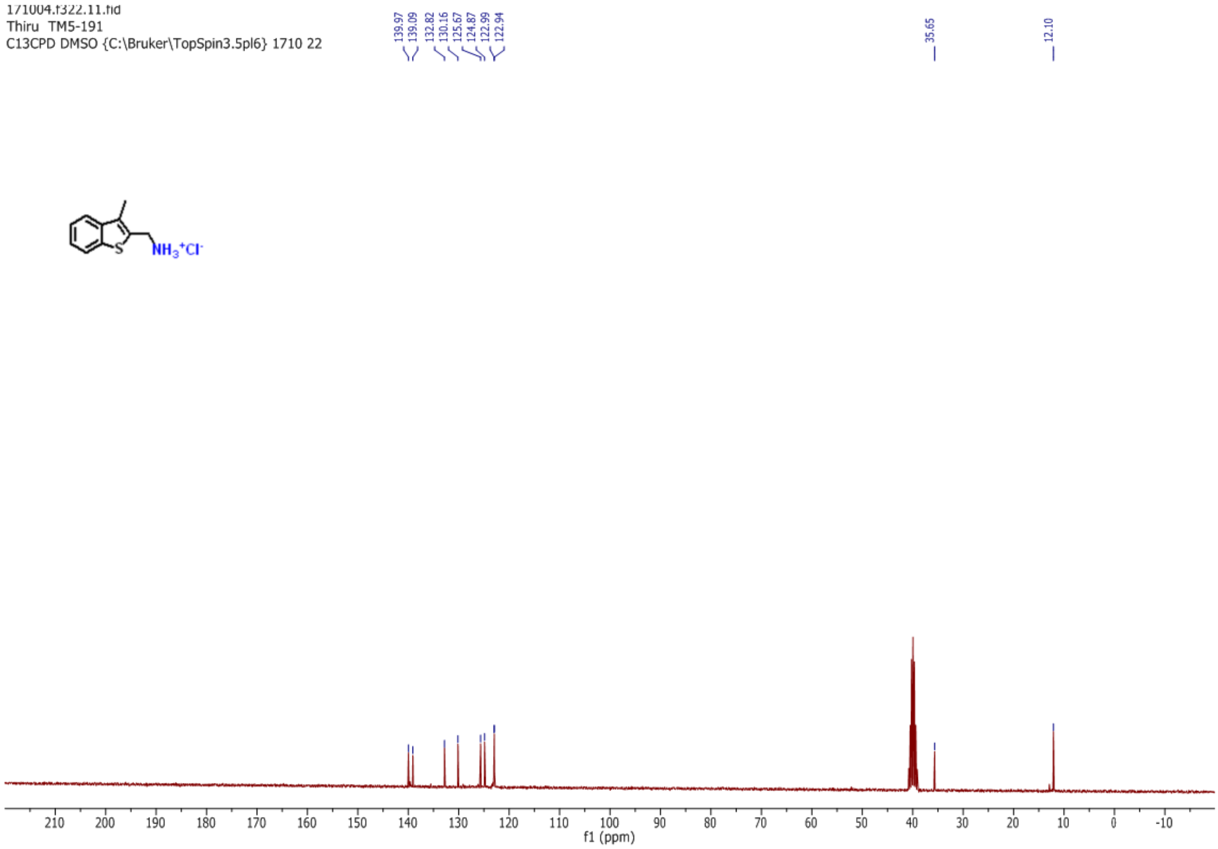

Supplementary Figure 69. ^{13}C NMR spectrum

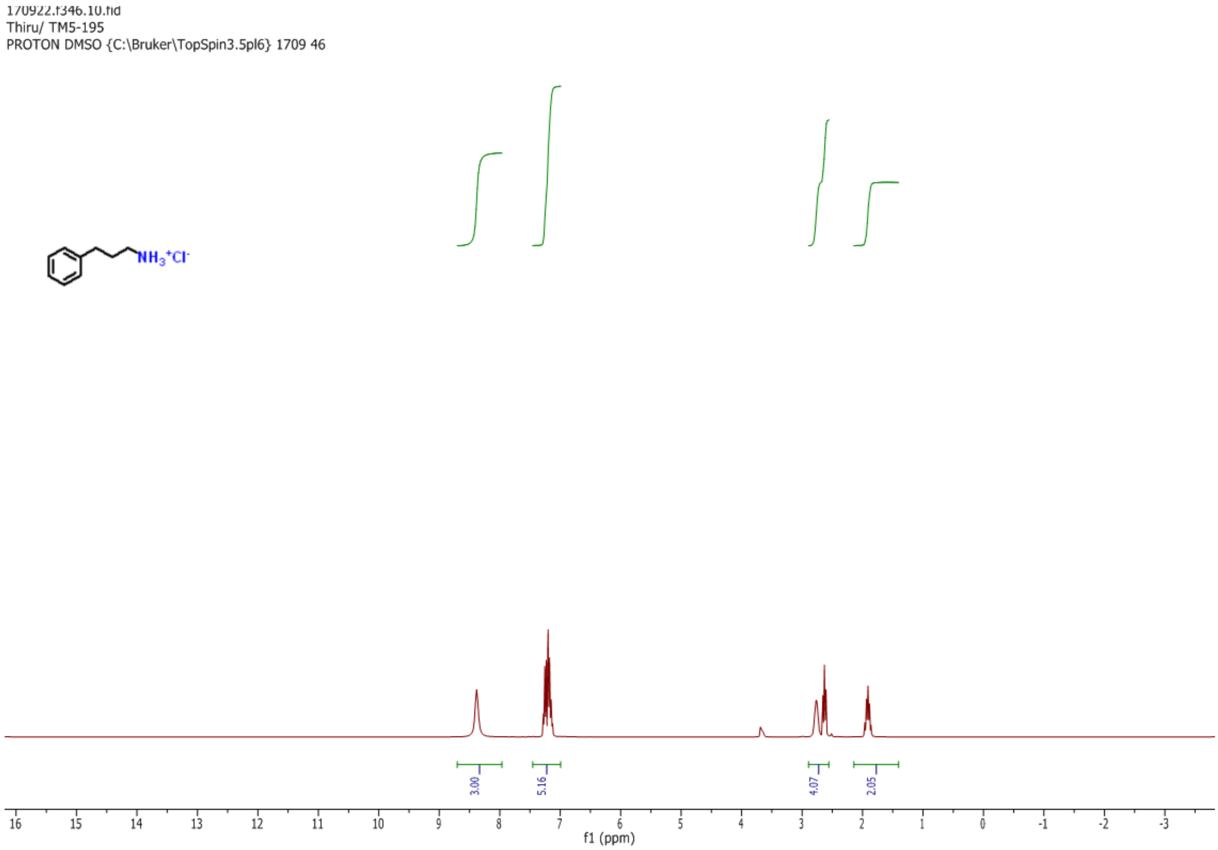

Supplementary Figure 70. ^{1}H NMR spectrum

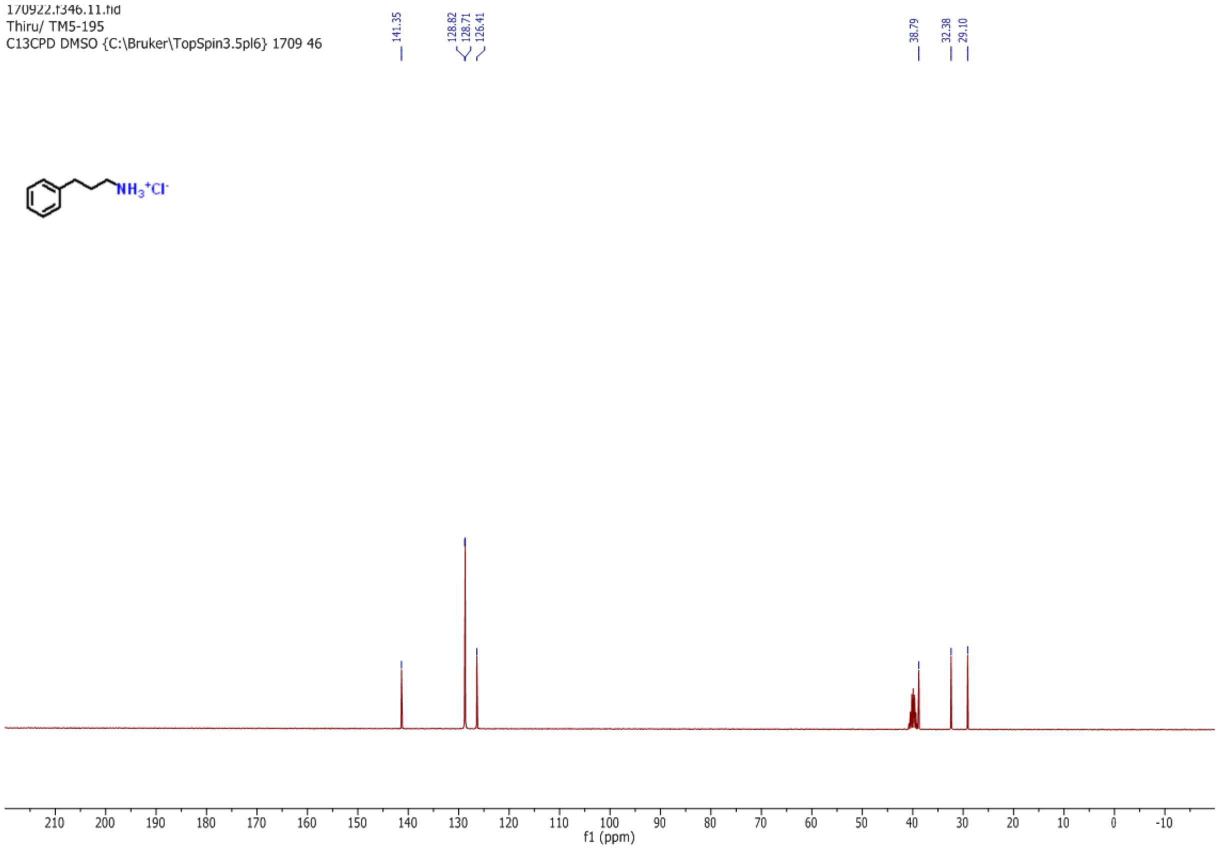

Supplementary Figure 71. ^{13}C NMR spectrum

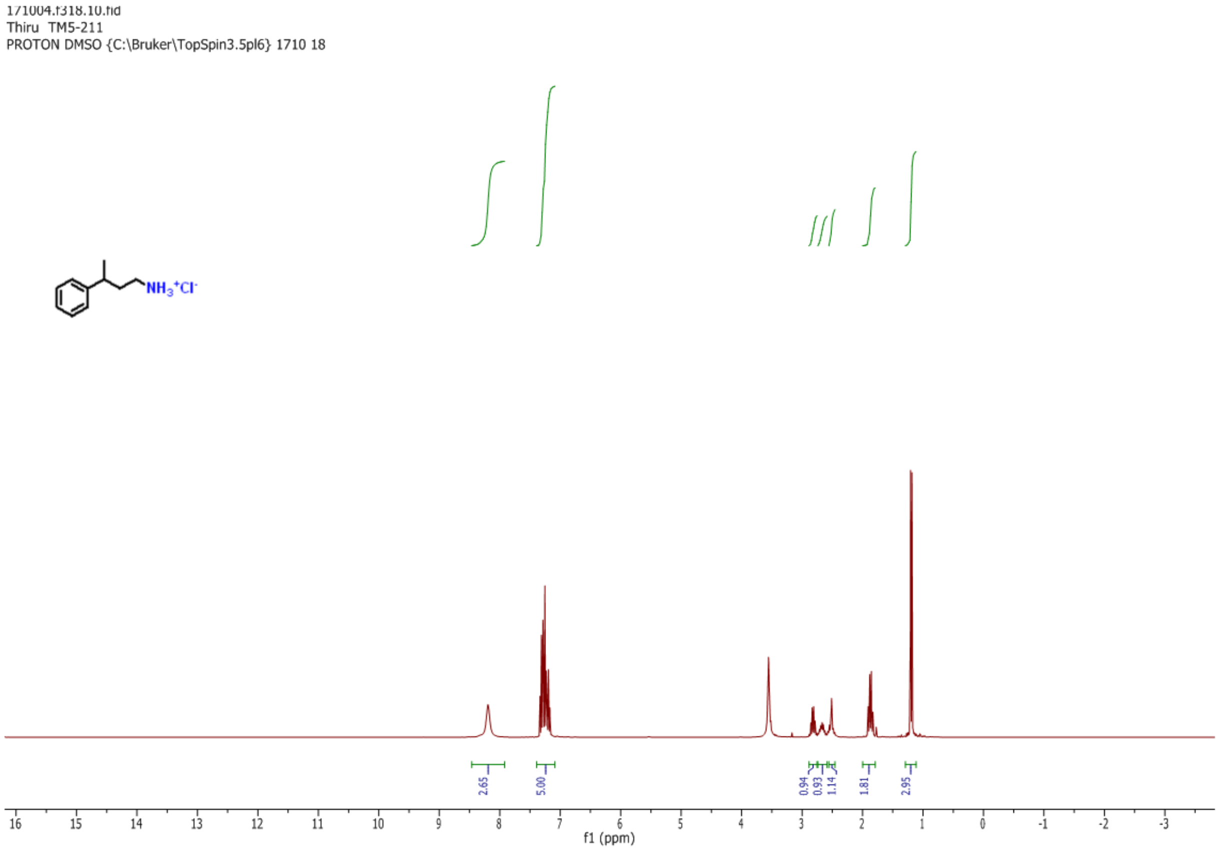

Supplementary Figure 72. ^{1}H NMR spectrum

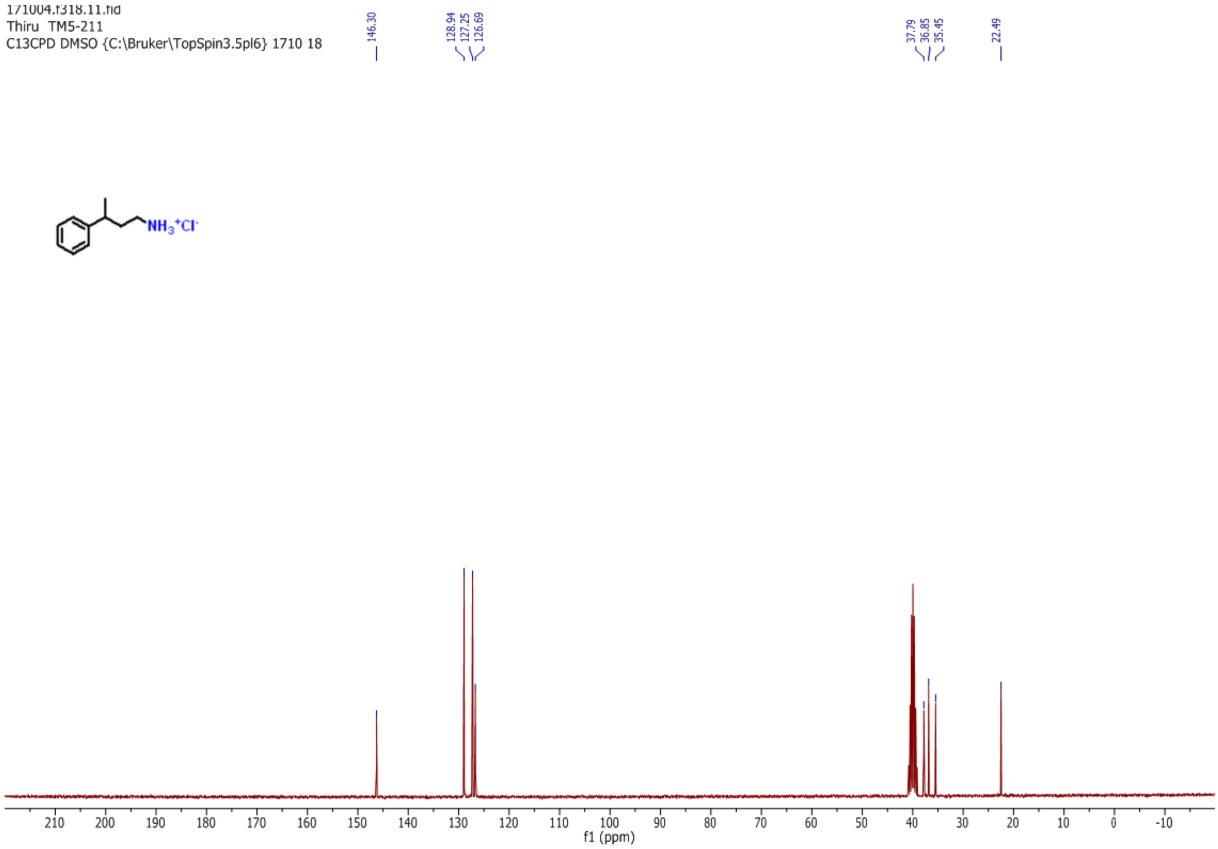

Supplementary Figure 73. ^{13}C NMR spectrum

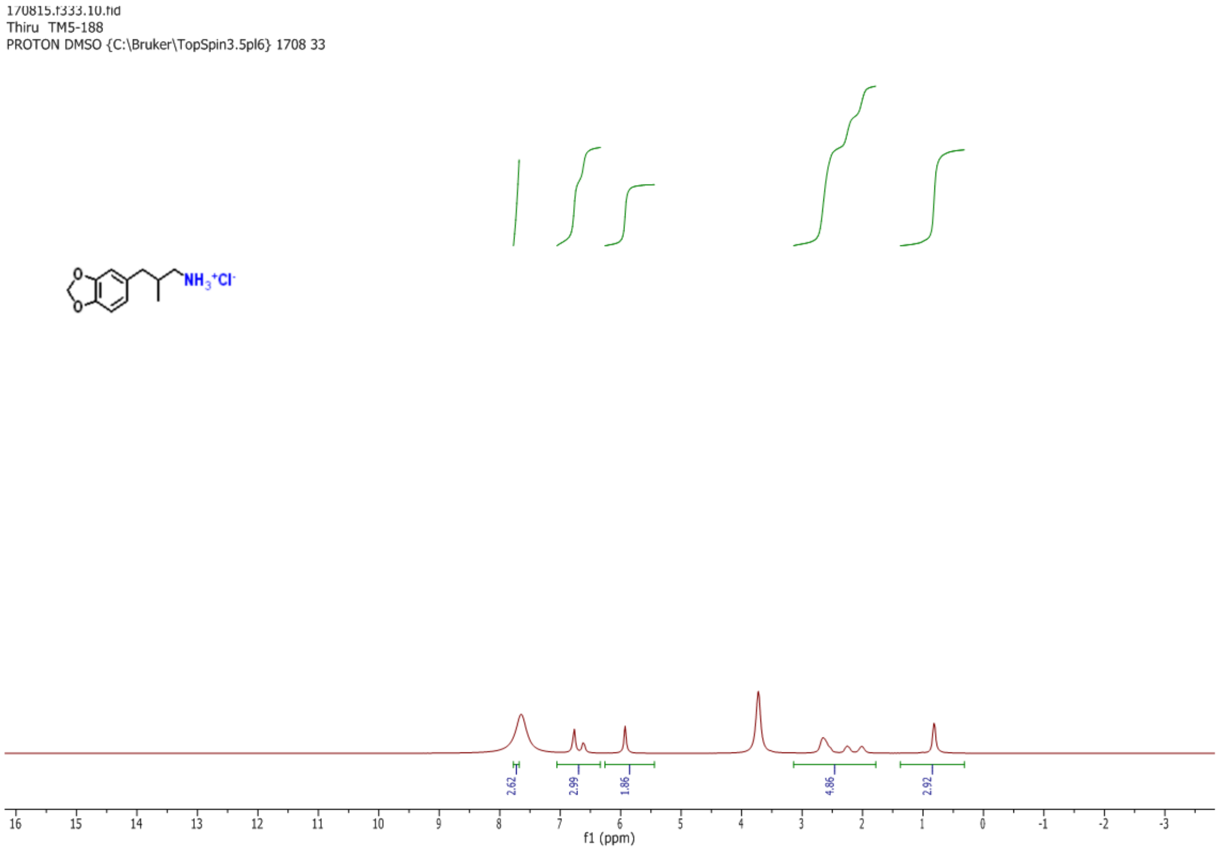

Supplementary Figure 74. ^{1}H NMR spectrum

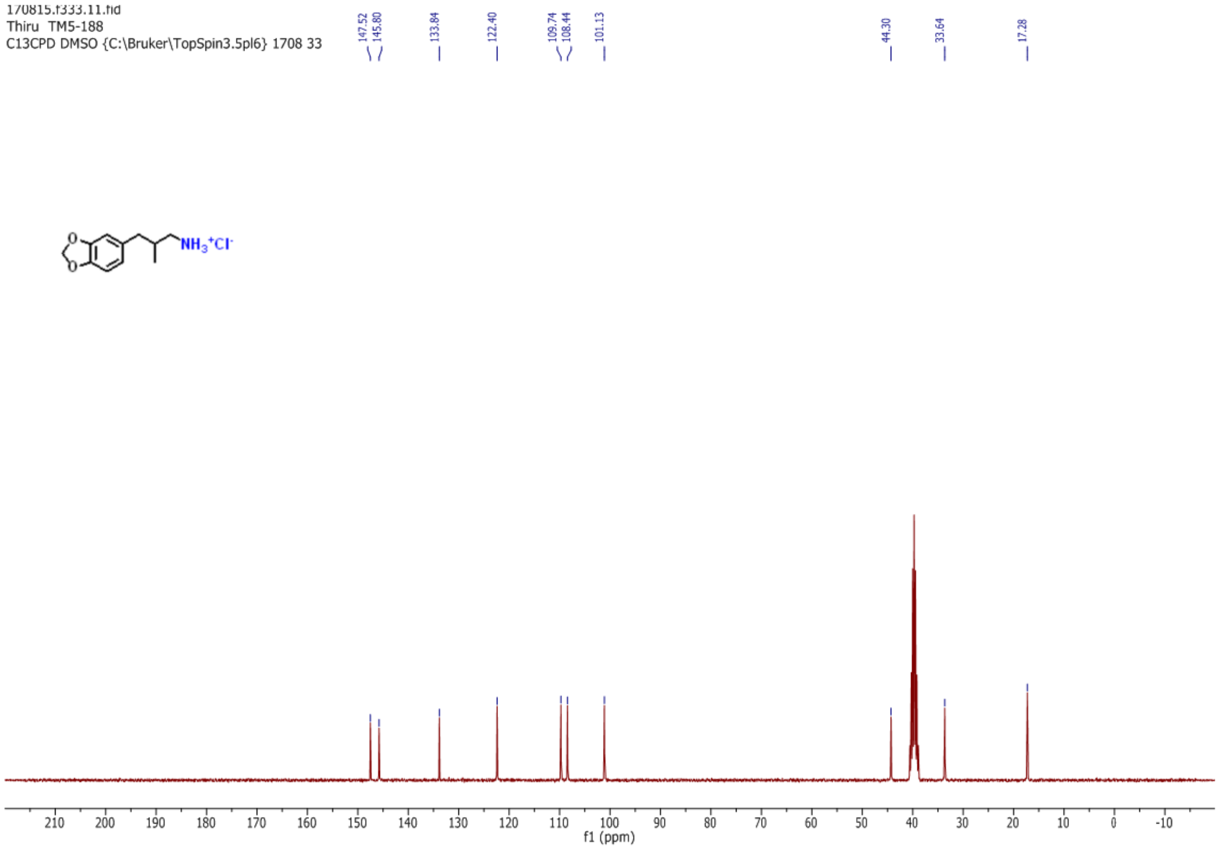

Supplementary Figure 75. ^{13}C NMR spectrum

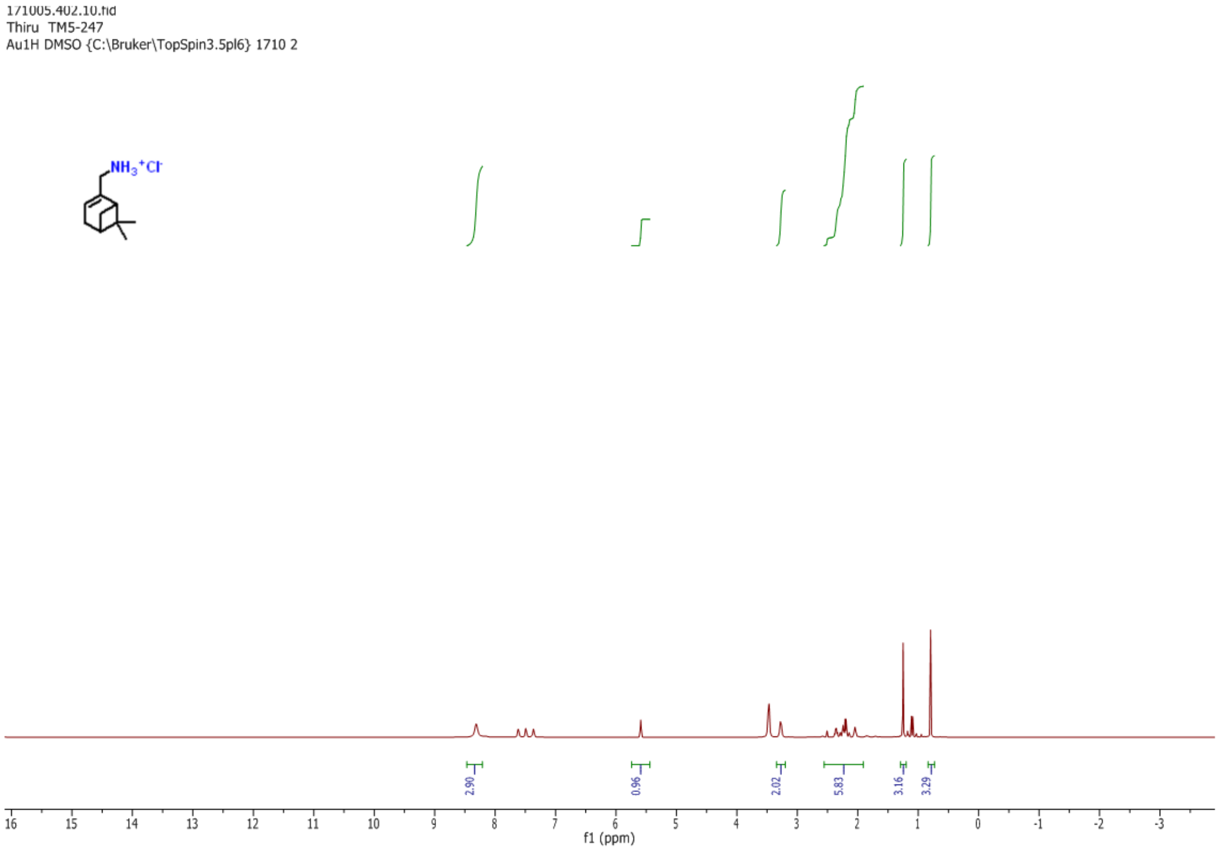

Supplementary Figure 76. ^{1}H NMR spectrum

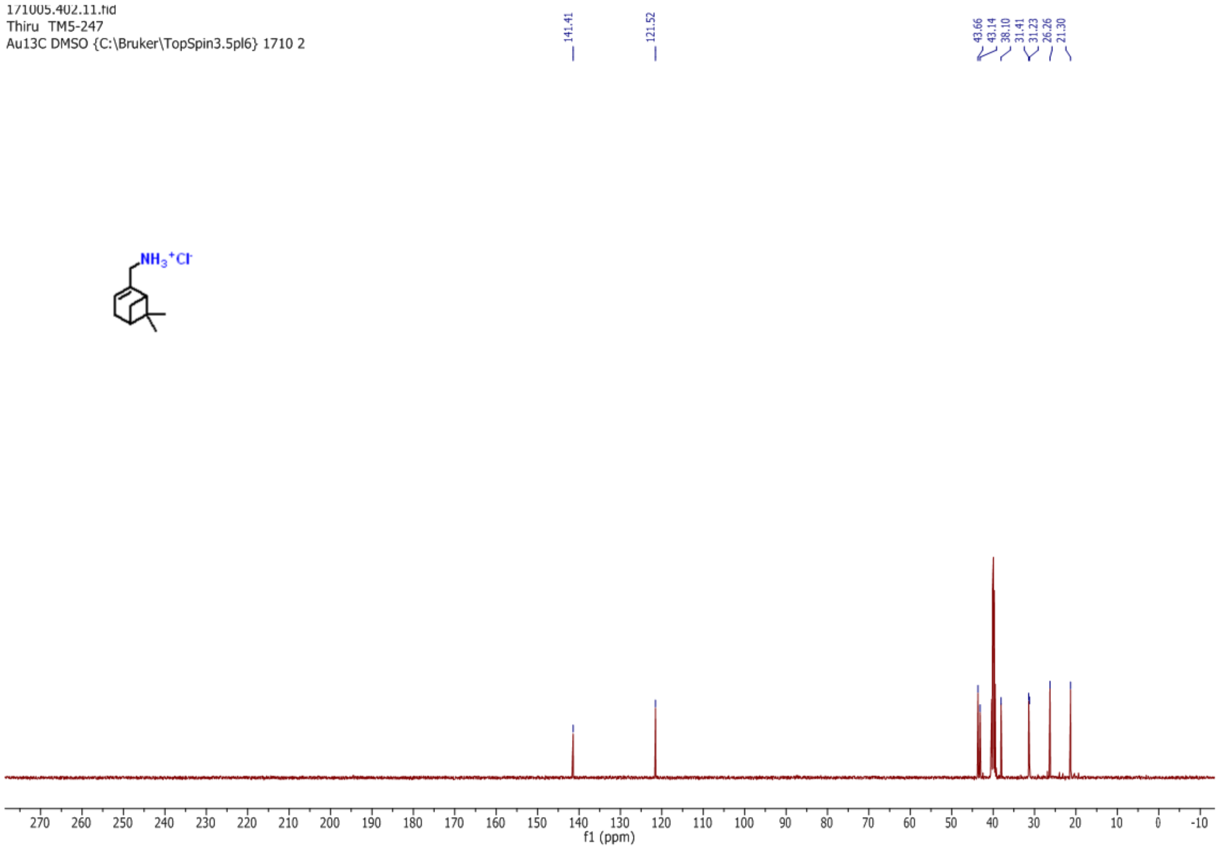

Supplementary Figure 77. ^{13}C NMR spectrum

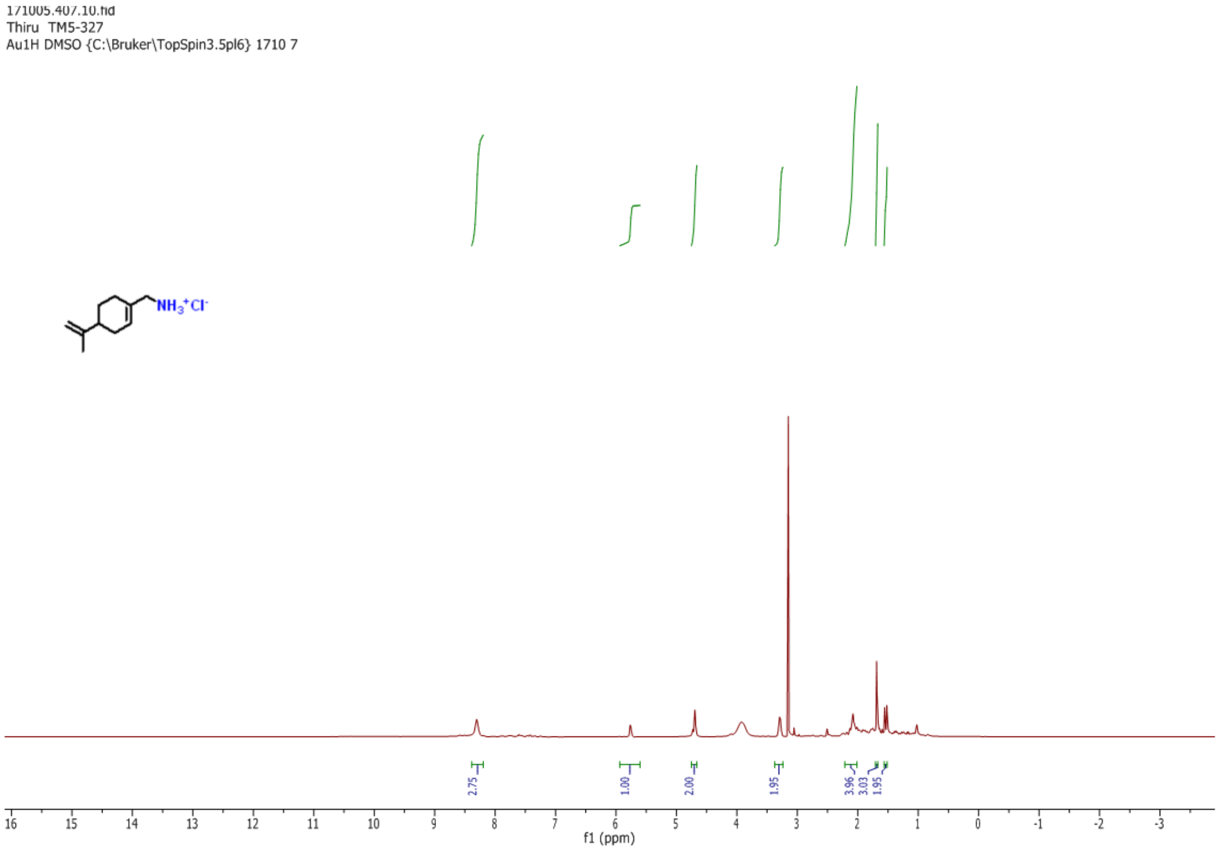

Supplementary Figure 78. ^{1}H NMR spectrum

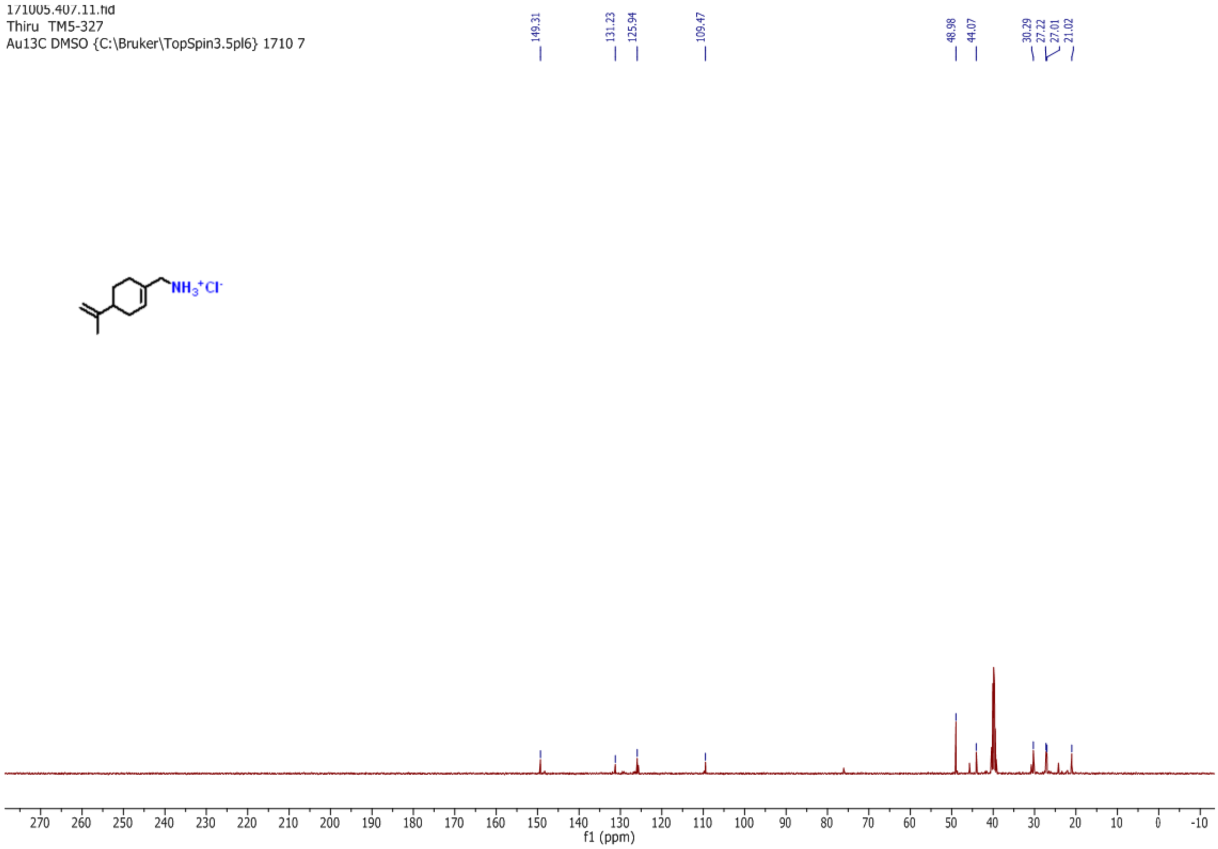

Supplementary Figure 79. ^{13}C NMR spectrum

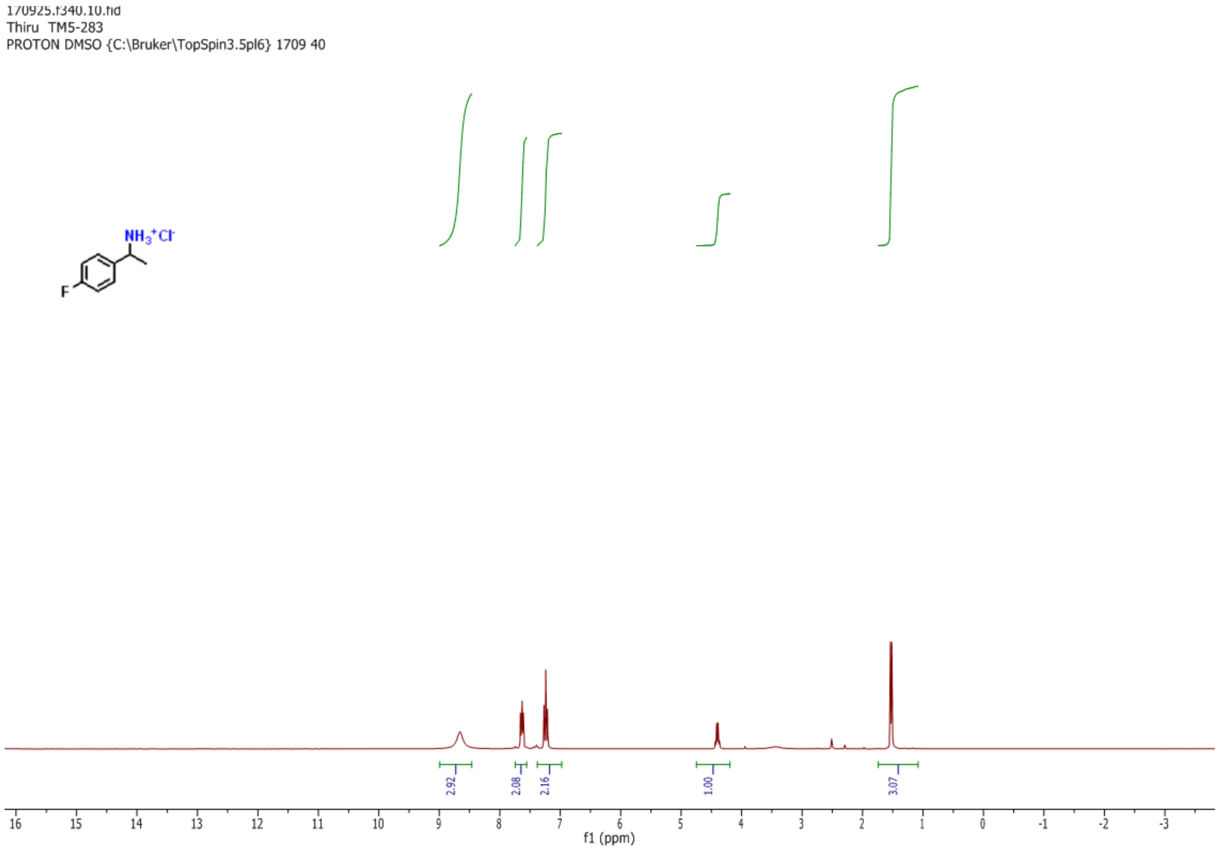

Supplementary Figure 80. ^{1}H NMR spectrum

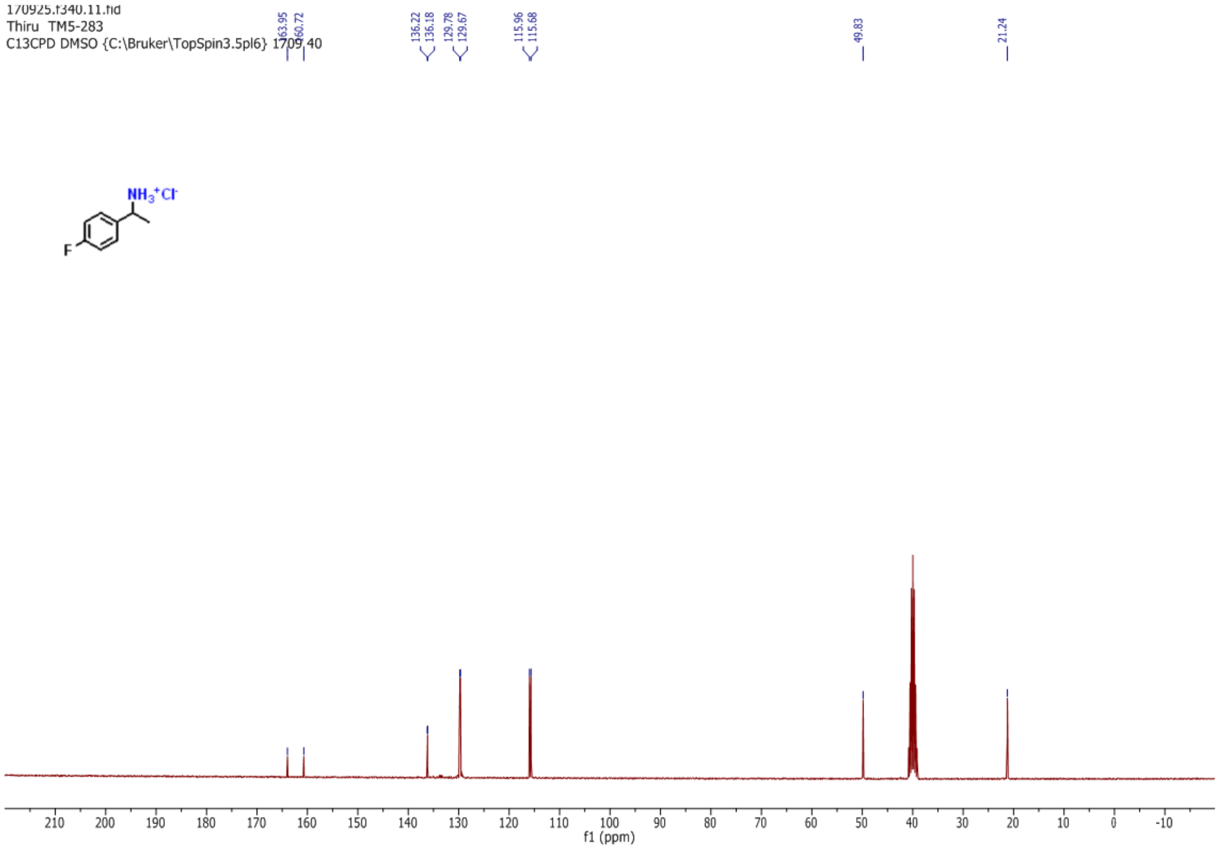

Supplementary Figure 81. ^{13}C NMR spectrum

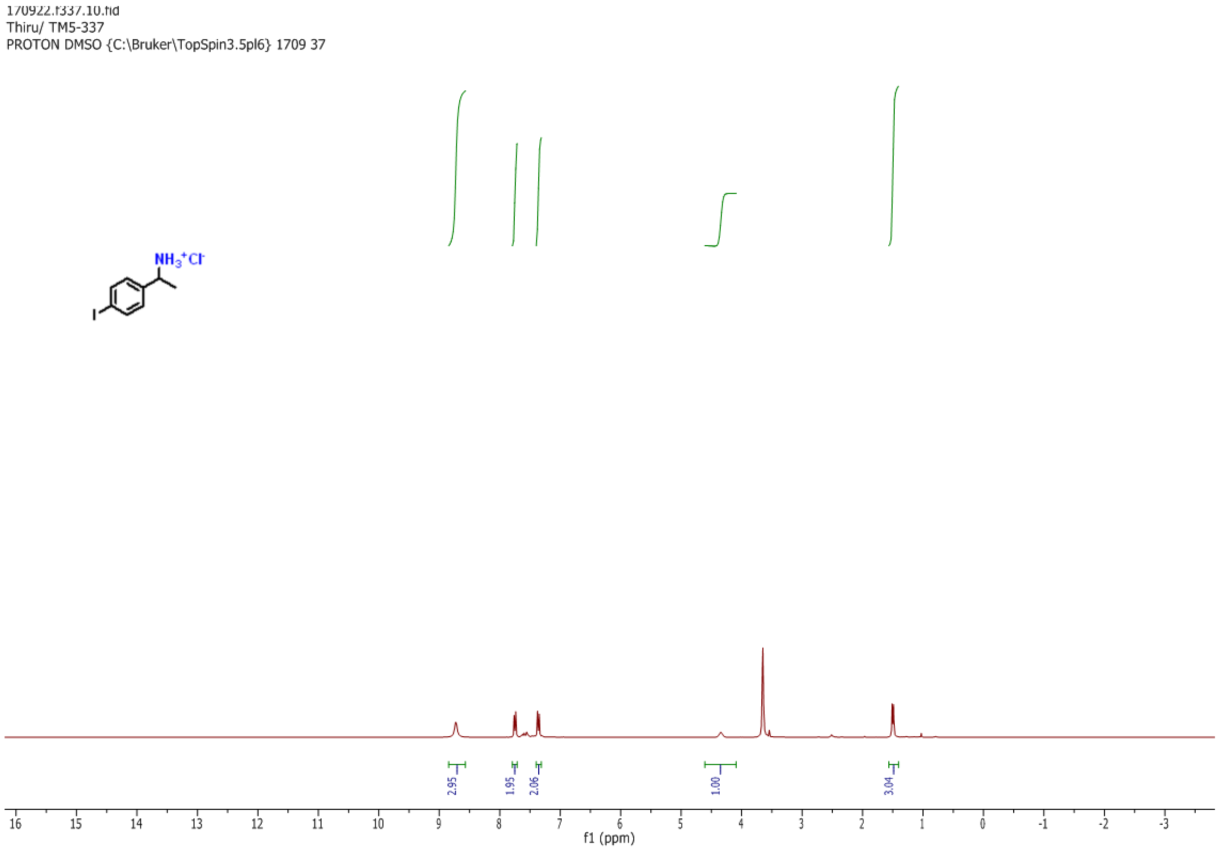

Supplementary Figure 82. ^{1}H NMR spectrum

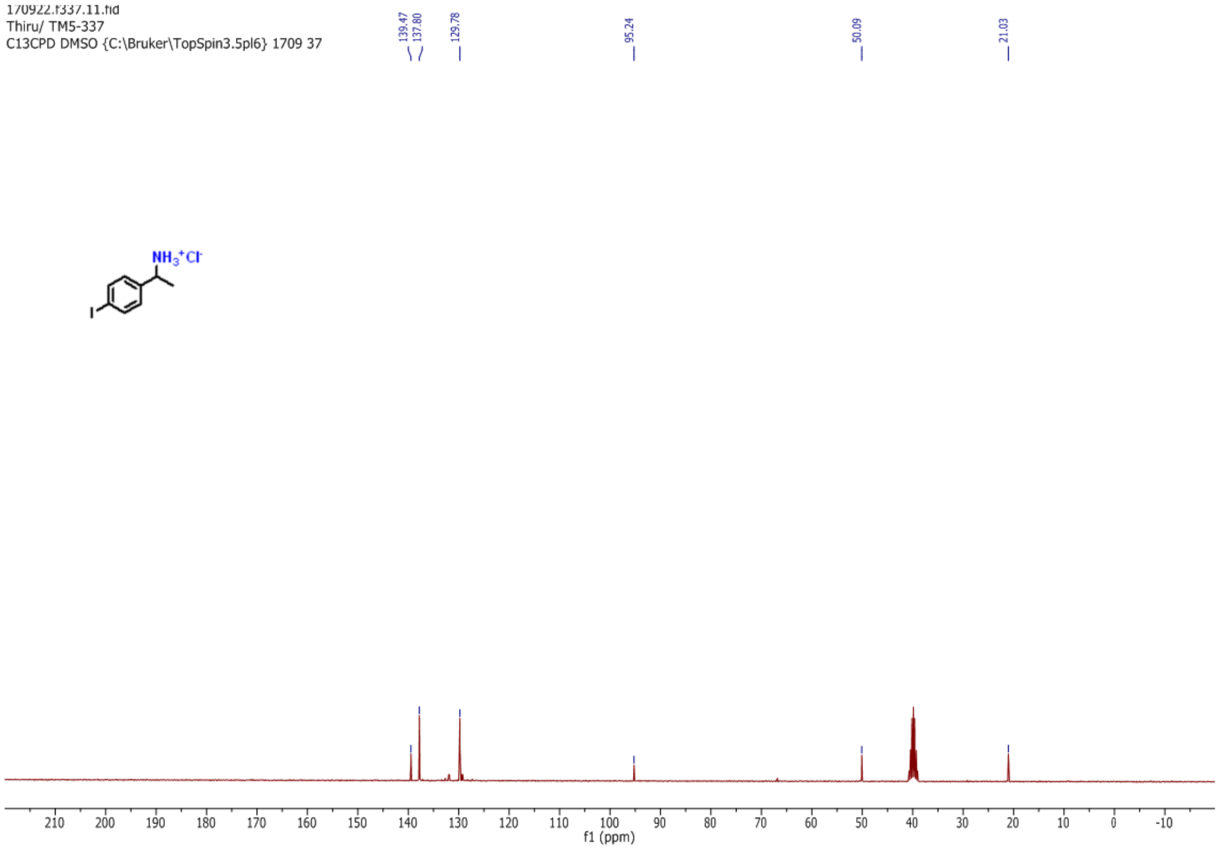

Supplementary Figure 83. ^{13}C NMR spectrum

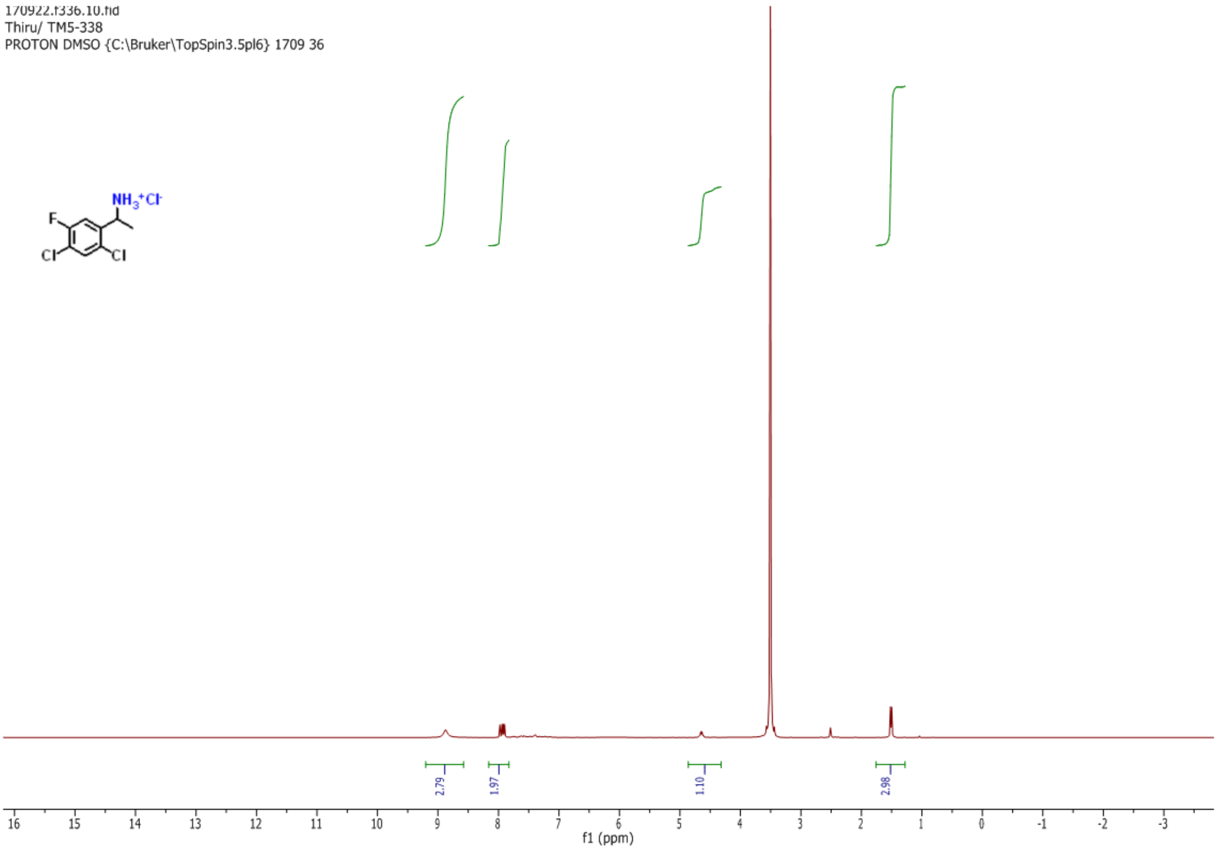

Supplementary Figure 84. ^{1}H NMR spectrum

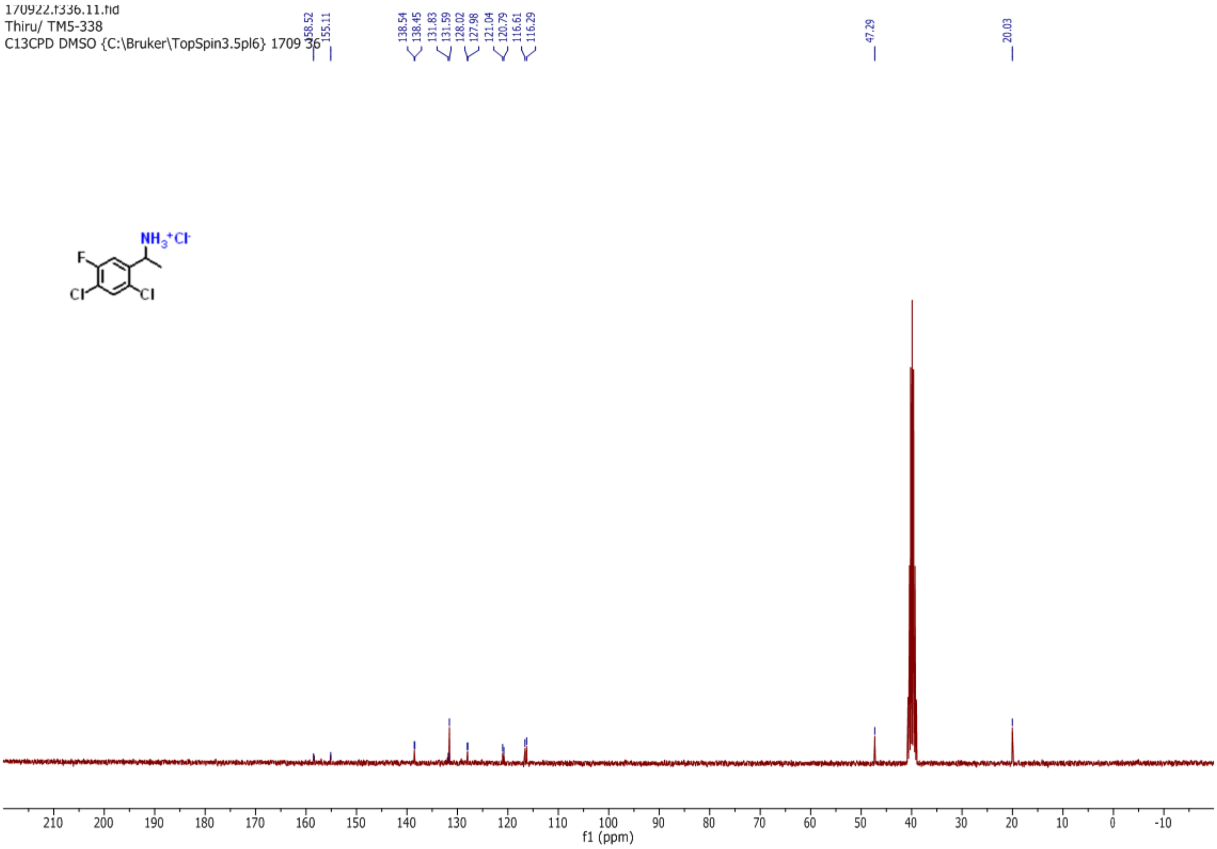

Supplementary Figure 85. ^{13}C NMR spectrum

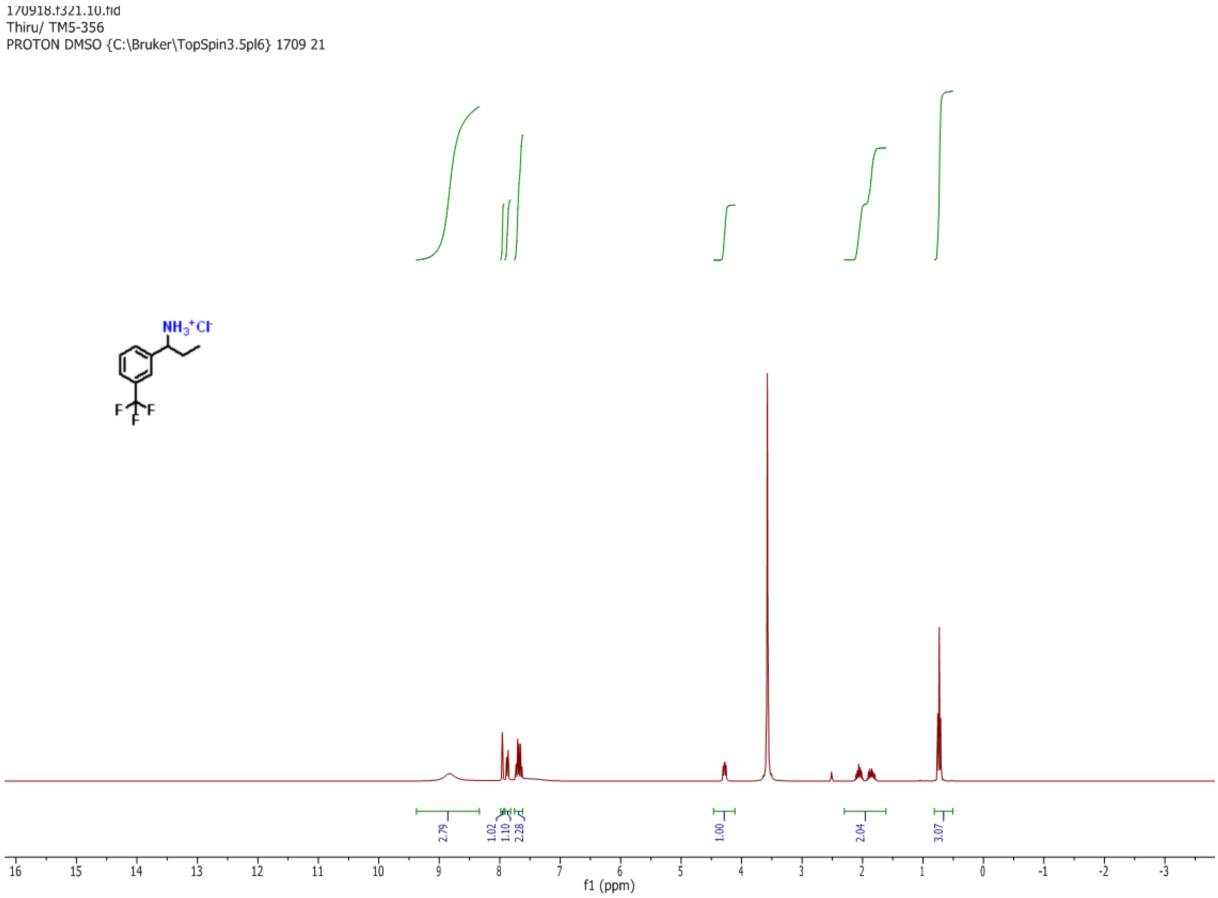

Supplementary Figure 86. ^{1}H NMR spectrum

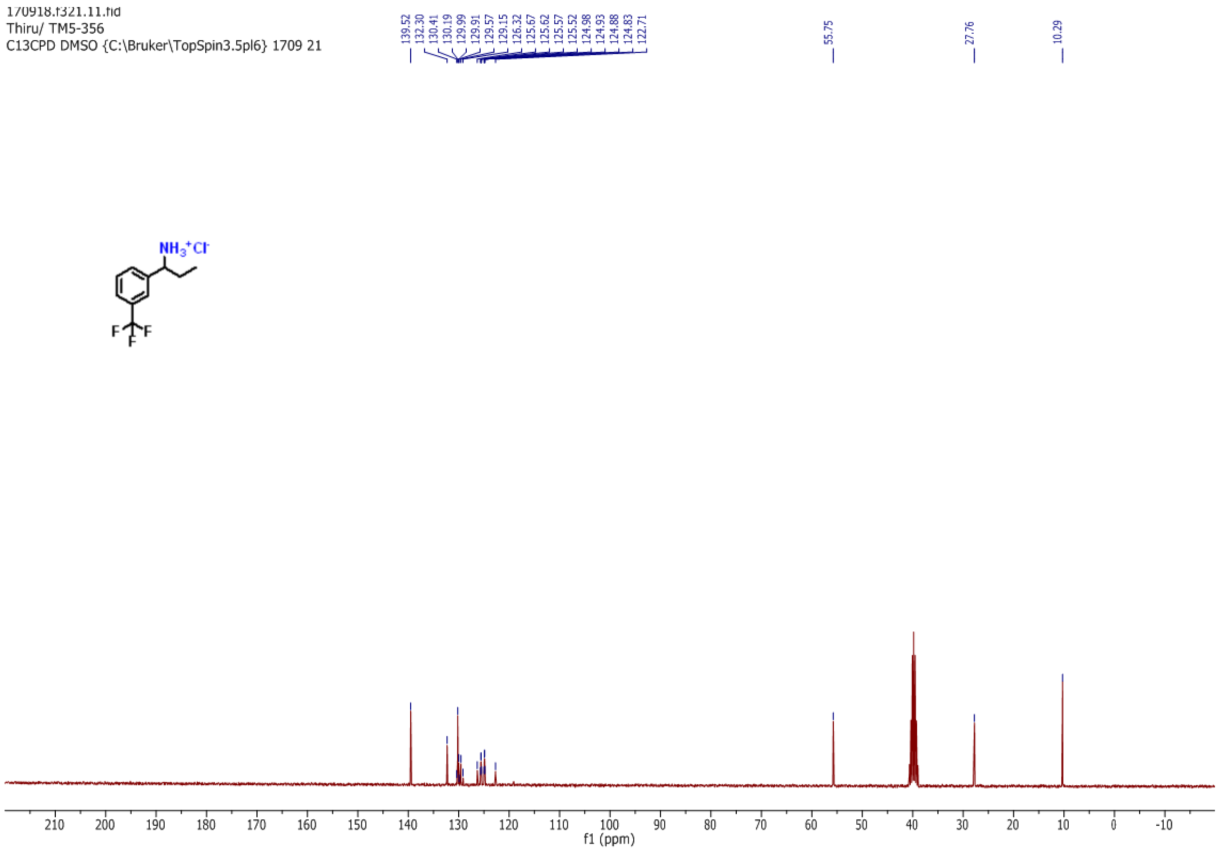

Supplementary Figure 87. ^{13}C NMR spectrum

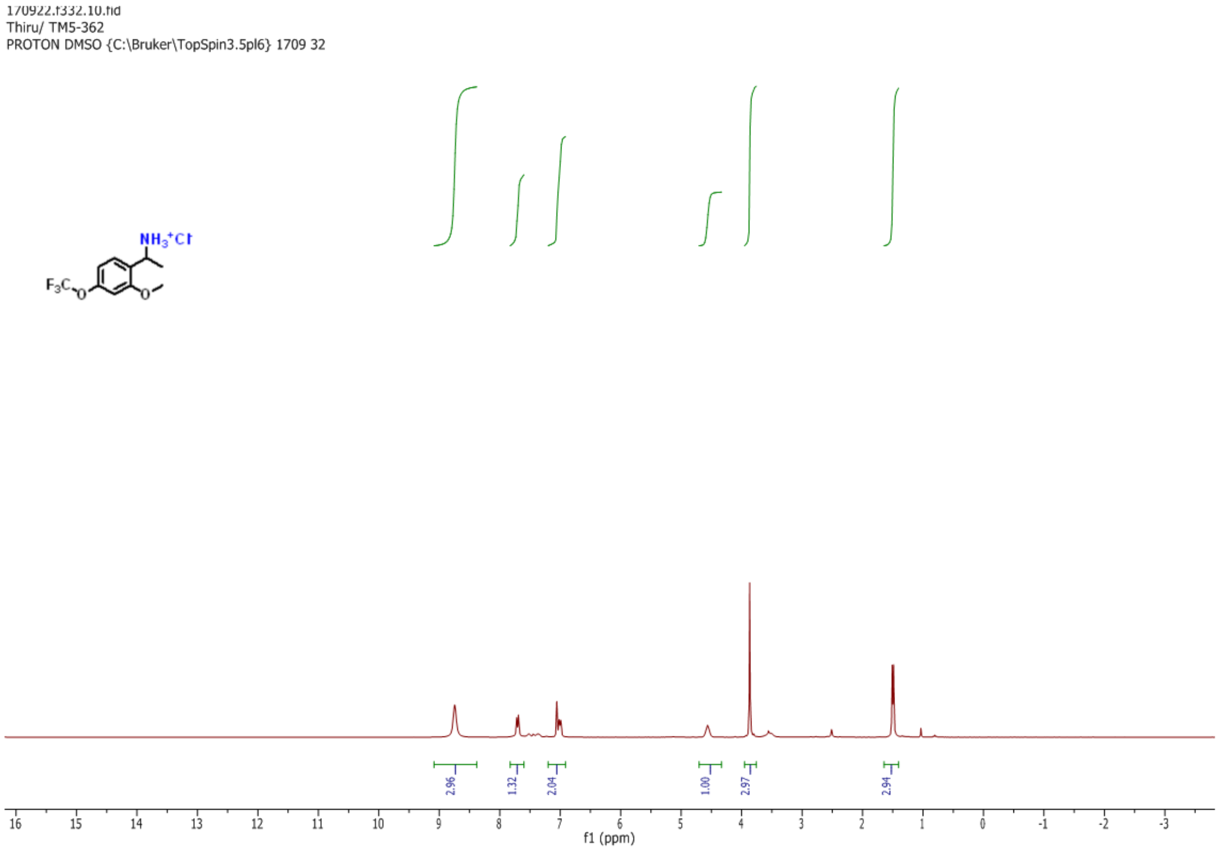

Supplementary Figure 88. ^{1}H NMR spectrum

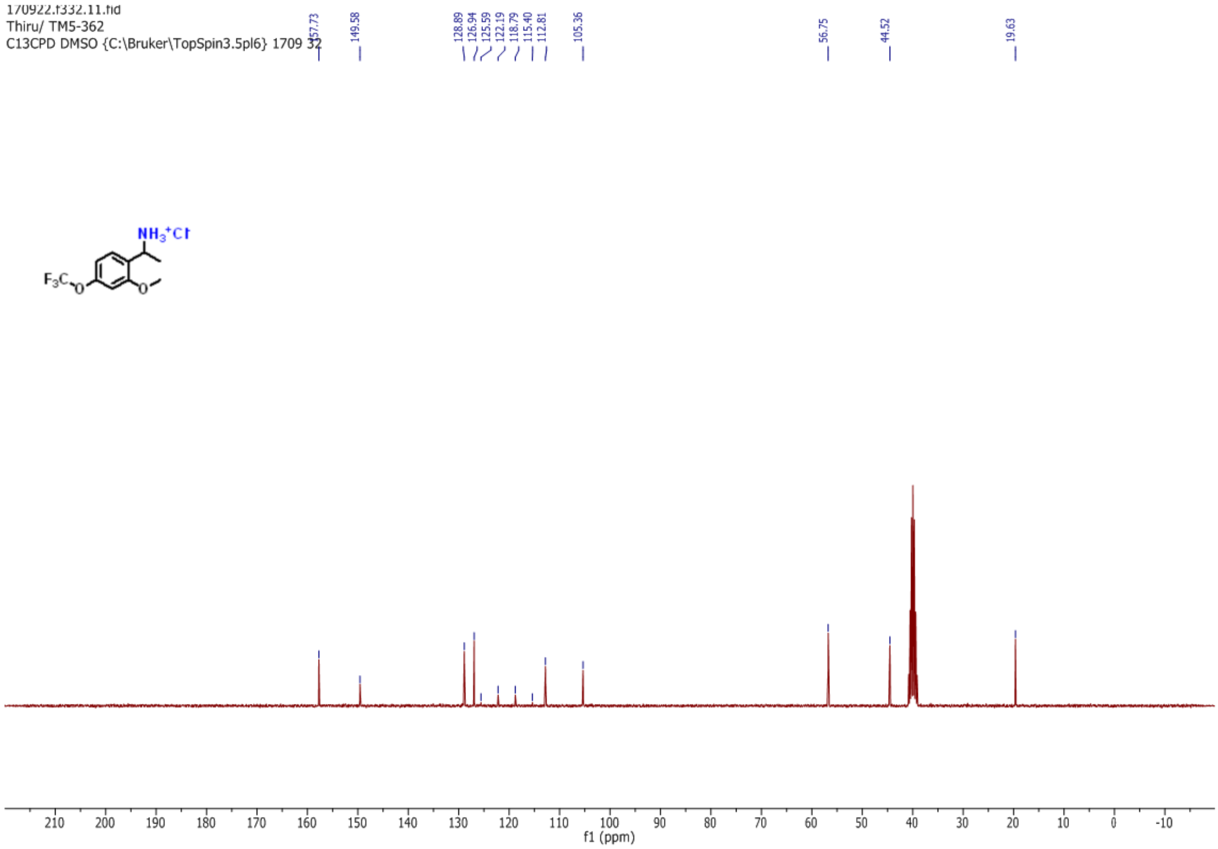

Supplementary Figure 90. ^{13}C NMR spectrum

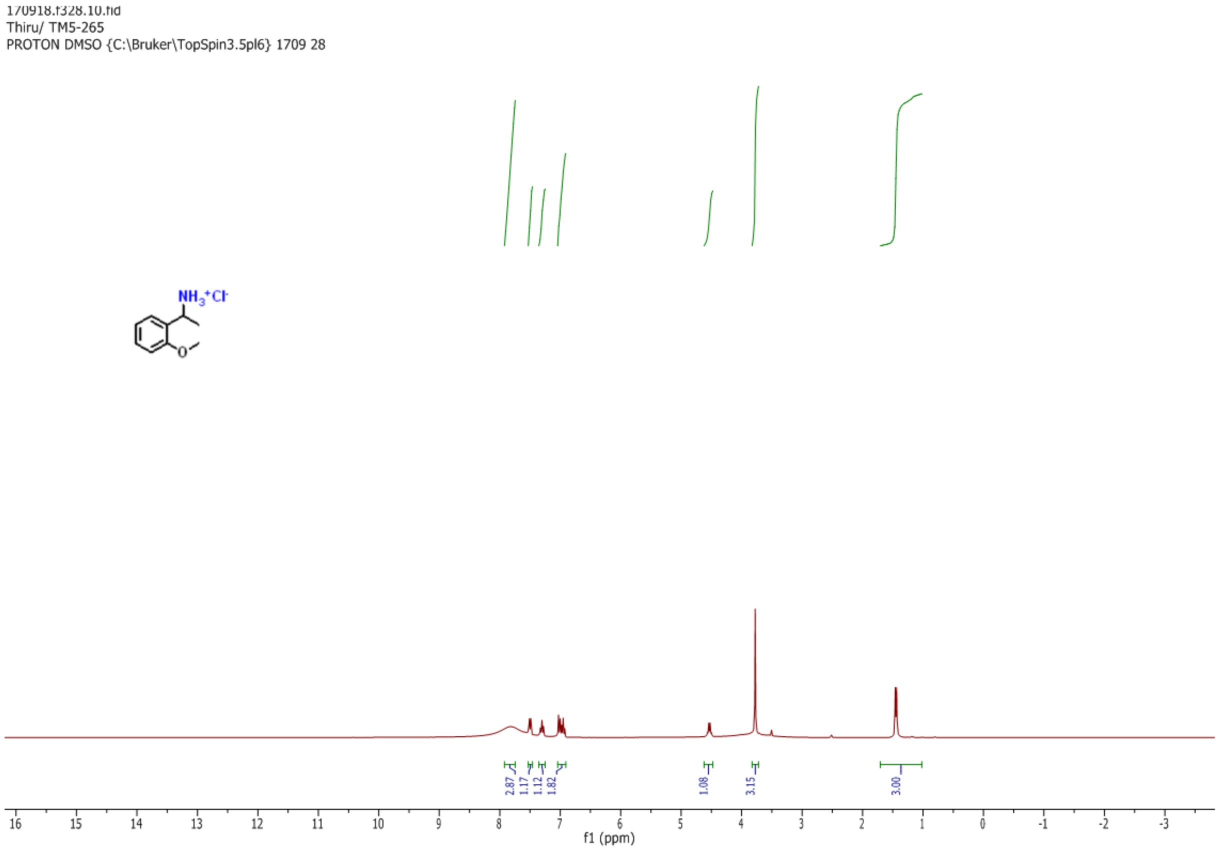

Supplementary Figure 91. ^{1}H NMR spectrum

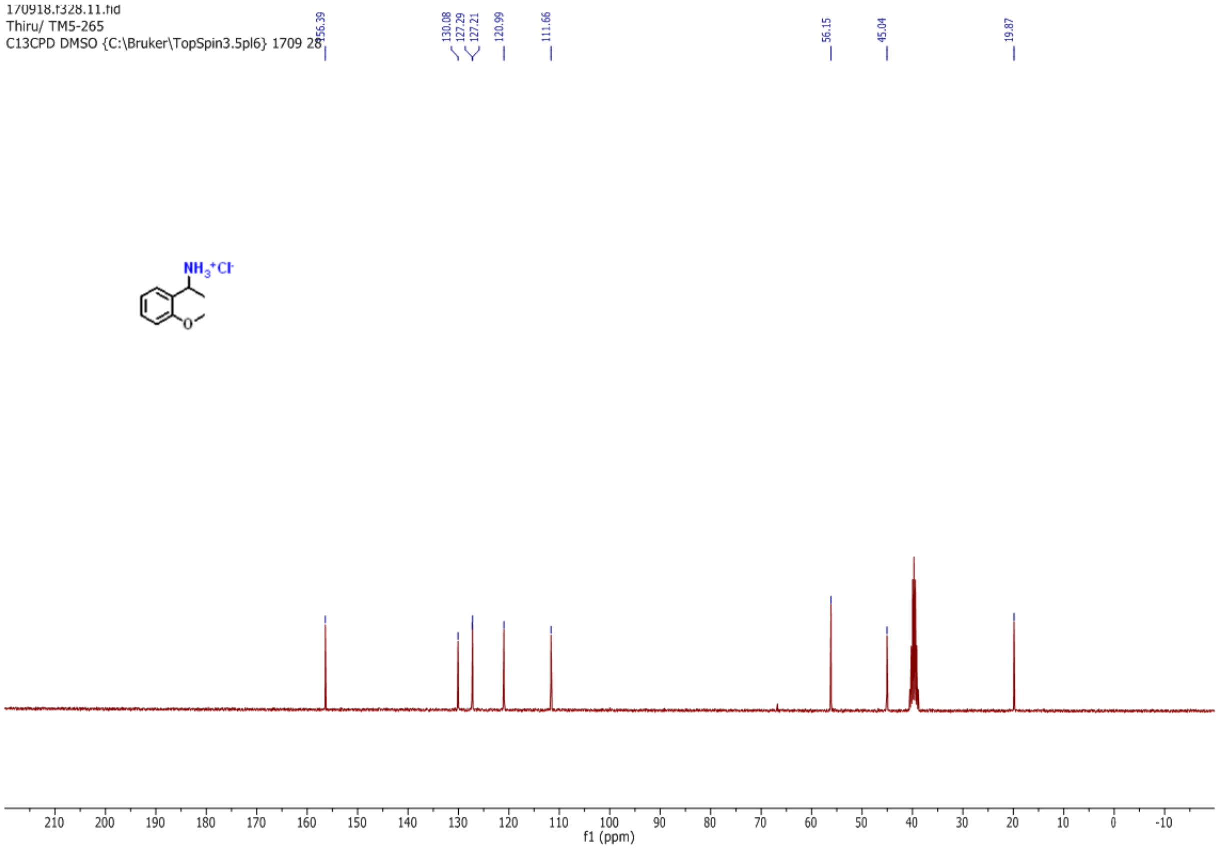

Supplementary Figure 92. ^{13}C NMR spectrum

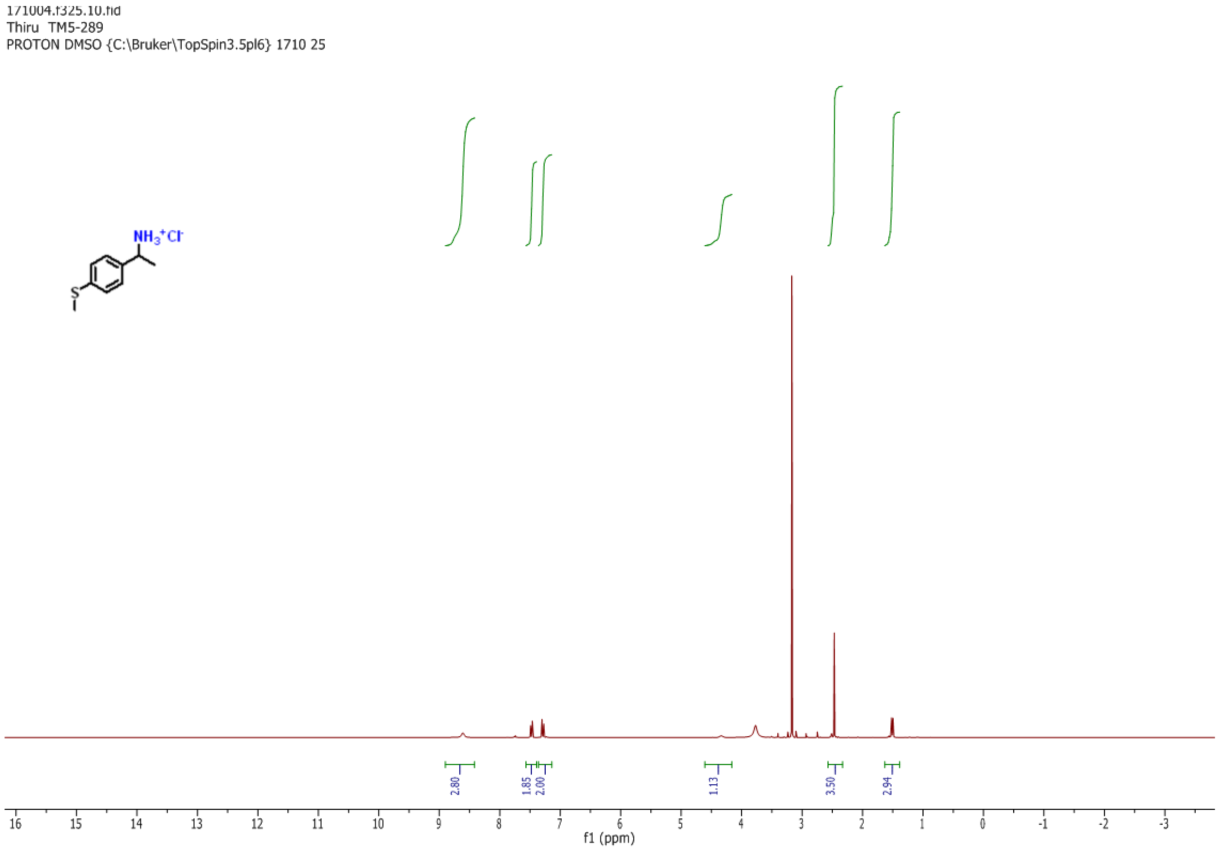

Supplementary Figure 93. ^{1}H NMR spectrum

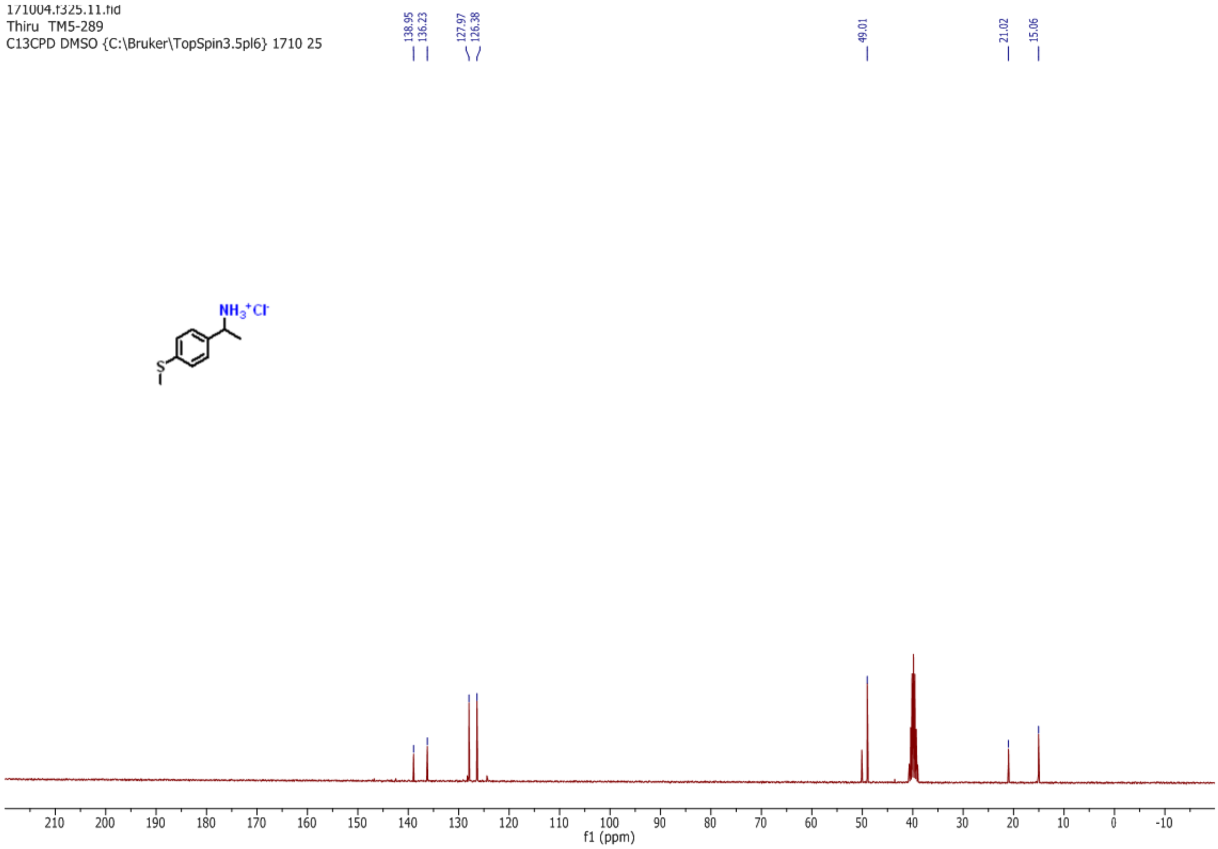

Supplementary Figure 94. ^{13}C NMR spectrum

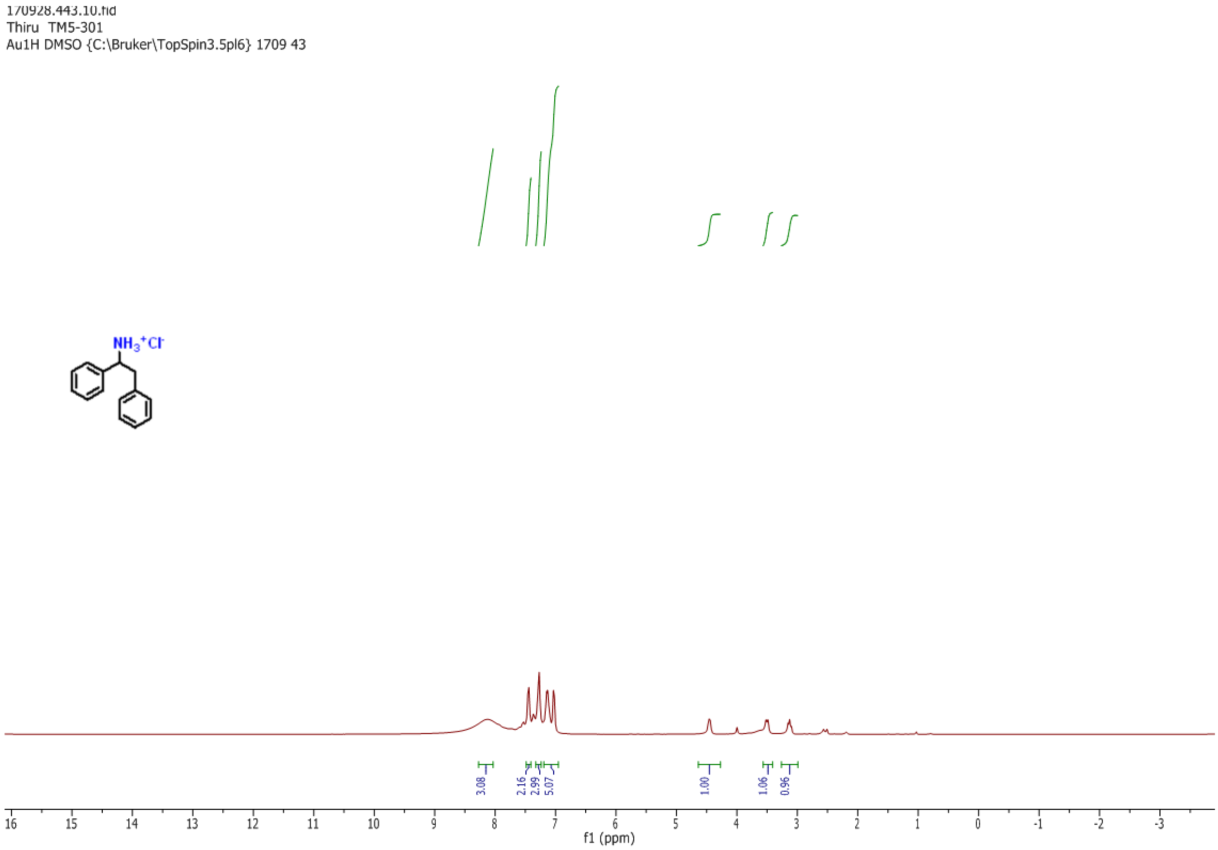

Supplementary Figure 95. ^{1}H NMR spectrum

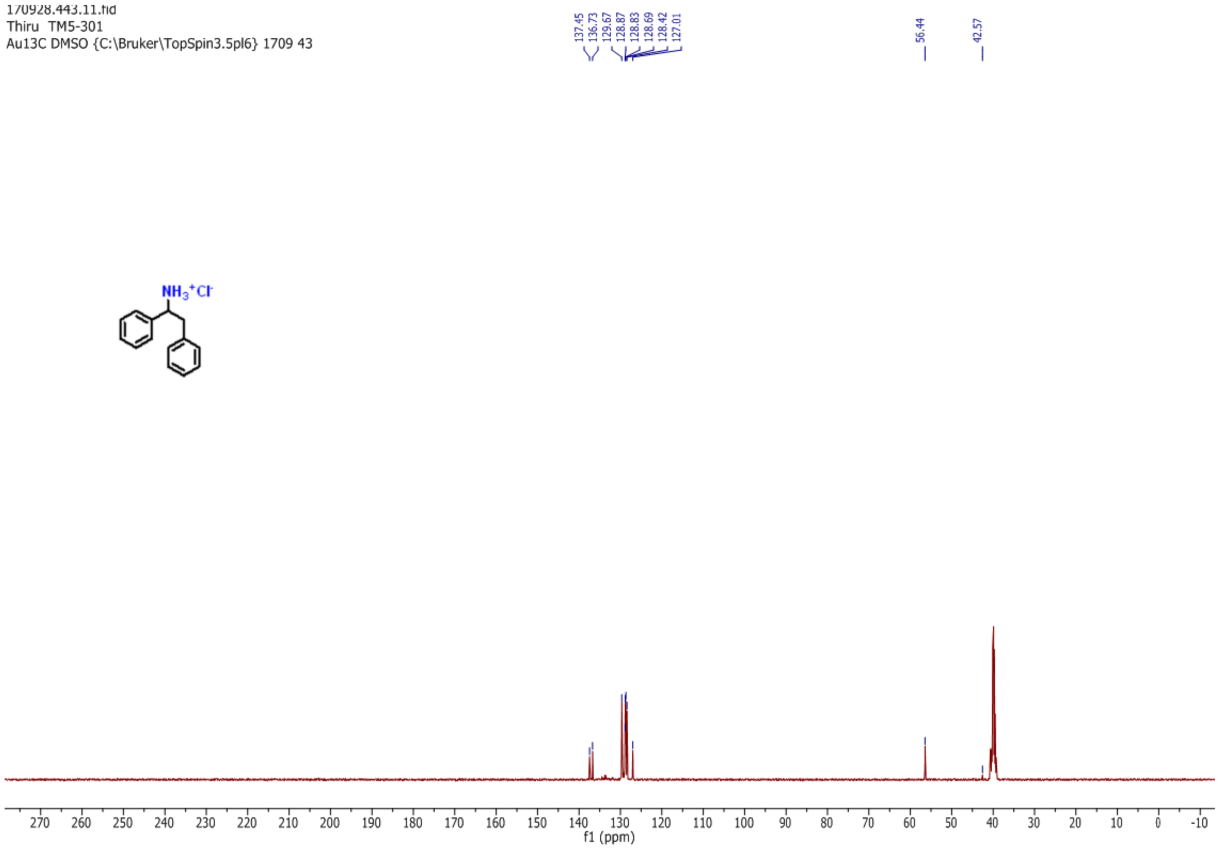

Supplementary Figure 96. ^{13}C NMR spectrum

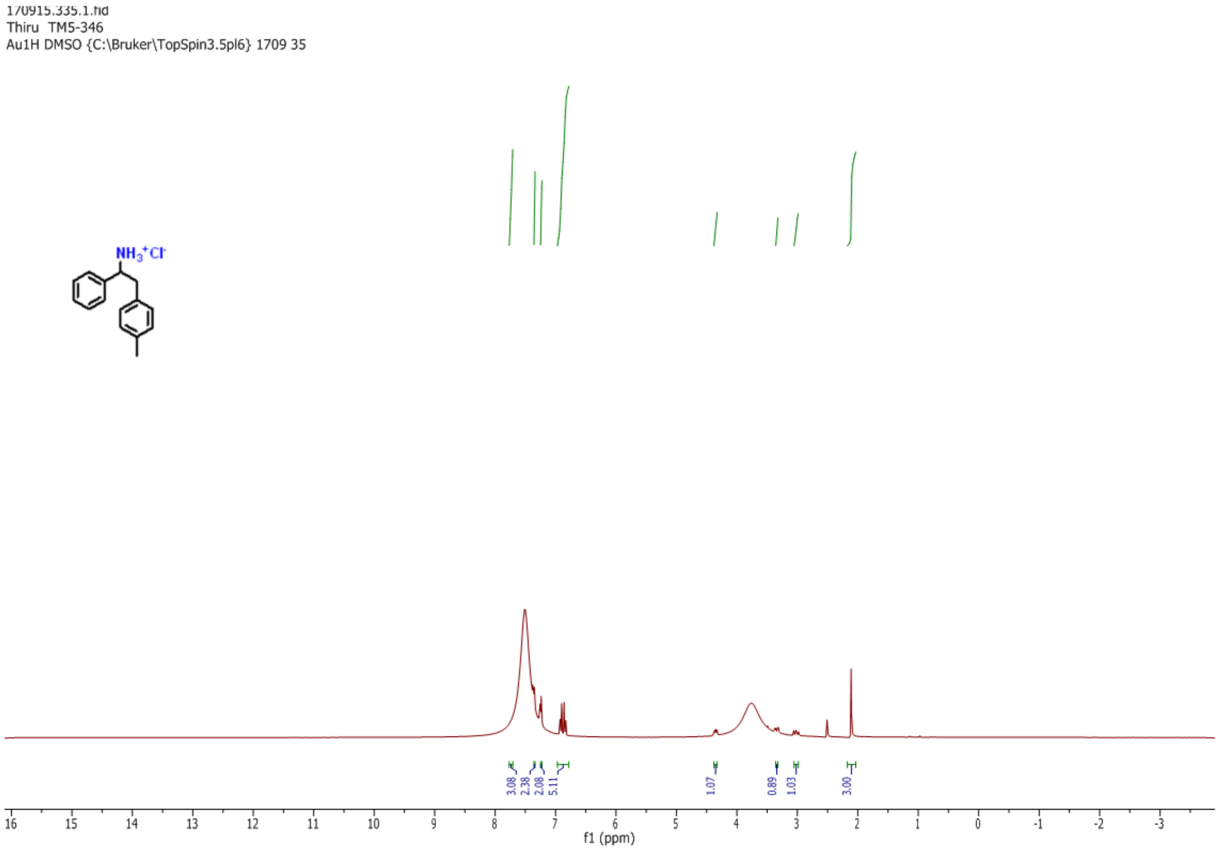

Supplementary Figure 97. ^{1}H NMR spectrum

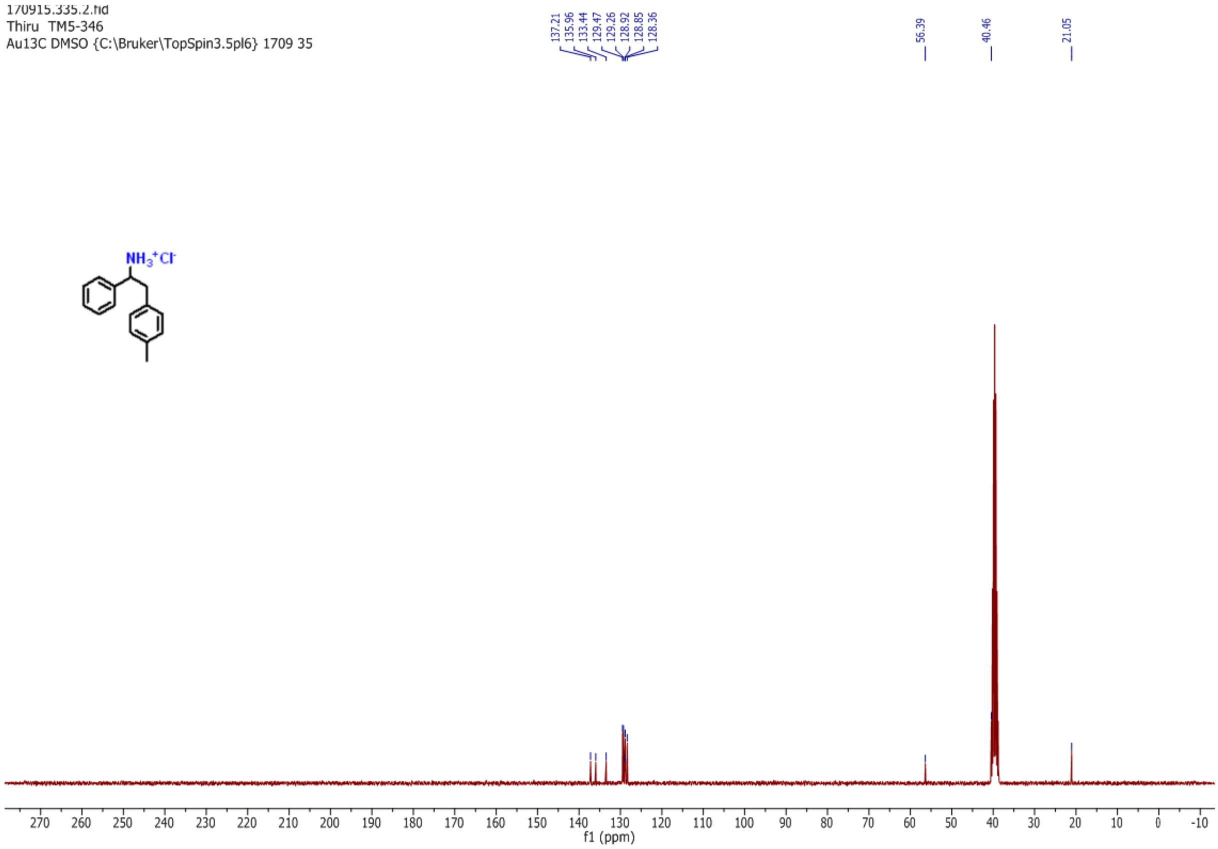

Supplementary Figure 98. ^{13}C NMR spectrum

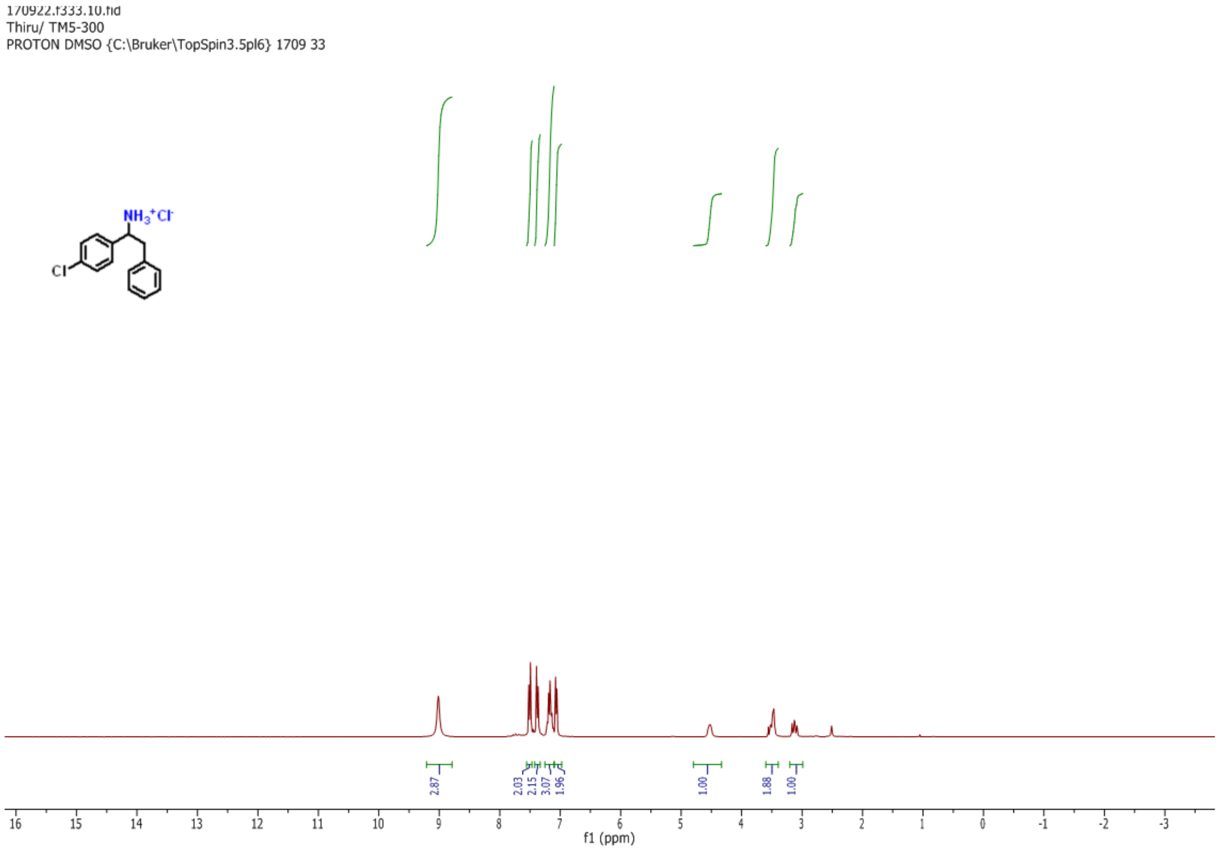

Supplementary Figure 99. ^{1}H NMR spectrum

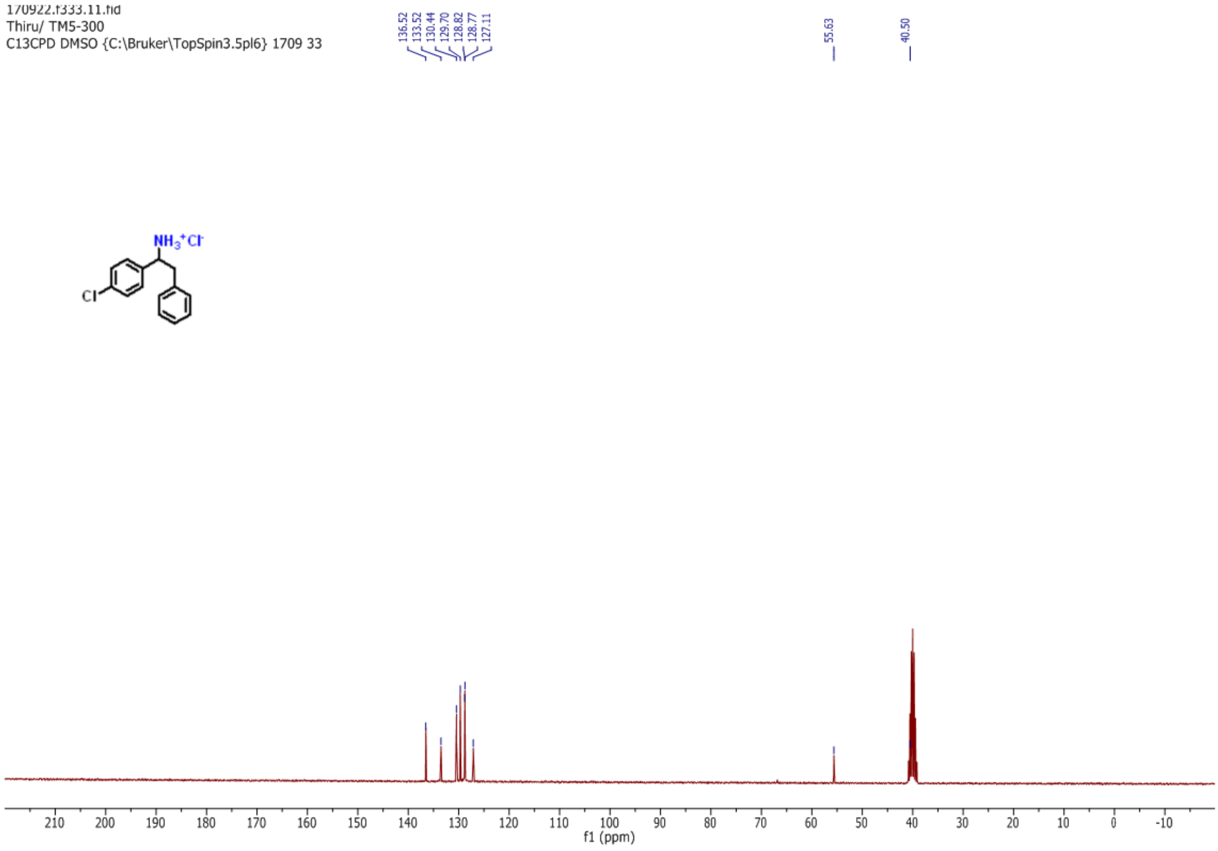

Supplementary Figure 100. ^{13}C NMR spectrum

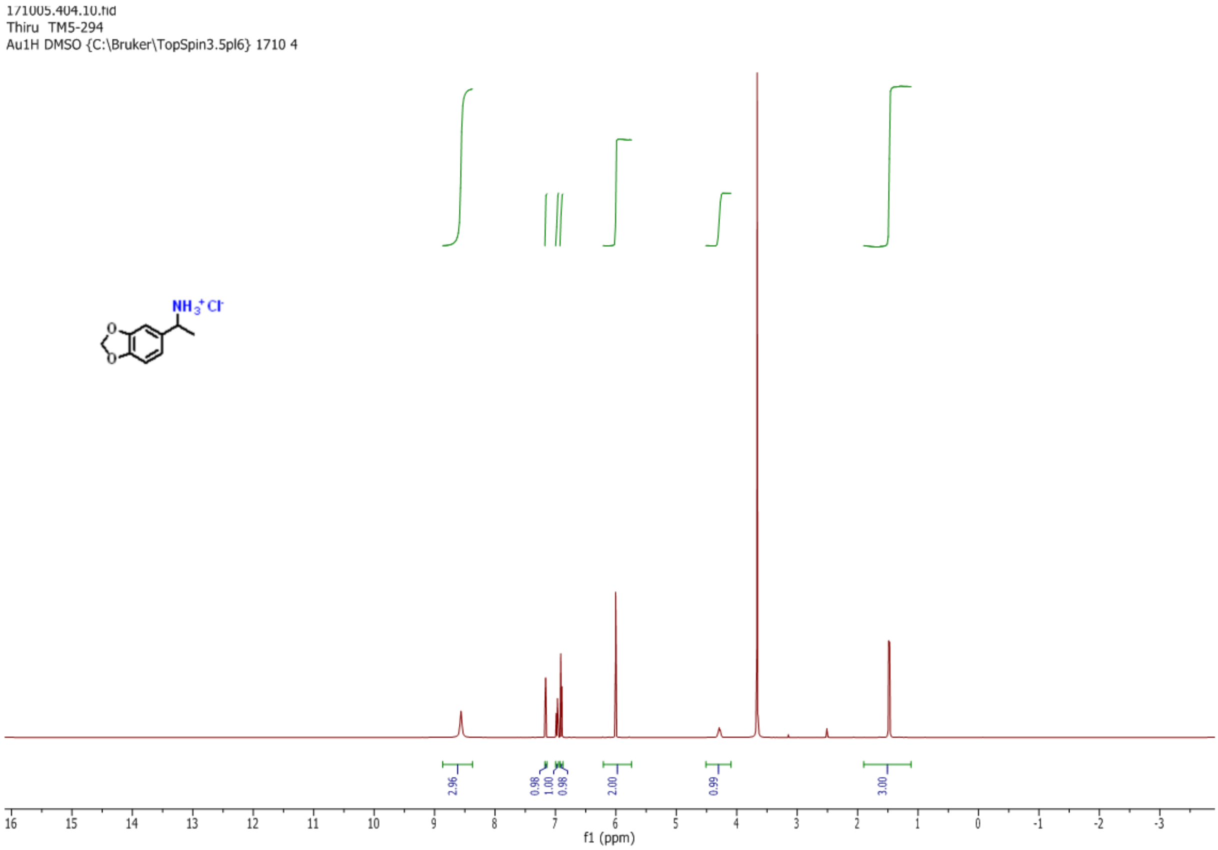

Supplementary Figure 101. ^{1}H NMR spectrum

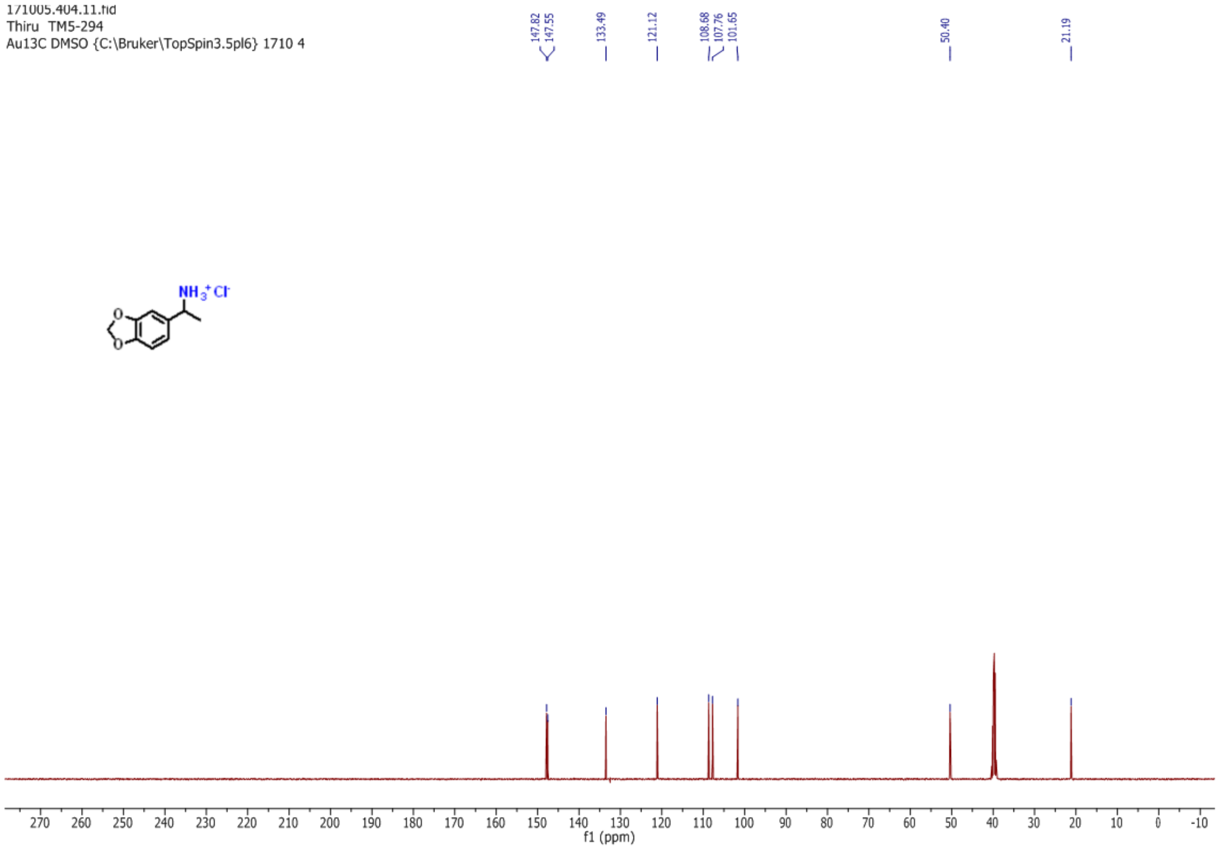

Supplementary Figure 102. ^{13}C NMR spectrum

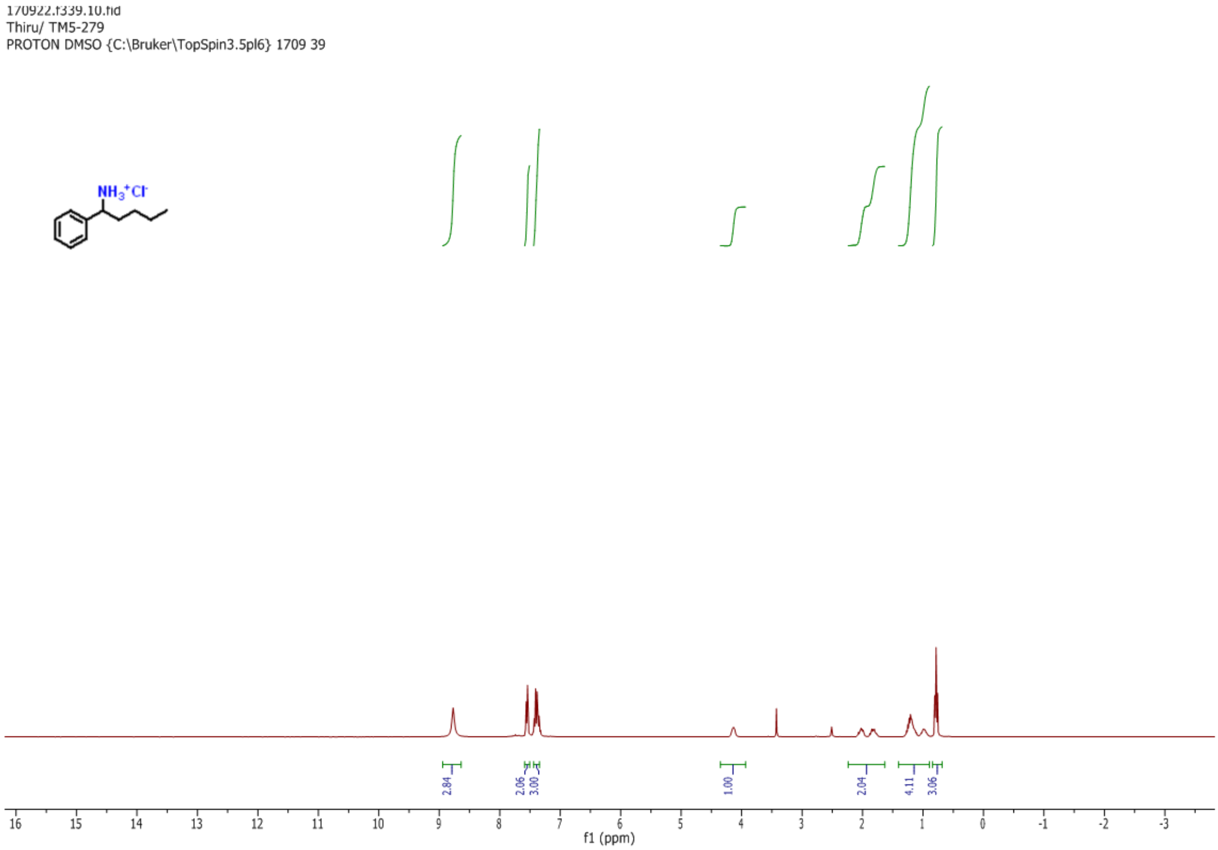

Supplementary Figure 103. ^{1}H NMR spectrum

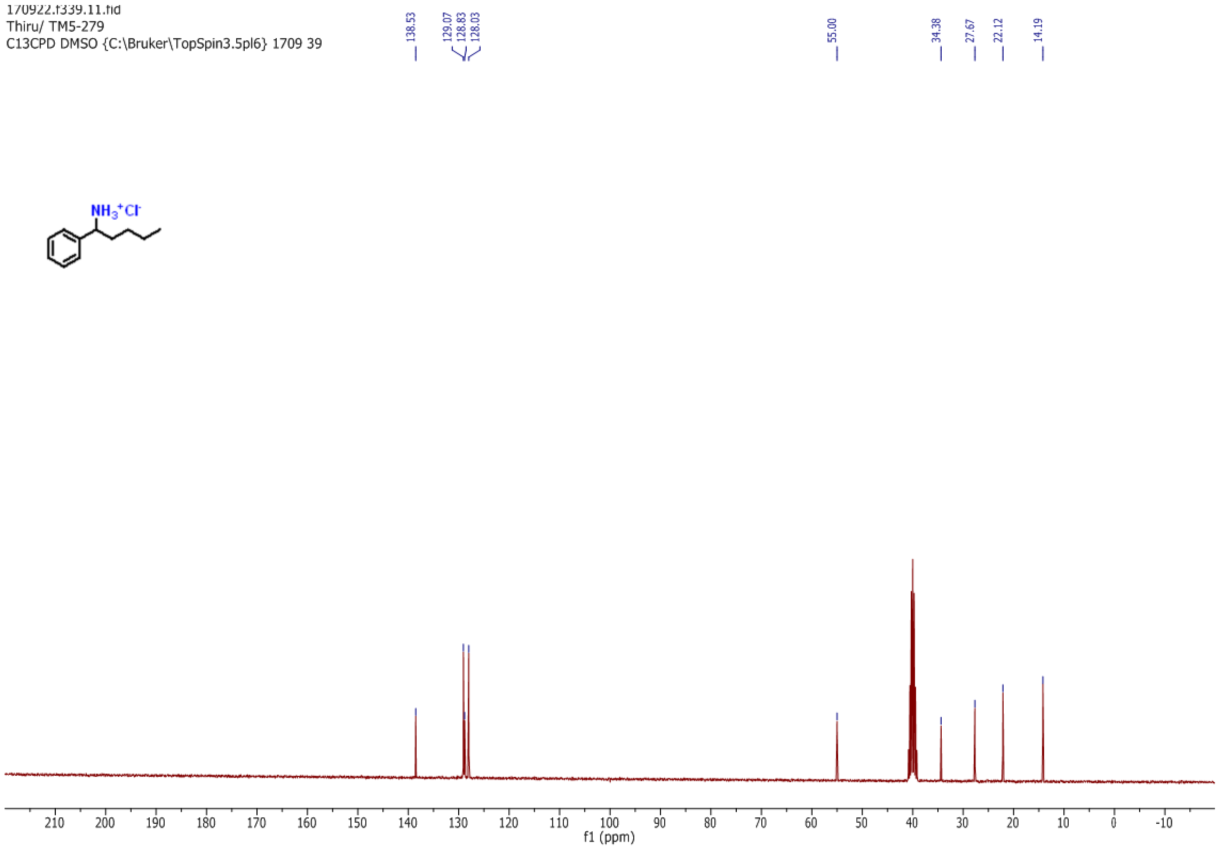

Supplementary Figure 104. ^{13}C NMR spectrum

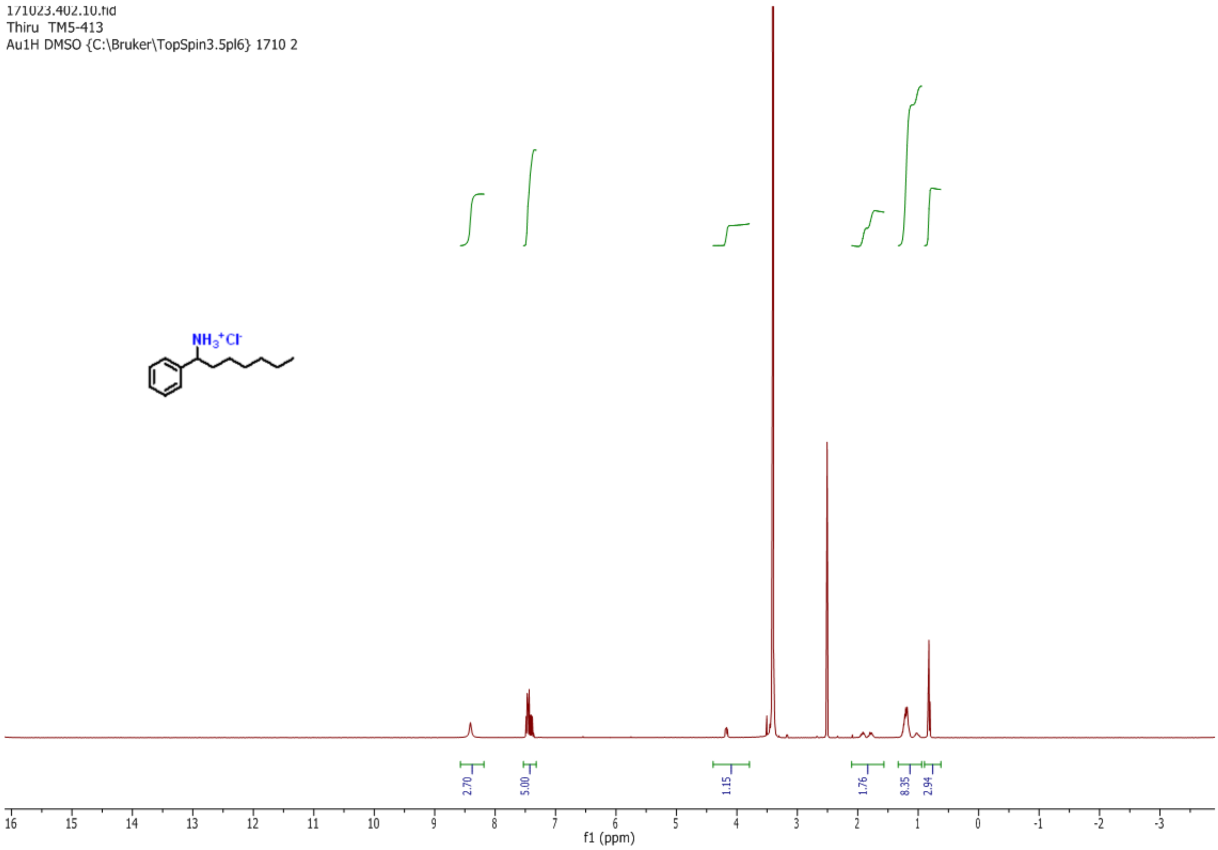

Supplementary Figure 105. ^{1}H NMR spectrum

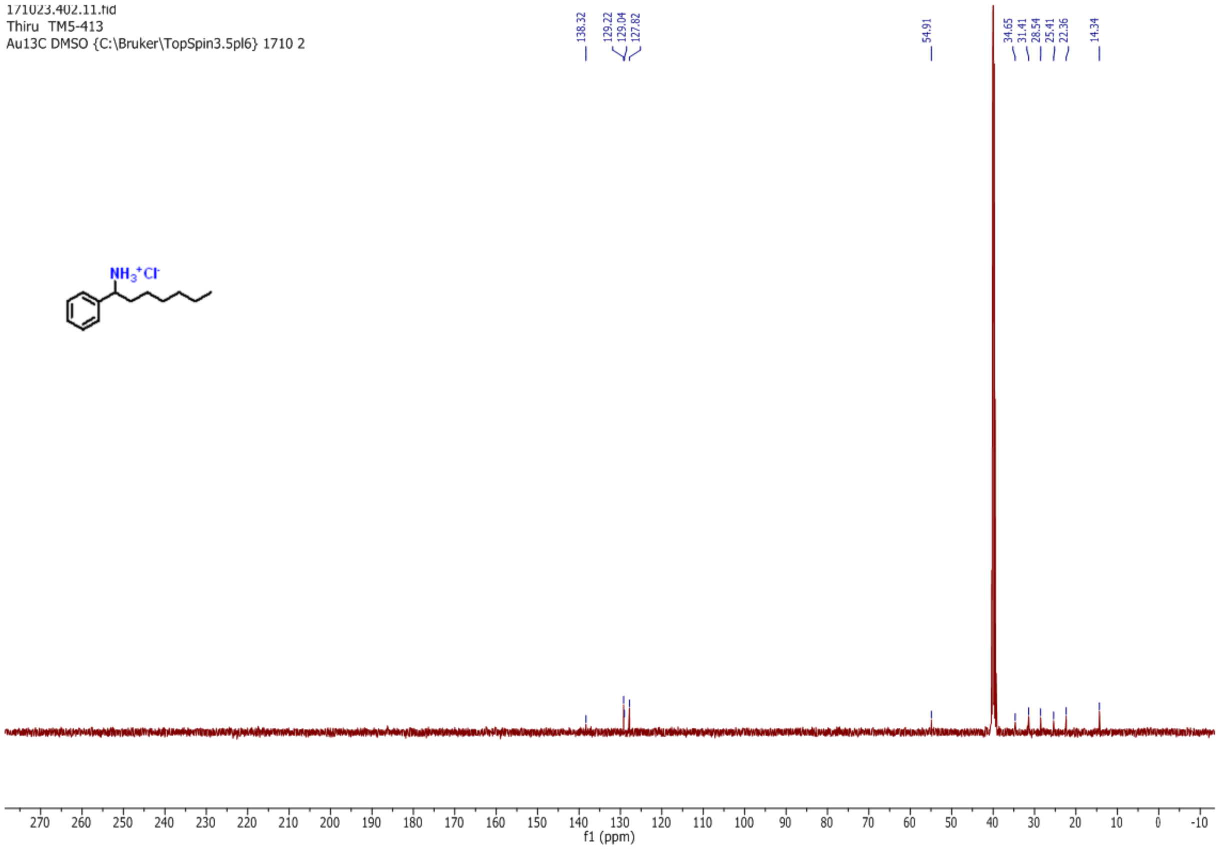

Supplementary Figure 106. ^{13}C NMR spectrum

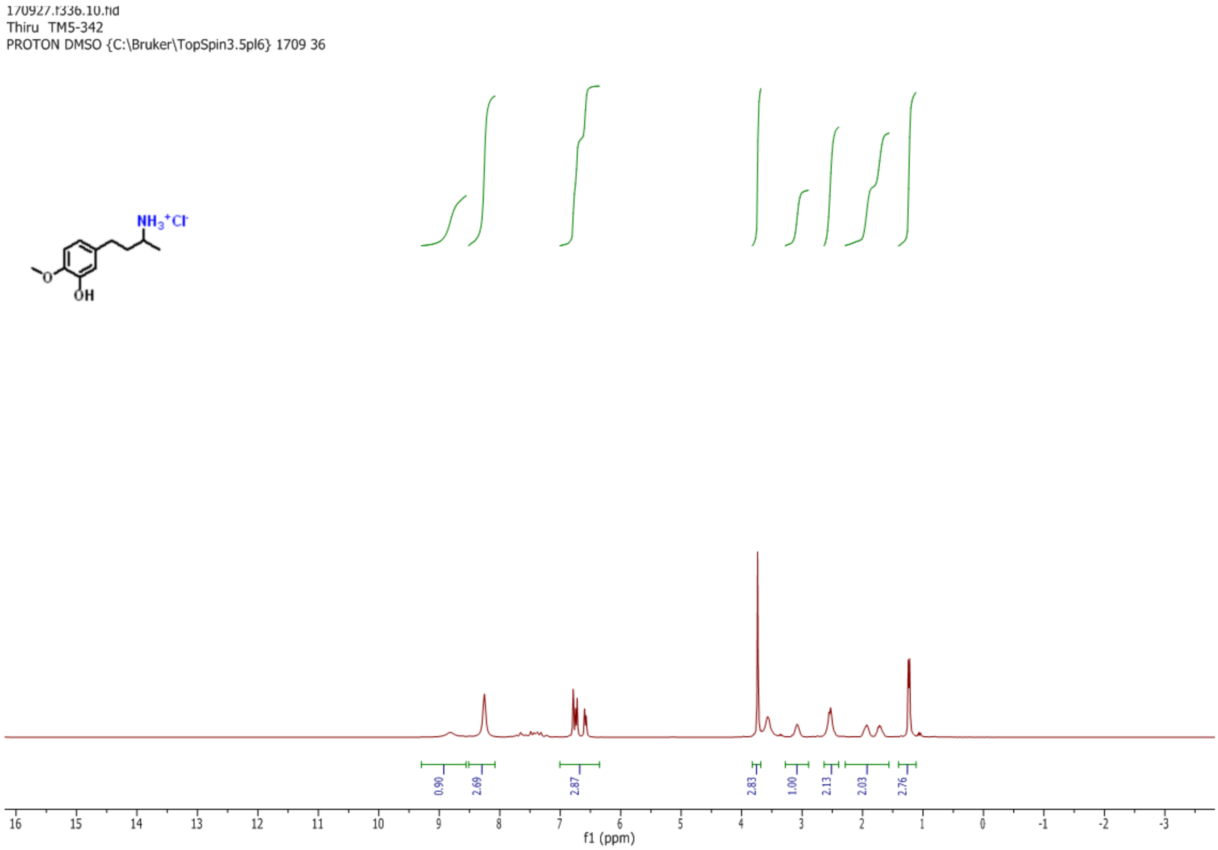

Supplementary Figure 107. ^{1}H NMR spectrum

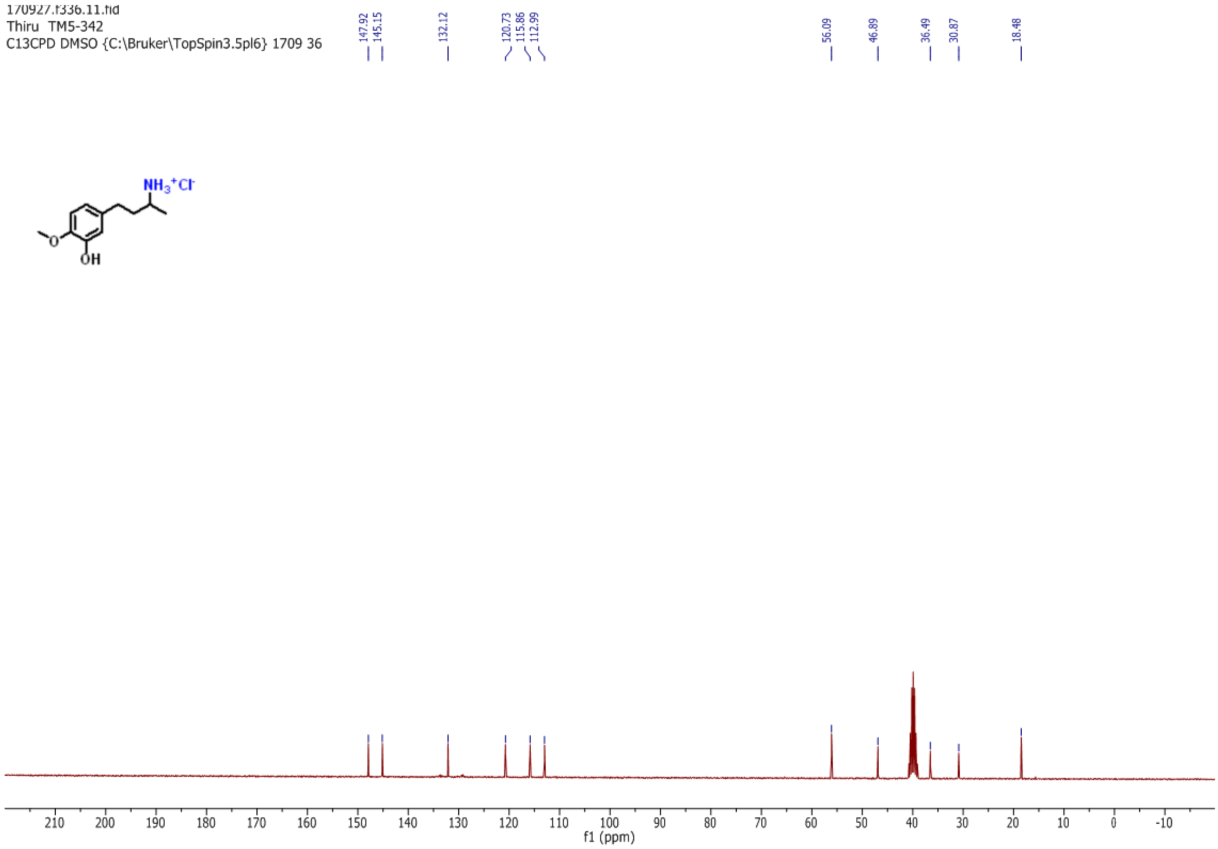

Supplementary Figure 108. ^{13}C NMR spectrum

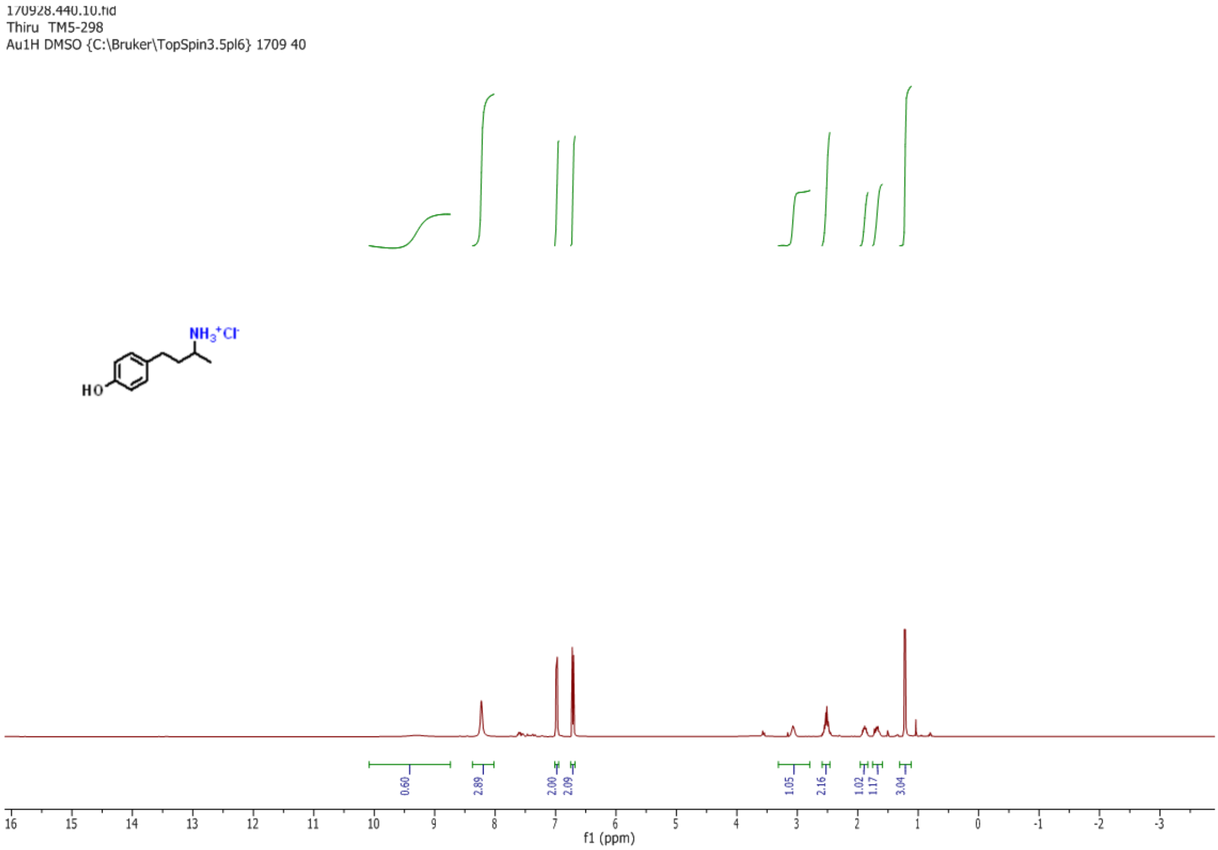

Supplementary Figure 109. ^{1}H NMR spectrum

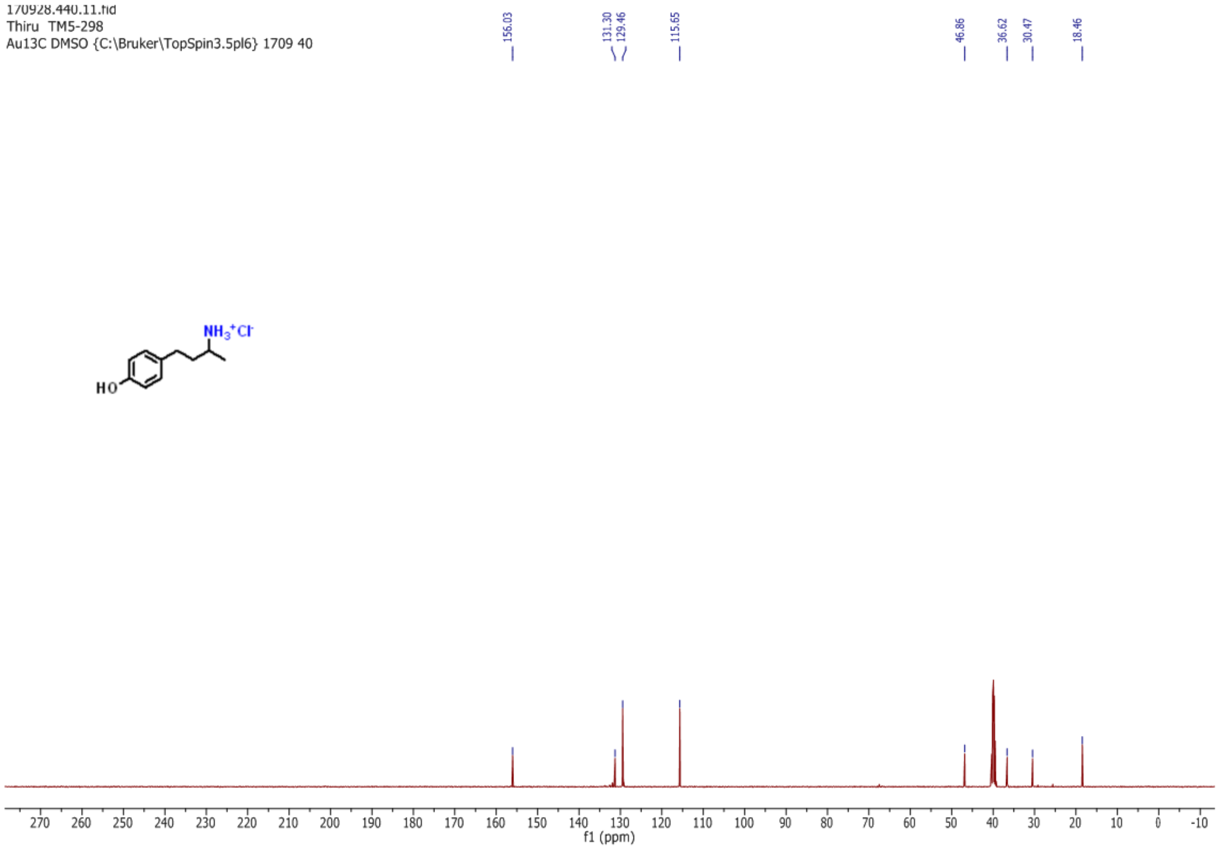

Supplementary Figure 110. ^{13}C NMR spectrum

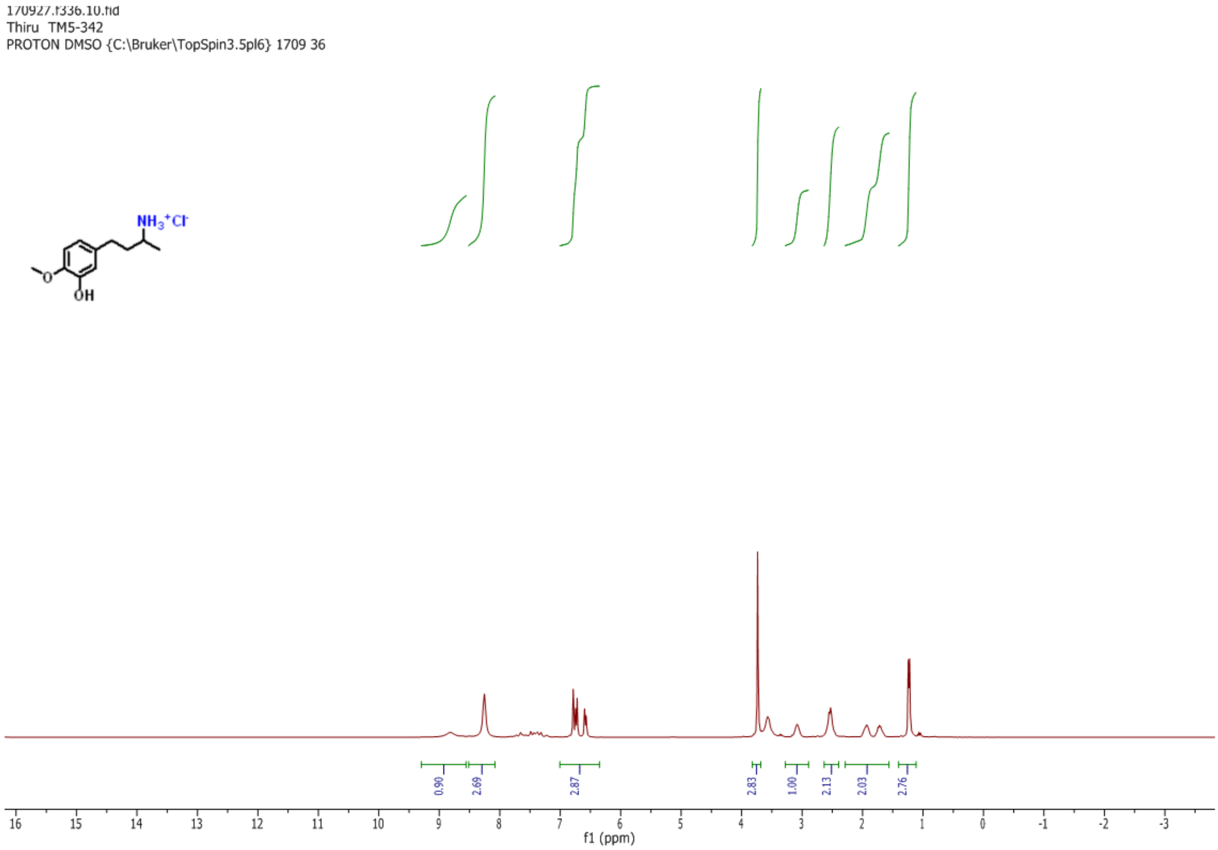

Supplementary Figure 111. ^{1}H NMR spectrum

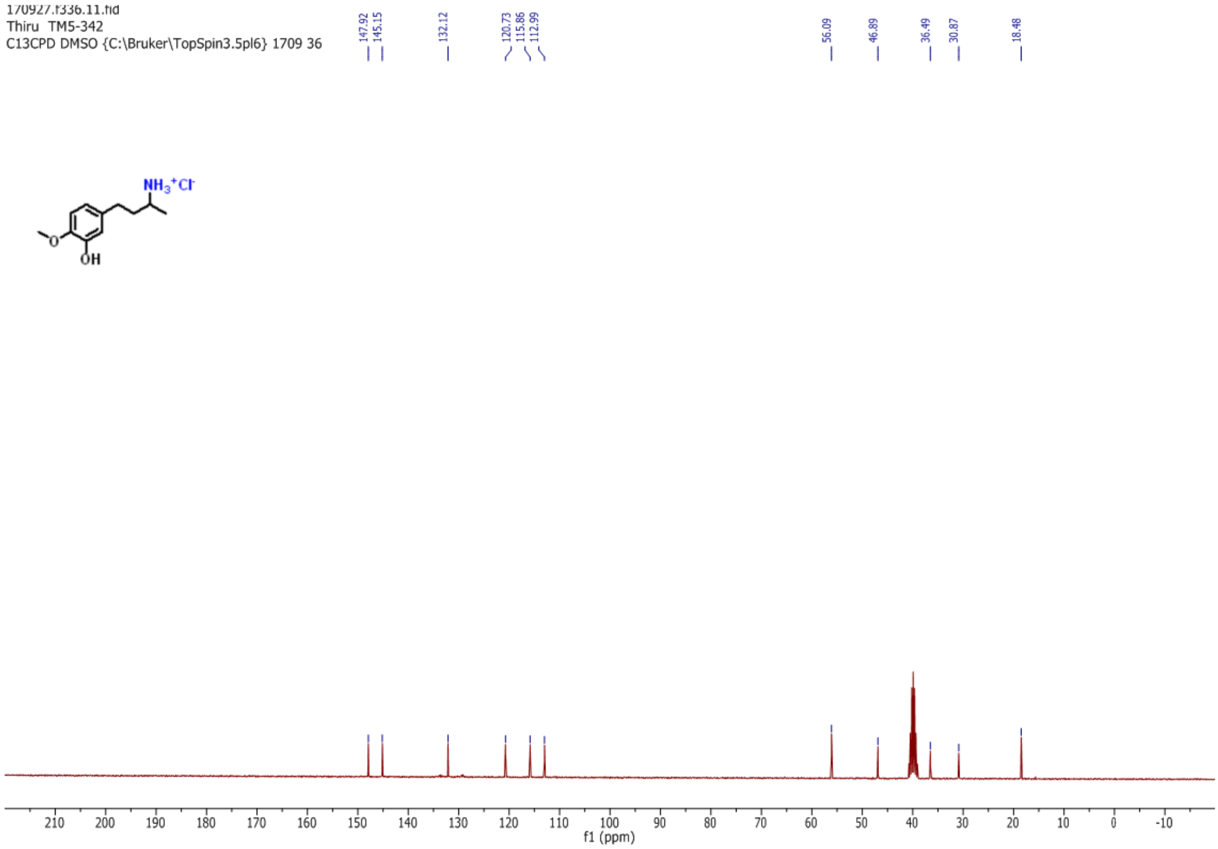

Supplementary Figure 112. ^{13}C NMR spectrum

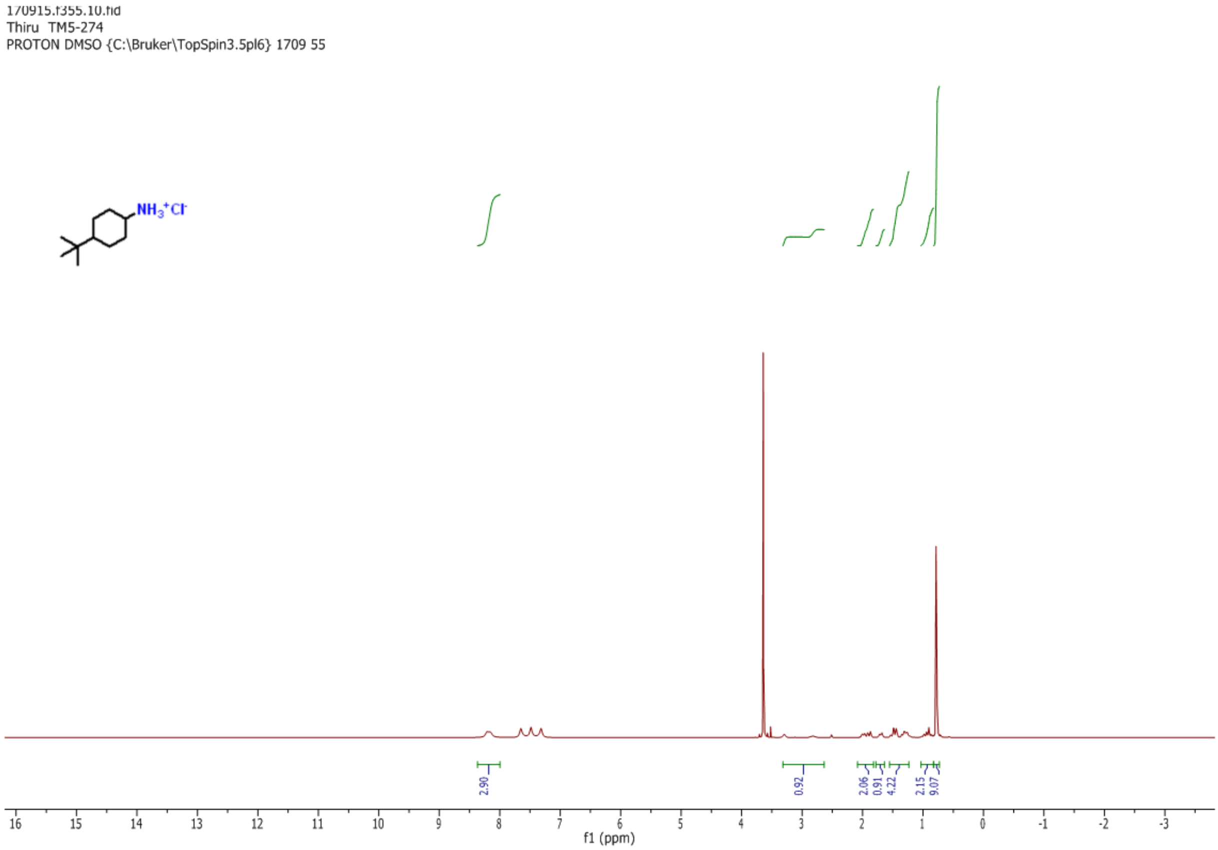

Supplementary Figure 113. ^{1}H NMR spectrum

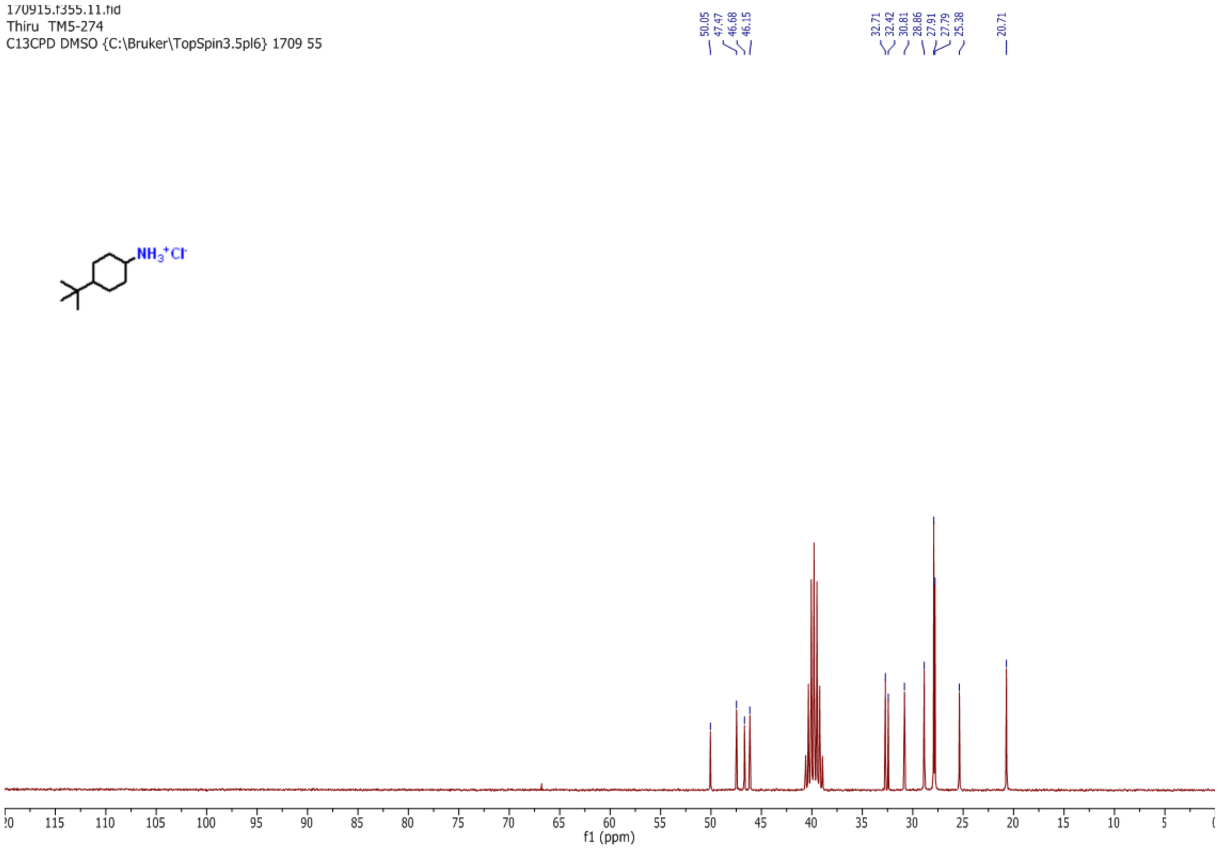

Supplementary Figure 114. ^{13}C NMR spectrum

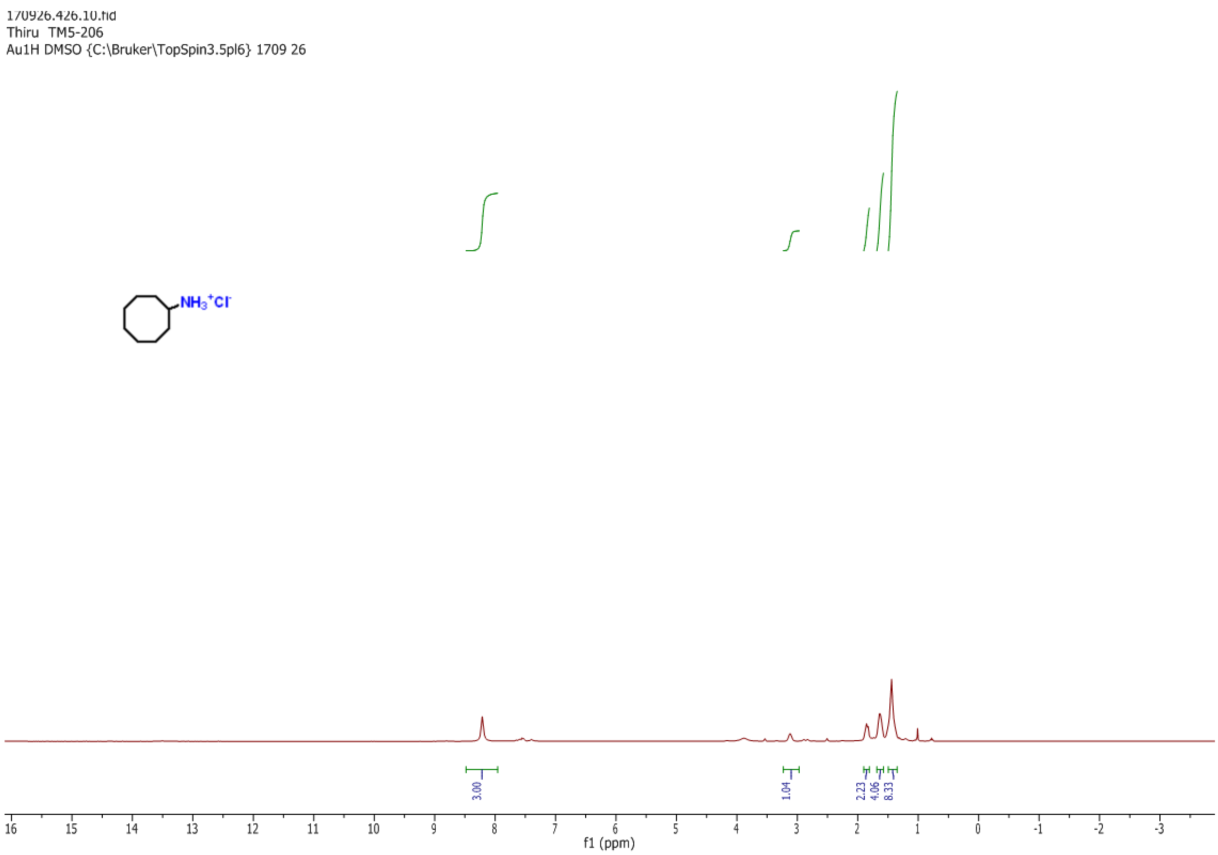

Supplementary Figure 115. ^{1}H NMR spectrum

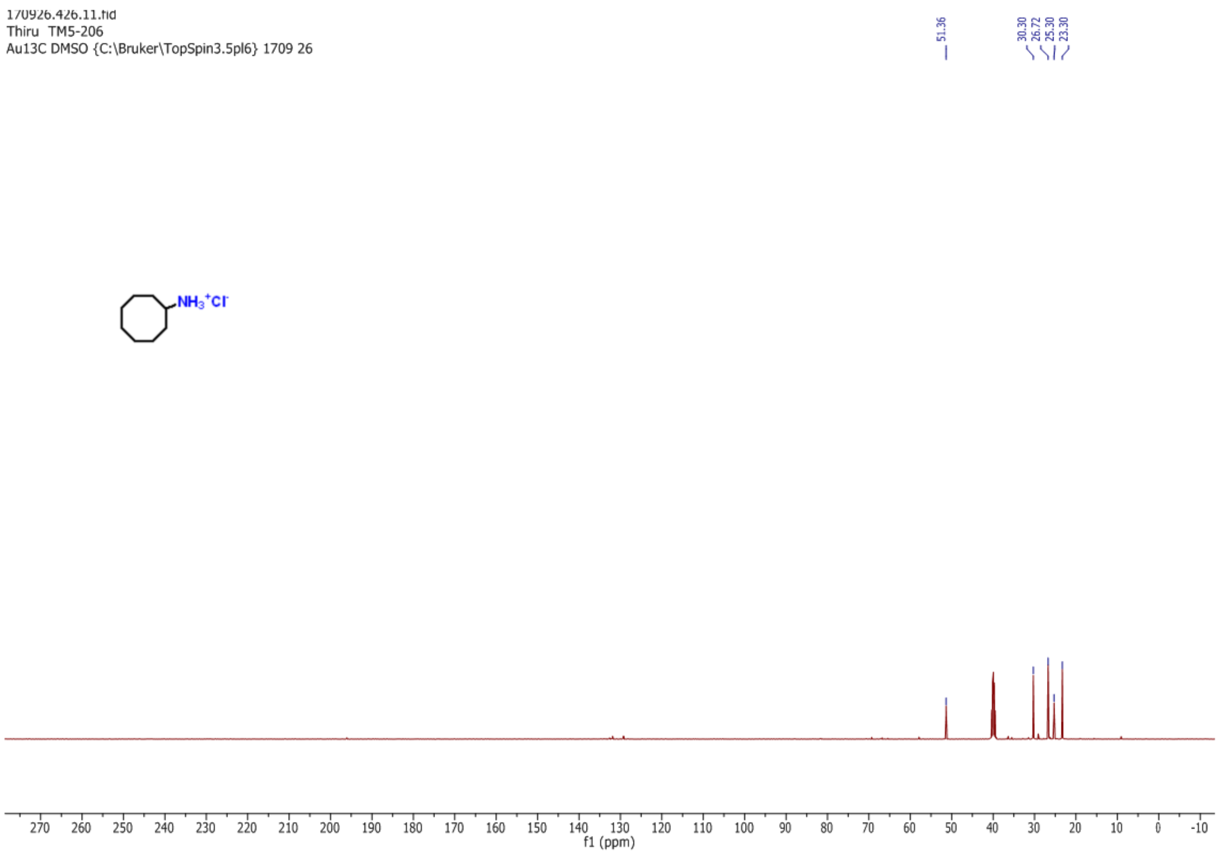

Supplementary Figure 116. ^{13}C NMR spectrum

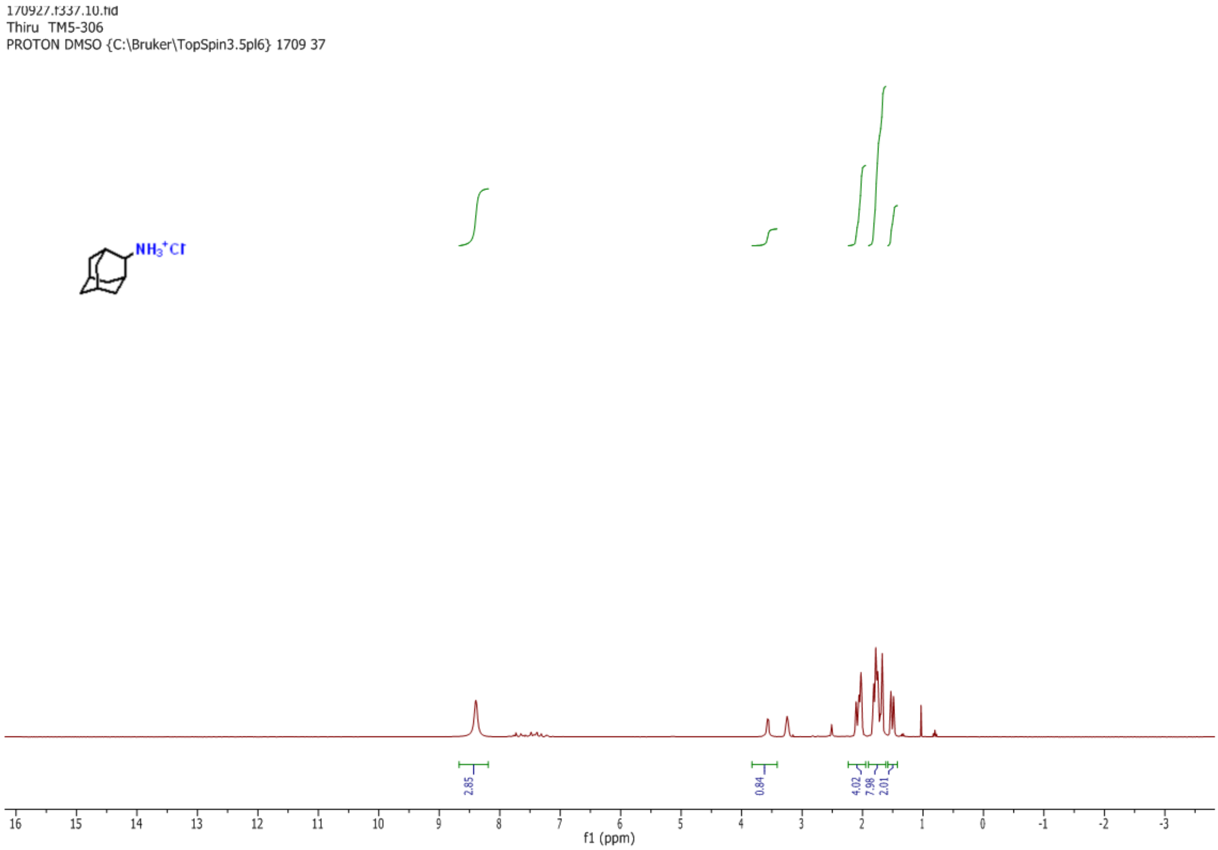

Supplementary Figure 117. ^{1}H NMR spectrum

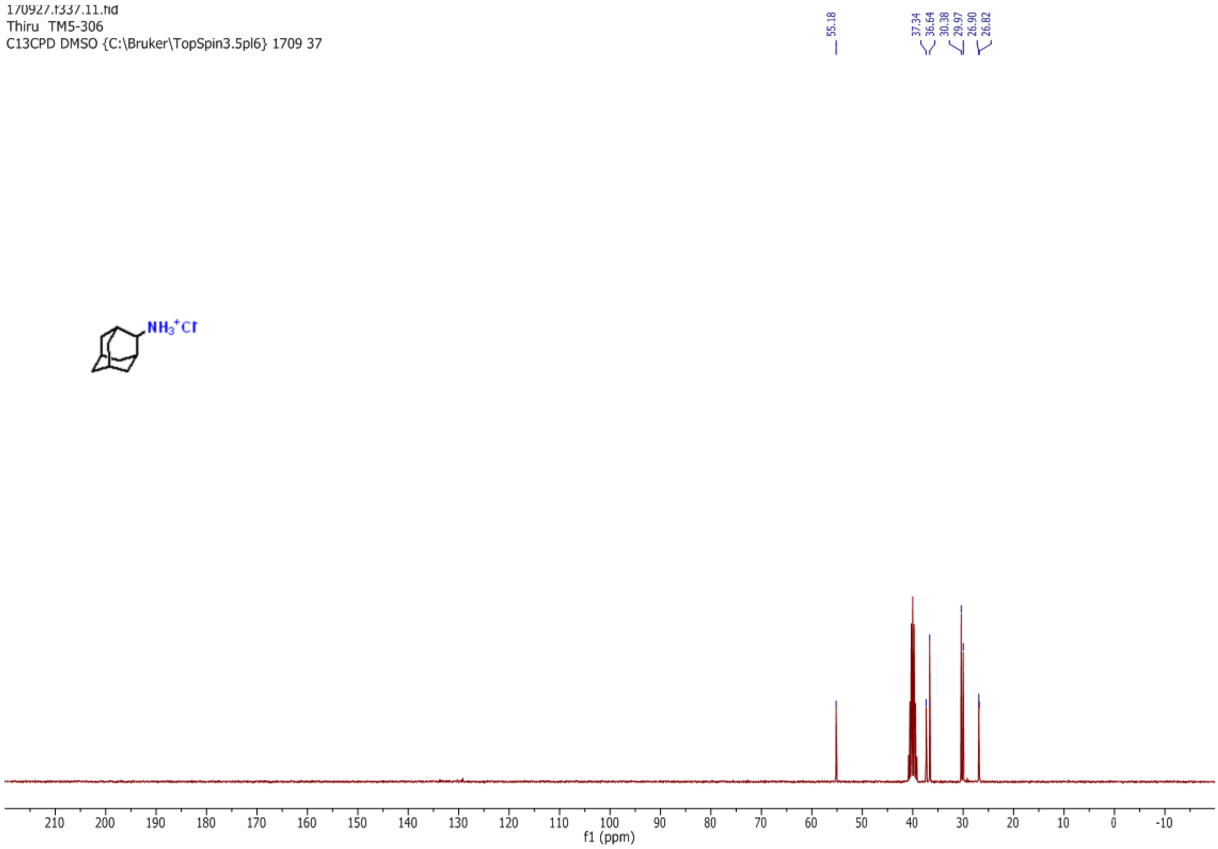

Supplementary Figure 118. ^{13}C NMR spectrum

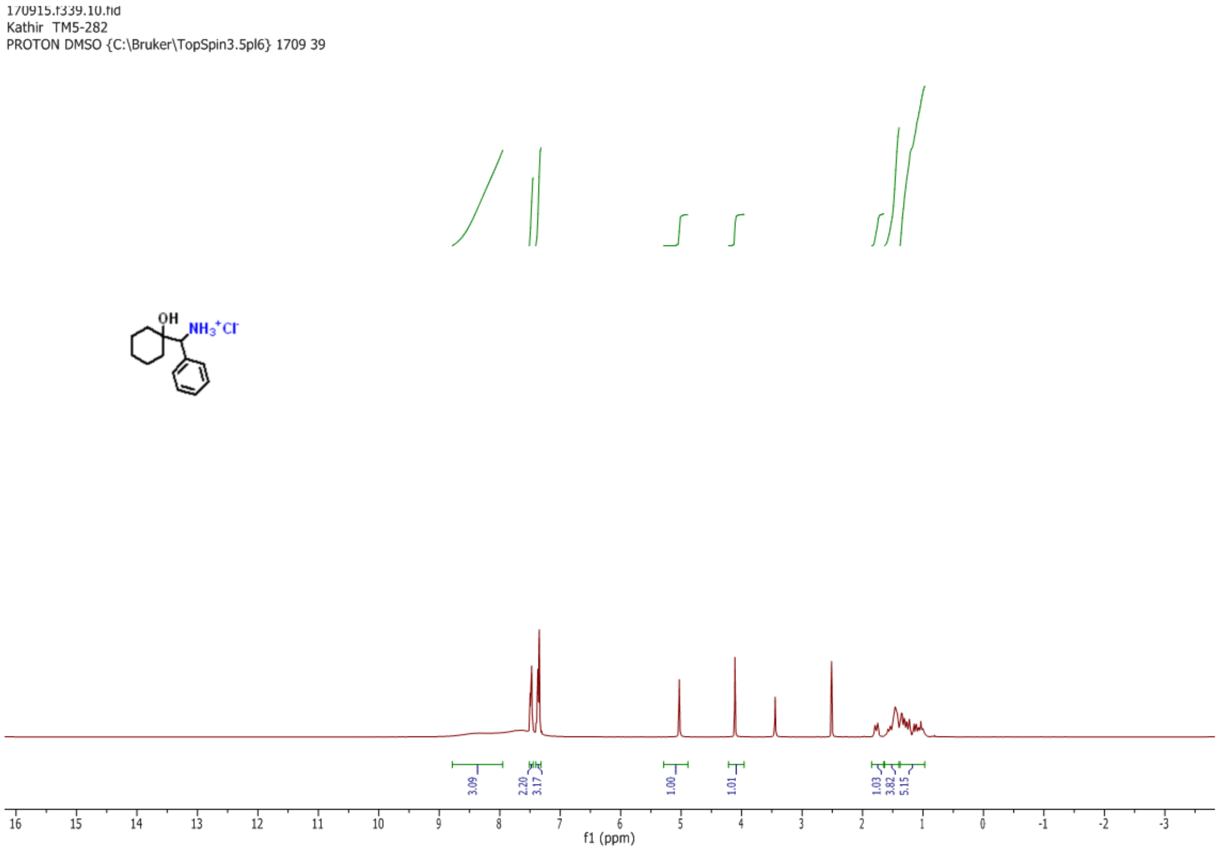

Supplementary Figure 119. ^{1}H NMR spectrum

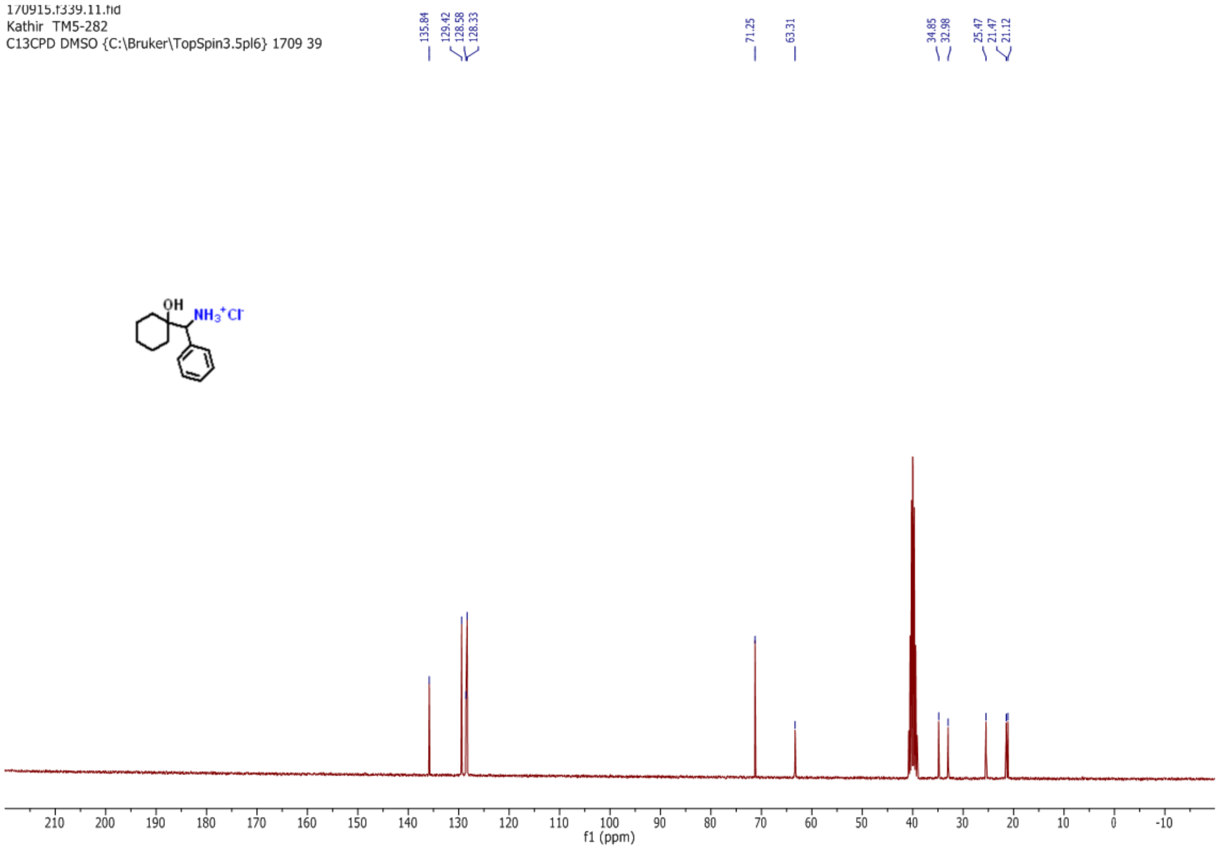

Supplementary Figure 120. ^{13}C NMR spectrum

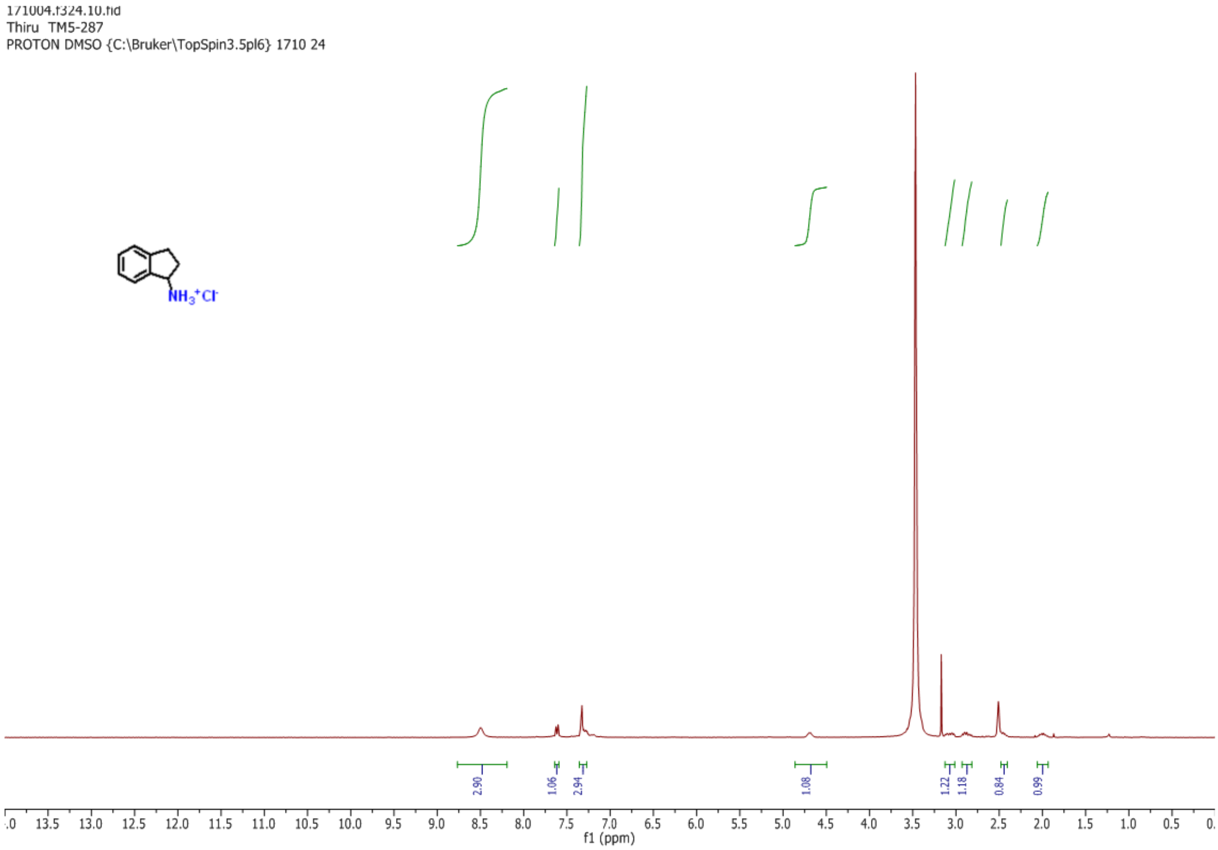

Supplementary Figure 121. ^{1}H NMR spectrum

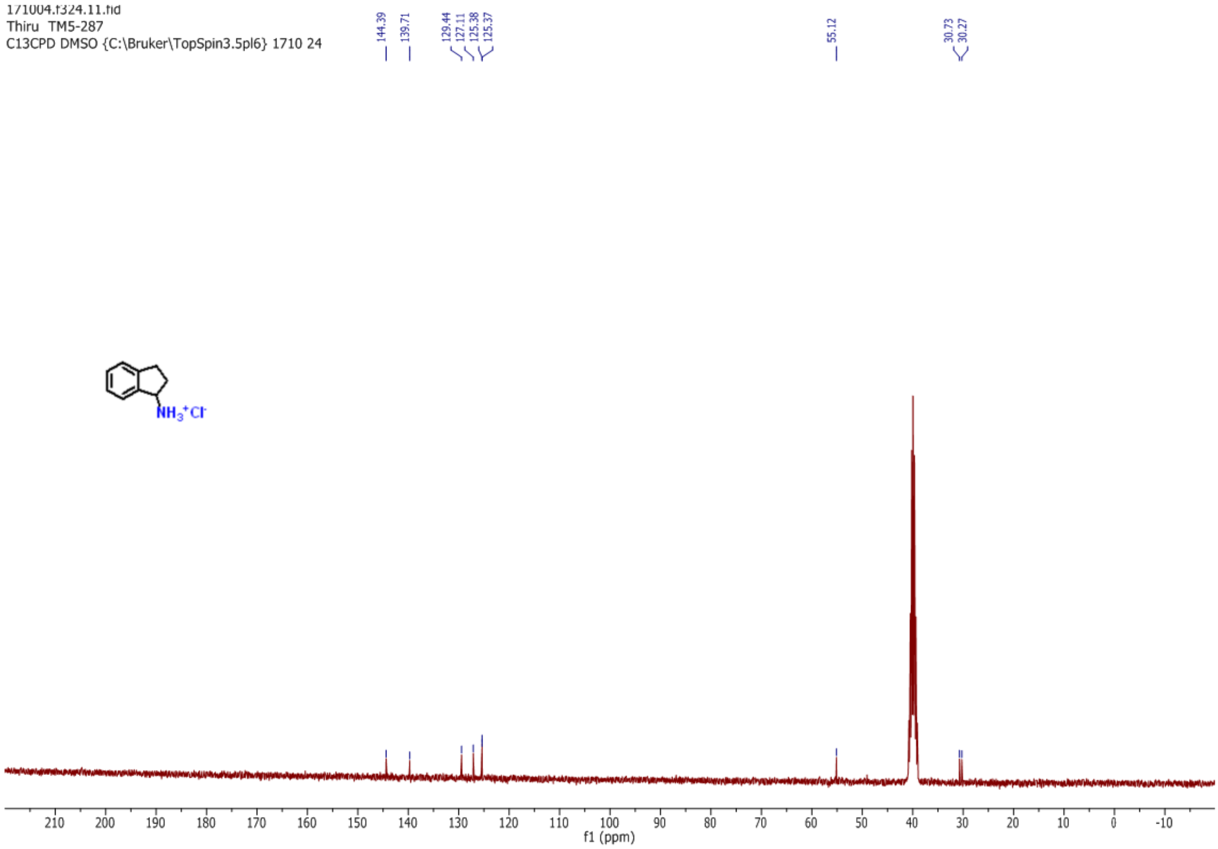

Supplementary Figure 122. ^{13}C NMR spectrum

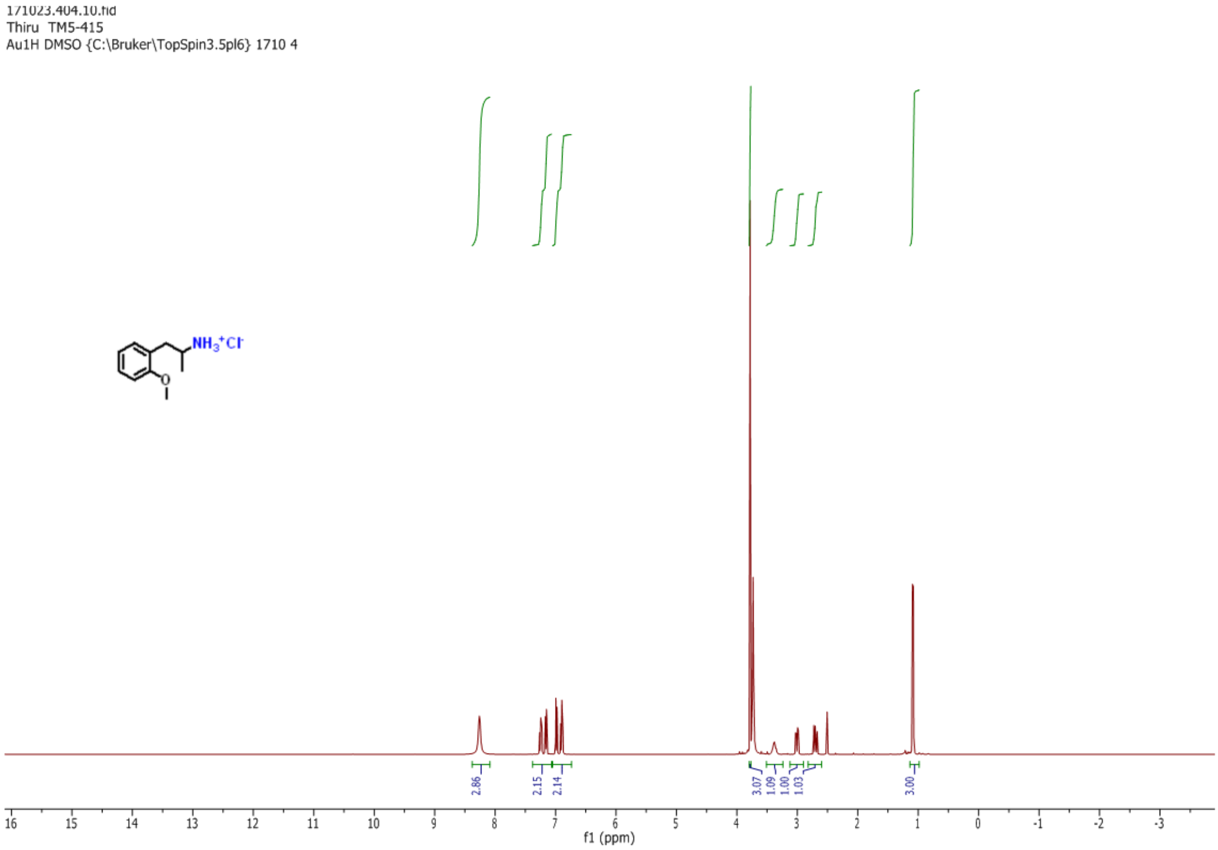

Supplementary Figure 125. ^{1}H NMR spectrum

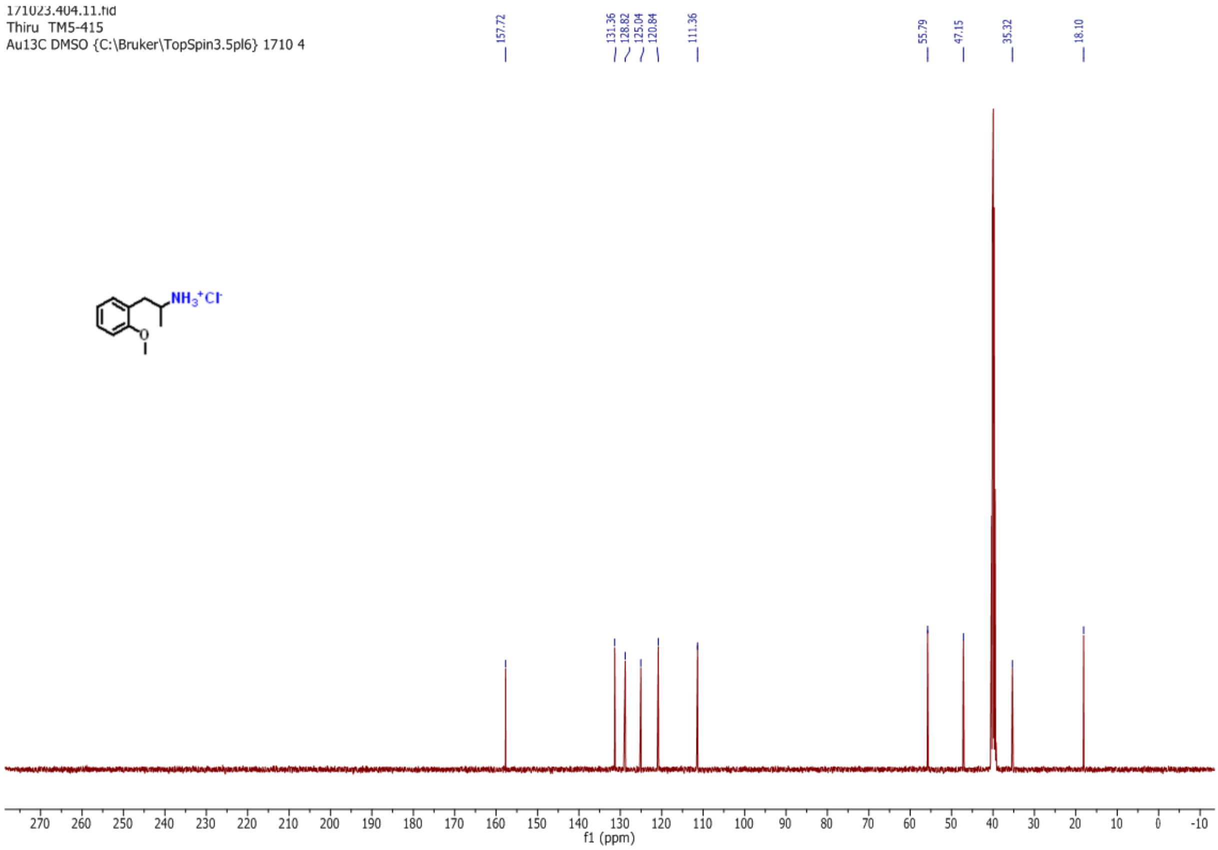

Supplementary Figure 126. ^{13}C NMR spectrum

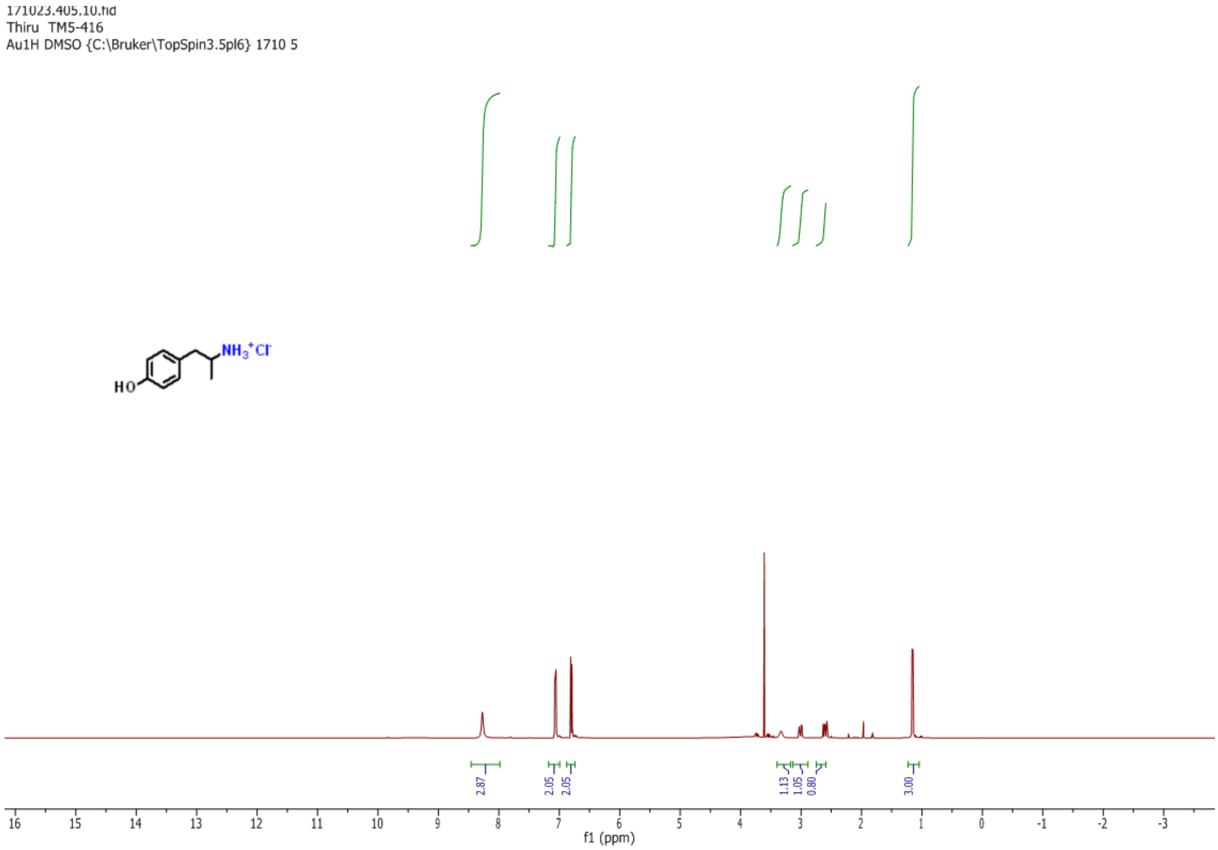

Supplementary Figure 127. ^{1}H NMR spectrum

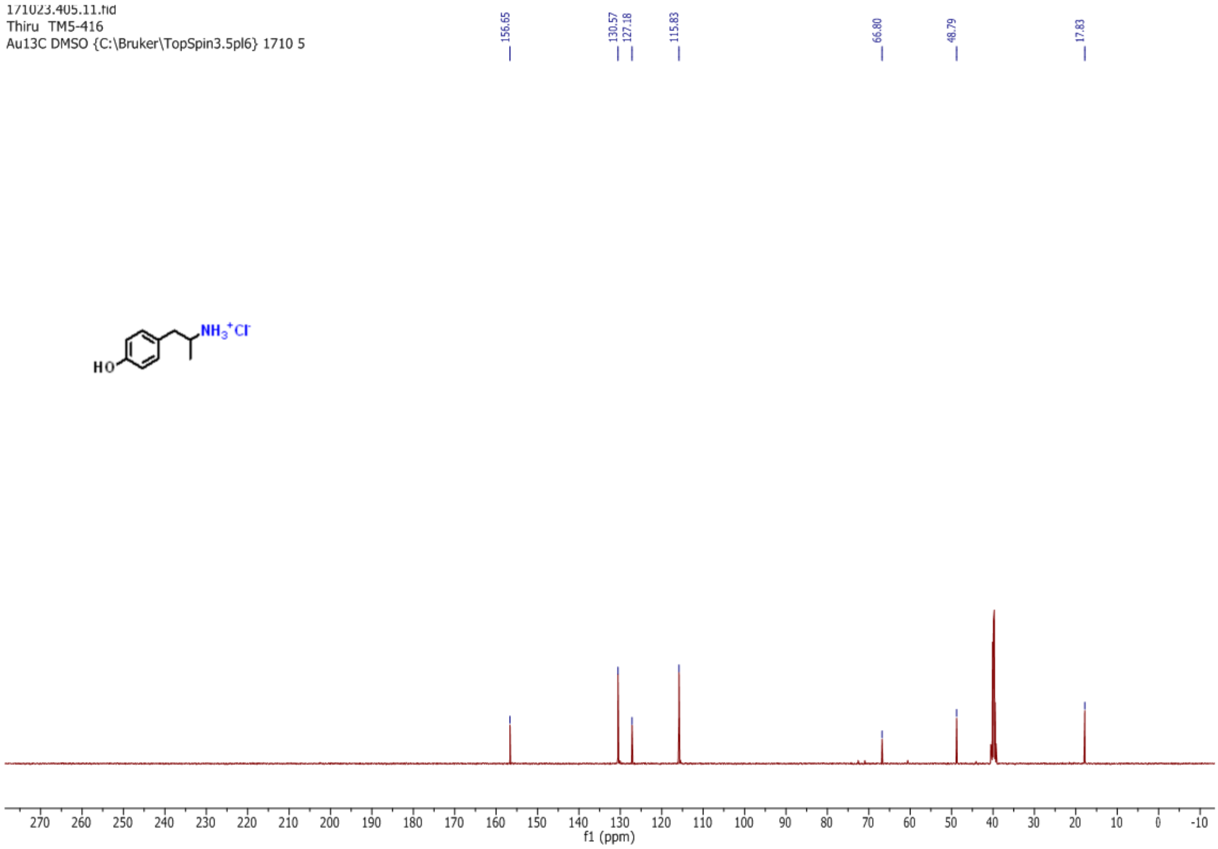

Supplementary Figure 128. ^{13}C NMR spectrum

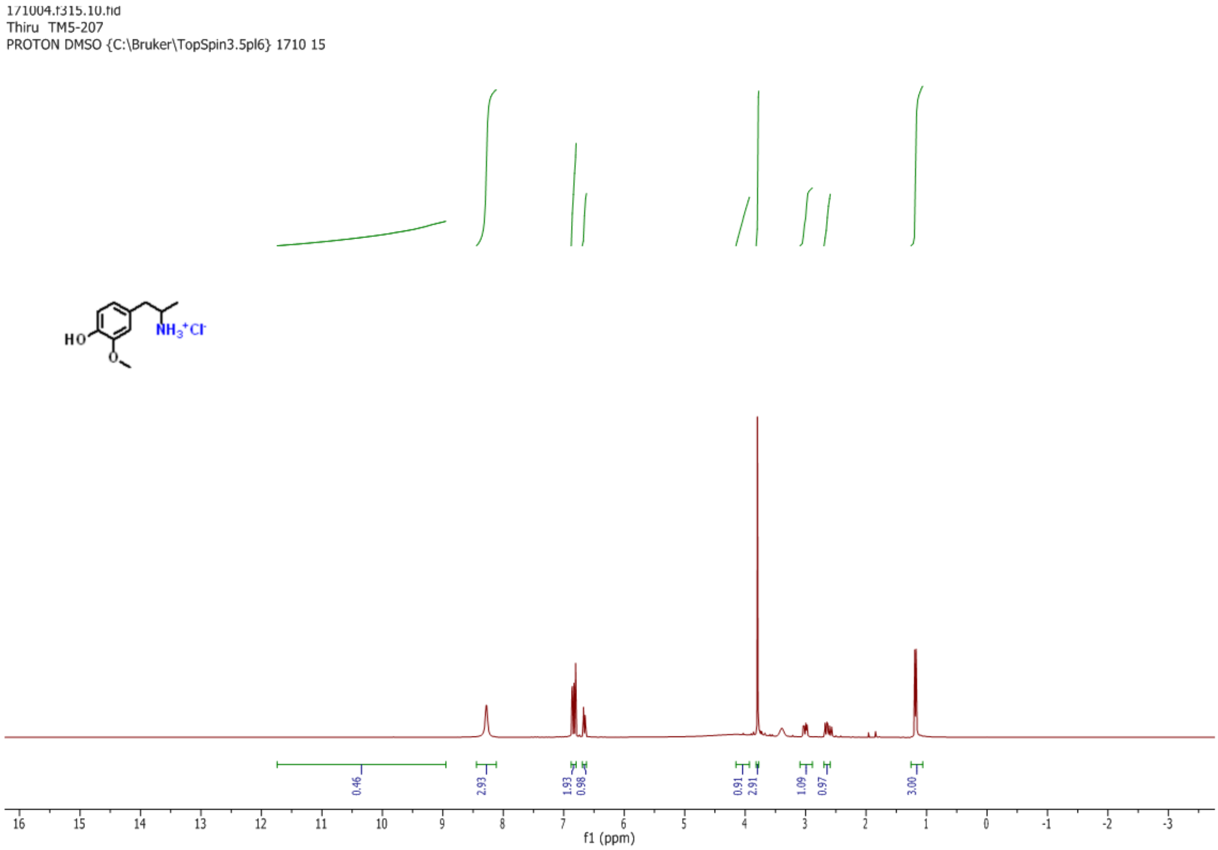

Supplementary Figure 129. ^{1}H NMR spectrum

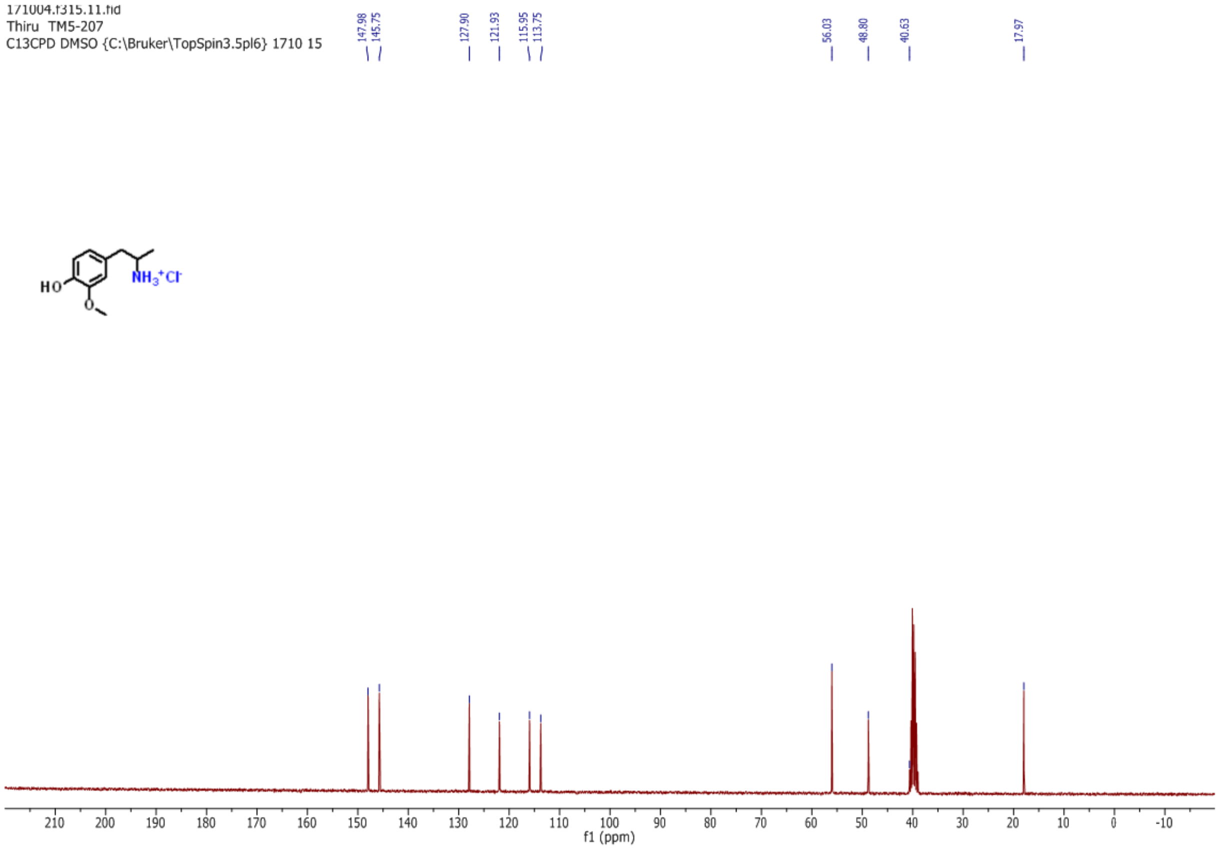

Supplementary Figure 130. ^{13}C NMR spectrum

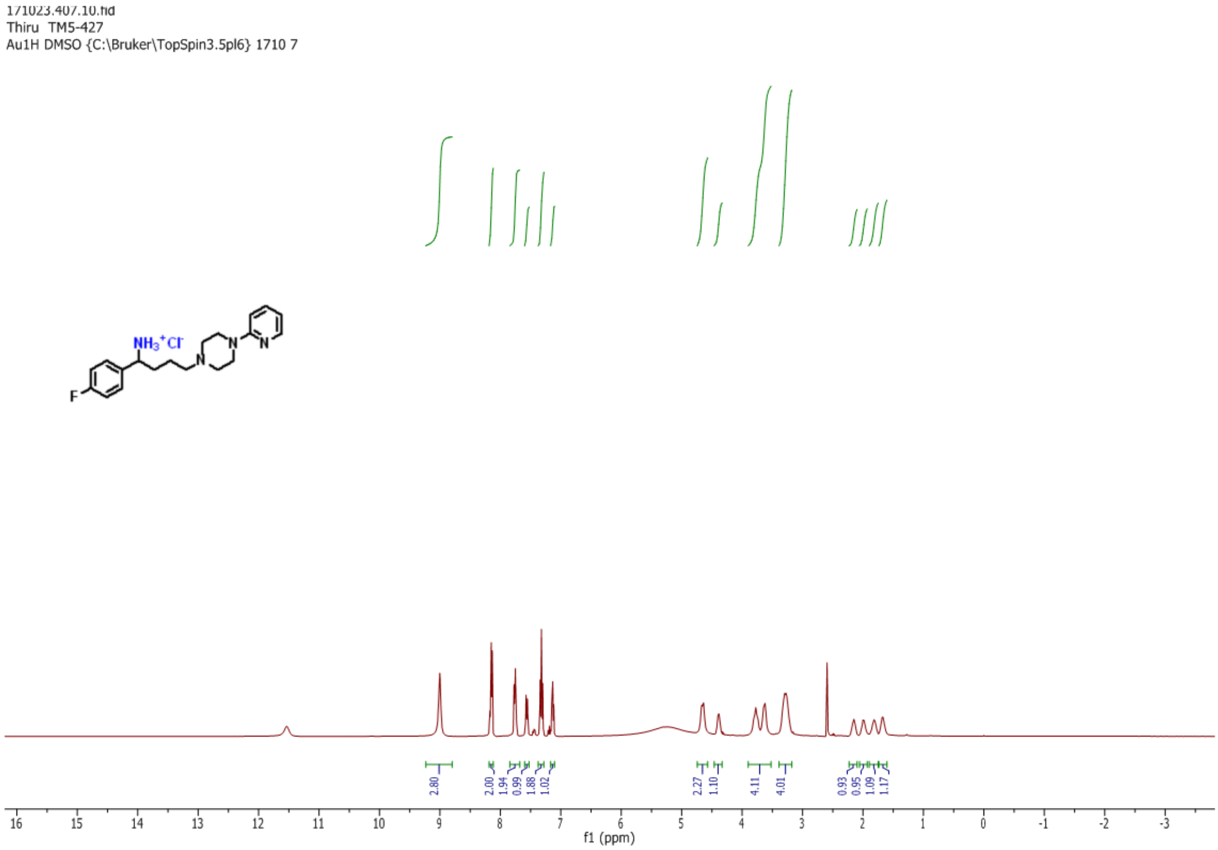

Supplementary Figure 131. ^{1}H NMR spectrum

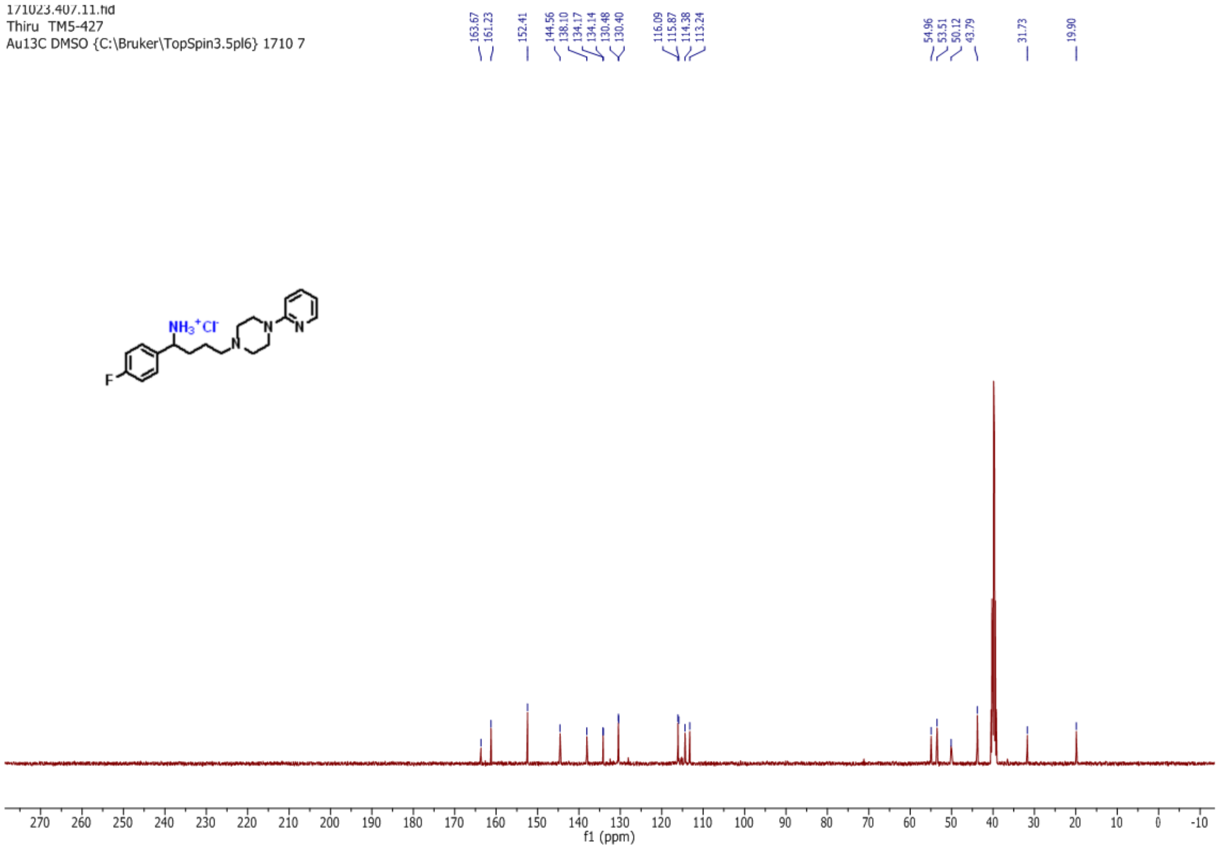

Supplementary Figure 132. ^{13}C NMR spectrum

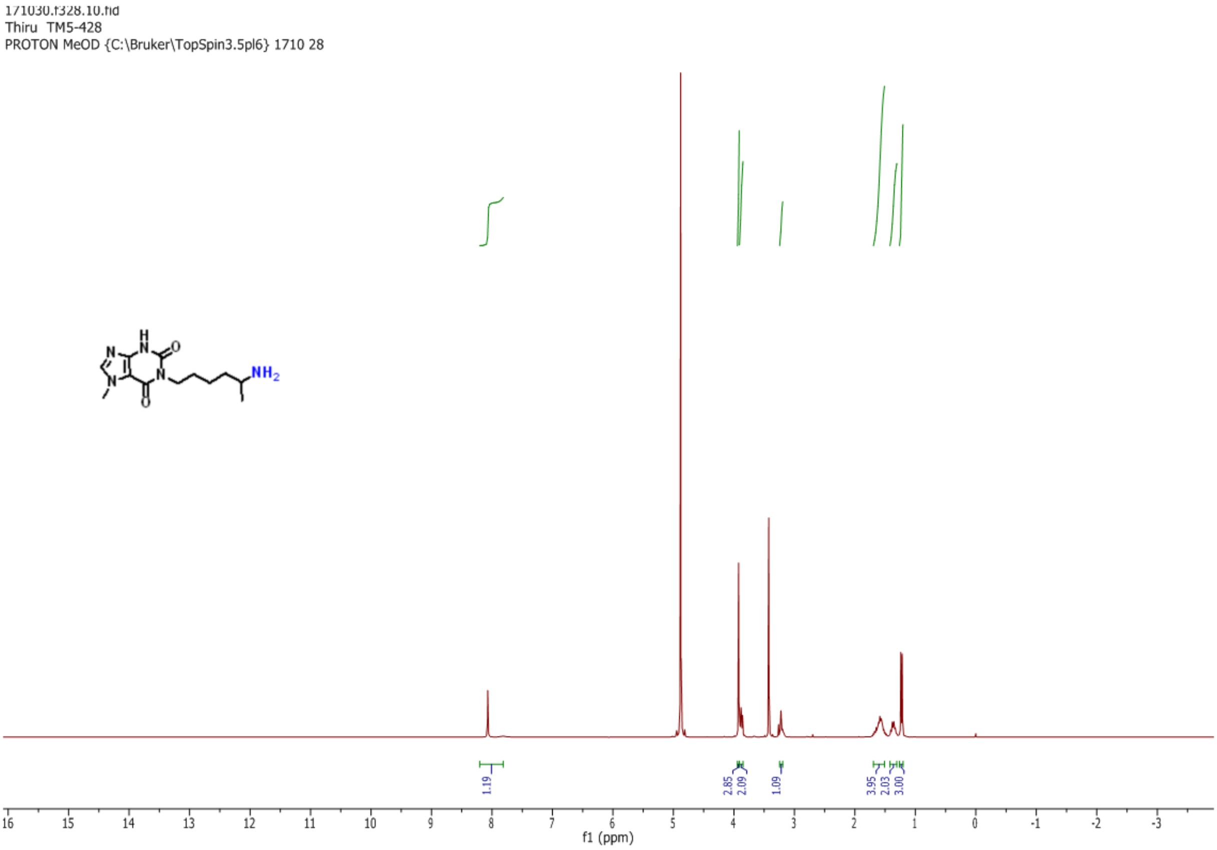

Supplementary Figure 133. ^{1}H NMR spectrum

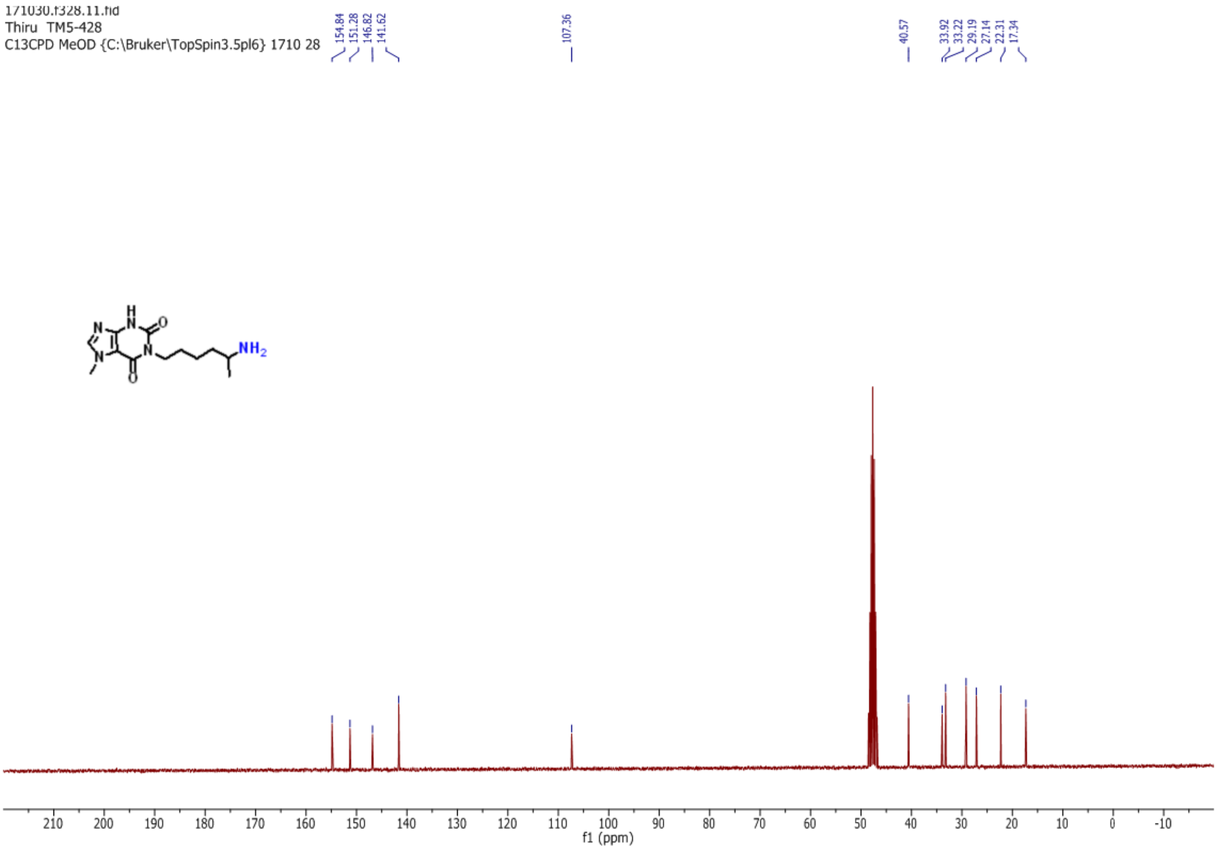

Supplementary Figure 134. ^{13}C NMR spectrum

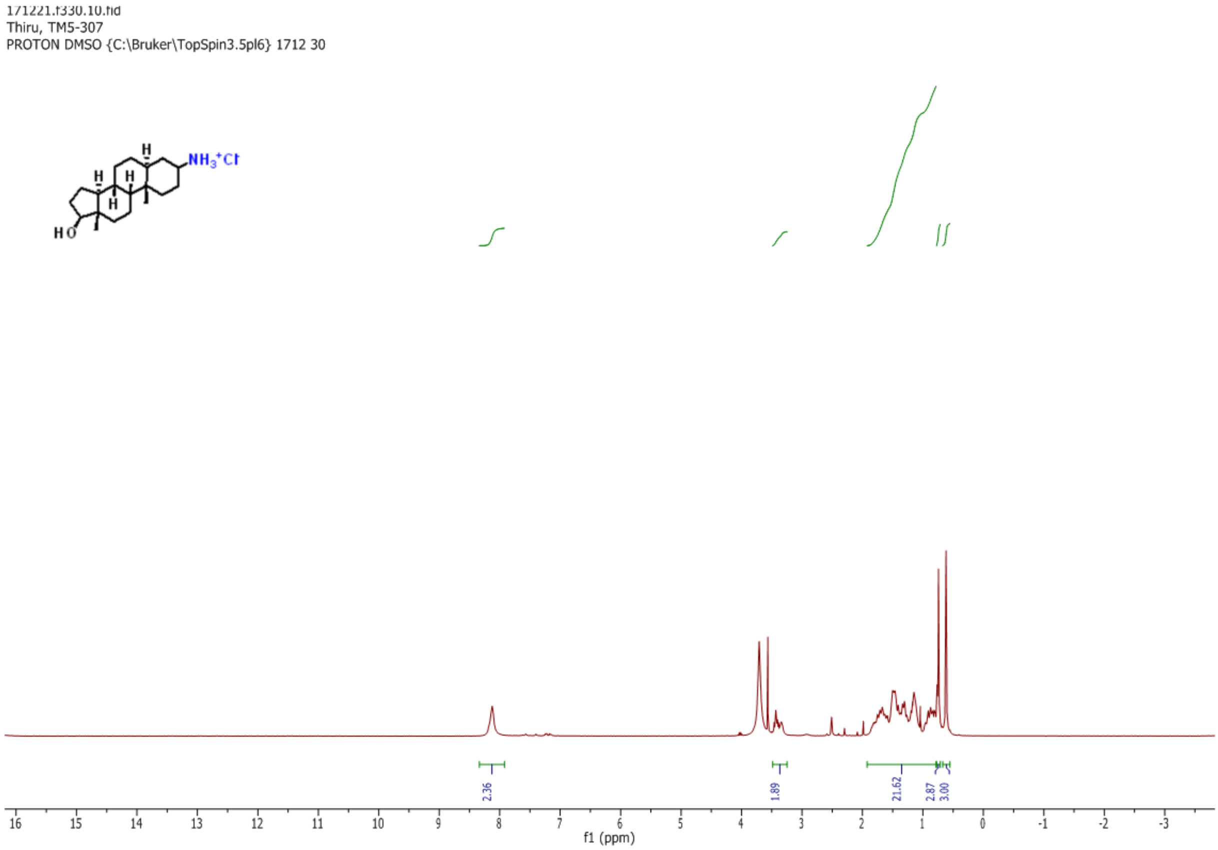

Supplementary Figure 135. ^{1}H NMR spectrum

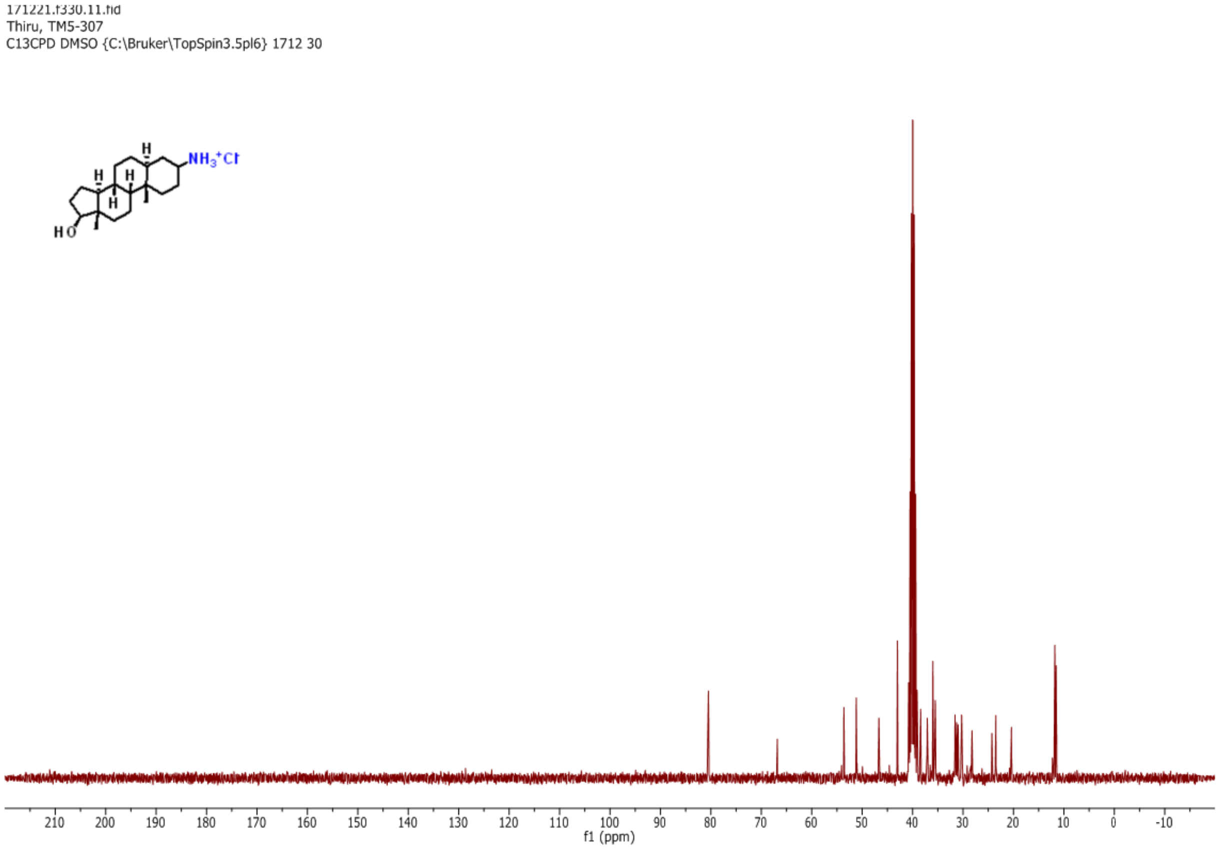

Supplementary Figure 136. ^{13}C NMR spectrum

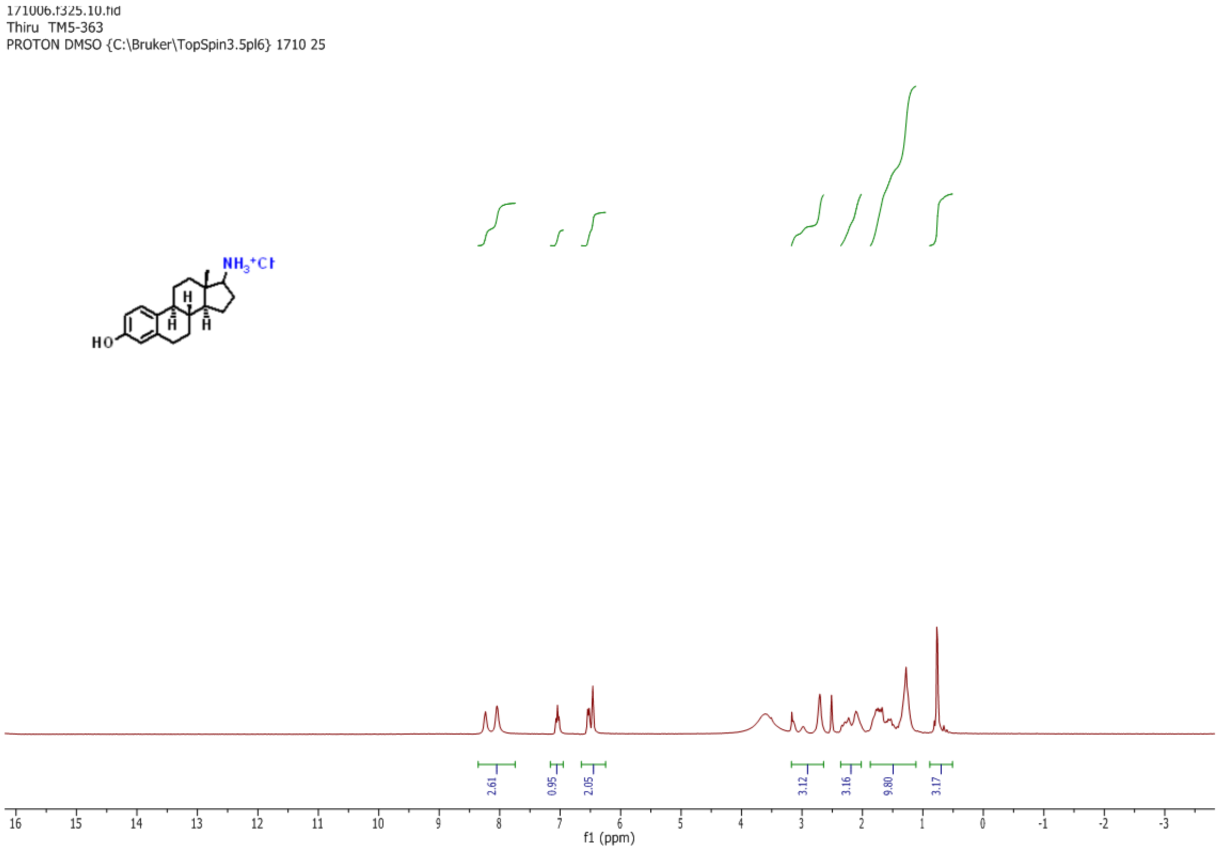

Supplementary Figure 137. ^{1}H NMR spectrum

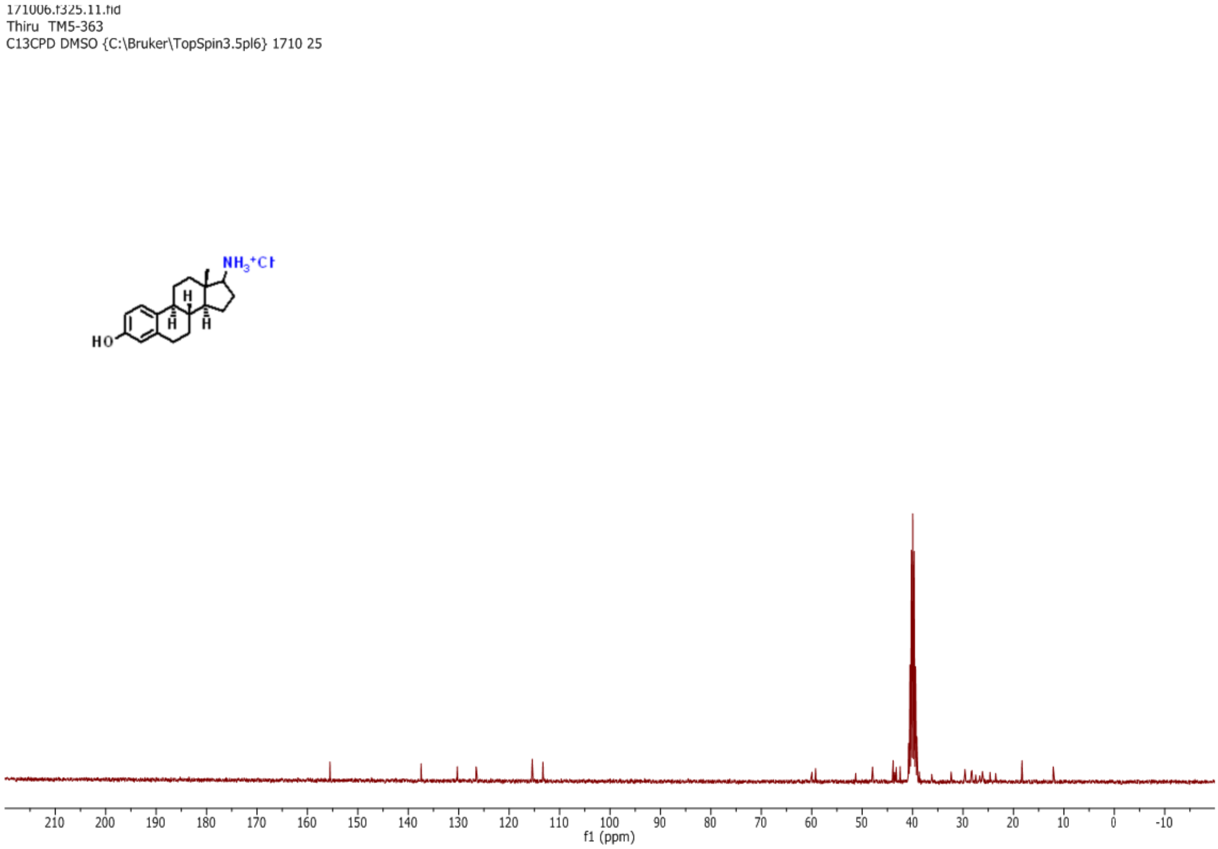

Supplementary Figure 138. ^{13}C NMR spectrum

170918.f325.10.fid
Thiru/ TM5-311
PROTON DMSO {C:\Bruker\TopSpin3.5pl6} 1709 25

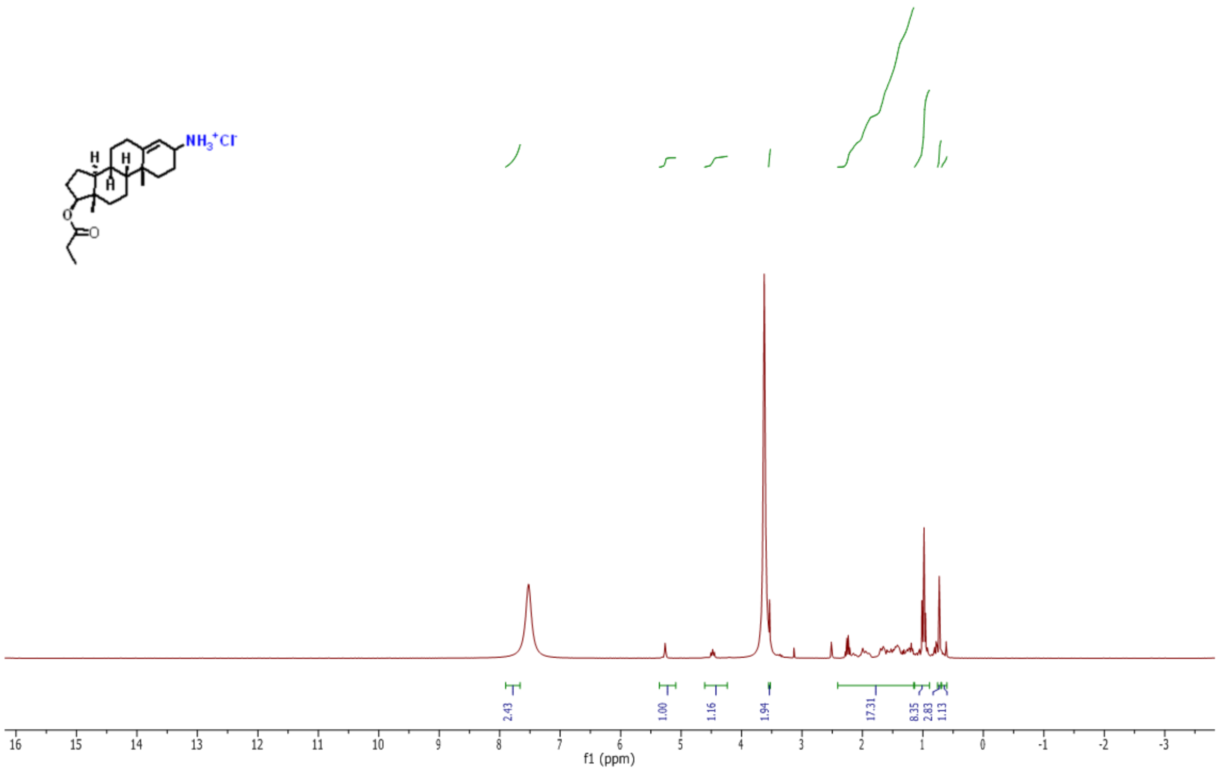

Supplementary Figure 139. ^{1}H NMR spectrum

170918.f325.11.fid
Thiru/ TM5-311
C13CPD DMSO {C:\Bruker\TopSpin3.5pl6} 1709 25

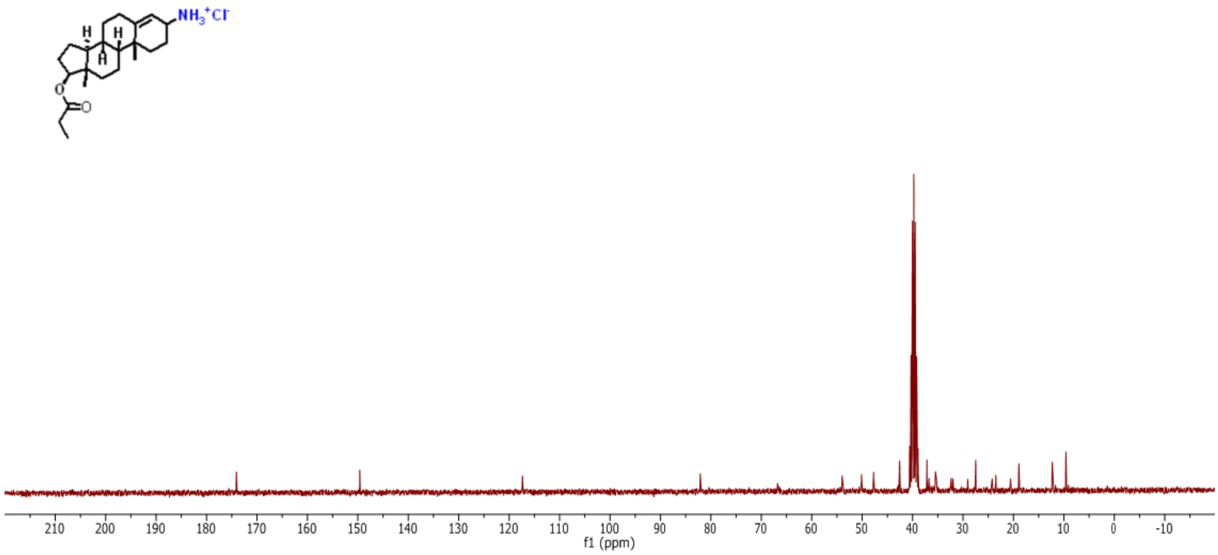

Supplementary Figure 140. ^{13}C NMR spectrum

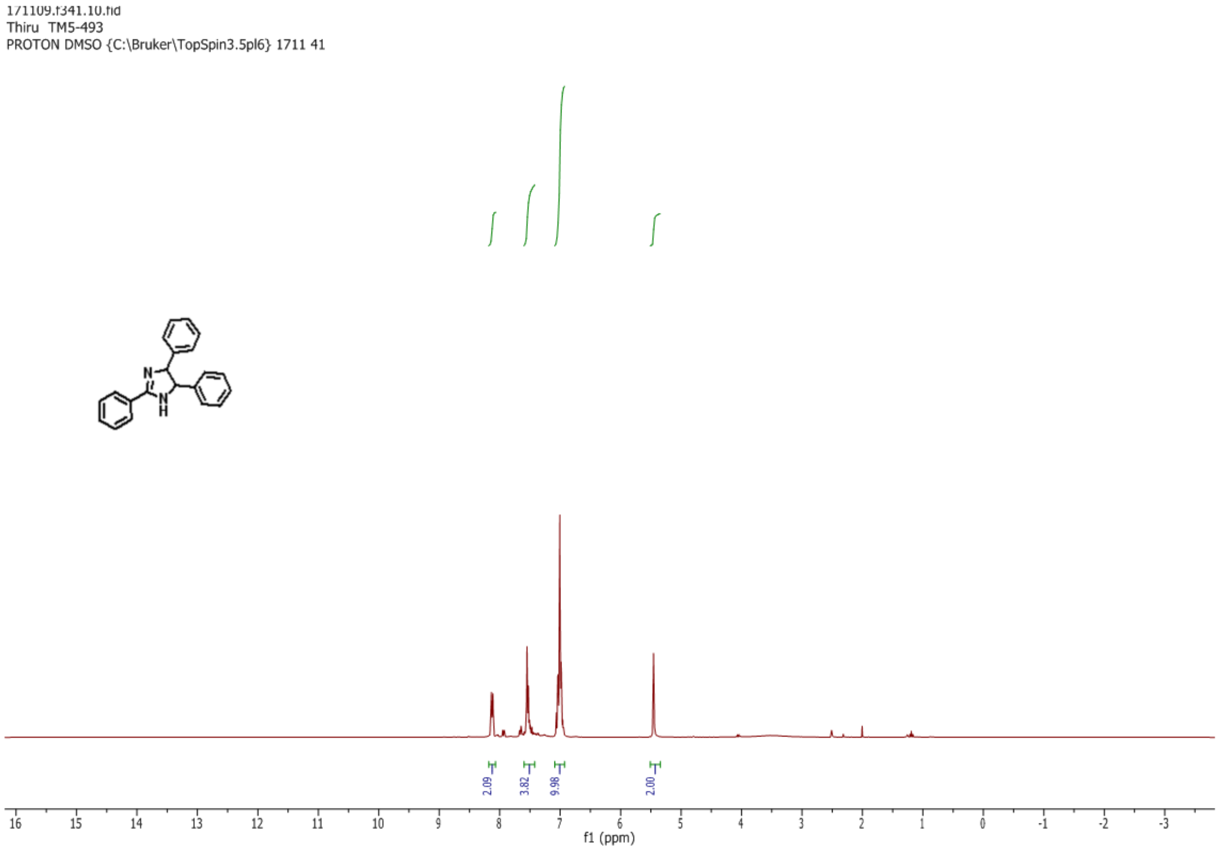

Supplementary Figure 141. ^{1}H NMR spectrum

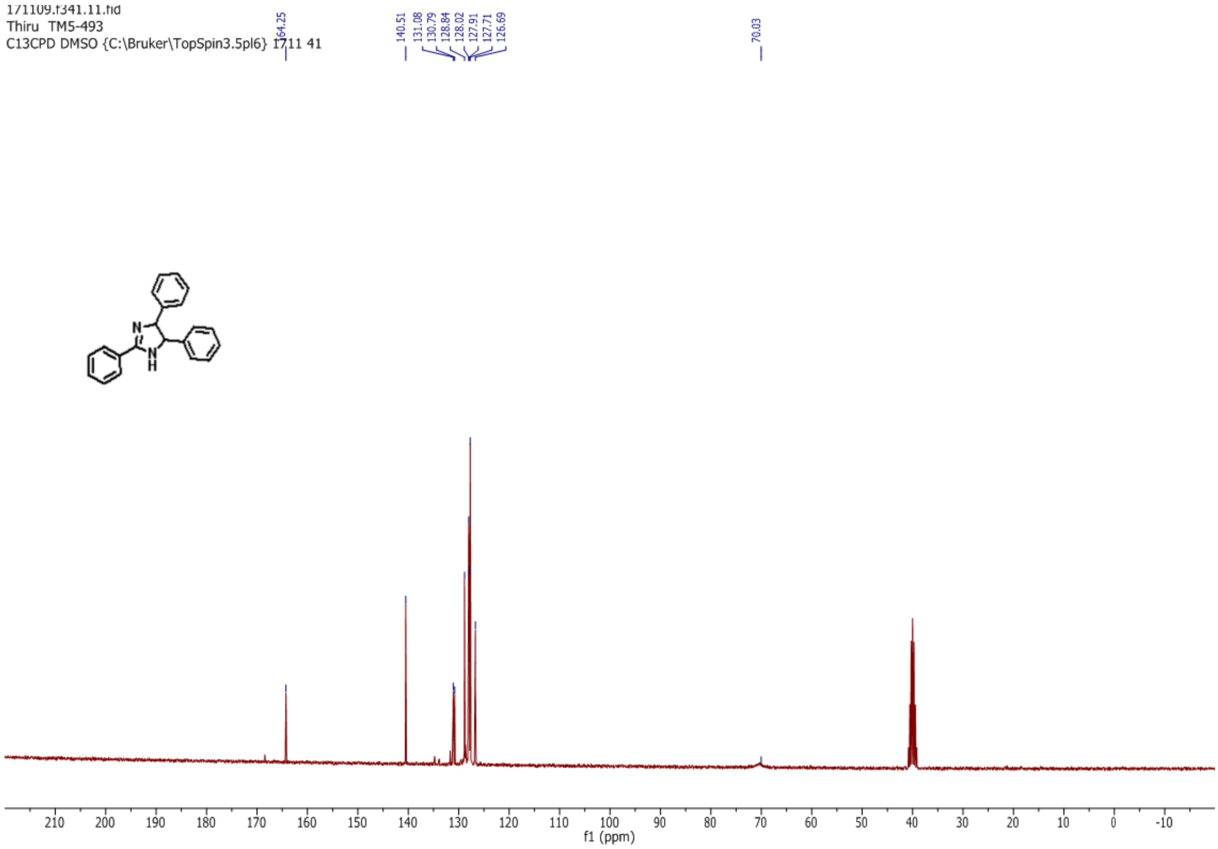

Supplementary Figure 142. ^{13}C NMR spectrum
